# Publisher Correction: Computation-guided asymmetric total syntheses of resveratrol dimers

**DOI:** 10.1038/s41467-022-30160-7

**Published:** 2022-04-27

**Authors:** Masaya Nakajima, Yusuke Adachi, Tetsuhiro Nemoto

**Affiliations:** Graduate School of Pharmaceutical Sciences, Chiba University, 1-8-1, Inohana, Chuo-ku, Chiba 260-8675 Japan

**Keywords:** Natural product synthesis, Density functional theory

Correction to: *Nature Communications* 10.1038/s41467-021-27546-4, published online 10 January 2022.

The original version of this Article included an incorrect Supplementary Information file, in which only the first 10 pages were present. The full Supplementary Information file is now available.

## Supplementary information

Updated Supplementary Information

