# Supplementary material for: Computation-guided asymmetric total syntheses of resveratrol dimers
Source: Nat Commun. 2022 Jan 10;13:152. doi: 10.1038/s41467-021-27546-4 (PMC8748746; doi:10.1038/s41467-021-27546-4)
Supplement: Supplementary file 1 — Supplementary Information [file 41467_2021_27546_MOESM1_ESM.pdf]

Supplementary Information

Computation-Guided Asymmetric Total Syntheses Resveratrol Dimers

Masaya Nakajima*[1], Yusuke Adachi[1], Tetsuhiro Nemoto*[1]

[1]Graduate School of Pharmaceutical Sciences, Chiba University, 1-8-1, Inohana, Chuo-ku, Chiba 260-8675, Japan.

E-mail: m.nakajima@chiba-u.jp (MN), tnemoto@faculty.chiba-u.jp (TN)

Table of Contents

I. Supplementary Methods…………S-3

1. **General Methods …………S-3**

2. **Computational details…………S-4**

3. **Experimental procedure for the synthesis of Malibatol A, Vaticahainols and Hopeahainol A…………S-16**

4. **Experimental procedure of kinetic resolution…………S-32**

5. **Data of (+)- and (-)-Malibatol A and Vaticahainols…………S-35**

6. **^{1}H and ^{13}C NMR Spectra of new compounds…………S-38**

7. **Structural figures of X-ray crystallographic analysis…………S-50**

II. Supplementary References…………S-52

I. Supplementary Methods

1.General Methods

All calculations were performed with Gaussian 16 program. Structure optimizations were carried out at 298.15 K, using the ωB97X-D functional with an ultrafine grid and the LANL2DZ (for Fe) and 6-31G(d,p) (for other atoms) basis sets. All the biosynthetic calculations were performed in water solvent using the solvation model based on density (SMD). The other calculations were performed in gas phase. Harmonic vibrational frequencies were computed at the same level of theory to confirm no imaginary vibration was observed for the optimized structure, and only one imaginary vibration was observed for the transition state. The intrinsic reaction coordinate (IRC) method was used to track minimum energy paths from transition structures to the corresponding local minima.

NMR spectra were recorded on a JEOL eca 400, ecz 400, ecz 600, eca 600 spectrometer. Chemical shifts in $CDCl_3$, acetone-d_6, DMSO-d_6 or CD_3OD were reported downfield from TMS (= 0 ppm) or solvent signal [acetone-d_6 (= 2.04 ppm), DMSO-d_6 (= 2.49 ppm) or CD_3OD (= 3.30 ppm)] for ^1H NMR. Data are reported as follows: chemical shift, multiplicity (s = singlet, d = doublet, t = triplet, m = multiplet, and br = broad), integration and coupling constants in Hz. For ^{13}C NMR, chemical shifts were reported in the scale relative to the solvent signal [$CHCl_3$ (77.0 ppm), acetone-d6 (29.8 ppm), DMSO-d6 (39.5 ppm) or CD_3OD (49.0 ppm)] as an internal reference. ESI mass spectra were measured on JEOL AccuTOF LC-plus JMS-T100LP. Optical rotations were measured on a JASCO P-1020 polarimeter. The enantiomeric excess (ee) was determined by HPLC analysis. HPLC was performed on JASCO HPLC systems consisting of the following: pump, PU-980; detector, UV-970; column DAICEL CHIRALPAK OD-3; mobile phase, n-hexane/*i*-PrOH. Analytical thin layer chromatography was performed on Kieselgel 60F254, 0.25 mm thickness plates. Column chromatography was performed with silica gel 60 N (spherical, neutral 63-210 mesh). Reactions were conducted in dry solvent. Other reagents were purified by the usual methods.

2.Computational details

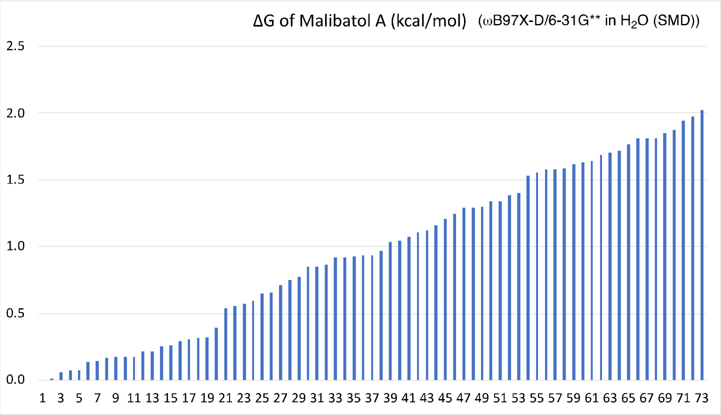

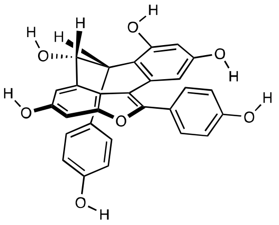

Global minimum of Malibatol A
(ωB97X-D/6-31G** in H_2O (SMD))

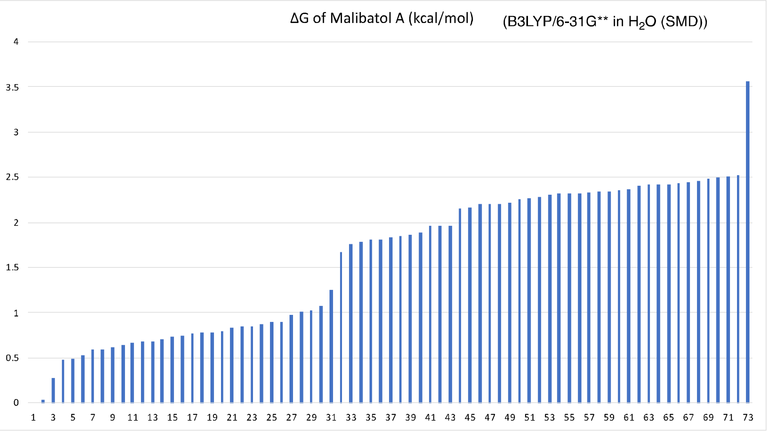

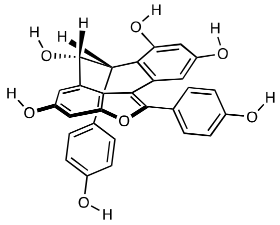

Global minimum of Malibatol A
(B3LYP/6-31G** in H_2O)

Supplementary Fig. 1. Conformational search of malibatol A

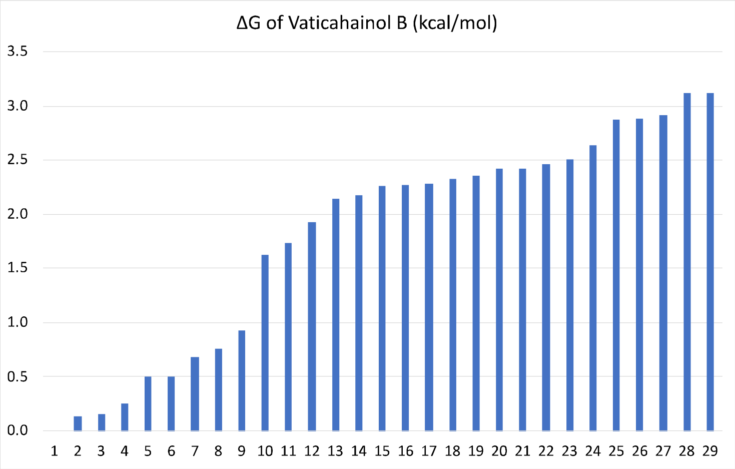

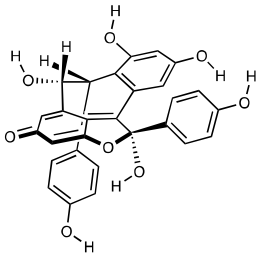

Global minimum of Vaticahainol B

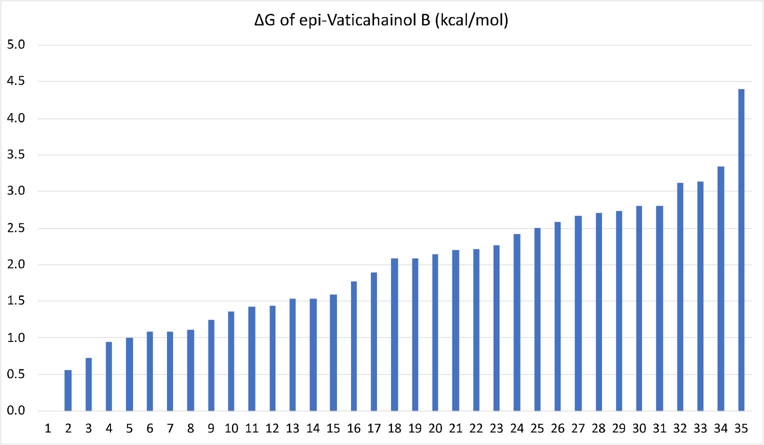

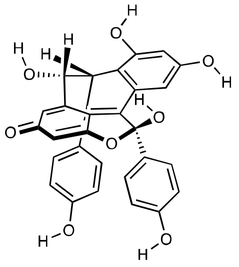

Global minimum of epi-Vaticahainol B

Supplementary Fig. 2. Conformational search of (epi-)-vaticahainol B

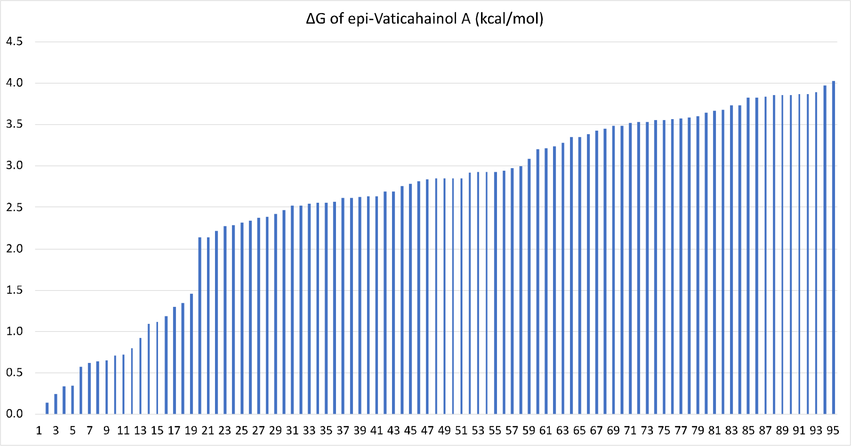

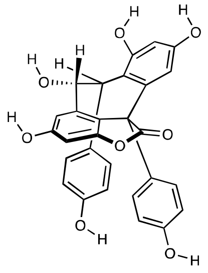

Global minimum of epi-Vaticahainol A

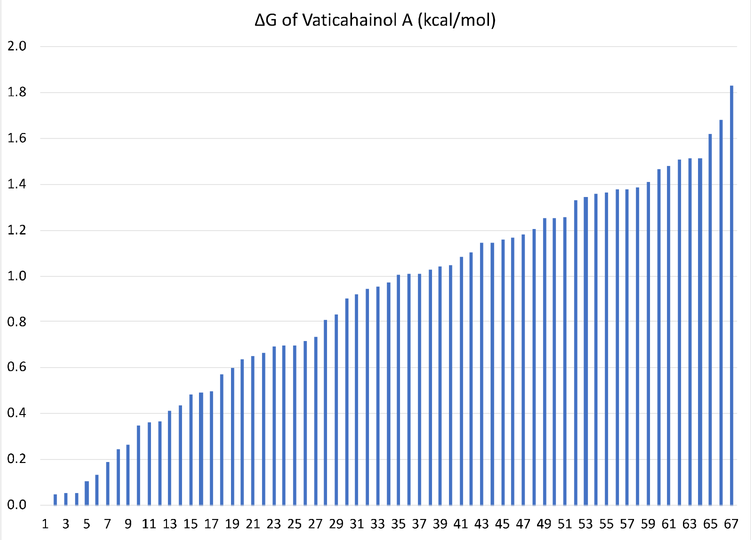

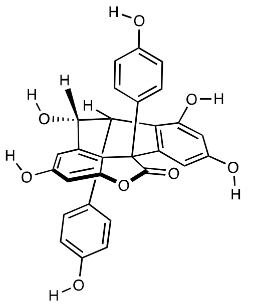

Global minimum of Vaticahainol A

Supplementary Fig. 3. Conformational search of (epi-)-vaticahainol A

A)

①
73 conformers generated by Spartan (MM)

②
Opt and Freq of all 73 conformers by DFT (wB97X-D/6-31G** in H_2O) with Gaussian16

③
Sort by G and determine a global minimum

④
Optimization with various functionals/basis sets using global minimum (wB97X-D/6-31G** in H2O) as initial geometry

⑤
Compared the optimized structure with the result of MP2/6-311G** in H_2O by RMSD

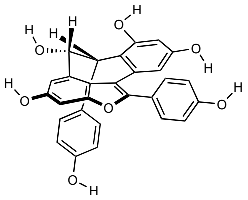

Global minimum of Malibatol A
(ωB97X-D/6-31G** in H_2O)

Method	ab initio (MP2)	DFT												
Functional		B3LYP	B3PW91	B98	BLYP	cam-B3LYP	mPW1PW91	PBEPBE	PBE1PBE	M06	M06-L	M06-2X	wB97	wB97X-D
Basis set	6-311G**	6-31G**												
RMSD (Å)	0.00	0.37	0.35	0.34	0.40	0.34	0.32	0.34	0.31	0.29	0.20	0.19	0.26	0.17

Method	ab initio (MP2)	DFT (wB97X-D)										
Basis set	6-311G**	STO-3G	6-31G*	6-31G**	6-31+g**	6-311g**	6-311+g**	cc-pvDZ	aug-cc-pvDZ	cc-pvTZ	def2SVP	def2TZVP
RMSD (Å)	0.00	0.21	0.17	0.17	0.16	0.15	0.14	0.19	0.14	0.20	0.17	0.19

B)

①
73 conformers generated by Spartan (MM)

②
Opt and Freq of all 73 conformers by DFT (B3LYP/6-31G** in H_2O) with Gaussian16

③
Sort by G and determine a global minimum

④
Optimization with various functionals/basis sets using global minimum (B3LYP/6-31G** in H2O) as initial geometry

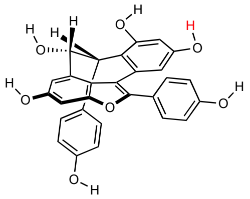

Global minimum of Malibatol A
(B3LYP/6-31G** in H_2O)

⑤
Compared the optimized structure with the result of MP2/6-311G** in H_2O by RMSD

Method	ab initio (MP2)	DFT												
Functional		B3LYP	B3PW91	B98	BLYP	cam-B3LYP	mPW1PW91	PBEPBE	PBE1PBE	M06	M06-L	M06-2X	wB97	wB97X-D
Basis set	6-311G**	6-31G**												
RMSD (Å)	0.00	0.37	0.35	0.34	0.40	0.33	0.33	0.34	0.31	0.29	0.20	0.19	0.26	0.17

Supplementary Fig. 4. Benchmarking study of functional/basis set.

Opt (SMD in water)	Functional	wB97X-D							
	Basis set	6-31G**							
SP (SMD in water)	Method	ab initio (MP2)				–	DFT (wB97X-D)		
	Basis set	6-311+G**	cc-pvTZ	def2TZVP	average	–	6-311+G**	cc-pvTZ	def2TZVP
ΔE (Vaticahainol A - epi-Vaticahainol A) (kcal/mol)		-2.1	-2.4	-2.6	-2.4	-2.3	-3.1	-3.3	-3.4
ΔE (Vaticahainol B - epi-Vaticahainol B) (kcal/mol)		2.3	2.3	2.2	2.3	2.7	2.0	1.9	1.7
Mean absolute error (kcal/mol)					0.0	0.3	0.5	0.7	0.8

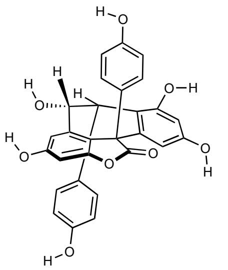

Global minimum of Vaticahainol A

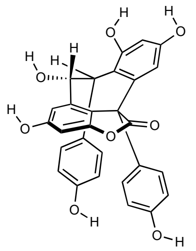

Global minimum of epi-Vaticahainol A

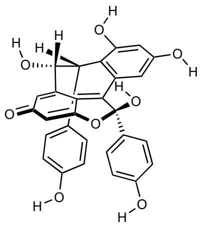

Global minimum of epi-Vaticahainol B

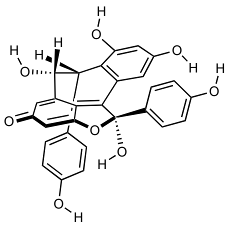

Global minimum of Vaticahainol B

Supplementary Fig. 5. Benchmarking study of single point energy calculation with ωB97X-D/triple

zeta basis sets.

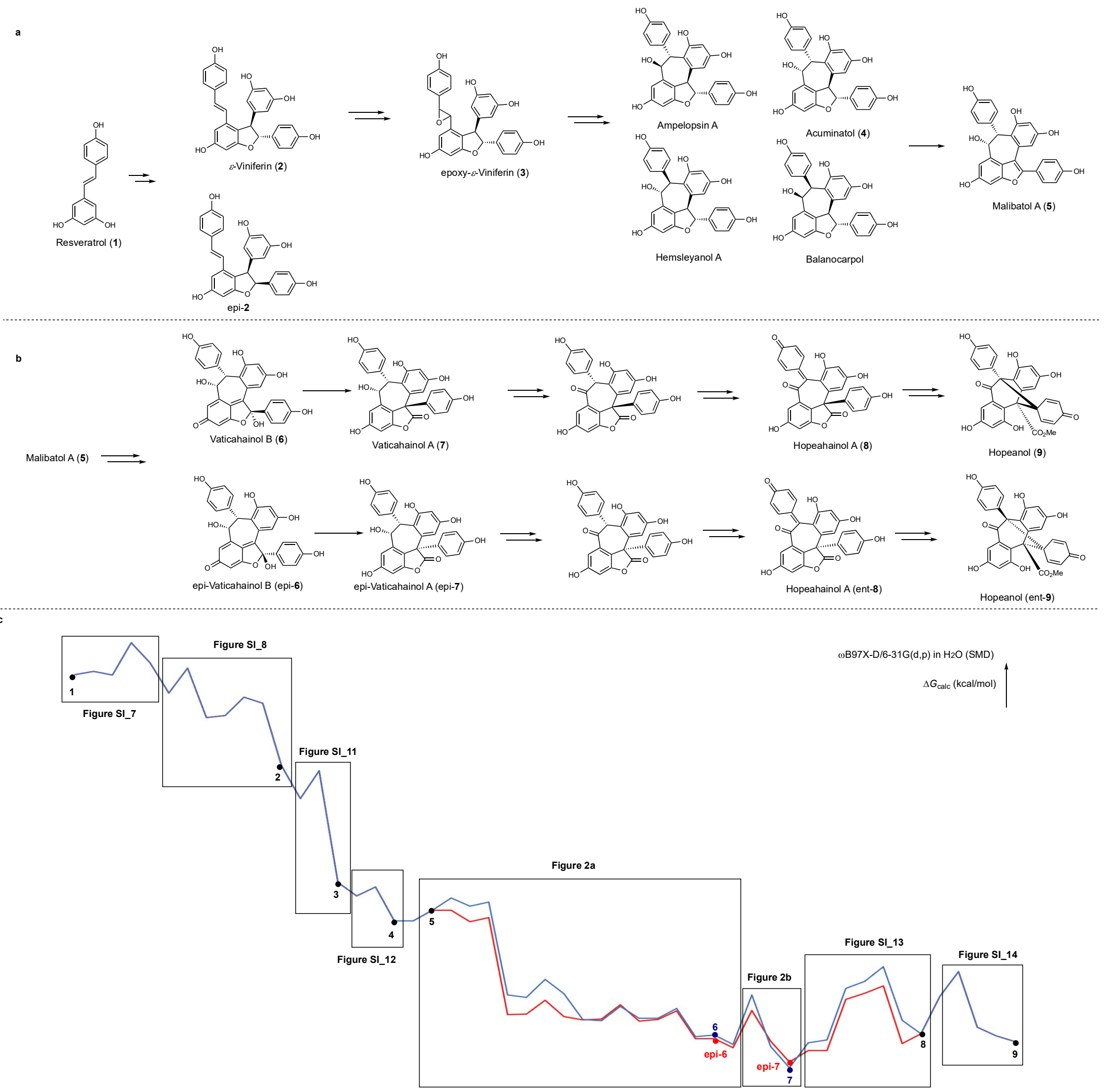

Supplementary Fig. 6. ab) Biosynthetic pathway of resveratrol dimers. c) Index of the figures of energy diagram.

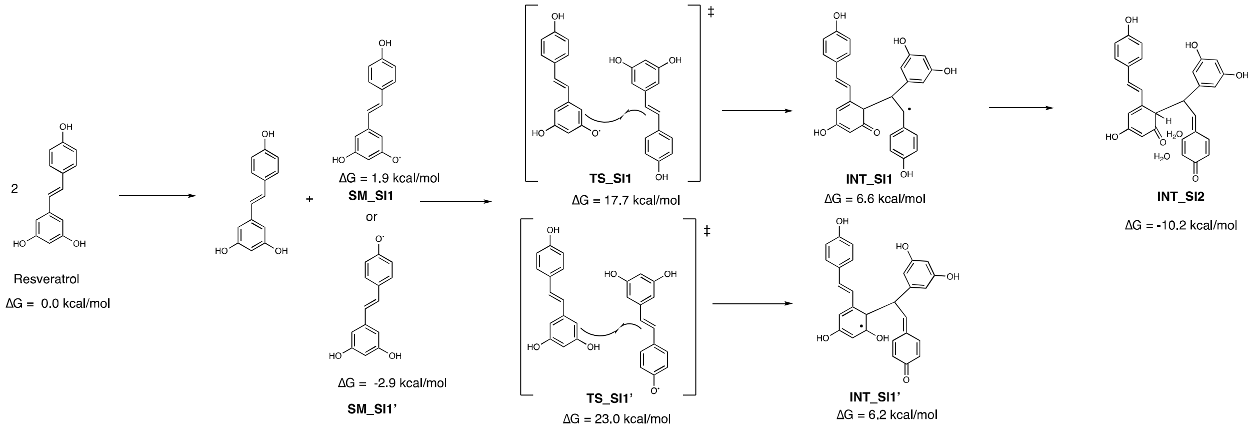

Supplementary Fig. 7. Radical dimerization of resveratrol. The energy of hydrogen radical was approximated from the energy difference between HOOH and •OOH.

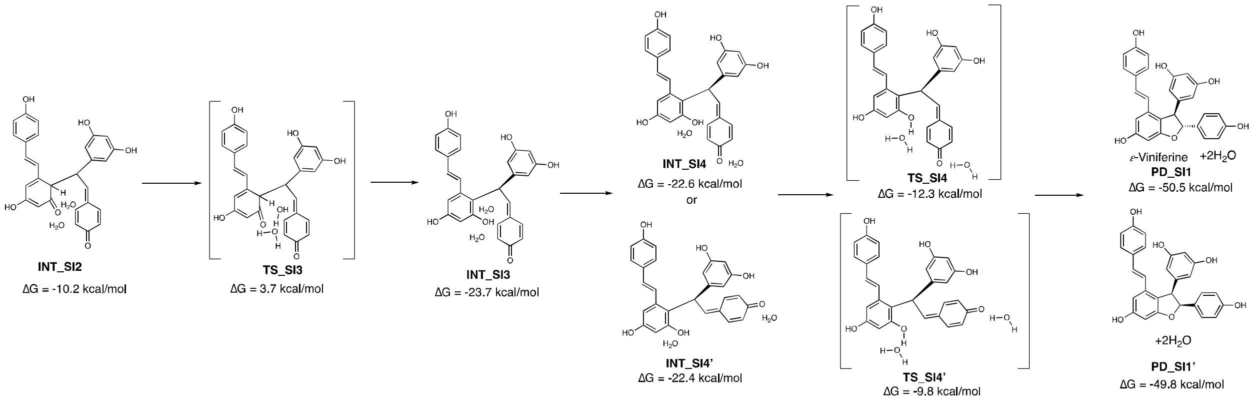

Supplementary Fig. 8. Aromatization and cyclization to form ε-viniferin and its diastereomer.

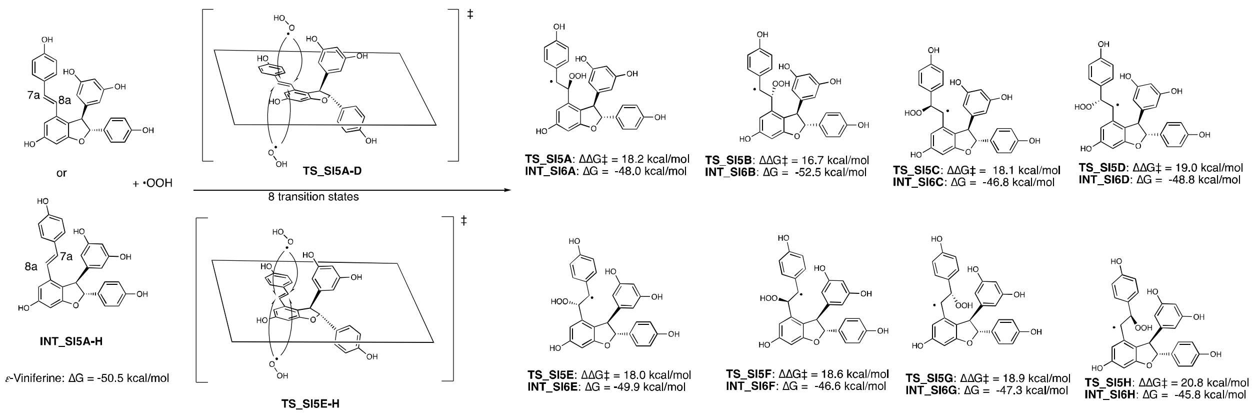

Supplementary Fig. 9. Addition of hydroperoxy radical to *ε*-viniferin.

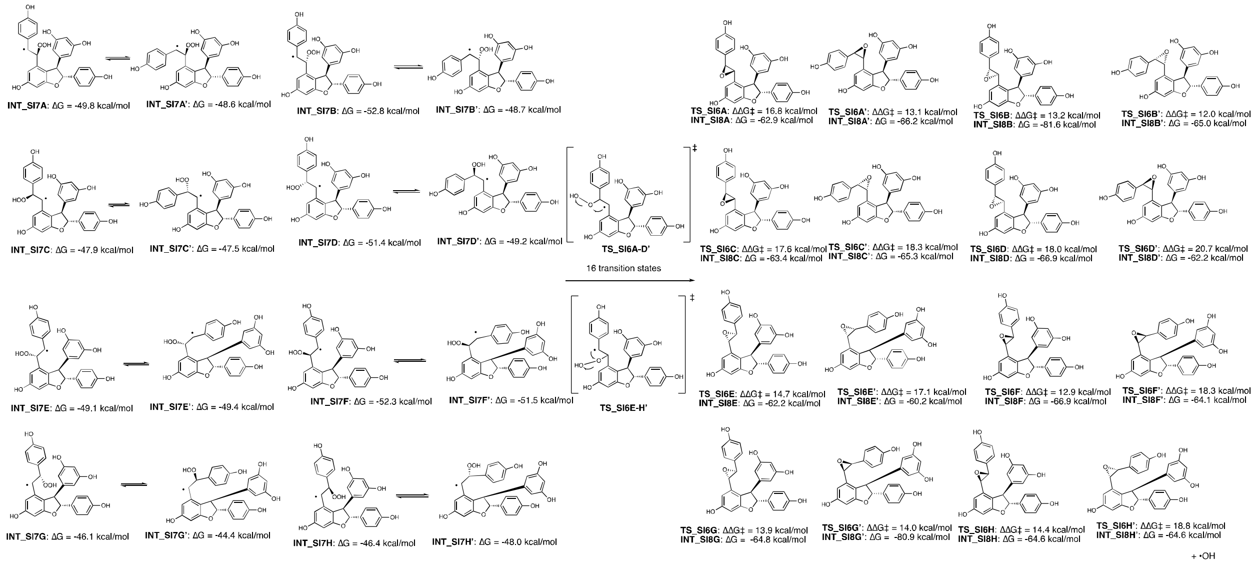

Supplementary Fig. 10. Epoxidation.

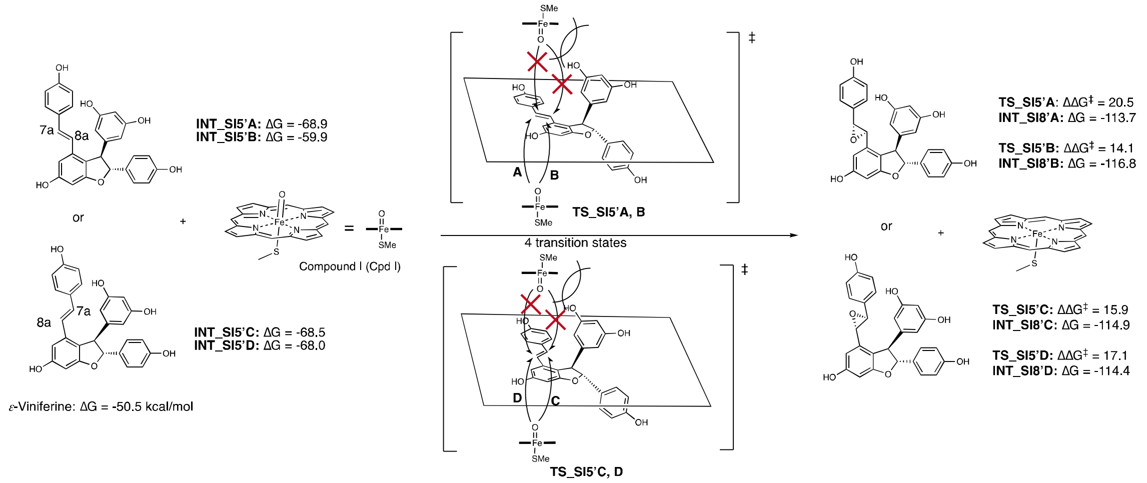

Supplementary Fig. 11. Epoxidation by Cpd I. Elimination of Cpd I to afford epoxide spontaneously proceeded during the optimization process after the IRC calculation.

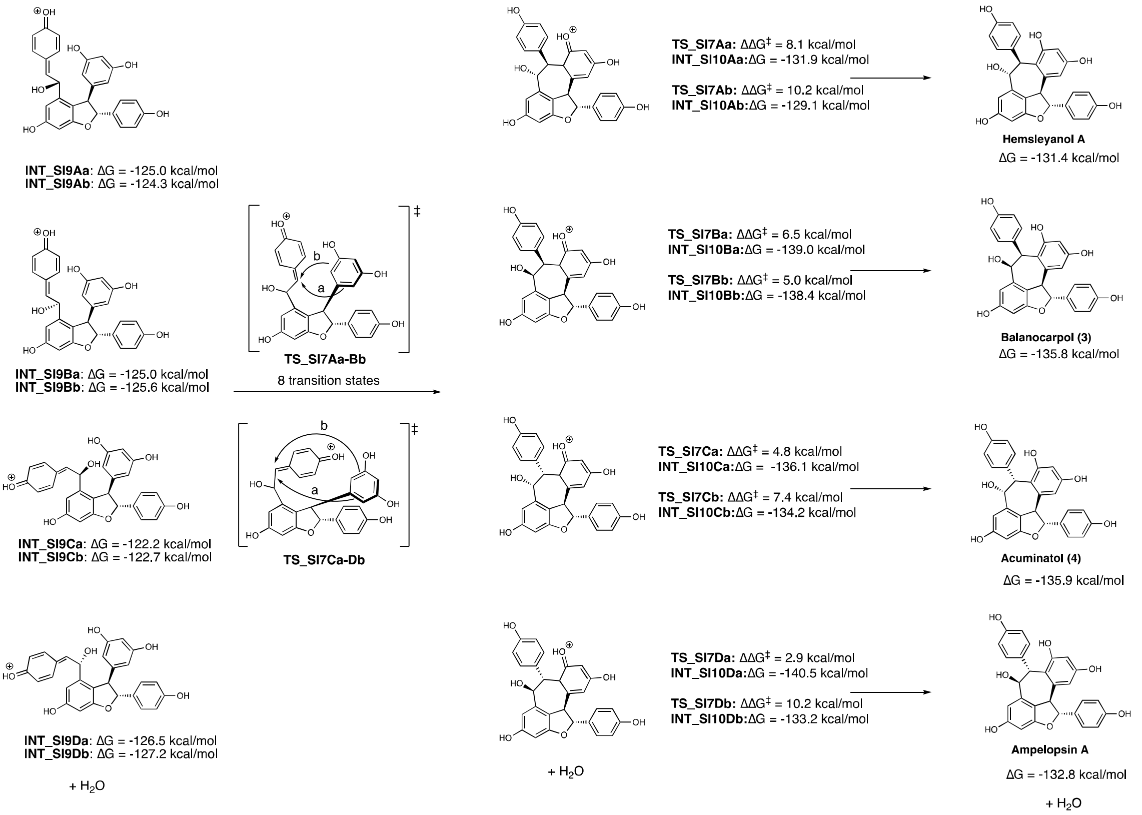

Supplementary Fig. 12. Intramolecular Friedel-Crafts type cyclization.

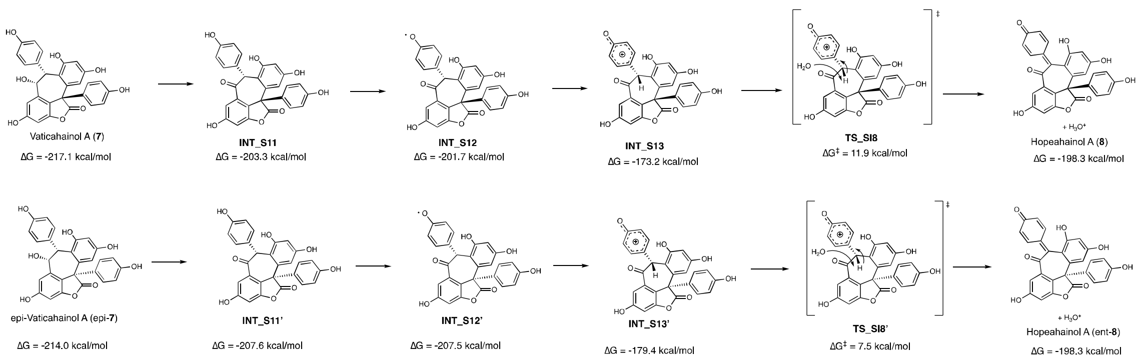

Supplementary Fig. 13. Energy diagram of the oxidation from (epi-)vaticahainol A to hopeahainol A.

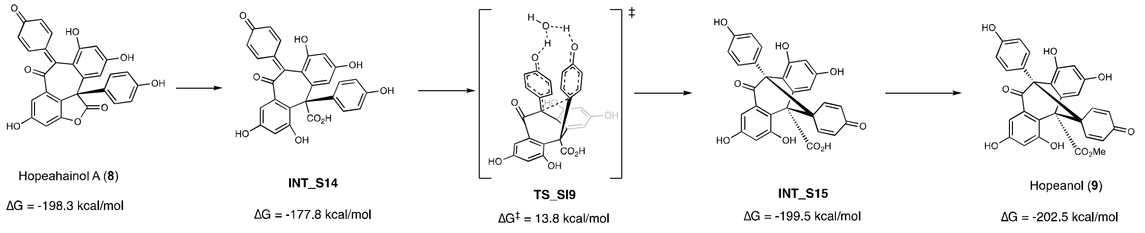

Supplementary Fig. 14. Transformation from hopeahainol A to hopeanol

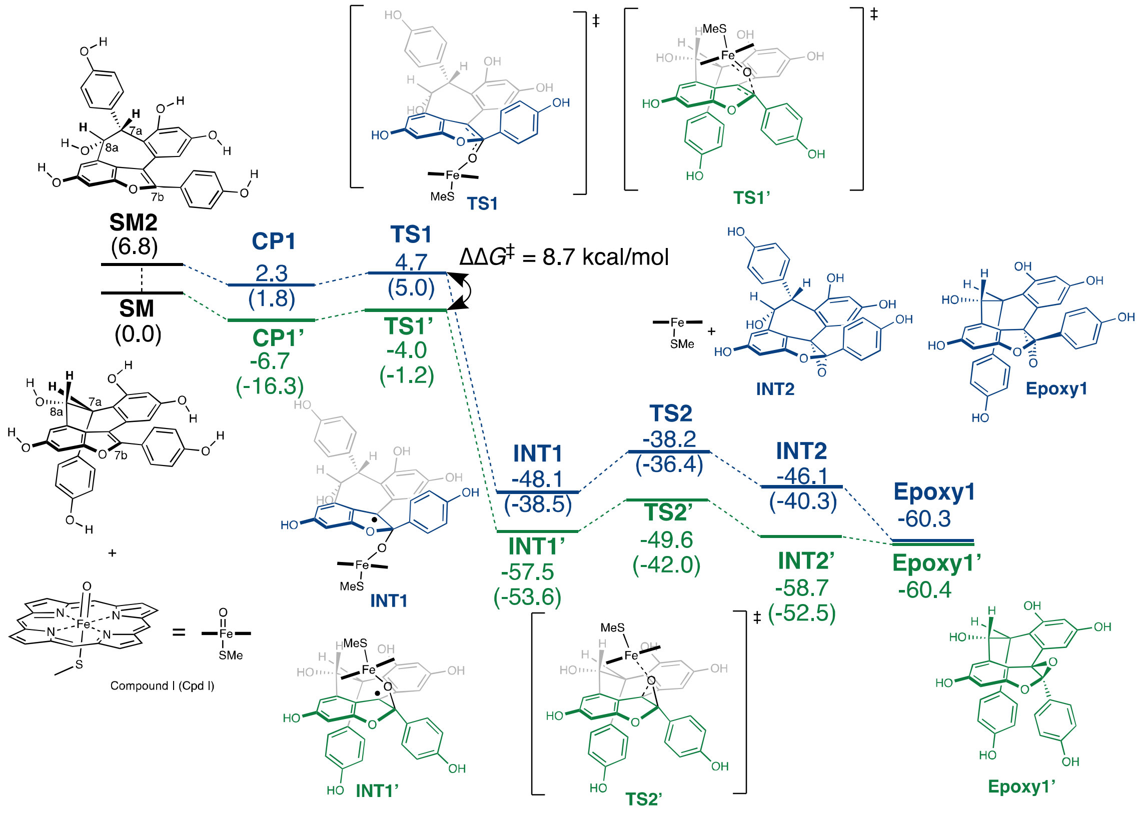

Supplementary Fig. 15. Epoxidation pathways with Cpd I. The numbers in parentheses are the energy of the quartet-state

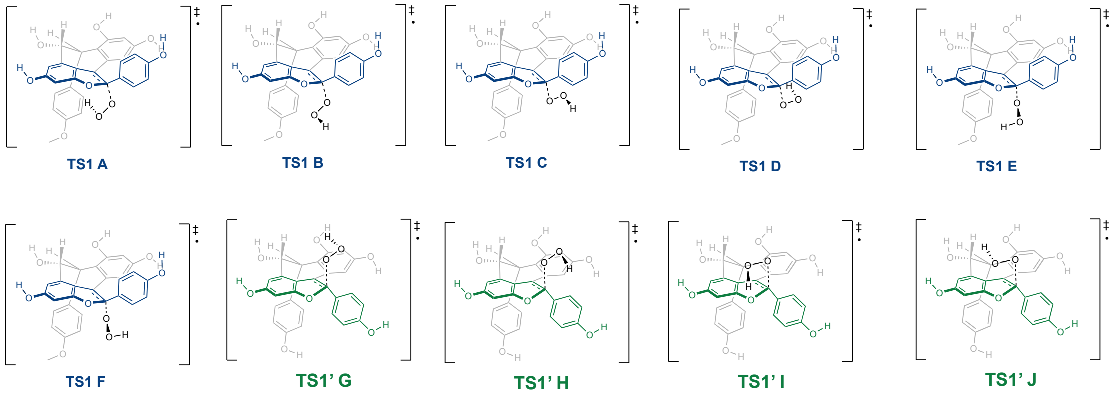

num	Opt/Freq		TS1 (ΔG)						TS1' (ΔG)				ΔΔG‡
	Functional	Basis set	A	B	C	D	E	F	G	H	I	J	(ΔGTS1 - ΔGTS1')
1	wB97X-D	6-31G**	0.6	2.6	5.4	3.4	3.6	3.8	2.3	1.4	0.0	0.2	0.9
2	wB97		0.4	2.3	5.6	3.8	3.9	3.7	2.5	1.7	0.0	0.5	0.7
3	B3LYP		2.6	3.3	5.6	4.4	3.9	3.6	1.9	1.1	0.1	0.0	2.8
4	B98		3.2	4.1	6.2	5.4	5.0	5.1	2.9	2.2	1.3	0.0	3.1
5	BLYP		3.0	4.2	6.0	5.1	4.2	4.3	2.2	1.3	0.6	0.0	3.1
6	B3PW91		2.7	3.7	6.2	4.8	4.4	4.1	2.2	1.6	0.7	0.0	2.7
7	cam-B3LYP		2.1	2.9	5.3	4.1	3.9	4.0	2.0	1.4	0.7	0.0	2.1
8	mPW1PW91		1.7	2.4	5.3	4.3	3.9	3.6	1.7	1.0	0.0	0.3	1.9
9	M06		1.0	2.0	5.6	4.2	3.6	3.5	1.4	1.0	0.0	0.5	1.2
10	M06-L		1.5	2.6	6.3	5.6	3.7	3.4	2.3	1.4	0.3	0.0	1.7
11	M06-2X		0.0	2.4	4.3	3.6	4.3	4.2	3.1	2.4	0.4	1.0	-0.2
12	PBEPBE		1.1	3.2	5.0	3.7	3.4	3.5	1.7	1.2	0.4	0.0	1.4
13	PBE1PBE		1.9	3.5	5.9	4.8	4.6	4.5	2.5	1.6	0.1	0.0	2.3
14	HF		4.2	4.9	6.5	5.4	3.7	3.8	6.7	2.9	0.0	0.1	3.5
15	wB97X-D	def2SVP	0.2	2.4	5.2	3.8	4.1	3.9	2.5	1.6	0.4	0.0	0.5
16	wB97X-D	cc-pvDZ	0.8	2.8	5.9	4.0	3.6	3.9	2.9	1.2	0.3	0.0	1.1

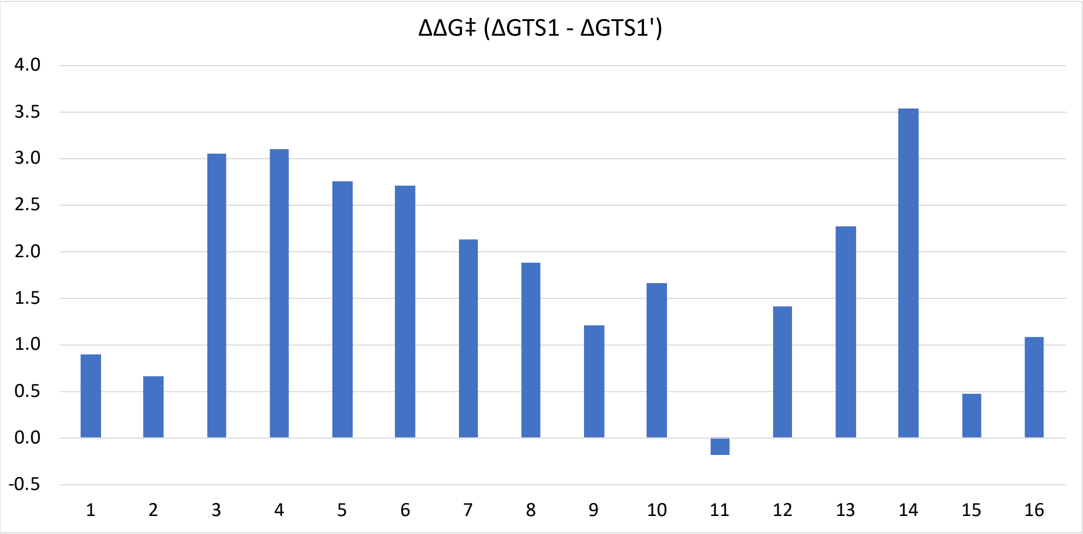

Supplementary Fig. 16. Non-enzymatic epoxidation pathways with hydroperoxy radical.

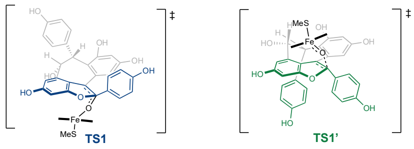

num	Opt/Freq		TS1	TS1'
	Functional	Basis set	ΔG	ΔG
1	wB97X-D	6-31G**	8.7	0.0
2	wB97		7.7	0.0
3	B3LYP		6.1	0.0
4	B98		5.6	0.0
5	BLYP		5.8	0.0
6	B3PW91		5.9	0.0
7	cam-B3LYP		6.7	0.0
8	mPW1PW91		6.3	0.0
9	M06		5.1	0.0
10	M06-L		7.7	0.0
11	M06-2X		6.9	0.0
12	PBEPBE		6.1	0.0
13	PBE1PBE		6.2	0.0
14	HF		5.4	0.0
15	wB97X-D	def2SVP	18.8	0.0
16	wB97X-D	cc-pvDZ	7.5	0.0

(kcal/mol)

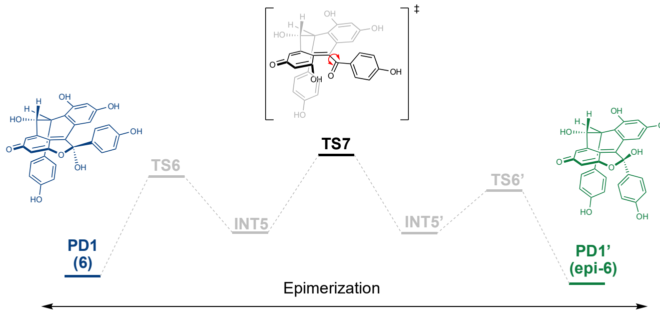

num	Opt/Freq		PD1	TS7	PD1'
	Functional	Basis set	ΔG	ΔG	ΔG
1	wB97X-D	6-31G**	2.0	35.8	0.0
2	wB97		1.4	34.9	0.0
3	B3LYP		-0.4	31.9	0.0
4	B98		0.3	33.5	0.0
5	BLYP		-0.4	27.6	0.0
6	B3PW91		-1.1	34.6	0.0
7	cam-B3LYP		0.7	36.0	0.0
8	mPW1PW91		-0.1	36.1	0.0
9	M06		0.8	33.3	0.0
10	M06-L		0.9	28.9	0.0
11	M06-2X		1.6	36.1	0.0
12	PBEPBE		-0.2	32.1	0.0
13	PBE1PBE		0.1	37.0	0.0
14	HF		0.5	36.8	0.0
15	wB97X-D	def2SVP	0.8	34.8	0.0
16	wB97X-D	cc-pvDZ	2.7	35.2	0.0

(kcal/mol)

Supplementary Fig. 17. Validation of TS1, TS1' and TS7 with various functionals/basis sets.

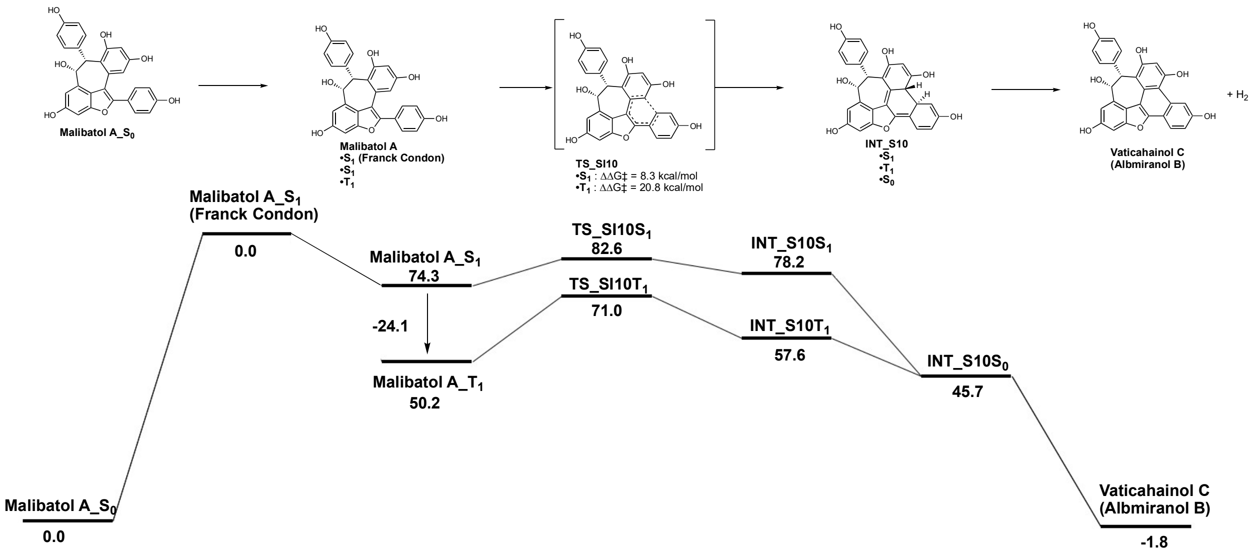

Supplementary Fig. 18. Photo 6π electrocyclization of malibatol A via excited singlet state (S_1) or triplet state (T_1) to form vaticahainol C (albmiranol B). S_1 was calculated by TD-DFT calculation and T_1 was calculated using unrestricted functional with (charge, spin) = (0, 3).

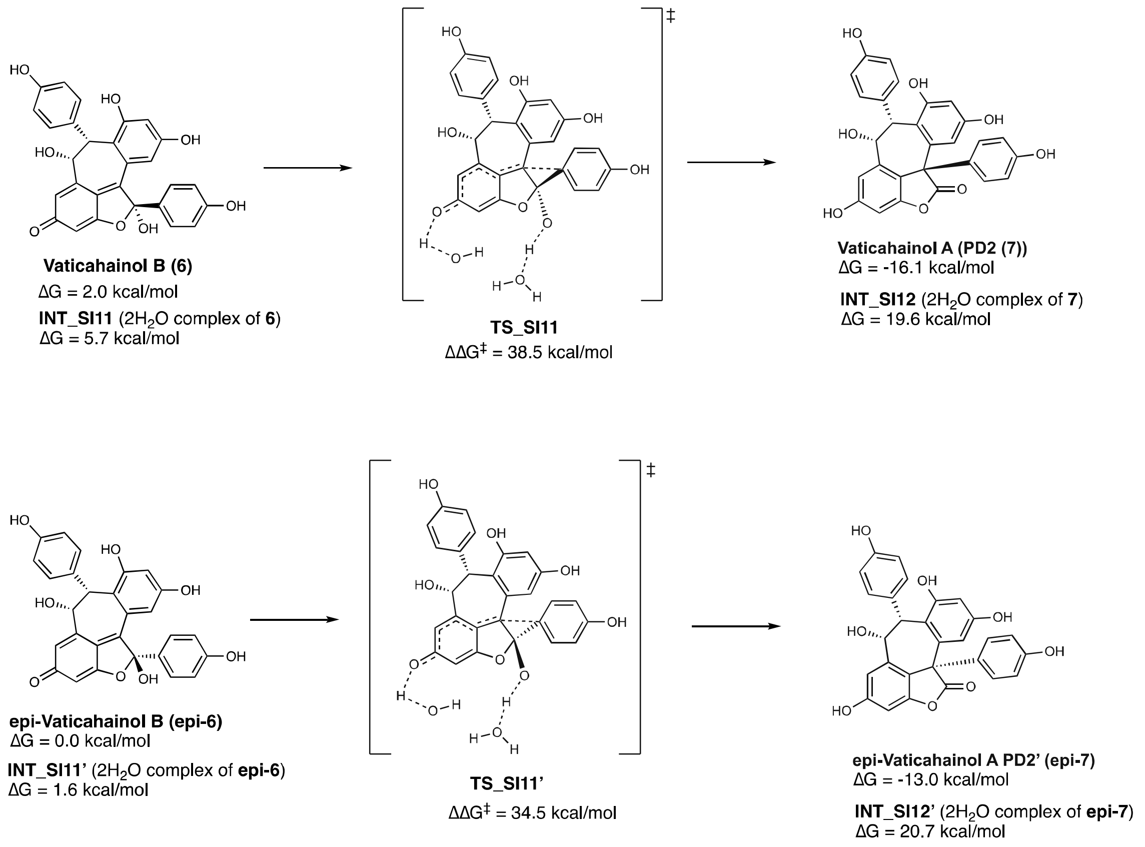

Supplementary Fig. 19. Ar rearrangement to form (epi-)vaticahainol B to A under neutral condition.

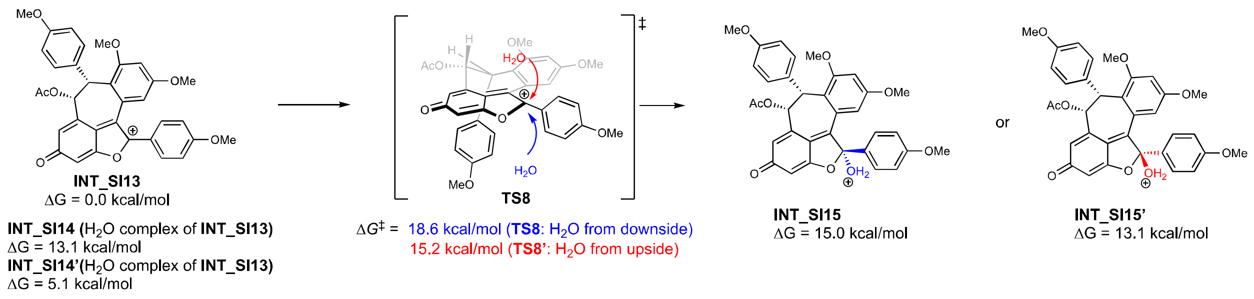

Supplementary Fig. 20. Details for the CAN oxidation shown in Figure 3a.

a

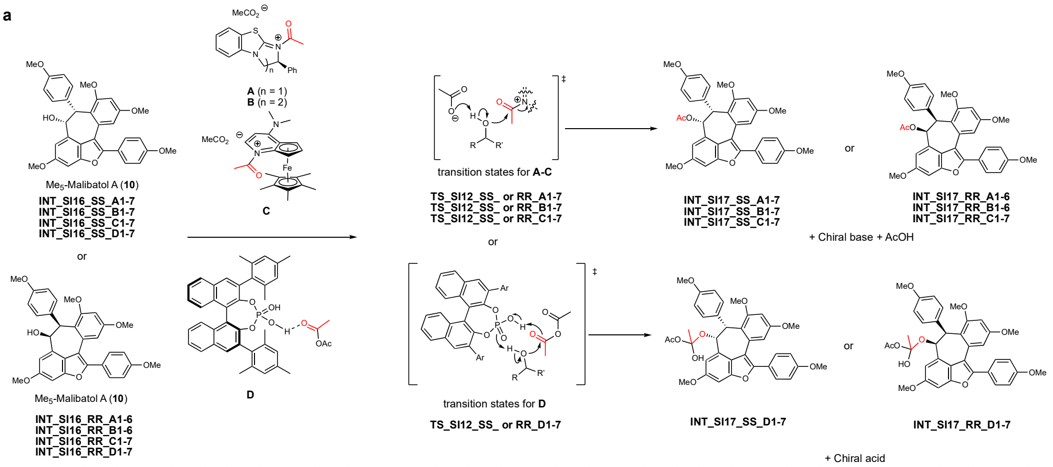

b

Chiral reagent	Absolute configuration	TS	G (kcal/mol)	ΔG‡
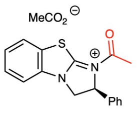 A	(S, S)	TS_SI12_SS_A1	-2051135.772	0.0
		TS_SI12_SS_A2	-2051134.408	1.4
		TS_SI12_SS_A3	-2051132.843	2.9
		TS_SI12_SS_A4	-2051129.307	6.5
		TS_SI12_SS_A5	-2051133.884	1.9
		TS_SI12_SS_A6	-2051129.507	6.3
		TS_SI12_SS_A7	-2051133.454	2.3
	(R, R)	TS_SI12_RR_A1	-2051130.373	5.4
		TS_SI12_RR_A2	-2051135.026	0.7
		TS_SI12_RR_A3	-2051131.965	3.8
		TS_SI12_RR_A4	-2051133.796	2.0
		TS_SI12_RR_A5	-2051127.966	7.8
		TS_SI12_RR_A6	-2051133.218	2.6

Chiral reagent	Absolute configuration	TS	G (kcal/mol)	ΔG‡
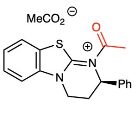 B	(S, S)	TS_SI12_SS_B1	-2075781.929	1.7
		TS_SI12_SS_B2	-2075783.61	0.0
		TS_SI12_SS_B3	-2075782.134	1.5
		TS_SI12_SS_B4	-2075782.891	0.7
		TS_SI12_SS_B5	-2075782.897	0.7
		TS_SI12_SS_B6	-2075778.295	5.3
		TS_SI12_SS_B7	-2075781.58	2.0
	(R, R)	TS_SI12_RR_B1	-2075780.414	3.2
		TS_SI12_RR_B2	-2075780.867	2.7
		TS_SI12_RR_B3	-2075781.14	2.5
		TS_SI12_RR_B4	-2075783.616	0.0
		TS_SI12_RR_B5	-2075774.488	9.1
		TS_SI12_RR_B6	-2075781.873	1.7

Chiral reagent	Absolute configuration	TS	G (kcal/mol)	ΔG‡
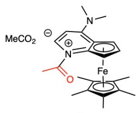 C	(S, S)	TS_SI12_SS_C1	-2003452.751	7.5
		TS_SI12_SS_C2	-2003457.556	2.7
		TS_SI12_SS_C3	-2003458.499	1.7
		TS_SI12_SS_C4	-2003444.374	15.9
		TS_SI12_SS_C5	-2003459.694	0.5
		TS_SI12_SS_C6	-2003459.331	0.9
		TS_SI12_SS_C7	-2003459.173	1.1
	(R, R)	TS_SI12_RR_C1	-2003452.737	7.5
		TS_SI12_RR_C2	-2003446.335	13.9
		TS_SI12_RR_C3	-2003454.309	5.9
		TS_SI12_RR_C4	-2003453.526	6.7
		TS_SI12_RR_C5	-2003460.243	0.0
		TS_SI12_RR_C6	-2003456.777	3.5
		TS_SI12_RR_C7	-2003455.859	4.4

Chiral reagent	Absolute configuration	TS	G (kcal/mol)	ΔG‡
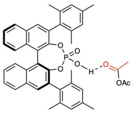 D	(S, S)	TS_SI12_SS_D1	-2693194.63	0.4
		TS_SI12_SS_D2	-2693191.193	3.8
		TS_SI12_SS_D3	-2693191.749	3.3
		TS_SI12_SS_D4	-2693188.714	6.3
		TS_SI12_SS_D5	-2693189.599	5.4
		TS_SI12_SS_D6	-2693186.991	8.0
		TS_SI12_SS_D7	-2693191.985	3.1
	(R, R)	TS_SI12_RR_D1	-2693189.912	5.1
		TS_SI12_RR_D2	-2693173.797	21.2
		TS_SI12_RR_D3	-2693187.196	7.8
		TS_SI12_RR_D4	-2693189.028	6.0
		TS_SI12_RR_D5	-2693190.857	4.2
		TS_SI12_RR_D6	-2693192.969	2.1
		TS_SI12_RR_D7	-2693195.041	0.0

Supplementary Fig. 21. Computational details for kinetic resolution. a) scheme and the name of intermediates and transition states. b) Table for the G of transition states of acylation with A-D. The data of $\Delta\Delta G_{TS}$ shown in Figure 4 were calculated using G in these table with considering Boltzmann distribution.

Chiral reagent	Absolute configuration	TS	ΔG‡								
			1	2	3	4	5	6	7	8	9
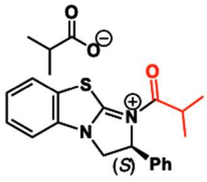	(S, S)	iPr_SS_A1	5.2	2.0	6.0	4.6	1.9	6.0	5.3	2.3	5.7
		iPr_SS_A2	0.5	5.0	7.7	1.0	5.8	8.1	1.2	7.5	9.6
		iPr_SS_A3	5.3	12.4	5.9	6.3	9.3	4.0	6.5	10.9	5.7
		iPr_SS_A4	6.3	9.3	4.1	8.8	6.4	5.7	10.9	5.3	2.8
		iPr_SS_A5	0.9	12.0	10.9	1.9	12.7	10.6	2.2	13.4	10.7
		iPr_SS_A6	12.4	15.5	8.3	11.5	15.5	0.9	13.4	1.9	8.8
		iPr_SS_A7	1.2	6.6	6.0	0.0	6.6	6.3	2.2	6.2	6.4
	(R, R)	iPr_RR_A1	8.4	12.8	6.2	9.9	11.3	5.8	8.7	7.2	5.8
		iPr_RR_A2	1.5	10.5	10.6	1.9	8.6	9.8	1.5	8.5	10.3
		iPr_RR_A3	4.3	8.8	6.6	4.2	7.9	6.8	3.2	8.9	7.1
		iPr_RR_A4	2.8	7.9	7.3	2.1	8.8	8.4	3.3	7.6	7.8
		iPr_RR_A5	8.9	9.4	16.7	9.0	16.8	7.1	16.9		
		iPr_RR_A6	4.1	4.3	6.6	3.8	7.1	4.3	5.9	7.2	3.8

Supplementary Fig. 22. Table for the $\Delta G^{\ddagger}$ of transition states of acylation with A (iPr). The data of $\Delta\Delta G_{TS}$ shown in Figure 5 were calculated using the data in these table with considering Boltzmann distribution.

3.Experimental procedure of synthesis for Malibatol A, Vaticahainols and Hopeahainol A

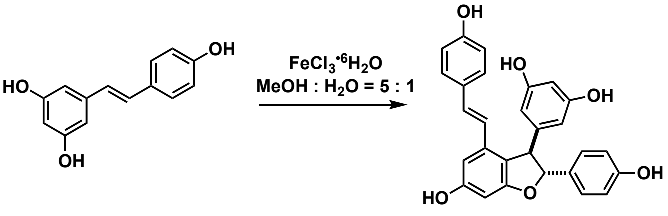

Resveratrol
1

ε-Viniferin
4

ε-Viniferin (2); To a solution of **Resveratrol (1)** (10.0 g, 43.8 mmol, 1 equiv.) in MeOH (110 mL), a solution of 17.8 g (64.8 mmol, 1.5 equiv.) of $FeCl_3 \cdot 6H_2O$ in H_2O (18 mL) was added at room temperature under argon atmosphere and the mixture was stirred. After 48 hours, H_2O was added and the reaction mixture was extracted with EtOAc. The combined organic layers were dried over Na_2SO_4, and the solvent was removed in *vacuo*. The residue was purified by silica gel column chromatography (SiO_2, hexane/acetone = 2/1 to 1/1) to give the desired product ε-**Viniferin(2)** (3.12 g, 6.78 mmol, 31% yield) as yellow amorphous solid. The spectral data of **2** were identical to those reported in the literature.[1]

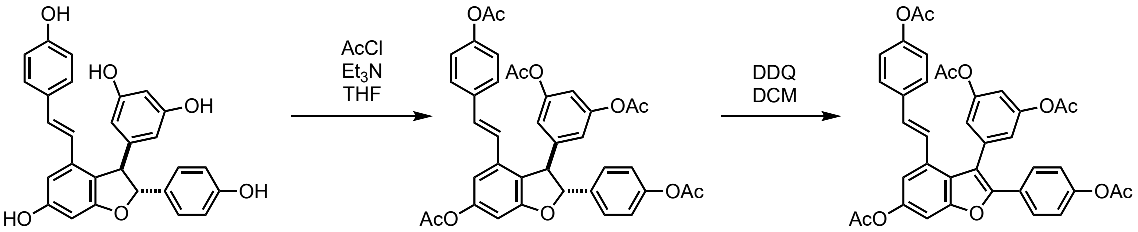

ε-Viniferin
2

Ac5-Viniferin
SI-1

Ac5-Viniferifuran
SI-2

(E)-5-(6-acetoxy-2-(4-acetoxyphenyl)-4-(4-acetoxystyryl)benzofuran-3-yl)-1,3-phenylene diacetate (SI-2); To a solution of **ε-Viniferin (2)** (3.12 g, 6.78 mmol, 1 equiv.) and Et_3N (11.3 ml, 81.8 mmol, 12 equiv.) in THF (135 mL), AcCl (2.9 ml, 40.7 mmol, 6 equiv.) was added dropwise for 5 minutes at 0 °C under argon atmosphere and the mixture was stirred at 0 °C. After 18 hours, H_2O was added and the reaction mixture was extracted with EtOAc. The combined organic layers were dried over Na_2SO_4, and the solvent was removed in *vacuo*. The residue was directly utilized for the next reaction without further purification. To a solution of crude **SI-1** in CH_2Cl_2 (68 mL), DDQ (23.1 g, 101.7 mmol) was added and the reaction mixture was refluxed with stirring for 48 hours. The reaction mixture was cooled to room temperature and filtrated with celite. The solvent was removed in *vacuo*. The residue was purified by silica gel column chromatography (SiO_2, hexane/EtOAc = 6/4) to give **SI-2** (3.09 g, 4.66 mmol, 69% yield in 2 steps) as yellow amorphous solid. The spectral data of **SI-2** were identical to those reported in the literature.[2]

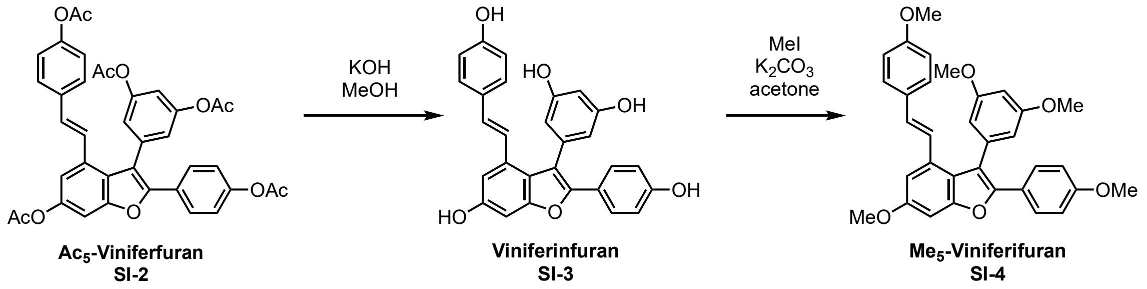

(E)-3-(3,5-dimethoxyphenyl)-6-methoxy-2-(4-methoxyphenyl)-4-(4-methoxystyryl)benzofuran (SI-4); To a solution of **SI-2** (3.09 g, 4.66 mmol, 1 equiv.) in MeOH (47 mL), KOH (2.61 g, 40.7 mmol, 10 equiv.) was added at 0 °C and mixture was stirred at 0 °C under argon atmosphere. After 1 hours, 1N HCl aqueous was added and the reaction mixture was extracted with EtOAc. The combined organic layers were dried over Na_2SO_4, and the solvent was removed in *vacuo*. The residue was directly utilized for the next reaction without further purification. To a solution of crude **ε-Viniferifuran (SI-3)** and K_2CO_3 (6.44 g, 46.6 mmol) in Acetone (93 mL), MeI (1.74 ml, 28.0 mmol) was added and the reaction mixture was refluxed with stirring for 18 hours. The reaction mixture was cooled to room temperature and filtrated with celite. The solvent was removed in *vacuo*. The residue was purified by silica gel column chromatography (SiO_2, hexane/EtOAc = 10/1 to 5/1) to give **SI-4** (1.41 g, 2.70 mmol, 58% yield in 2 steps) as yellow amorphous solid. The spectral data of **SI-4** were identical to those reported in the literature.[3]

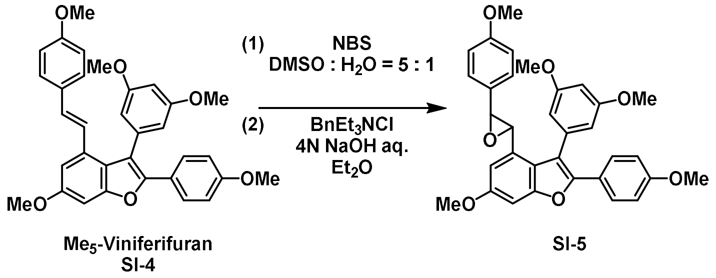

3-(3,5-Dimethoxyphenyl)-6-methoxy-2-(4-methoxyphenyl)-4-(3-(4-methoxyphenyl)oxiran-2-yl)benzofuran (SI-5); To a solution of **SI-4** (1.41 g, 2.70 mmol, 1 equiv.) in DMSO (27 mL) and H_2O (5.4 mL), NBS (504 mg, 2.84 mmol, 1.05 equiv.) was added one portion at 0 °C and the mixture was stirred at 0 °C under argon atmosphere. After 1 hours, 0.1 M aqueous $Na_2S_2O_3$ was added and the reaction mixture was extracted with Et_2O. The combined organic layers were dried over Na_2SO_4, and the solvent was removed in *vacuo*. The residue was directly utilized for the next reaction without further purification. To a solution of crude in Et_2O (27 mL) and 4N aqueous NaOH (9 mL), $BnEt_3NCl$ (613 mg, 2.70 mmol) was added and the reaction mixture was stirred at room temperature under argon atmosphere. After 3 hours, 0.1 M aqueous $Na_2S_2O_3$ was added and the reaction mixture was extracted with Et_2O. The solvent was removed in *vacuo*. The residue was purified by silica gel column chromatography (SiO_2, hexane/EtOAc = 10/1 to 5/1) to give **SI-5** (1.06 g, 1.97 mmol, 73% yield in 2

steps) as yellow amorphous solid. The spectral data of **SI-5** were identical to those reported in the literature.[3]

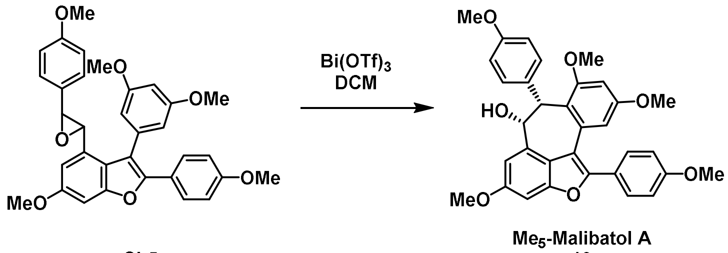

SI-5

Me5-Malibatol A
10

1,3,8-Trimethoxy-5,11-bis(4-methoxyphenyl)-10,11-dihydrobenzo[6,7]cyclohepta[1,2,3-cd]benzofuran-10-ol (10); To a solution of **SI-5** (215 mg, 0.400 mmol, 1 equiv.) in DCM (40 mL), $Bi(OTf)_3$ (26 mg, 0.040 mmol, 0.1 equiv.) was added at -78 °C and mixture was stirred at -78 °C under argon atmosphere. After 1 hours, saturated aqueous K_2CO_3 was added and the reaction mixture was extracted with DCM. The combined organic layers were dried over Na_2SO_4, and the solvent was removed in *vacuo*. The residue was purified by silica gel column chromatography (SiO_2, hexane/EtOAc = 5/1 to 2/1) to give **10** (114 mg, 0.212 mmol, 53% yield) as yellow amorphous solid; IR(ATR) ν 2937, 1608, 1509, 1295, 1249, 1197, 1143, 1039, 1030, 620 cm^{-1}; ^1H NMR ($CDCl_3$): δ 3.46 (s, 3H), 3.58 (s, 3H), 3.79 (s, 3H), 3.81 (s, 3H), 3.83 (s, 3H), 5.40 (s, 1H), 5.57 (d, J = 2.3 Hz, 1H), 6.41 (d, J = 2.3 Hz, 1H), 6.50 (d, J = 8.7 Hz, 2H), 6.71 (d, J = 2.3 Hz, 1H), 6.78 (d, J = 2.3 Hz, 1H), 6.93 (d, J = 8.7 Hz, 2H), 7.11 (d, J = 8.7 Hz, 2H), 7.12 (s, 1H), 7.61 (d, J = 8.7 Hz, 2H); ^{13}C NMR ($CDCl_3$): δ 47.61, 54.87(2C), 55.31, 55.59, 55.93, 73.54, 93.79, 98.01, 106.03, 108.18, 112.95, 113.93, 116.25, 117.92, 121.89, 124.30, 129.53, 130.16, 131.74, 134.08, 137.29, 150.83, 153.91, 157.40, 158.05, 158.22, 158.50, 159.95 ; (+)-ESI-HRMS. Calcd for $C_{33}H_{30}Na_1O_7$ ($M+Na^+$) 561.1889 Found: 561.1883.

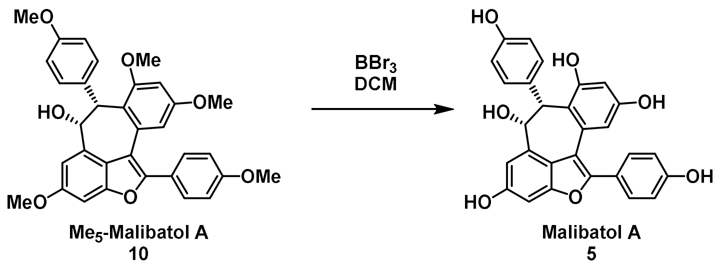

Me5-Malibatol A
10

Malibatol A
5

Malibatol A (5); To a solution of **10** (60.0 mg, 0.112 mmol, 1 equiv.) in DCM (11 mL), BBr_3 solution (1.0 M in DCM, 1.1 mL) was added at -78 °C under argon atmosphere and warm up to room temperature with stirring. After 3 hours, H_2O was added and the reaction mixture was extracted with AcOEt. The combined organic layers were dried over Na_2SO_4, and the solvent was removed in *vacuo*.

The residue was purified by silica gel column chromatography (SiO_2, DCM/MeOH = 9/1) to give **Malibatol A (5)** (15.7 mg, 0.034 mmol, 30% yield) as tan amorphous solid; IR(ATR) ν 3331, 1697, 1611, 1511, 1432, 1233, 1139, 1014, 836 cm^{-1}; ^1H NMR ($CDCl_3$): δ 5.28 (s, 1H), 5.47 (s, 1H), 6.31 (d, J = 2.8 Hz, 1H), 6.33 (d, J = 8.7 Hz, 2H), 6.51 (d, J = 2.3 Hz, 1H), 6.58 (d, J = 2.3 Hz, 1H), 6.81 (d, J = 8.7 Hz, 2H), 7.01 – 7.03 (m, 3H), 7.44 (d, J = 8.7 Hz, 2H) ; ^{13}C NMR ($CDCl_3$): δ 49.85, 74.83, 95.88, 102.15, 109.73, 109.89, 114.71, 116.37, 117.28, 119.05, 121.23, 124.67, 130.63, 130.89, 133.44, 135.83, 139.66, 151.23, 155.17, 155.37, 156.20, 156.72, 157.47, 159.10 ; (+)-ESI-HRMS. Calcd for $C_{28}H_{20}Na_1O_7$ ($M+Na^+$) 491.1107 Found: 491.1097.

Reported[4] Malibatol A (5); ^1H NMR (500 MHz, CD_3OD): δ 5.28 (ddd, J = 2.5, 1.0, 1.0 Hz, 1H), 5.46 (dd, J = 2.5, 1.0 Hz, 1H), 6.30 (d, J = 2.5 Hz, 1H), 6.33 (dd, J = 9.0, 2.5 Hz, 2H), 6.51 (d, J = 2.5 Hz, 1H), 6.57 (dd, J = 2.0, 1.0 Hz, 1H), 6.80 (dd, J = 8.5, 2.5 Hz, 2H), 7.01 (dd, J = 2.0, 1.0 Hz, 1H), 7.02 (dd, J = 9.0, 2.5 Hz, 2H), 7.45, (dd, J = 8.5, 2.5 Hz, 2H) ; ^{13}C NMR(125 MHz, CD_3OD) δ 48.8, 74.8, 95.9, 102.2, 109.7, 109.9, 114.7, 116.4, 117.3, 119.1, 121.3, 124.7, 130.6, 130.9, 133.4, 135.8, 139.7, 151.2, 155.2, 155.4, 156.2, 156.7, 157.5, 159.1.

	1H NMR				
H	Synthetic Malibatol A		Natural Malibatol A		Δx (ppm)
1	5.28	(s, 1H)	5.28	(ddd, J = 2.5, 1.0, 1.0 Hz, 1H)	0.00
2	5.47	(s, 1H)	5.46	(dd, J = 2.5, 1.0 Hz, 1H)	0.01
3	6.31	(d, J = 2.8 Hz, 1H)	6.30	(d, J = 2.5 Hz, 1H)	0.01
4	6.33	(d, J = 8.7 Hz, 2H)	6.33	(dd, J = 9.0, 2.5 Hz, 2H)	0.00
5	6.51	(d, J = 2.3 Hz, 1H)	6.51	(d, J = 2.5 Hz, 1H)	0.00
6	6.58	(d, J = 2.3 Hz, 1H)	6.57	(dd, J = 2.0, 1.0 Hz, 1H)	0.01
7	6.81	(d, J = 8.7 Hz, 2H)	6.80	(dd, J = 8.5, 2.5 Hz, 2H)	0.01
8	7.01 – 7.03	(m, 3H)	7.01	(dd, J = 2.0, 1.0 Hz, 1H)	0.01
9			7.02	(dd, J = 9.0, 2.5 Hz, 2H)	0.00
10	7.44	(d, J = 8.7 Hz, 2H)	7.45	(dd, J = 8.5, 2.5 Hz, 2H)	-0.01

	13C NMR		
C	Synthetic Malibatol A	Natural Malibatol A	Δx (ppm)
1	49.9	48.8	1.1
2	74.8	74.8	0.0
3	95.9	95.9	0.0
4	102.2	102.2	0.0
5	109.7	109.7	0.0
6	109.9	109.9	0.0
7	114.7	114.7	0.0
8	116.4	116.4	0.0
9	117.3	117.3	0.0
10	119.1	119.1	0.0
11	121.2	121.3	-0.1
12	124.7	124.7	0.0
13	130.6	130.6	0.0
14	130.9	130.9	0.0
15	133.4	133.4	0.0
16	135.8	135.8	0.0
17	139.7	139.7	0.0
18	151.2	151.2	0.0
19	155.2	155.2	0.0
20	155.4	155.4	0.0
21	156.2	156.2	0.0
22	156.7	156.7	0.0
23	157.5	157.5	0.0
24	159.1	159.1	0.0

Supplementary Fig. 23. Comparison of ^1H and ^{13}C NMR of synthetic and natural malibatol A.

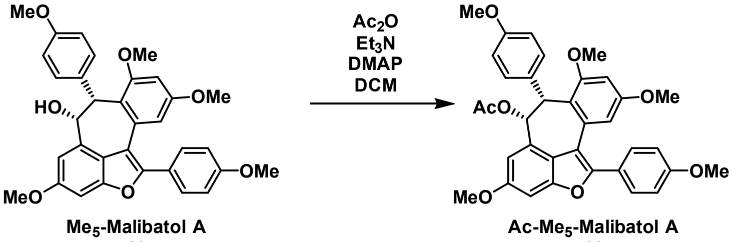

Me5-Malibatol A
10

Ac-Me5-Malibatol A
11

5-Hydroxy-1,3-dimethoxy-5,11-bis(4-methoxyphenyl)-8-oxo-5,8,10,11-

tetrahydrobenzo[6,7]cyclohepta[1,2,3-cd]benzofuran-10-yl acetate (11); To a solution of **10** (200 mg, 0.372 mmol, 1 equiv.), Et_3N (104 μL , 0.746 mmol, 2 equiv.) and DMAP (4.5 mg, 0.037 mmol, 0.1 equiv.) in DCM (2.8 mL), Ac_2O (70.5 μL, 0.740 mmol, 2 equiv.) was added at room temperature and the mixture was stirred under argon atmosphere. After 3 hours, H_2O was added and the reaction mixture was extracted with AcOEt. The combined organic layers were dried over Na_2SO_4, and the solvent was removed in *vacuo*. The residue was purified by silica gel column chromatography (SiO_2, hexane/EtOAc = 2/1 to 1/1) to give **11** (205 mg, 0.354 mmol, 95% yield); IR(ATR) ν 2935, 1739, 1609, 1509, 1460, 1374, 1249, 1198, 1177, 1145, 1069, 1028, 836 cm^{-1}; ^1H NMR ($CDCl_3$): δ 2.38 (s, 3H), 3.48 (s, 3H), 3.59(s, 3H), 3.81 (s, 3H), 3.84 (s, 6H), 5.57 (s, 1H), 6.45 (d, *J* = 2.7 Hz, 1H), 6.50 (d, *J* = 8.7 Hz, 2H), 6.52 (s, 1H), 6.69 (d, *J* = 2.7 Hz, 1H), 6.76 (d, *J* = 2.3 Hz, 1H), 6.78 (d, *J* = 2.3 Hz, 1H), 6.93 (d, *J* = 8.7 Hz, 2H), 7.05 (d, *J* = 8.7 Hz, 2H), 7.59 (d, *J* = 8.7 Hz, 2H);^{13}C NMR ($CDCl_3$): *δ* 21.42, 44.26, 54.86, 54.91, 55.30, 55.54, 56.18, 74.80, 93.33, 98.25, 106.21, 108.56, 112.79, 113.92, 115.83, 118.01, 121.01, 124.06, 129.08, 130.06, 131.61, 133.41, 134.13, 150.87, 153.93, 157.25, 157.71, 158.51, 158.71, 159.97, 170.02; (+)-ESI-HRMS. Calcd for $C_{33}H_{30}Na_1O_7$ ($M+Na^+$) 603.1995 Found: 603.1996.

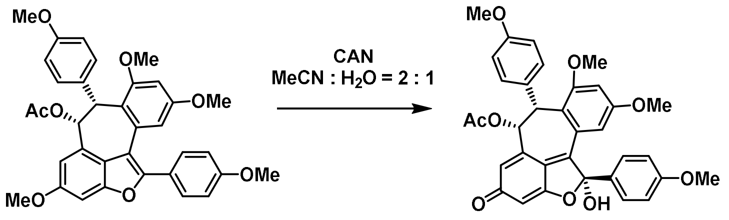

Ac-Me_5-Malibatol A
11

Ac-Me_4-Vaticahainol B
12

To a solution of **11** (205 mg, 0.353 mmol, 1 equiv.) in MeCN (24 mL), CAN (426 mg, 0.777 mmol, 2.2 equiv.) in H_2O (12 mL) was added dropwise for 3 minutes at room temperature and the mixture was stirred under argon atmosphere. After 24 hours, 0.1 M aqueous $Na_2S_2O_3$ was added and the reaction mixture was extracted with DCM, and washed brine. The solvent was removed in *vacuo*. The residue was purified by silica gel column chromatography (SiO_2, hexane/EtOAc = 1/1) to give **12** (162 mg, 0.279 mmol, 79% yield) as yellow amorphous solid; IR(ATR) ν 3366, 1794, 1737, 1712, 1629, 1604, 1508, 1457, 1364, 1300, 1250, 1201, 1178, 1083, 1032, 994, 832, 733 cm^{-1}; ^1H NMR ($CDCl_3$): δ 2,26 (s, 3H), 3.70 (s, 3H), 3.71 (s, 3H), 3.74 (s, 3H), 3.77 (s, 3H), 5.28 (s, 1H), 5.58 (s, 1H), 6.01 (s, 1H), 6.23 (s, 1H), 6.58 (d, *J* = 1.4 Hz, 1H), 6.62 (d, *J* = 8.7 Hz, 2H), 6.72 (d, *J* = 8.7 Hz, 2H), 6.76 (d, J = 8.7 Hz, 2H), 7.13 (d, *J* = 8.7 Hz, 2H), 7.59 (d, *J* = 1.4 Hz, 1H); ^{13}C NMR ($CDCl_3$): *δ* 21.14, 41.56, 55.08, 55.21, 55.32, 56.32, 73.42, 99.89, 103.25, 107.11, 113.25, 113.40, 114.83, 122.47, 123.05, 124.44, 127.41, 128.80, 128.91, 129.13, 130.76, 140.56, 153.08, 157.74, 158.16, 159.15, 160.07, 169.76, 170.40, 189.42; (+)-ESI-HRMS. Calcd for $C_{34}H_{30}Na_1O_9$ ($M+Na^+$) 605.1788 Found: 605.1771.

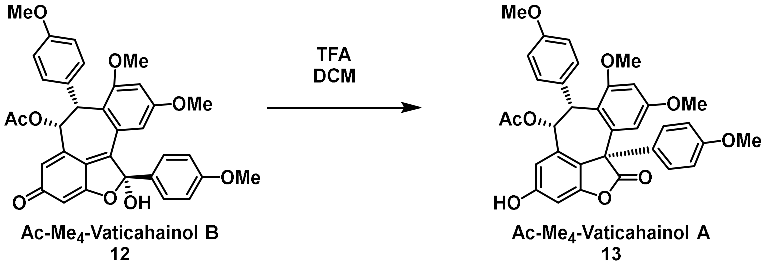

8-Hydroxy-1,3-dimethoxy-4b,11-bis(4-methoxyphenyl)-5-oxo-4b,5,10,11-tetrahydrobenzo[6,7]cyclohepta[1,2,3-cd]benzofuran-10-yl acetate (13); To a solution of **12** (58.0 mg, 0.0996 mmol, 1 equiv.) in DCM (10 mL), TFA (57 μL, 0.747 mmol, 7.5 equiv.) was added at room temperature and the mixture was warmed up to 70 °C with stirring. After 24 hours, 0.1 M aqueous $Na_2S_2O_3$ was added and the reaction mixture was extracted with DCM, and washed brine. The solvent was removed in *vacuo*. The residue was purified by silica gel column chromatography (SiO_2, hexane/EtOAc = 1/1) to give **13** (62 mg, 0.107 mmol, 79% yield) as yellow amorphous solid. IR(ATR) ν 1805, 1739, 1605, 1463, 1303, 1252, 1031, 872 cm^{-1}; ^{1}H NMR ($CDCl_3$): δ 1.12 (s, 3H), 3.41 (s, 3H), 3.71 (s, 3H), 3.73 (s, 3H), 3.87 (s, 3H), 4.95 (d, J = 5.0 Hz, 1H), 5.91 (d, J = 5.0 Hz, 1H), 6.44 (d, J = 2.3 Hz, 1H), 6.58 (d, J = 8.7 Hz, 2H), 6.65 (d, J = 2.3 Hz, 1H), 6.74 (d, J = 9.2 Hz, 2H), 6.76 (d, J = 2.3 Hz, 1H), 6.85 (d, J = 8.7 Hz, 2H), 6.91-7.09 (br, 2H), 7.69 (d, J = 2.3 Hz, 1H) ; ^{13}C NMR ($CDCl_3$): δ 19.87, 45.95, 54.91, 55.16, 55.32, 55.90, 58.67, 73.33, 99.84, 104.80, 111.01, 112.82, 114.15, 121.12, 123.86, 127.05, 127.74, 129.07, 133.59, 134.07, 134.75, 135.00, 137.15, 153.17, 157.17, 158.70, 158.87, 160.54, 169.57, 175.64 ; (+)-ESI-HRMS. Calcd for $C_{34}H_{30}Na_1O_9$ ($M+Na^+$) 605.1788 Found: 605.1775.

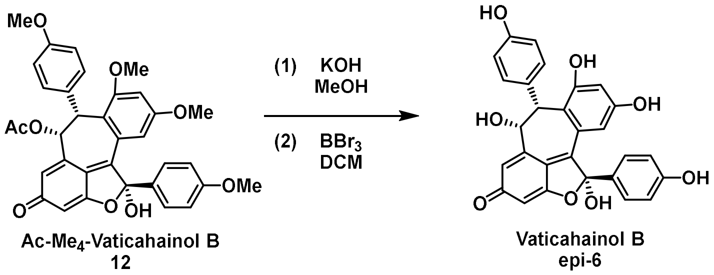

Vaticahainol B (epi-6) ; To a solution of **12** (22.3mg, 0.0383 mmol, 1 equiv.) in MeOH (3.8 mL), KOH (6.4 g, 0.114 mmol, 3 equiv.) was added at room temperature and mixture was stirred under argon atmosphere. After 3 hours, 1N HCl aqueous was added and the reaction mixture was extracted with EtOAc. The combined organic layers were dried over Na_2SO_4, and the solvent was removed in *vacuo*. The residue was directly utilized for the next reaction without further purification. To a solution of crude in DCM (11 mL), BBr_3 solution (1.0 M in DCM, 1.1 mL) was added at -78 °C under argon atmosphere and warm up to room temperature with stirring. After 16 hours, H_2O was

added and the reaction mixture was extracted with AcOEt. The combined organic layers were dried over Na_2SO_4, and the solvent was removed in *vacuo*. The residue was purified by silica gel column chromatography (SiO_2, DCM/MeOH = 9/1) to give **Vaticahainol B (epi-6)** (14.1 mg, 0.0291 mmol, 76% yield) as yellow amorphous solid; IR(ATR) ν 3197, 2965, 2926, 1687, 1645, 1599, 1531, 1511, 1440, 1362, 1315, 1207, 1173, 1108, 1090, 1024, 886, 831 cm^{-1}; ^1H NMR (dmso-d6): δ 4.82 (s, 1H), 5.12 (s, 1H), 5.40 (d, *J* = 1.4 Hz, 1H), 6.18 (d, *J* = 2.7 Hz, 1H), 6.27 (dd, *J* = 2.0, 2.0 Hz, 1H), 6.45 (d, *J* = 8.7 Hz, 2H), 6.46 (d, *J* = 2.3 Hz, 1H), 6.64 (d, *J* = 8.7 Hz, 2H), 6.66 (d, *J* = 8.7 Hz, 2H), 6.95 (d, *J* = 8.7 Hz, 2H), 7.25 (d, *J* = 2.3 Hz, 1H), 8.69 (s, 1H), 9.06 (s, 1H), 9.44 (s, 1H), 9.61 (s, 1H), 9.68 (s, 1H) ; ^{13}C NMR (dmso-d6): *δ* 44.14, 71.77, 97.72, 105.84, 109.55, 114.09, 114.47, 114.58, 119.93, 122.43, 124.42, 127.35, 128.67, 128.76, 129.06, 130.92, 144.90, 151.23, 155.35, 155.35, 155.92, 157.73, 169.30, 186.97; (+)-ESI-HRMS. Calcd for $C_{28}H_{20}Na_1O_8$ ($M+Na^+$) 507.1056 Found: 507.1061.

Reported[5] Vaticahainol B; ^1H NMR (dmso-d6): δ 4.81 (s, 1H), 5.10 (s, 1H), 5.39 (d, *J* = 1.5 Hz, 1H), 6.15 (d, *J* = 1.5 Hz, 1H), 6.26 (s, 1H), 6.43 (d, *J* = 8.5 Hz, 2H), 6.44 (d, *J* = 2.0 Hz, 1H), 6.63 (d, *J* = 8.5 Hz, 2H), 6.65 (d, *J* = 8.5 Hz, 2H), 6.95 (d, *J* = 8.5 Hz, 2H), 7.23 (d, *J* = 2.0 Hz, 1H), 8.67 (s, 1H), 9.04 (br, 1H), 9.43 (br, 1H), 9.59 (br, 1H), 9.67 (s, 1H); ^{13}C NMR (dmso-d6): *δ* 45.9, 73.5, 99.4, 107.6, 111.3, 115.8, 116.1, 116.3, 121.7, 124.1, 126.1, 129.0, 130.4, 130.5, 130.7, 132.6, 146.6, 152.9, 157.0, 157.0, 157.6, 159.4, 171.0, 188.6.

	1H NMR				
H	Synthetic epi-Vaticahainol B		Natural Vaticahainol B		Δx (ppm)
1	4.82	(s, 1H)	4.81	(s, 1H)	0.01
2	5.12	(s, 1H)	5.10	(s, 1H)	0.02
3	5.4	(d, J = 1.4 Hz, 1H)	5.39	(d, J = 1.5 Hz, 1H)	0.01
4	6.18	(d, J = 2.7 Hz, 1H)	6.15	(d, J = 1.5 Hz, 1H)	0.03
5	6.27	(dd, J = 2.0, 2.0 Hz, 1H)	6.26	(s, 1H)	0.01
6	6.45	(d, J = 8.7 Hz, 2H)	6.43	(d, J = 8.5 Hz, 2H)	0.02
7	6.46	(d, J = 2.3 Hz, 1H)	6.44	(d, J = 2.0 Hz, 1H)	0.02
8	6.64	(d, J = 8.7 Hz, 2H)	6.63	(d, J = 8.5 Hz, 2H)	0.01
9	6.66	(d, J = 8.7 Hz, 2H)	6.65	(d, J = 8.5 Hz, 2H)	0.01
10	6.95	(d, J = 8.7 Hz, 2H)	6.95	(d, J = 8.5 Hz, 2H)	0.00
11	7.25	(d, J = 2.3 Hz, 1H)	7.23	(d, J = 2.0 Hz, 1H)	0.02
12	8.69	(s, 1H)	8.67	(s, 1H)	0.02
13	9.06	(s, 1H)	9.04	(br, 1H)	0.02
14	9.44	(s, 1H)	9.43	(br, 1H)	0.01
15	9.61	(s, 1H)	9.59	(br, 1H)	0.02
16	9.68	(s, 1H)	9.67	(s, 1H)	0.01

	13C NMR		
C	Synthetic epi-Vaticahainol B	Natural Vaticahainol B	Δx (ppm)
1	44.1	45.9	-1.8
2	71.8	73.5	-1.7
3	97.7	99.4	-1.7
4	105.8	107.6	-1.8
5	109.6	111.3	-1.8
6	114.1	115.8	-1.7
7	114.5	116.1	-1.6
8	114.6	116.3	-1.7
9	119.9	121.7	-1.8
10	122.4	124.1	-1.7
11	124.4	126.1	-1.7
12	127.4	129.0	-1.7
13	128.7	130.4	-1.7
14	128.8	130.5	-1.7
15	129.1	130.7	-1.6
16	130.9	132.6	-1.7
17	144.9	146.6	-1.7
18	151.2	152.9	-1.7
19	155.4	157.0	-1.7
20	155.4	157.0	-1.7
21	155.9	157.6	-1.7
22	157.7	159.4	-1.7
23	169.3	171.0	-1.7
24	187.0	188.6	-1.6

Supplementary Fig. 24. Comparison of ^1H and ^{13}C NMR of synthetic and natural Vaticahainol B.

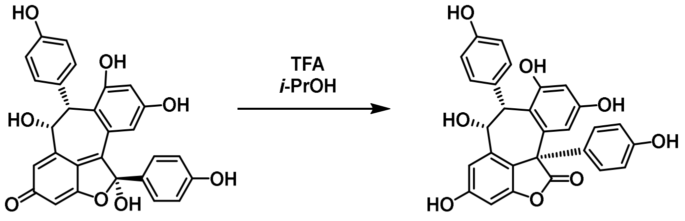

Vaticahainol B
epi-6

Vaticahainol A
epi-7

Vaticahainol A (epi-7) : To a solution of **Vaticahainol B (epi-6)** (8.0 mg, 0.0165 mmol, 1 equiv.) in *i*-PrOH (1.6 mL), TFA (9.5 μL, 0.123 mmol, 7.5 equiv.) was added at room temperature and the mixture was warmed up to 70 °C with stirring. After 15 hours, H_2O was added and the reaction mixture was extracted with AcOEt, and washed brine. The solvent was removed in *vacuo*. The residue was purified by silica gel column chromatography (SiO_2, DCM/MeOH = 1/1) to give **Vaticahainol A (epi-7)** (5.0 mg, 0.0102 mmol, 62% yield) as yellow amorphous solid; IR(ATR) ν 3258, 1775, 1595, 1510, 1443, 1349, 1322, 1246, 1178, 1146, 1117, 1060, 1035, 1019, 997, 826, 796 cm^{-1}; ^1H NMR (dmso-d6): δ 4.66 (dd, *J* = 4.8, 4.8 Hz, 1H), 5.21 (d, *J* = 4.8 Hz, 1H), 5.50 (d, *J* = 4.8 Hz, 1H), 6.05 (d, *J* = 9.0 Hz, 2H), 6.14 (br, 4H), 6.42 (d, *J* = 2.0 Hz, 1H), 6.51 (d, *J* = 8.3 Hz, 2H), 6.55 (d, *J* = 2.1 Hz, 1H), 7.06 (d, *J* = 1.4 Hz, 1H), 7.24 (d, *J* =2.1 Hz, 1H), 8.58 (br, 1H), 9.09 (br, 1H), 9.39 (br, 1H), 9.61 (br, 1H), 10.03 (br, 1H) ; ^{13}C NMR ($CDCl_3$): *δ* 44.05, 57.89, 71.10, 96.04, 101.94, 105.84, 109.76, 113.53, 114.37, 114.62, 118.21, 127.14, 129.10, 130.32, 131.14, 137.78, 143.07, 152.68, 153.94, 155.82, 155.95, 157.46, 158.46, 175.74, ; (+)-ESI-HRMS. Calcd for $C_{28}H_{20}Na_1O_8$ ($M+Na^+$) 507.1056 Found: 507.1057.

Reported[5] Vaticahainol A ; ^1H NMR (dmso-d6): δ 4.67 (t, *J* = 4.0 Hz, 1H), 5.22 (d, *J* = 4.0 Hz, 1H), 5.55 (d, *J* = 4.0 Hz, 1H), 6.05 (d, *J* = 8.2 Hz, 2H), 6.15 (br, 4H), 6.44 (d, *J* = 2.0 Hz, 1H), 6.52 (d, *J* = 8.2 Hz, 2H), 6.56 (d, *J* = 1.5 Hz, 1H) 7.08 (d, *J* = 1.5 Hz, 1H), 7.25 (d, *J* = 2.0 Hz, 1H), 8.60 (br, 1H), 9.11 (br, 1H), 9.42 (br, 1H), 9.64 (br, 1H), 10.05 (br, 1H); ^{13}C NMR ($CDCl_3$): *δ* 44.0, 57.9, 71.1, 96.0, 101.9, 105.8, 109.7, 113.5, 114.4, 114.6, 118.2, 127.1, 129.1, 130.3, 131.1, 137.8, 143.1, 152.7, 153.9, 155.8, 155.9, 157.4, 158.4, 175.7.

	1H NMR				
H	Synthetic epi-Vaticahainol A		Natural Vaticahainol A		Δx (ppm)
1	4.66	(dd, J = 4.8, 4.8 Hz, 1H)	4.67	(t, J = 4.0 Hz, 1H)	-0.01
2	5.21	(d, J = 4.8 Hz, 1H)	5.22	(d, J = 4.0 Hz, 1H)	-0.01
3	5.50	(d, J = 4.8 Hz, 1H)	5.55	(d, J = 4.0 Hz, 1H)	-0.05
4	6.05	(d, J = 9.0 Hz, 2H)	6.05	(d, J = 8.2 Hz, 2H)	0.00
5	6.14	(br, 4H)	6.15	(br, 4H)	-0.01
6	6.42	(d, J = 2.0 Hz, 1H)	6.44	(d, J = 2.0 Hz, 1H)	-0.02
7	6.51	(d, J = 8.3 Hz, 2H)	6.52	(d, J = 8.2 Hz, 2H)	-0.01
8	6.55	(d, J = 2.1 Hz, 1H)	6.56	(d, J = 1.5 Hz, 1H)	-0.01
9	7.06	(d, J = 1.4 Hz, 1H)	7.08	(d, J = 1.5 Hz, 1H)	-0.02
10	7.24	(d, J =2.1 Hz, 1H)	7.25	(d, J = 2.0 Hz, 1H)	-0.01
11	8.58	(br, 1H)	8.60	(br, 1H)	-0.02
12	9.09	(br, 1H)	9.11	(br, 1H)	-0.02
13	9.39	(br, 1H)	9.42	(br, 1H)	-0.03
14	9.61	(br, 1H)	9.64	(br, 1H)	-0.03
15	10.03	(br, 1H)	10.05	(br, 1H)	-0.02

	13C NMR		
C	Synthetic epi-Vaticahainol A	Natural Vaticahainol A	Δx (ppm)
1	44.1	44.0	0.0
2	57.9	57.9	0.0
3	71.1	71.1	0.0
4	96.0	96.0	0.0
5	101.9	101.9	0.0
6	105.8	105.8	0.0
7	109.8	109.7	0.1
8	113.5	113.5	0.0
9	114.4	114.4	0.0
10	114.6	114.6	0.0
11	118.2	118.2	0.0
12	127.1	127.1	0.0
13	129.1	129.1	0.0
14	130.3	130.3	0.0
15	131.1	131.1	0.0
16	137.8	137.8	0.0
17	143.1	143.1	0.0
18	152.7	152.7	0.0
19	153.9	153.9	0.0
20	155.8	155.8	0.0
21	156.0	155.9	0.0
22	157.5	157.4	0.1
23	158.5	158.4	0.1
24	175.7	175.7	0.0

Supplementary Fig. 25. Comparison of ^{1}H and ^{13}C NMR of synthetic and natural Vaticahainol A.

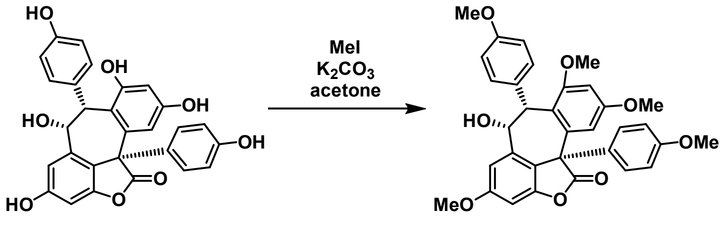

Vaticahainol A
epi-7

Me_5-Vaticahainol A
14

10-Hydroxy-1,3,8-trimethoxy-4b,11-bis(4-methoxyphenyl)-10,11-dihydrobenzo[6,7]cyclohepta[1,2,3-cd]benzofuran-5(4bH)-one (14); To solution of **Vaticahainol A (epi-7)** (4.8 mg, 0.010mmol, 1 equiv.) and K_2CO_3 (13.8 mg, 0.10 mmol, 10 equiv.) in acetone (1 mL), MeI (6.0 µL, 0.010 mmol, 1 equiv.) was added and the reaction mixture was refluxed with stirring for 18 hours. The reaction mixture was cooled to room temperature and filtrated with celite. The solvent was removed in *vacuo*. The residue was purified by silica gel column chromatography (SiO_2, hexane/EtOAc = 5/1 to 3/1) to give **14** (5.3 mg, 0.0096 mmol, 96% yield) as colorless amorphous solid; IR(ATR) ν 1791, 1628, 1604, 1581, 1509, 1461, 1319, 1303, 1199, 1182, 1146, 1036, 996, 824, 735 cm^{-1}; ^{1}H NMR (acetone-d6): δ 3.47 (s, 3H), 3.50 (s, 3H), 3.73 (s, 3H), 3.77 (s, 3H), 3.81 (s, 3H), 4.84 (d, J = 4.1 Hz, 1H), 5.42 (d, J = 4.1 Hz, 1H), 6.08 (d, J = 8.2 Hz, 2H), 6.17 (br, 4H), 6.60 (d, J = 8.2 Hz, 2H), 6.64 (d , J = 2.3 Hz, 1H), 6.66 (d, J = 2.3 Hz, 1H), 7.25 (d, J = 2.3 Hz, 1H), 7.63 (d, J = 2.3 Hz, 1H) ; ^{13}C NMR ($CDCl_3$): δ 47.28, 55.05, 55.13, 55.49, 55.87, 55.97, 58.74, 73.73, 96.40, 99.45, 104.49, 109.15, 113.54, 114.01, 119.42, 121.67, 128.15, 129.10, 133.47, 133.76, 136.69, 140.29, 153.82, 157.29, 158.72, 158.85, 160.33, 161.03, 176.73; (+)-ESI-HRMS. Calcd for $C_{33}H_3Na_1O_8$ ($M+Na^+$) 577.1838 Found: 577.1842.

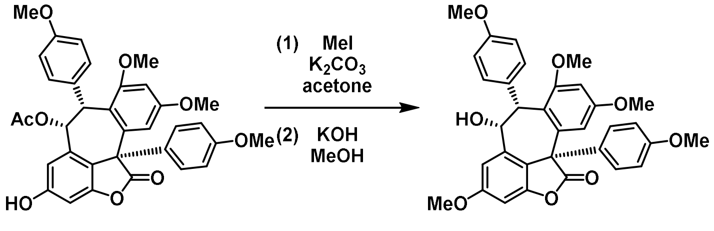

Ac-Me$_4$-Vaticahainol A
13

Me$_5$-Vaticahainol A
14

10-Hydroxy-1,3,8-trimethoxy-4b,11-bis(4-methoxyphenyl)-10,11-dihydrobenzo[6,7]cyclohepta[1,2,3-cd]benzofuran-5(4bH)-one (14); To a solution of **13** (20 mg, 0.0343 mmol, 1 equiv.) and K_2CO_3 (14.2 mg, 0.103 mmol, 3 equiv.) in acetone (3.8 mL), MeI (6.5 μL, 0.103 mmol, 3 equiv.) was added at room temperature and the mixture was warmed up to 60 °C with stirring under argon atmosphere. After 3 hours, the reaction mixture was cooled to room temperature and filtrated with celite. The solvent was removed in *vacuo*. The residue was directly utilized for the next reaction without further purification. To a solution of crude in MeOH (3 mL), KOH (5.6 mg, 0.10 mmol) was added at room temperature and the mixture was stirred under argon atmosphere. After 2 hours, 1N HCl aqueous was added and the reaction mixture was extracted with EtOAc. The combined organic layers were dried over Na_2SO_4, and the solvent was removed in *vacuo*. The residue was purified by silica gel column chromatography (SiO_2, hexane/EtOAc = 3/1 to 2/1) to give **14** (13.5 mg, 0.0244 mmol, 71% yield) as yellow amorphous solid.

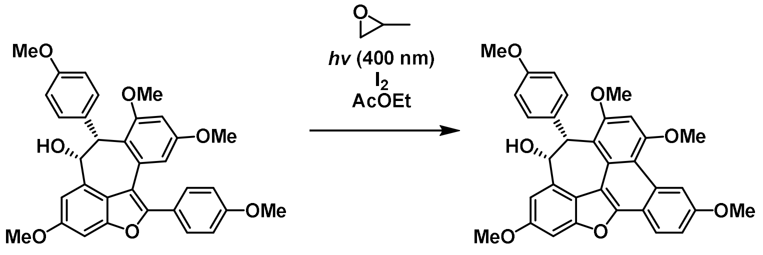

Me$_5$-Malibatol A
10

Me$_5$-Vaticahanol C
16

2,6,8,10-Tetramethoxy-5-(4-methoxyphenyl)-4,5-dihydro-13-oxabenzo[3,4]azuleno[7,8,1-jkl]phenanthren-4-ol (16); To a solution of **10** (68 mg, 0.126 mmol, 1 equiv.) and methyl oxirane (89.0 μL, 1.26 mmol, 10 equiv.) in AcOEt (12 mL), I_2 (34 mg, 1.33 mmol, 1.05 equiv.) was added at room temperature to 400 nm visible light and the mixture was stirred at room temperature. After 1 hours, 0.1 M aqueous $Na_2S_2O_3$ was added and the reaction mixture was extracted with AcOEt. The combined organic layers were dried over Na_2SO_4, and the solvent was removed in *vacuo*. The residue was purified by silica gel column chromatography (SiO_2, hexane/EtOAc = 3/1 to 1/1) to give **16** (48 mg, 0.0895 mmol, 71% yield) as yellow amorphous solid; IR(ATR) ν 1709, 1580, 1491, 1453, 1436, 1350, 1278, 1212, 1180, 1129, 1111, 1071, 1030, 1007, 863, 808 cm^{-1}; ^1H NMR ($CDCl_3$): δ 2.45

(br, 1H), 3.56 (s, 3H), 3.87 (s, 3H), 3.97 (s, 3H), 4.02 (s, 3H), 4.14 (s, 3H), 5.50 (d, *J* = 5.5 Hz, 1H), 5.87 (d, *J* = 3.2 Hz, 1H), 6.45 (d, *J* = 8.7 Hz, 2H), 6.81 (s, 1H), 6.90 (d, *J* = 8.7 Hz, 2H), 7.05 (s, 1H), 7.07 (s, 1H), 7.31 (dd, *J* = 8.7, 2.3 Hz, 1H), 8.36 (d, *J* = 8.7 Hz, 1H), 9.24 (d, *J* = 2.3 Hz, 1H); ^{13}C NMR ($CDCl_3$): δ 48.34, 54.90, 55.35, 55.79, 55.98, 56.36, 73.50, 94.16, 94.94, 107.44, 111.48, 112.86, 113.30, 113.78, 114.86, 115.56, 115.88, 117.41, 121.66, 130.35, 130.65, 131.83, 132.13, 137.10, 151.47, 155.51, 156.16, 157.93, 158.23, 158.95, 159.45; (+)-ESI-HRMS. Calcd for $C_{33}28Na_1O_7$ (M+Na^+) 559.1733 Found: 559.1739.

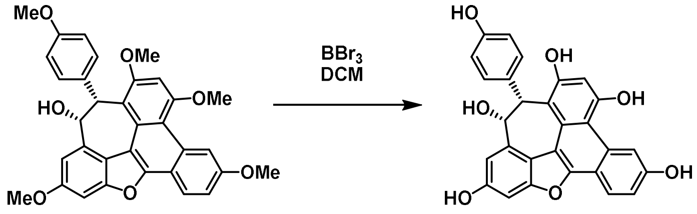

Vaticahainol C (17); To a solution of **16** (30.0 mg, 0.0559 mmol, 1 equiv.) in DCM (5.6 mL), BBr_3 solution (1.0 M in DCM, 0.56 mL) was added at -78 °C under argon atmosphere and the mixture was warmed up to room temperature with stirring. After 2 hours, H_2O was added and the reaction mixture was extracted with AcOEt. The combined organic layers were dried over Na_2SO_4, and the solvent was removed in *vacuo*. The residue was purified by silica gel column chromatography (SiO_2, DCM/MeOH = 9/1) to give **Vaticahainol C (17)** (6.8 mg, 0.0145 mmol, 26% yield) as tan amorphous solid; IR(ATR) ν 3310, 2923, 2852, 1686, 1627, 1610, 1581, 1532, 1510, 1451, 1404, 1362, 1350, 1259, 1212, 1179, 1126, 1053, 1025, 983, 871, 818 cm^{-1}; ^{1}H NMR (acetone-d6): δ 5.03 (br, 1H) 5.50 (s, 1H), 5.82 (d, J = 2.3 Hz, 1H), 6.25 (d, J = 8.7 Hz, 2H), 6.82 (d, J = 8.7 Hz, 2H), 6.86 (d, J = 2.3 Hz, 1H), 6.93 (s, 1H), 7.10 (d, J = 2.3 Hz, 1H), 7.15 (dd, J = 8.7, 2.3 Hz, 1H), 8.14 (d, J = 8.7 Hz, 1H), 9.46 (s, 1H) ; ^{13}C NMR (acetone-d6): δ 48.88, 74.33, 96.35, 102.23, 109.46, 111.90, 114.46, 114.59, 114.77, 115.51, 115.70, 115.74, 115.92, 121.90, 130.92, 132.84, 133.47, 133.73, 139.61, 151.52, 155.05, 155.77, 156.25, 156.74, 157.12, 157.58 ; (+)-ESI-HRMS. Calcd for $C_{28}H_{18}Na_1O_7$ ($M+Na^+$) 489.0950 Found: 489.0945.

Reported[6] Albiraminol B ; ^{1}H NMR (acetone-d6): δ 5.08 (br, 1H), 5.56 (br, 1H), 5.87 (br, 1H), 6.31 (d, J = 8.8 Hz, 2H), 6.87 (d, J = 8.8 Hz, 2H), 6.92 (s, 1H), 6.95 (s, 1H), 7.15 (s, 1H), 7.23 (dd, J = 8.8, 2.4 Hz, 1H), 8.21 (d, J = 8.8 Hz, 1H), 9.41 (d, J = 2.4 Hz, 1H); ^{13}C NMR (acetone-d6): 49.0, 74.4, 96.4, 102.3, 109.5, 112.0, 114.5, 114.7, 114.8, 115.1, 115.6, 115.9, 116.1, 121.9, 130.9, 133.4, 133.7, 133.7, 139.6, 151.6, 155.0, 155.8, 156.3, 156.8, 157.2, 157.5.

Reported[5] Vaticahainol C ; ^{1}H NMR (acetone-d6): δ 5.51 (d, J = 1.8 Hz, 1H), 5.82 (d, J = 1.8 Hz, 1H), 6.29 (d, J = 8.8 Hz, 2H), 6.83 (d, J = 8.8 Hz, 2H), 6.90 (dd, J = 2.0, 1.2 Hz, 1H), 6.96 (s, 1H), 7.11 (dd, J = 2.0, 1.2 Hz, 1H), 7.21 (dd, J = 8.8, 2.0 Hz, 1H), 8.17 (d, J = 8.8 Hz, 1H), 9.39 (d, J = 2.0 Hz, 1H); ^{13}C NMR (acetone-d6): 48.4, 73.9, 95.9, 101.7, 109.0, 111.3, 114.1, 114.2, 114.3, 114.9, 115.1, 115.2, 115.3, 121.4, 130.5, 131.8, 133.0, 133.3, 139.1, 151.0, 154.7, 155.4, 155.8, 156.4, 156.8, 157.3.

	1H NMR							
H	Synthetic Vaticahainol C (Albmiranol B)		Natural Vaticahainol C		Δx (ppm)	Natural Albiraminol B		Δx (ppm)
1	5.03	(br, 1H)	none	none		5.08	(br, 1H)	-0.05
2	5.5	(s, 1H)	5.51	(d, J = 1.8 Hz, 1H)	-0.01	5.56	(br, 1H)	-0.06
3	5.82	(d, J = 2.3 Hz, 1H)	5.82	(d, J = 1.8 Hz, 1H)	0.00	5.87	(br, 1H)	-0.05
4	6.25	(d, J = 8.7 Hz, 2H)	6.29	(d, J = 8.8 Hz, 2H)	-0.04	6.31	(d, J = 8.8 Hz, 2H)	-0.06
5	6.82	(d, J = 8.7 Hz, 2H)	6.83	(d, J = 8.8 Hz, 2H)	-0.01	6.87	(d, J = 8.8 Hz, 2H)	-0.05
6	6.86	(d, J = 2.3 Hz, 1H)	6.90	(dd, J = 2.0, 1.2 Hz, 1H)	-0.04	6.92	(s, 1H)	-0.06
7	6.93	(s, 1H)	6.96	(s, 1H)	-0.03	6.95	(s, 1H)	-0.02
8	7.1	(d, J = 2.3 Hz, 1H)	7.11	(dd, J = 2.0, 1.2 Hz, 1H)	-0.01	7.15	(s, 1H)	-0.05
9	7.15	(dd, J = 8.7, 2.3 Hz, 1H)	7.21	(dd, J = 8.8, 2.0 Hz, 1H)	-0.06	7.23	(dd, J = 8.8, 2.4 Hz, 1H)	-0.08
10	8.14	(d, J = 8.7 Hz, 1H)	8.17	(d, J = 8.8 Hz, 1H)	-0.03	8.21	(d, J = 8.8 Hz, 1H)	-0.07
11	9.46	(s, 1H)	9.39	(d, J = 2.0 Hz, 1H)	0.07	9.41	(d, J = 2.4 Hz, 1H)	0.05

	13C NMR				
C	Synthetic Vaticahainol C (Albmiranol B)	Natural Albiraminol B	Δx (ppm)	Natural Vaticahainol C	Δx (ppm)
1	48.9	48.4	0.5	49.0	0.5
2	74.3	73.9	0.4	74.4	0.4
3	96.4	95.9	0.4	96.4	0.4
4	102.2	101.7	0.5	102.3	0.5
5	109.5	109.0	0.5	109.5	0.5
6	111.9	111.3	0.6	112.0	0.6
7	114.5	114.1	0.4	114.5	0.4
8	114.6	114.2	0.4	114.7	0.4
9	114.8	114.3	0.5	114.8	0.5
10	115.5	114.9	0.6	115.1	0.6
11	115.7	115.1	0.6	115.6	0.6
12	115.7	115.2	0.5	115.9	0.5
13	115.9	115.3	0.6	116.1	0.6
14	121.9	121.4	0.5	121.9	0.5
15	130.9	130.5	0.4	130.9	0.4
16	132.8	131.8	1.0	133.4	1.0
17	133.5	133.0	0.5	133.7	0.5
18	133.7	133.3	0.4	133.7	0.4
19	139.6	139.1	0.5	139.6	0.5
20	151.5	151.0	0.5	151.6	0.5
21	155.1	154.7	0.4	155.0	0.4
22	155.8	155.4	0.4	155.8	0.4
23	156.3	155.8	0.4	156.3	0.4
24	156.7	156.4	0.3	156.8	0.3
25	157.1	156.8	0.3	157.2	0.3
26	157.6	157.3	0.3	157.5	0.3

Supplementary Fig. 26. Comparison of ^{1}H and ^{13}C NMR of synthetic and natural Vaticahainol C and Albiraminol B.

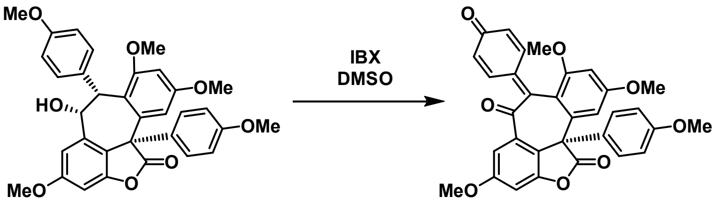

Me$_5$-Vaticahainol A
14

Me$_4$-Hopeahainol A
15

1,3,8-Trimethoxy-4b-(4-methoxyphenyl)-11-(4-oxocyclohexa-2,5-dien-1-ylidene)-4b,11-dihydrobenzo[6,7]cyclohepta[1,2,3-cd]benzofuran-5,10-dione (15); To a solution of **14** (10.0 mg,

0.0180 mmol, 1 equiv.) in DMSO (1 mL), IBX (50 mg, 0.178 mmol, 10 equiv.) was added at room temperature and the mixture was stirred under argon atmosphere. After 24 hours, 0.1 M aqueous $Na_2S_2O_3$ was added and the reaction mixture was extracted with Et_2O, and washed brine. The solvent was removed in *vacuo*. The residue was purified by silica gel column chromatography (SiO_2, hexane/EtOAc = 1/1) to give **15** (3.5 mg, 0.0065 mmol, 36% yield) as dark orange amorphous solid; IR(ATR) ν 1803, 1701, 1634, 1600, 1575, 1507, 1463, 1437, 1353, 1320, 1250, 1201, 1178, 1090, 1058, 1033, 989, 953, 862, 833 cm^{-1}; ^{1}H NMR (acetone-d6): δ 3.55 (s, 3H), 3.74 (s, 3H), 3.93 (s, 3H), 3.98 (s, 3H), 5.93 (dd, J = 10.0, 1.8 Hz, 1H), 6.12 (dd, J = 10.0, 1.8 Hz, 1H), 6.20-7.00 (br, 3H), 6.51 (dd, J = 10.0, 2.7 Hz,1H), 6.73 (d, J = 1.8 Hz, 1H), 7.00-7.40 (br, 1H), 7.08 (d, J = 2.3 Hz, 1H), 7.24 (d, J = 2.7 Hz, 1H), 7.36 (dd, J = 10.0, 2.7 Hz, 1H), 7.48 (d, J = 2.3 Hz, 1H) ; ^{13}C NMR (acetone-d6): δ 55.52, 56.08, 56.51, 56.64, 59.55, 98.18, 104.08, 105.36, 109.58, 113.22, 124.85, 129.95, 130.06, 131.61, 132.52, 132.57, 135.85, 136.40, 138.33, 141.89,148.45, 154.38, 160.37, 160.58, 162.45, 162.85, 174.61, 186.40, 187.54 (two carbons are missing at the 25 ºC measurement); (+)-ESI-HRMS. Calcd for $C_{32}H_{24}Na_1O_8$ ($M+Na^+$) 559.1369 Found: 559.1357.

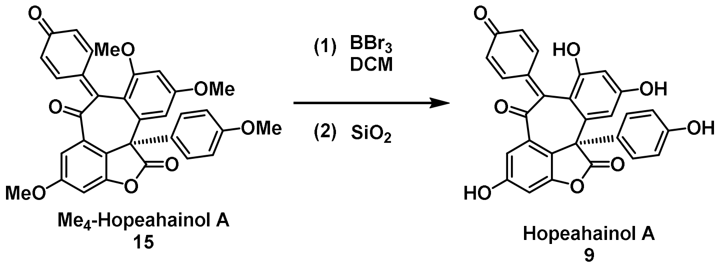

Hopeahainol A
9

Hopeahainol A (9); To a solution of **15** (3.5 mg, 0.0065 mmol, 1 equiv.) in DCM (1 mL), BBr_3 solution (1.0 M in DCM, 195 µL, 30 equiv.) was added at -78 °C under argon atmosphere and the mixture was warmed up to -20 °C with stirring. After 24 hours, saturated aqueous $NaHCO_3$ was added and the reaction mixture was extracted with AcOEt. The combined organic layers were dried over Na_2SO_4, and the solvent was removed in *vacuo*. The crude mixture was loaded onto a preparative TLC plate (silica gel) and was allowed to stand in the air for 6 h at ambient temperature before it was developed (DCM:MeOH 10:1). **Hopeahainol A (9)** (1.6 mg, 0.0032mmol, 50%) was isolated as an orange-red amorphous solid; IR(ATR) ν 3244, 2924, 1798, 1700, 1632, 1592, 1447, 1334, 1260, 1163, 1076, 1037, 865, 808 cm^{-1}; ^1H NMR (acetone-d_6, –30 ºC): δ 6.00 (dd, *J* = 10.0, 2.1 Hz, 1H), 6.12 (dd, *J* = 10.3, 2.1 Hz, 1H), 6.45 (dd, *J* = 8.9, 2.1 Hz, 1H), 6.48 (d, *J* = 2.1 Hz, 1H), 6.52 (dd, *J* = 8.9, 2.1 Hz, 1H), 6.66 (dd, *J* = 8.3, 2.1 Hz, 1H), 6.69 (dd, *J* = 10.0, 2.8 Hz, 1H), 6.90 (d, *J* = 2.1 Hz, 1H), 7.10 (d, *J* = 2.1 Hz, 1H), 7.11 (dd, *J* = 8.3, 2.1 Hz, 1H), 7.37 (dd, *J* = 10.3, 2.8 Hz, 1H), 7.44 (d, *J* = 2.1 Hz, 1H) ; ^{13}C NMR (acetone-d_6, –30 ºC): δ 59.17, 102.21, 104.65, 106.13, 110.25, 110.96, 114.97, 117.62, 123.67, 127.15, 129.35, 129.39, 130.43, 130.73, 132.24, 135.26, 136.47, 138.89, 142.10, 149.44, 154.04, 157.85, 158.27, 159.92, 159.97, 174.79, 186.58, 187.58; (+)-ESI-HRMS. Calcd for $C_{28}H_{16}Na_1O_8$ ($M+Na^+$) 503.0743 Found: 503.0754.

Reported[7] Hopeahainol A;^1H NMR (acetone-d_6, –30 ºC): δ 6.05 (dd, *J* = 10.0, 1.3 Hz, 1H), 6.17 (dd, *J* = 10.2, 1.3 Hz, 1H), 6.46 (dd, *J* = 8.5, 2.1 Hz, 1H), 6.49 (d, *J* = 1.7 Hz, 1H), 6.53 (dd, *J* = 8.5, 1.9 Hz, 1H), 6.68 (dd, *J* = 8.6, 2.1 Hz, 1H), 6.74 (dd, *J* = 10.0, 2.4 Hz, 1H), 6.90 (d, *J* = 1.7 Hz, 1H), 7.11 (d, *J* = 2.1 Hz, 1H), 7.12 (dd, *J* = 8.6, 1.9 Hz,1H) 7.41 (dd, *J* = 10.2, 2.4 Hz, 1H), 7.44 (d, *J* = 2.1 Hz, 1H) ; ^{13}C NMR (acetone-d_6, –30 ºC): δ 59.1, 102.1, 104.6, 106.0, 109.9, 110.9, 114.8, 117.5, 123.4, 127.1, 129.1, 129.2, 130.3, 130.7, 132.0, 135.0, 136.8, 139.3, 142.0, 150.3, 153.9, 157.8, 158.3, 160.0, 160.0, 174.7, 186.9, 187.6.

	1H NMR				
H	Synthetic Hopeahainol A		Natural Hopeahainol A		Δx (ppm)
1	6.00	(dd, J = 10.0, 2.1 Hz, 1H)	6.05	(dd, J = 10.0, 1.3 Hz, 1H)	-0.05
2	6.12	(dd, J = 10.3, 2.1 Hz, 1H)	6.17	(dd, J = 10.2, 1.3 Hz, 1H)	-0.05
3	6.45	(dd, J = 8.9, 2.1 Hz, 1H)	6.46	(dd, J = 8.5, 2.1 Hz, 1H)	-0.01
4	6.48	(d, J = 2.1 Hz, 1H)	6.49	(d, J = 1.7 Hz, 1H)	-0.01
5	6.52	(dd, J = 8.9, 2.1 Hz, 1H)	6.53	(dd, J = 8.5, 1.9 Hz, 1H)	-0.01
6	6.66	(dd, J = 8.3, 2.1 Hz, 1H)	6.68	(dd, J = 8.6, 2.1 Hz, 1H)	-0.02
7	6.69	(dd, J = 10.0, 2.8 Hz, 1H)	6.74	(dd, J = 10.0, 2.4 Hz, 1H)	-0.05
8	6.90	(d, J = 2.1 Hz, 1H)	6.90	(d, J = 1.7 Hz, 1H)	0.00
9	7.10	(d, J = 2.1 Hz, 1H)	7.11	(d, J = 2.1 Hz, 1H)	-0.01
10	7.11	(dd, J = 8.3, 2.1 Hz, 1H)	7.12	(dd, J = 8.6, 1.9 Hz,1H)	-0.01
11	7.37	(dd, J = 10.3, 2.8 Hz, 1H)	7.41	(dd, J = 10.2, 2.4 Hz, 1H)	-0.04
12	7.44	(d, J = 2.1 Hz, 1H)	7.44	(d, J = 2.1 Hz, 1H)	0.00

	13C NMR		
C	Synthetic Hopeahainol A	Natural Hopeahainol A	Δx (ppm)
1	59.2	59.1	0.1
2	102.2	102.1	0.1
3	104.7	104.6	0.1
4	106.1	106.0	0.1
5	110.3	109.9	0.3
6	111.0	110.9	0.1
7	115.0	114.8	0.2
8	117.6	117.5	0.1
9	123.7	123.4	0.3
10	127.2	127.1	0.1
11	129.4	129.1	0.3
12	129.4	129.2	0.2
13	130.4	130.3	0.1
14	130.7	130.7	0.0
15	132.2	132.0	0.2
16	135.3	135.0	0.3
17	136.5	136.8	-0.3
18	138.9	139.3	-0.4
19	142.1	142.0	0.1
20	149.4	150.3	-0.9
21	154.0	153.9	0.1
22	157.9	157.8	0.0
23	158.3	158.3	0.0
24	159.9	160.0	-0.1
25	160.0	160.0	0.0
26	174.8	174.7	0.1
27	186.6	186.9	-0.3
28	187.6	187.6	0.0

Supplementary Fig. 27. Comparison of ^{1}H and ^{13}C NMR of synthetic and natural Hopeahainol B.

4.Experimental procedure of Kinetic resolution

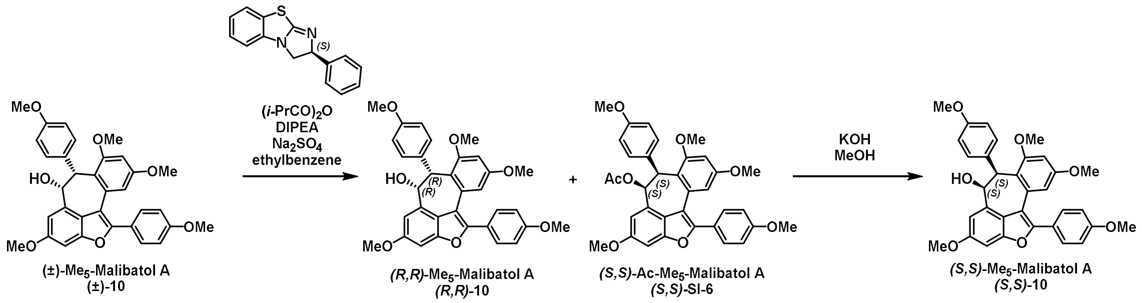

entry	scale	*(R,R)*-10	*(S,S)*-10 (over 2 steps)
1	0.4 mmol	33% (94% ee)	50% (-41% ee)
2	0.5 mmol	38% (94% ee)	50% (-42% ee)
3	0.5 mmol	33% (95% ee)	58% (-38% ee)
4	0.5 mmol	33% (97% ee)	52% (-40% ee)

To a solution of **(±)-10 (**0.4-0.5 mmol, 1 equiv.), *(S)*-Birman's cat.(1 equiv.) and Na_2SO_4 (500 mg/mmol) in ethylbenzene (0.5 M), DIPEA (0.6 equiv.) and (*i*-PrCO)$_2$O (0.6 equiv.) were added at room temperature and the mixture was stirred under argon atmosphere. After 3 hours, DIPEA (0.6 equiv.) and (*i*-PrCO)$_2$O (0.6 equiv.) was added to the reaction mixture again at room temperature and the resulting mixture was stirred under argon atmosphere. After 15 hours, MeOH was added and the mixture was stirred over 1 hour. 1N HCl aq. was added and the reaction mixture was extracted with DCM. The combined organic layers were washed 1N HCl aq. and saturated aqueous $NaHCO_3$. The combined organic layers were dried over Na_2SO_4, and the solvent was removed in *vacuo*. The residue was purified by silica gel column chromatography (SiO_2, Hexane/AcOEt = 4/1 to 2/1) to give ***(R,R)*-10** (33 - 38% yield, 94 - 97 %ee) as colorless amorphous solid and ***(S,S)*- SI-6**.

To a solution of ***(S,S)*- SI-6** (1 equiv.) in MeOH (0.1 M), KOH (3 equiv.) was added at 50 °C and the mixture was stirred under argon atmosphere. After 3 hours, 1N HCl aqueous was added and the reaction mixture was extracted with EtOAc. The combined organic layers were dried over Na_2SO_4, and the solvent was removed in *vacuo*. The residue was purified by silica gel column chromatography (SiO_2, Hexane/AcOEt = 4/1 to 2/1) to give ***(S,S)*-10** (50 - 58% yield, 39 - 42 %ee, over 2 steps) as colorless amorphous solid**.**

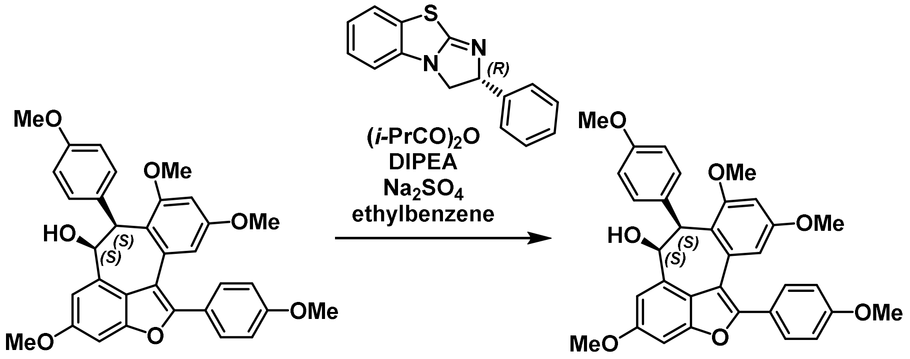

(S,S)-Me_5-Malibatol A
(S,S)-**10**

(S,S)-Me_5-Malibatol A
(S,S)-**10**

entry	(+)-Me-Malibatol A (alcohol) (3 steps)
1	23% (-97%ee)
2	31% (-95%ee)
3	29% (-98%ee)
4	30% (-97%ee)

To a solution of ***(S,S)*-10** (1 equiv.), *(R)*-Birman's cat.(1 equiv.) and Na_2SO_4 (500 mg/mmol) in ethylbenzene (0.5 M), DIPEA (0.6 equiv.) and (i-PrCO)$_2$O (0.6 equiv.) were added at room temperature and the mixture stirred under argon atmosphere. After 3 hours, DIPEA (0.6 equiv.) and (i-PrCO)$_2$O (0.6 equiv.) were added to the reaction mixture again at room temperature and the resulting mixure was stirred under argon atmosphere. After 15 hours, MeOH was added and the mixture was stirred over 1 hour. 1N HCl aq. was added and the reaction mixture was extracted with DCM. The combined organic layers were washed 1N HCl aq. and saturated aqueous $NaHCO_3$. The combined organic layers were dried over Na_2SO_4, and the solvent was removed in *vacuo*. The residue was purified by silica gel column chromatography (SiO_2, Hexane/AcOEt = 4/1 to 2/1) to give ***(S,S)*-10** (23 – 31% yield, 95 – 98%ee, over 3 steps) as colorless amorphous solid. The enantiomeric ratio of ***(R,R)*-10** and ***(S,S)*-10** were determined by HPLC analysis (Daicel Chiralcel OD-3; 320 nm; hexane/i-PrOH 80:20 ; 1 mL/min; 10.6 and 19.5 min)

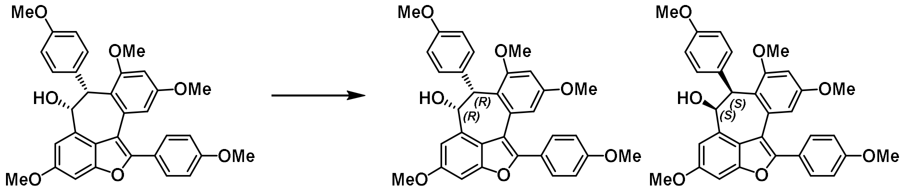

(±)-Me_5-Malibatol A
10
1027 mg (1.9 mmol)

(R,R)-Me_5-Malibatol A
(R,R)-10
360 mg (35%,95%ee)
$[\alpha]_D$ -141° (c=1, $CHCl_3$)

(S,S)-Me_5-Malibatol A
(S,S)-10
293 mg (29%,-97%ee)
$[\alpha]_D$ 144° (c=1,$CHCl_3$)

After kinetic resolution, Each of the combined ***(R,R)*-10** (entry1-4) and ***(S,S)*-10** (entry1-4) was used for the asymmetric total synthesis of malibtol A and vaticahainols. Asymmetric synthetic pathway is the same as that for the racemic synthesis.

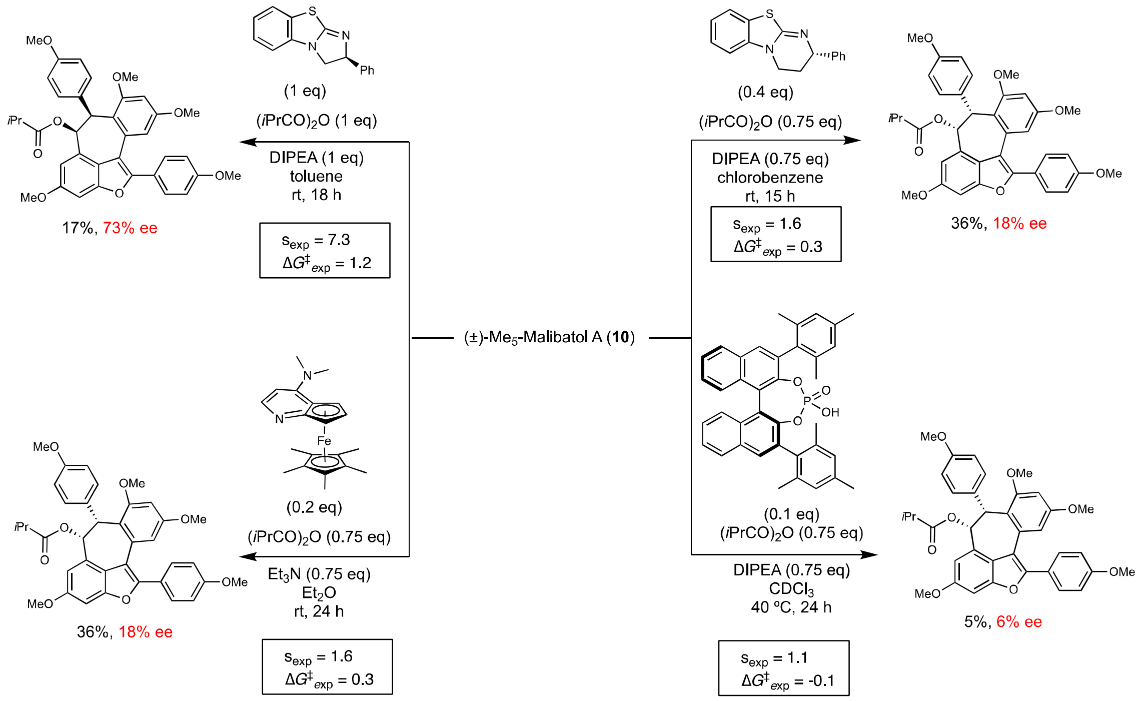

Supplementary Fig. 28. Attempted kinetic resolution.

5. Data of (+)- and (-)-Malibatol A and Vaticahainols

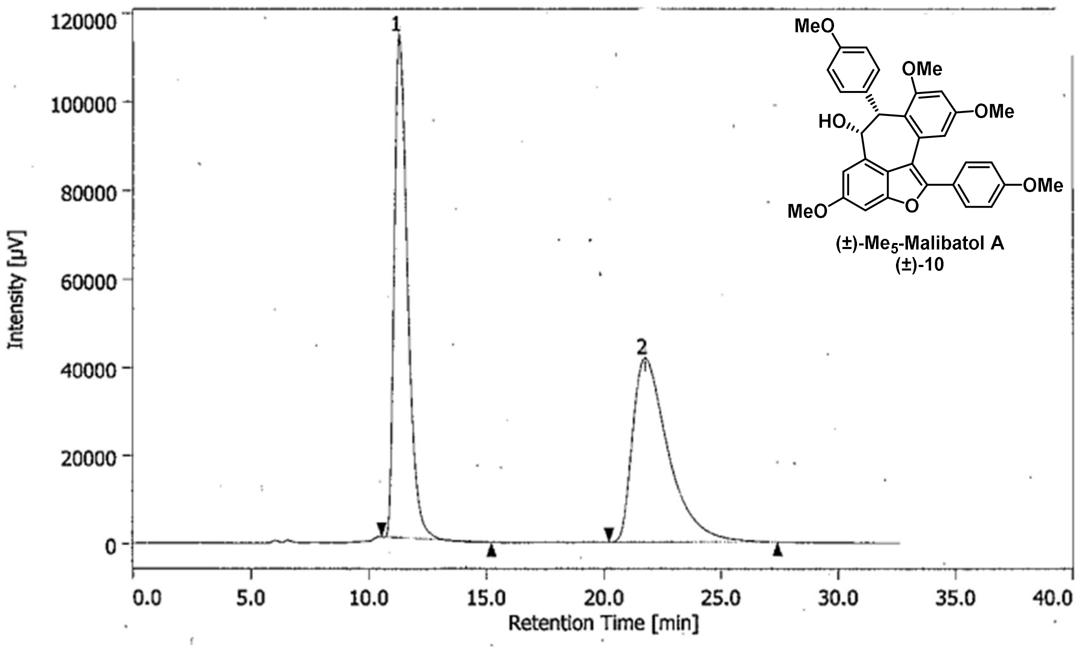

#	tR [min]	Area [µV·sec]	Area%	Height [µV]	Height%
1	11.292	4493850	50.194	113788	73.065
2	21.750	4459082	49.806	41948	26.935

Supplementary Fig. 29. HPLC analysis of (±)-Malibatol A.

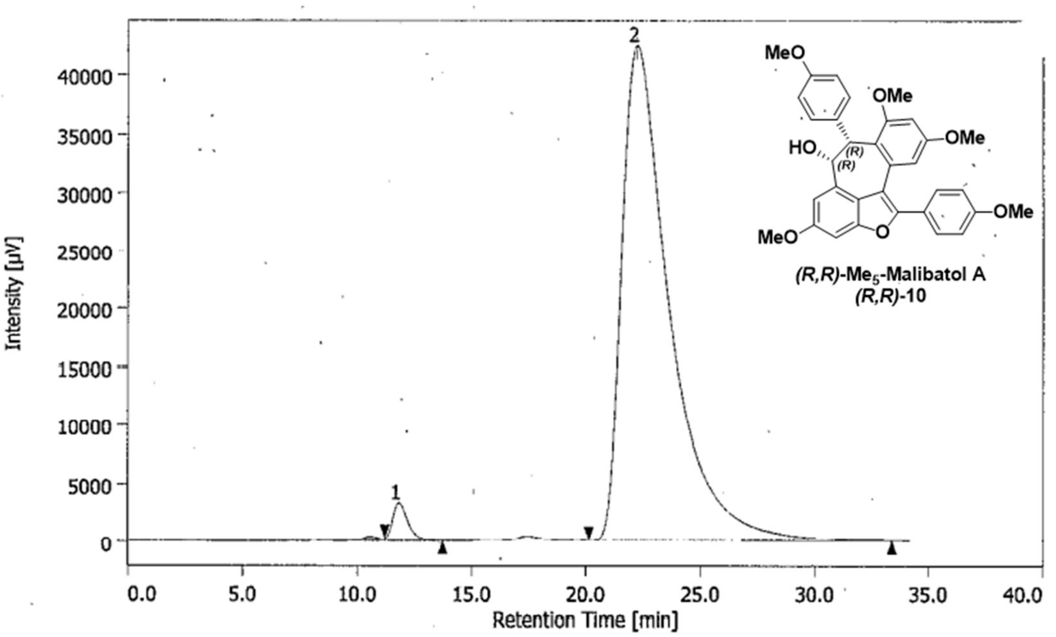

#	tR [min]	Area [µV·sec]	Area%	Height [µV]	Height%
1	11.825	153560	2.436	3298	7.231
2	22.225	6143856	97.562	42308	92.769

Supplementary Fig. 30. HPLC analysis of (*R*, *R*)-Malibatol A.

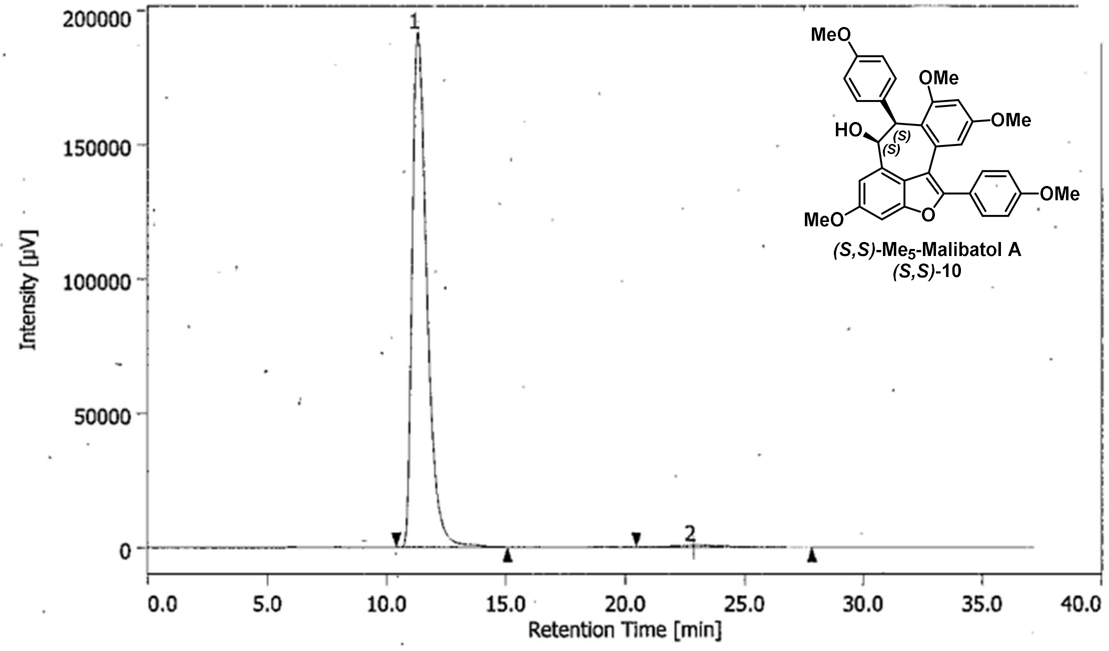

#	tR [min]	Area [µV·sec]	Area%	Height [µV]	Height%
1	11.325	8207291	98.489	191626	99.585
2	22.867	125920	1.511	798	0.415

Supplementary Fig. 31. HPLC analysis of (*S, S*)-Malibatol A.

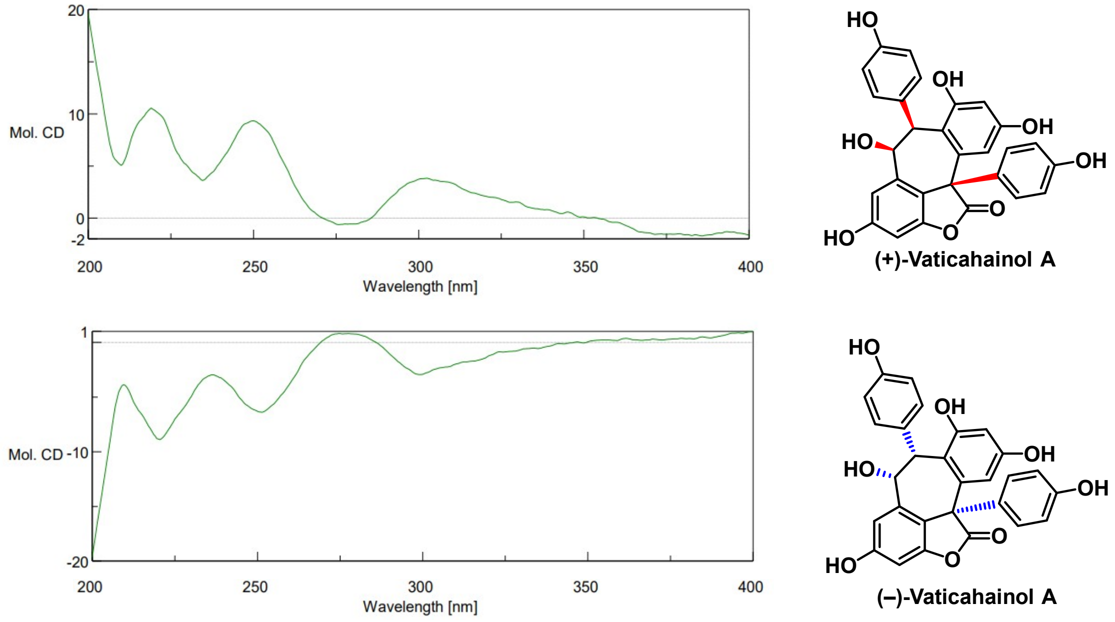

Supplementary Fig. 32. CD spectra of (+)- and (-)-Vaticahainol A.

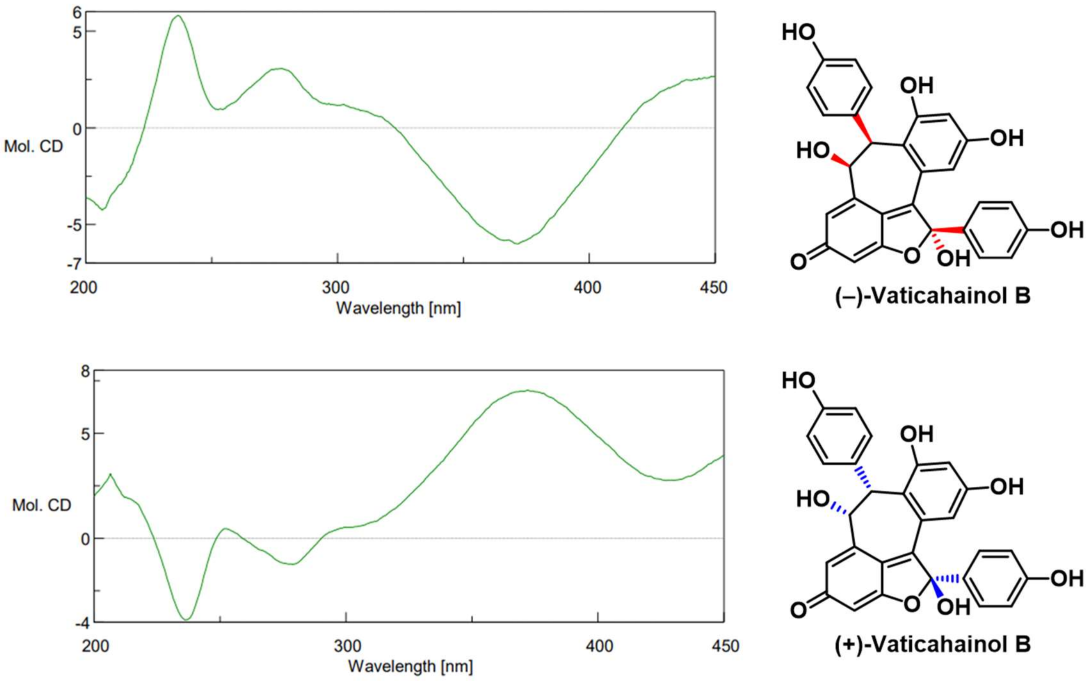

Supplementary Fig. 33. CD spectra of (+)- and (-)-Vaticahainol B.

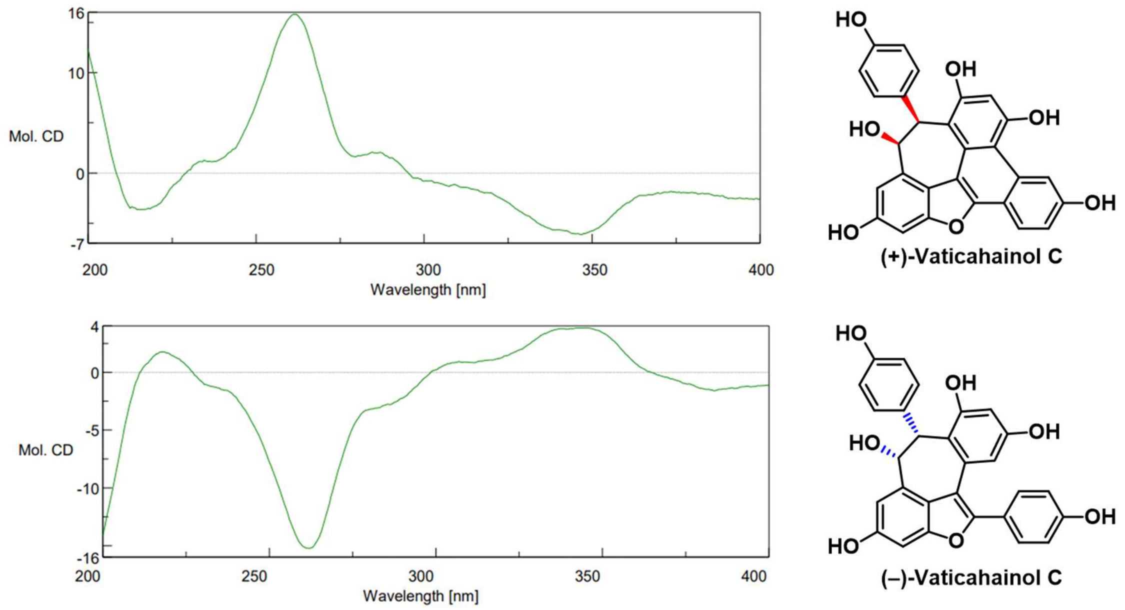

Supplementary Fig. 34. CD spectra of (+)- and (-)-Vaticahainol C.

6. ^{1}H and ^{13}C NMR Spectra

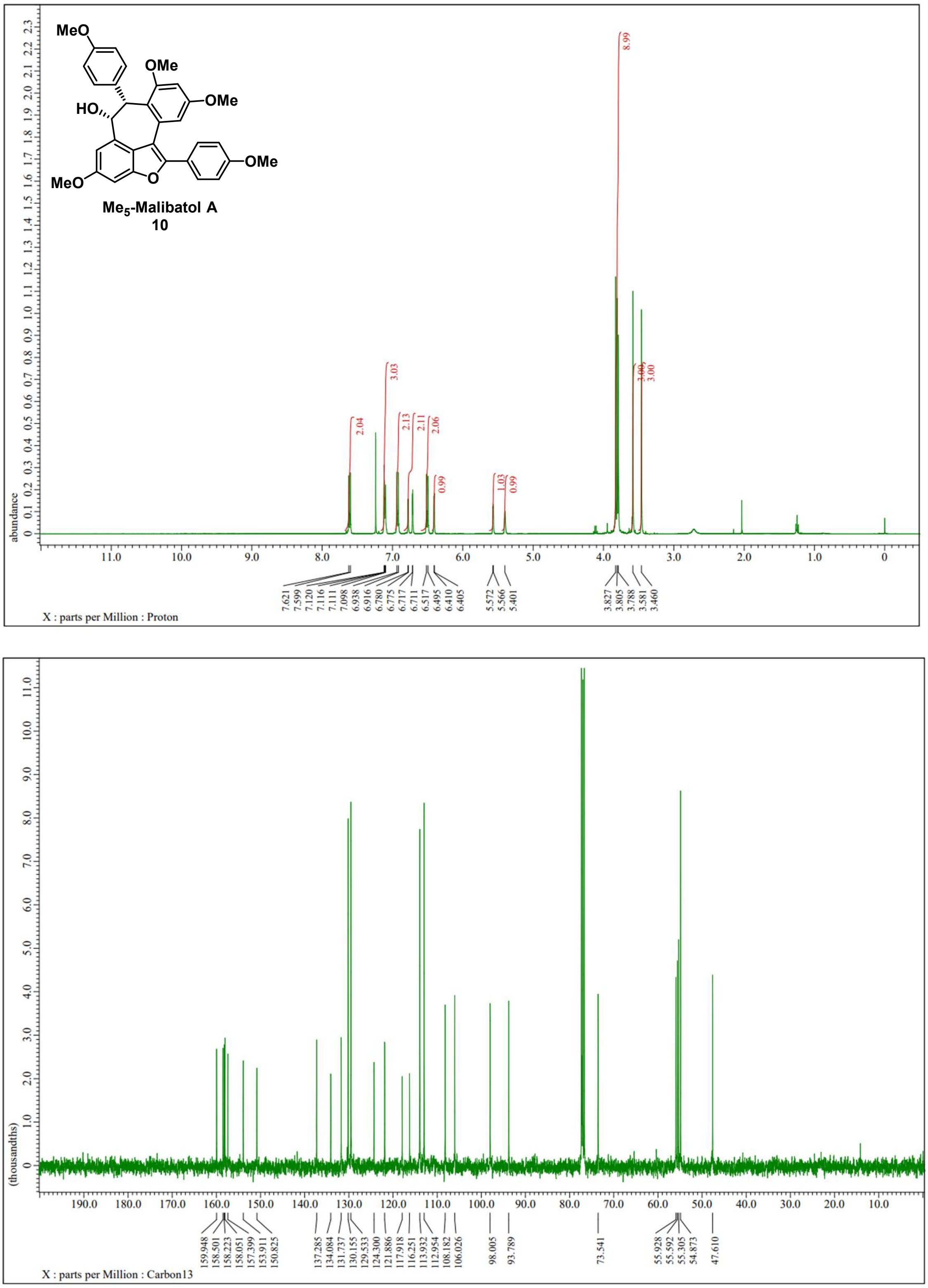

Supplementary Fig. 36. ^{1}H and ^{13}C NMR spectra of **10**.

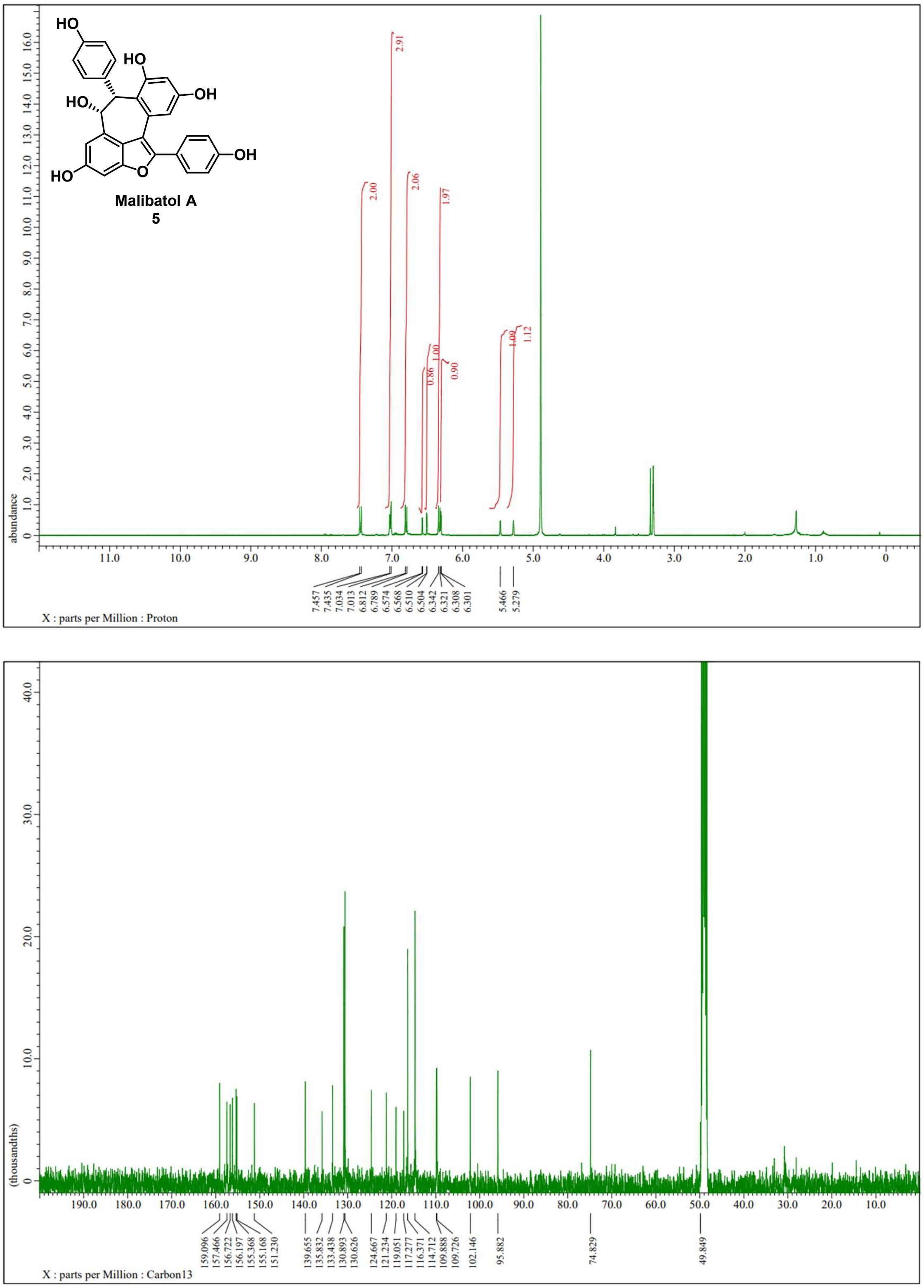

Supplementary Fig. 37. ^{1}H and ^{13}C NMR spectra of **5**.

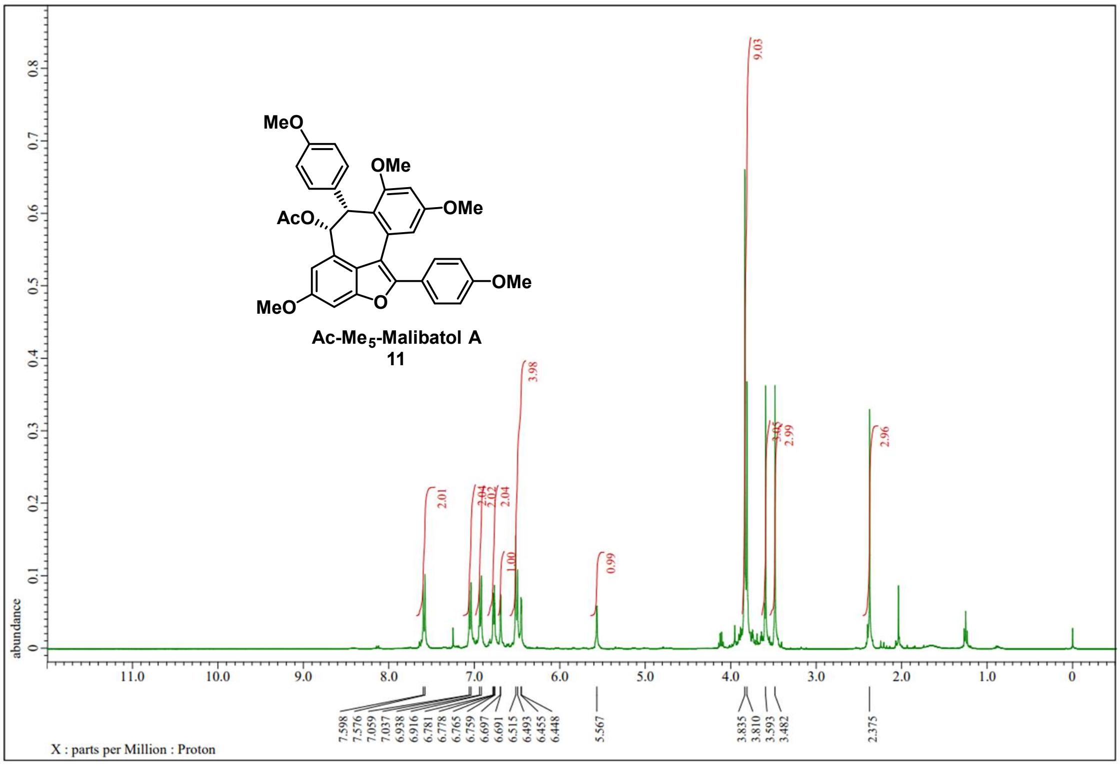

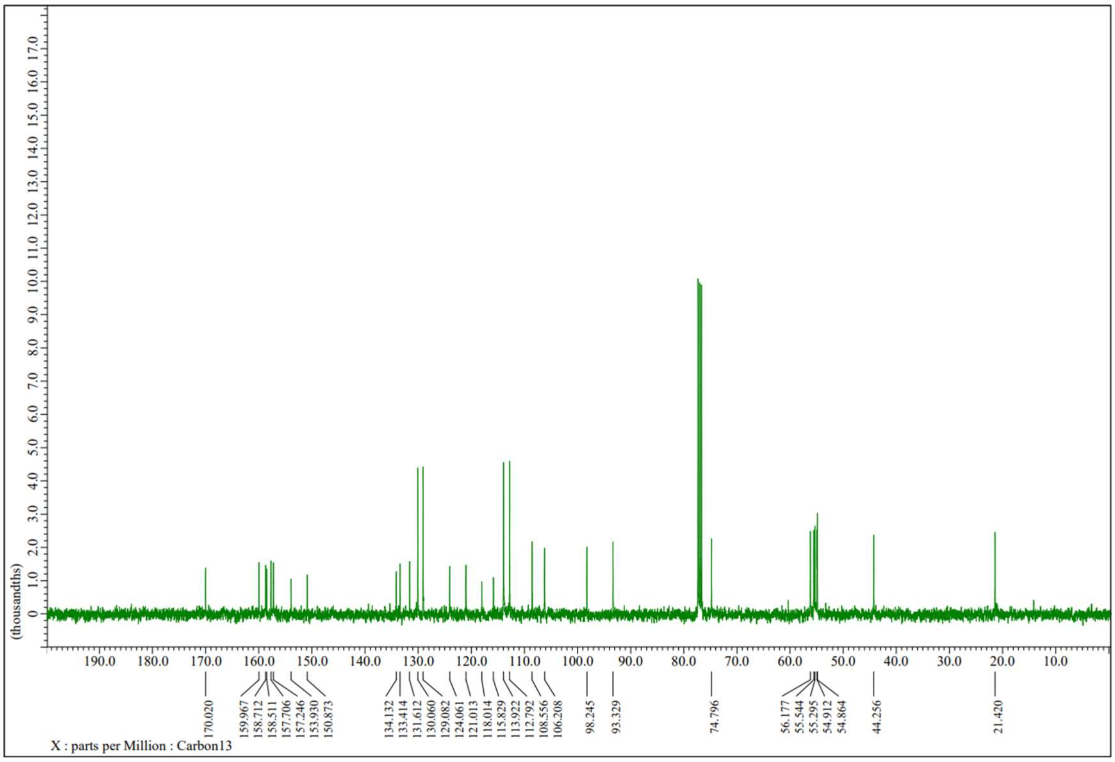

Supplementary Fig. 38. ^{1}H and ^{13}C NMR spectra of **11**.

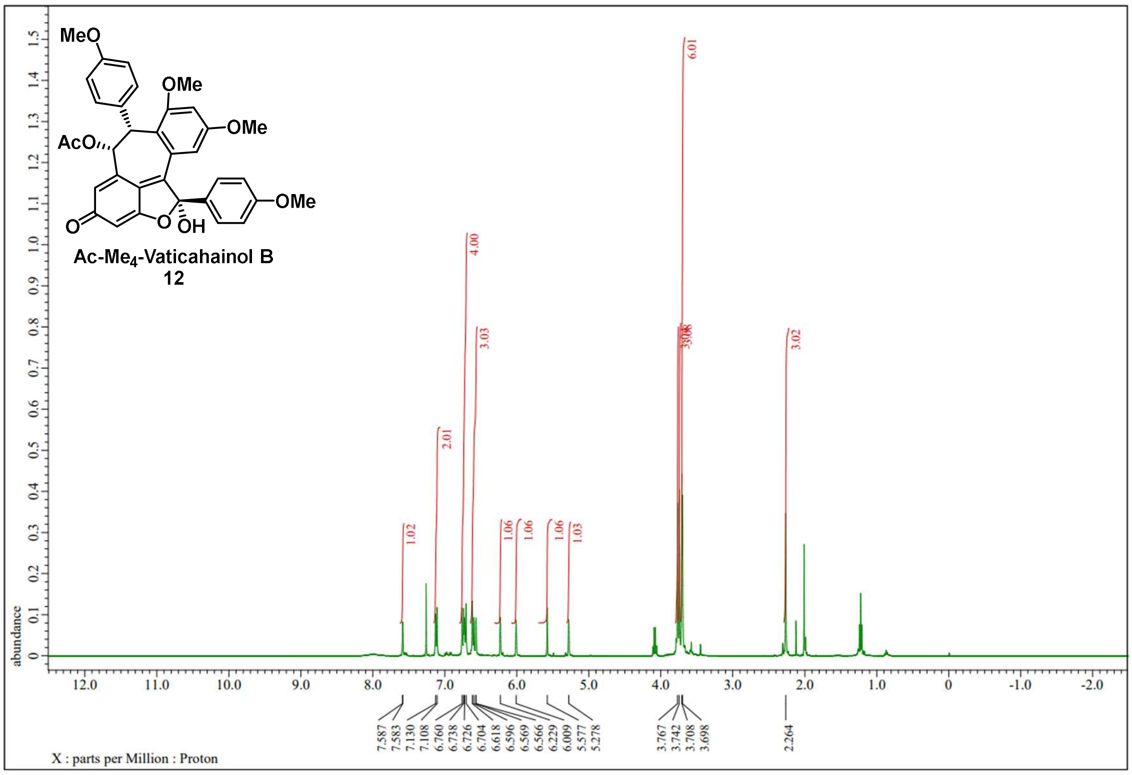

MeO
OMe
OMe
AcO
O
O
OH
OMe
Ac-Me4-Vaticahainol B
12
abundance
X : parts per Million : Proton

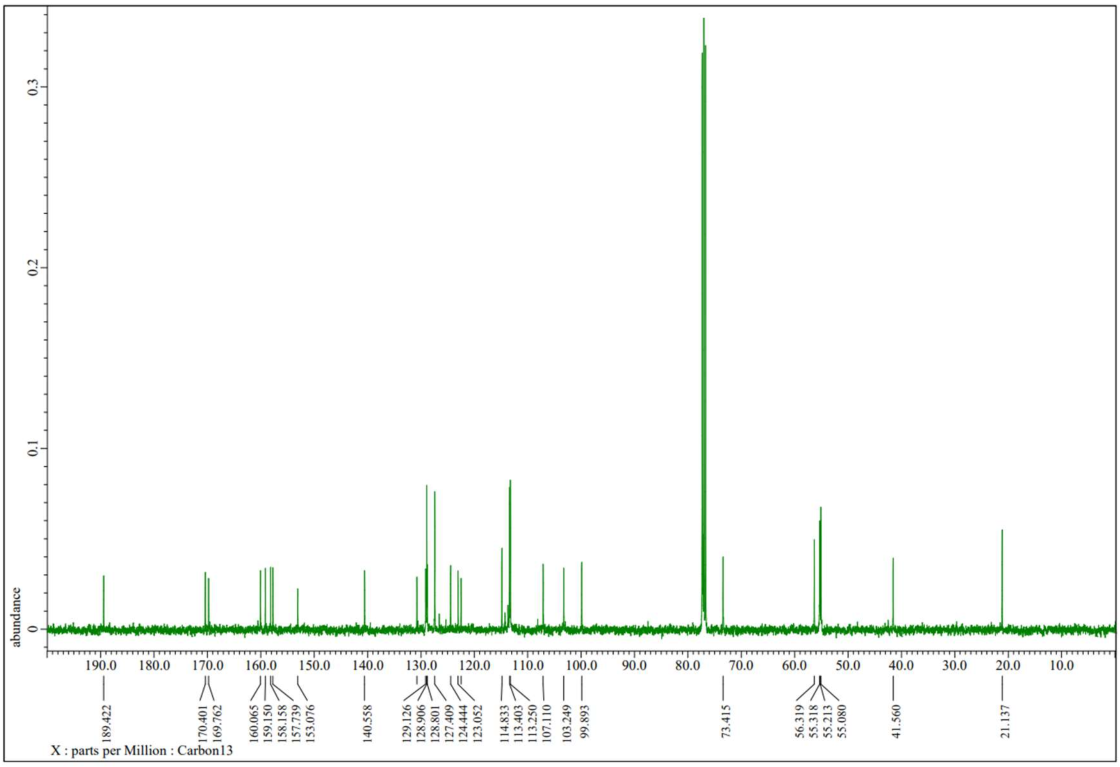

Supplementary Fig. 39. ^{1}H and ^{13}C NMR spectra of **12**.

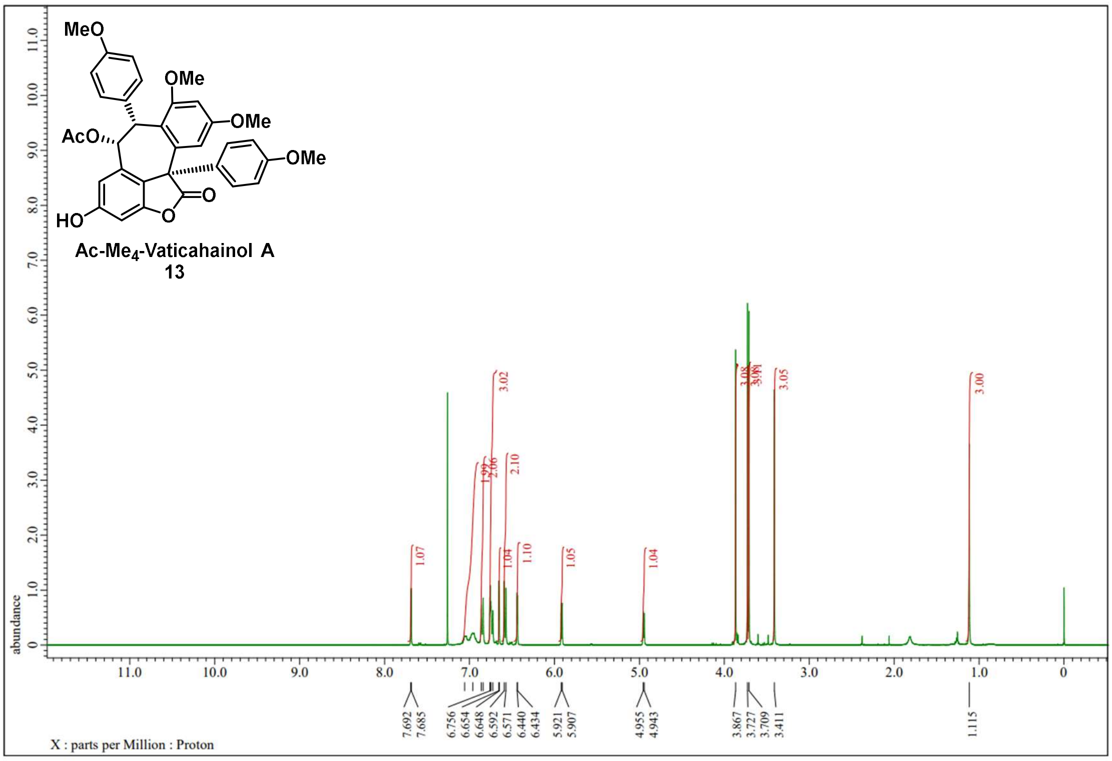

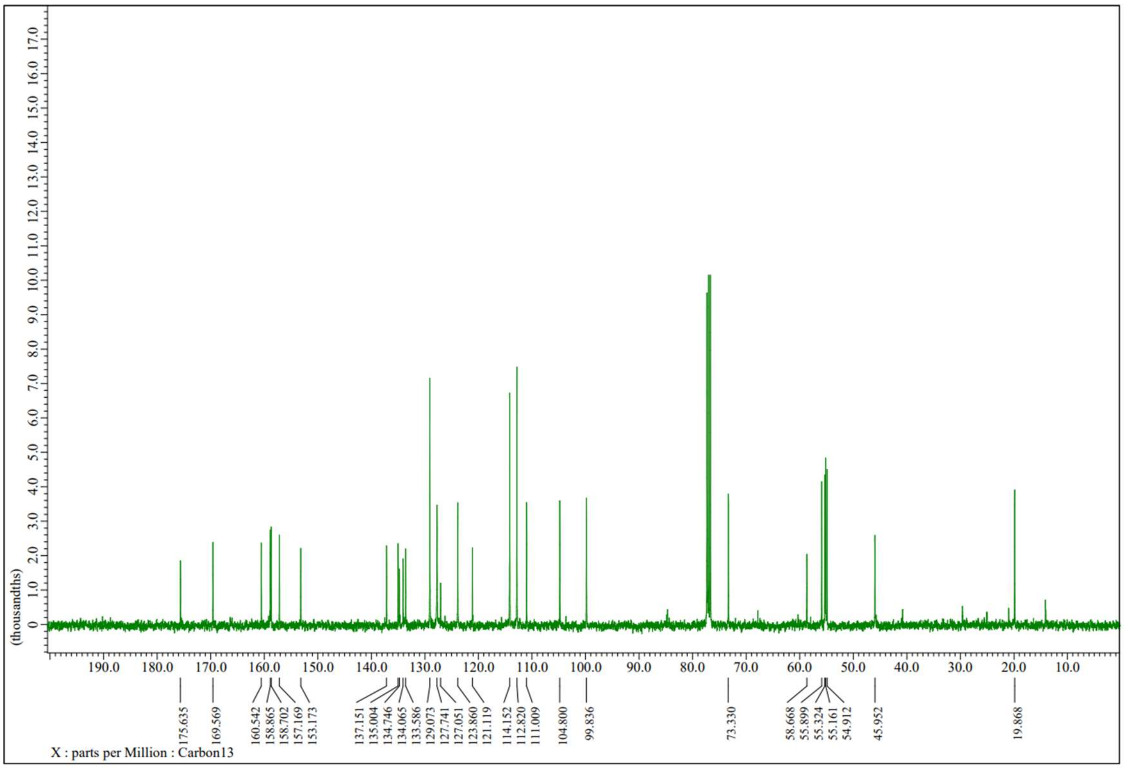

Supplementary Fig. 40. ^{1}H and ^{13}C NMR spectra of **13**.

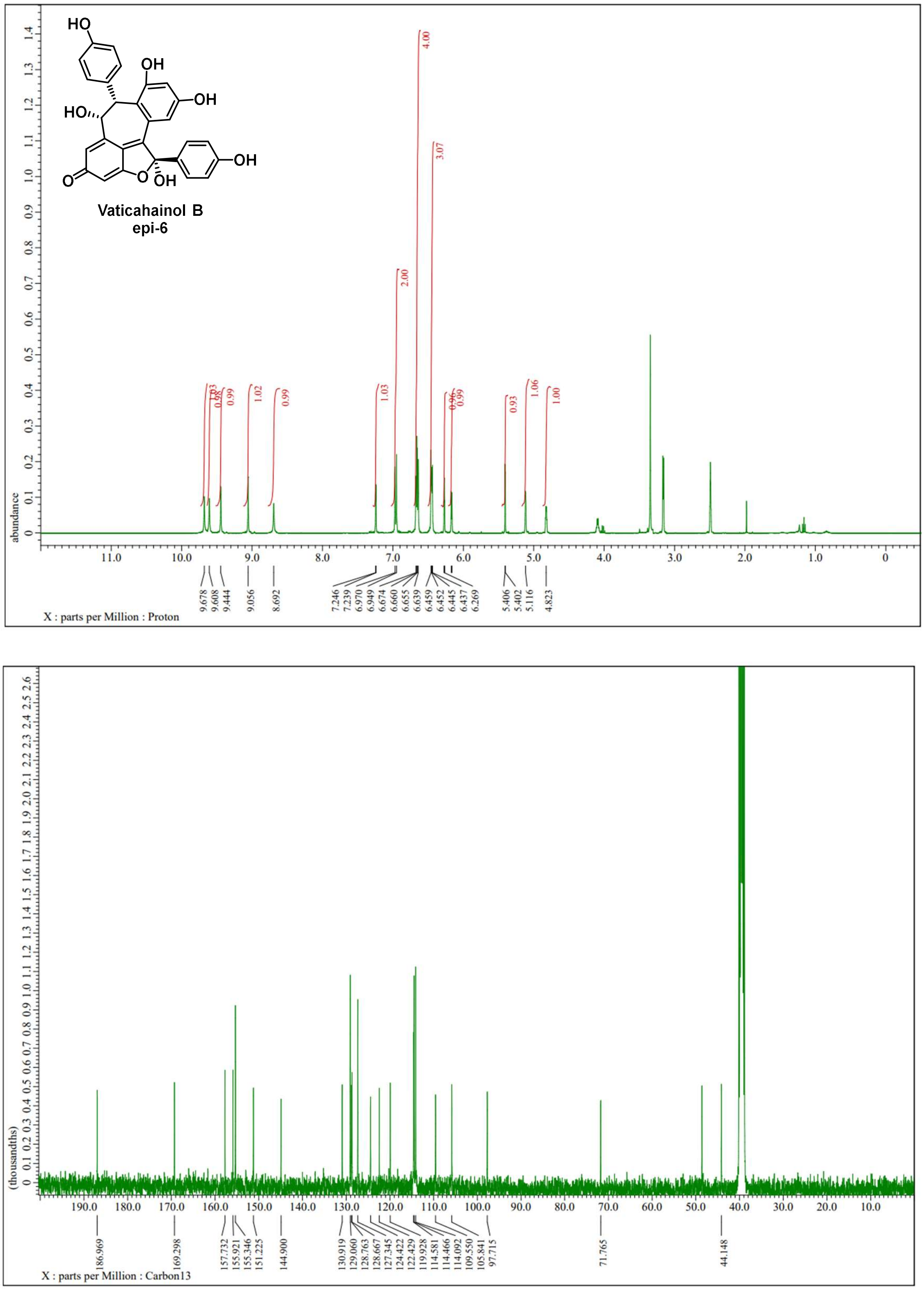

Supplementary Fig. 41. ^{1}H and ^{13}C NMR spectra of **epi-6**.

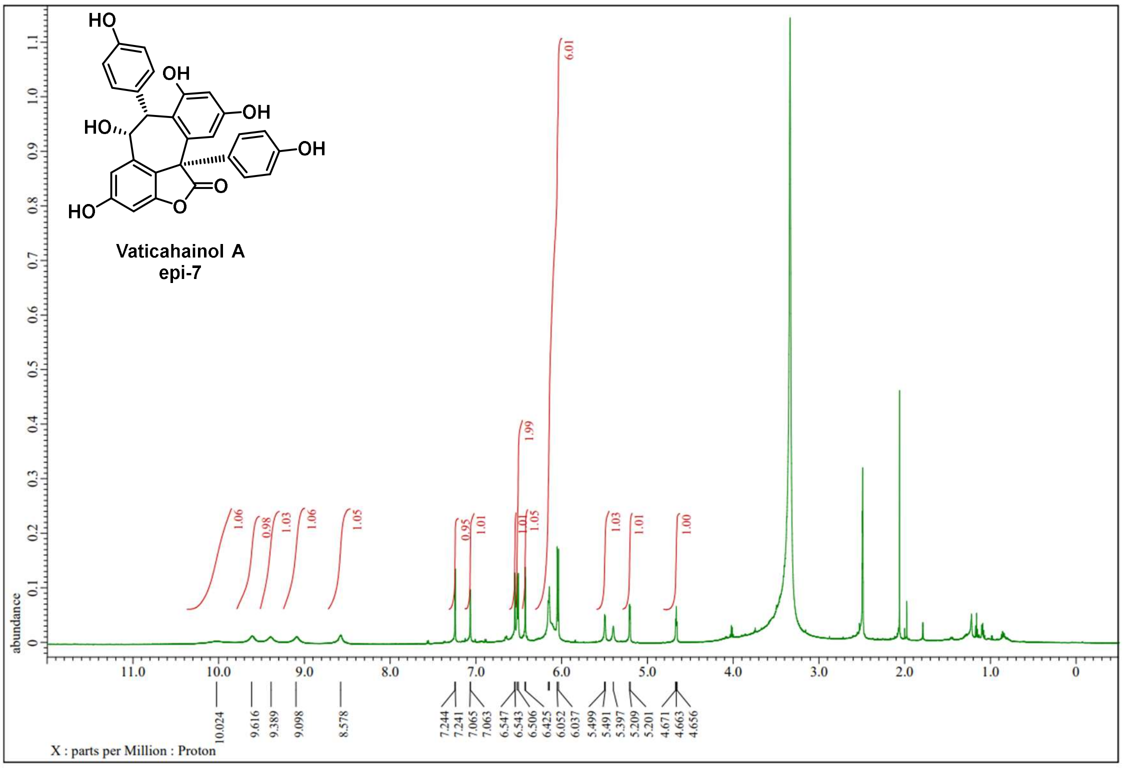

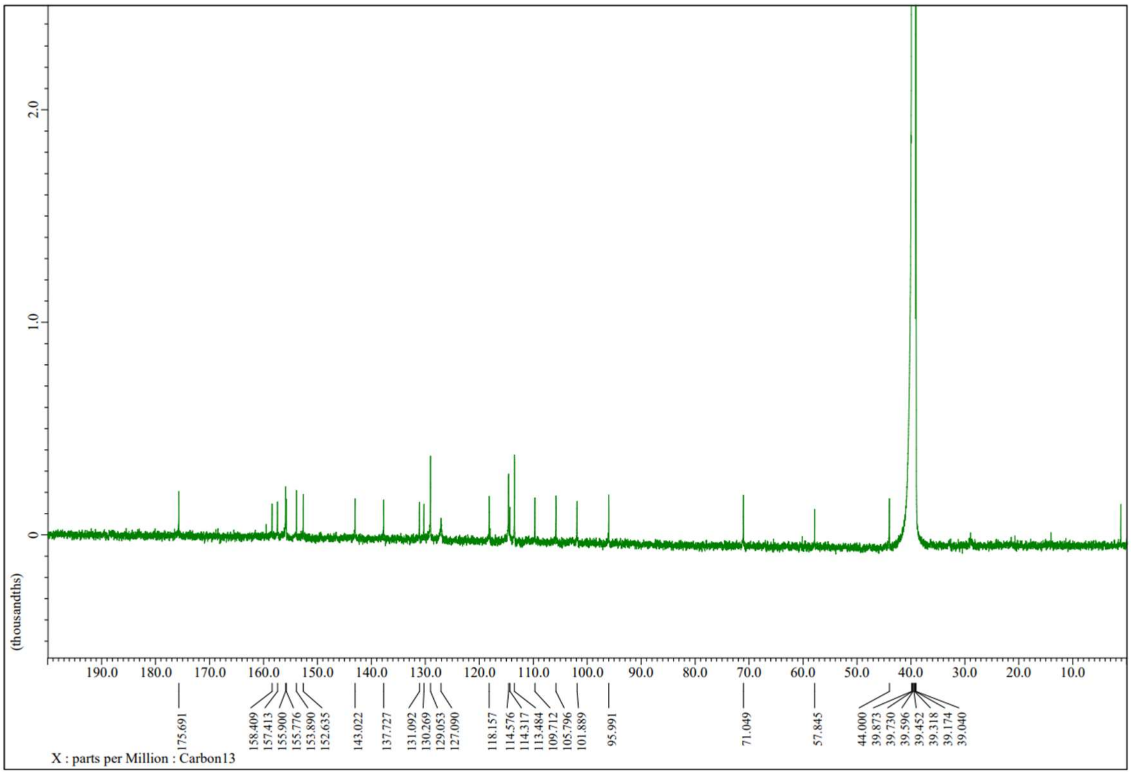

Supplementary Fig. 42. ^{1}H and ^{13}C NMR spectra of **epi-7**.

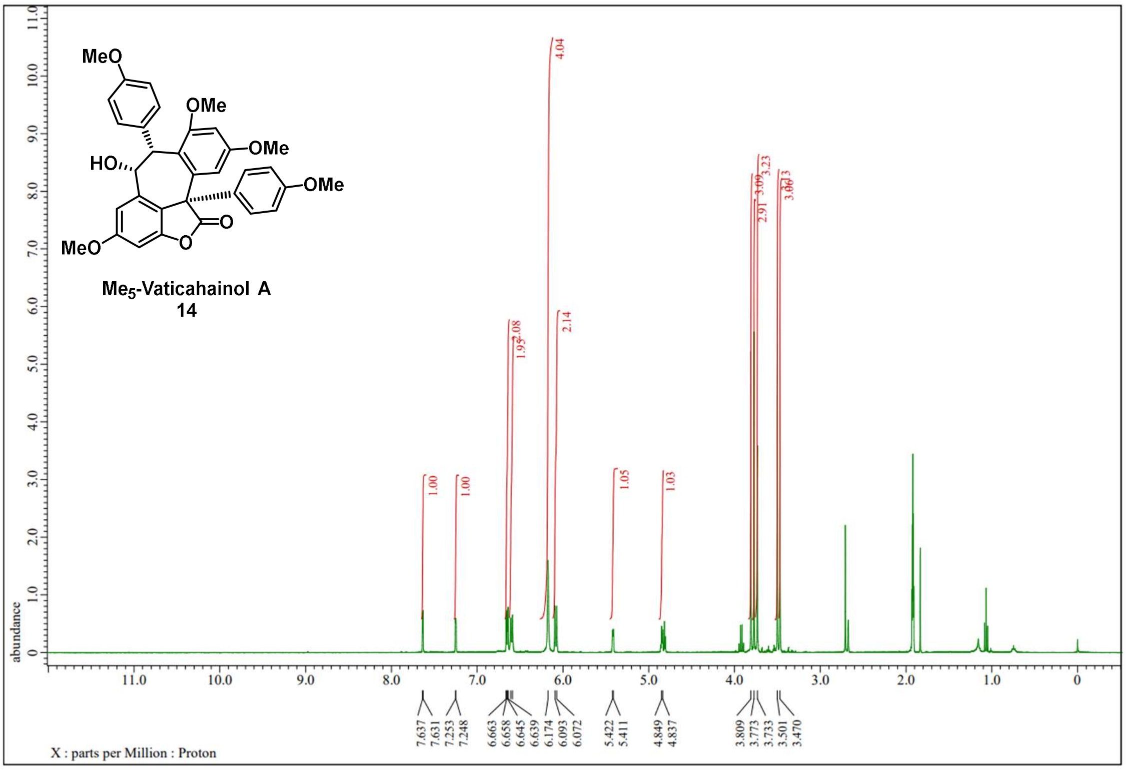
MeO
OMe
HO
OMe
OMe
MeO
O
O
Me5-Vaticahainol A
14
abundance
X : parts per Million : Proton

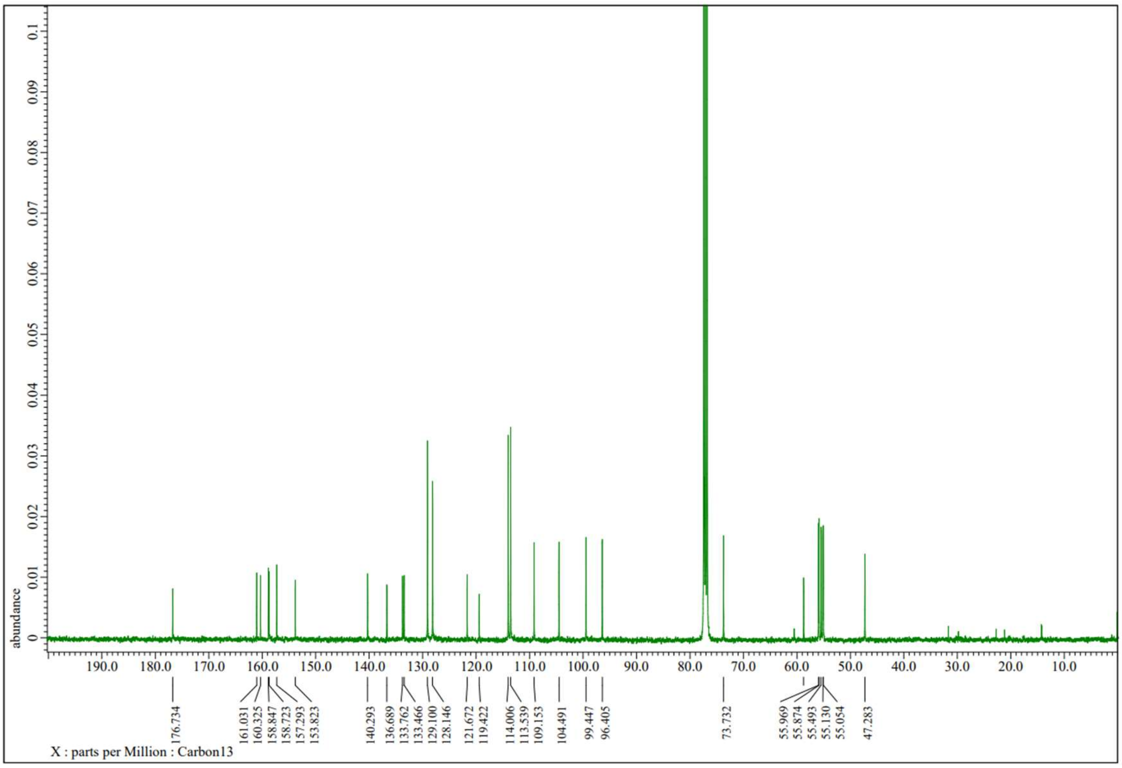

Supplementary Fig. 43. ^{1}H and ^{13}C NMR spectra of **14**.

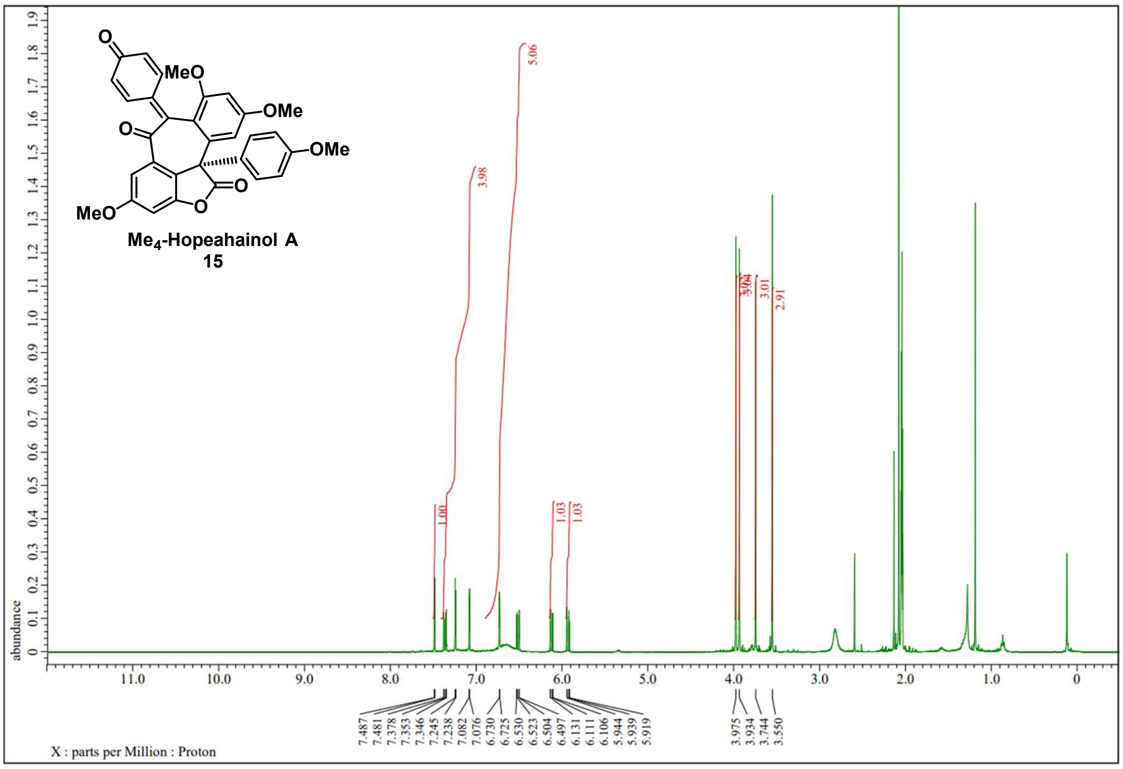

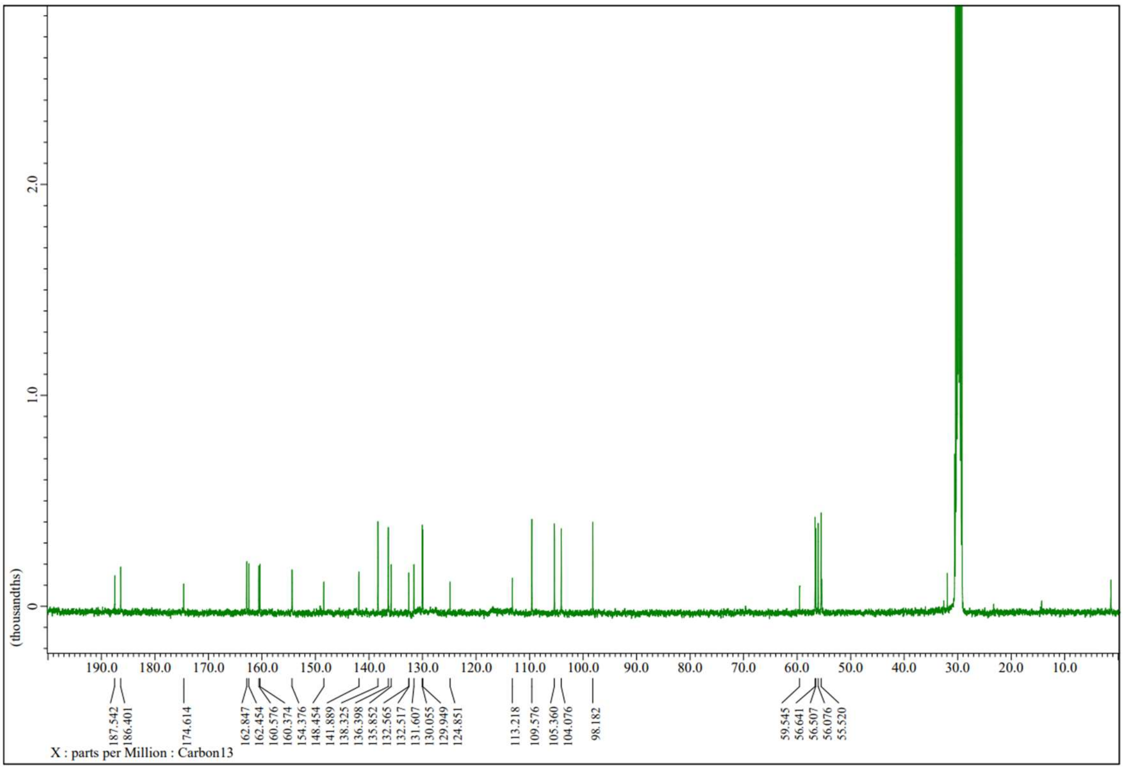

Supplementary Fig. 44. ^{1}H and ^{13}C NMR spectra of **15**.

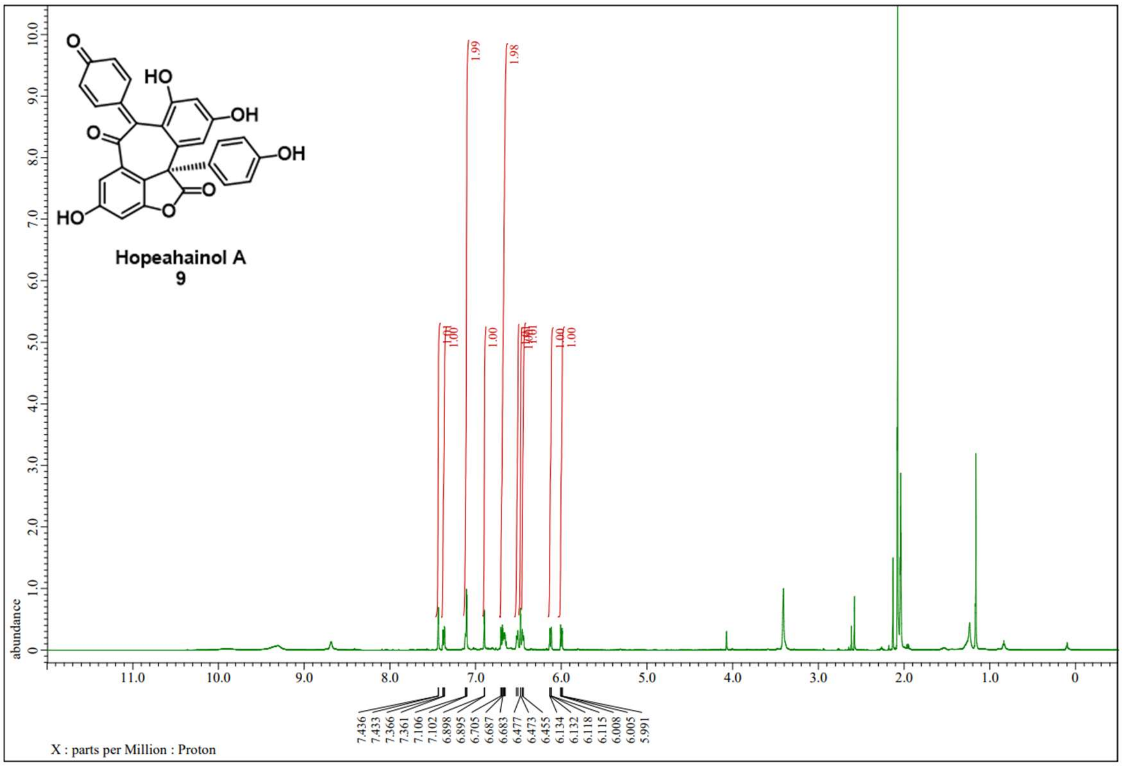

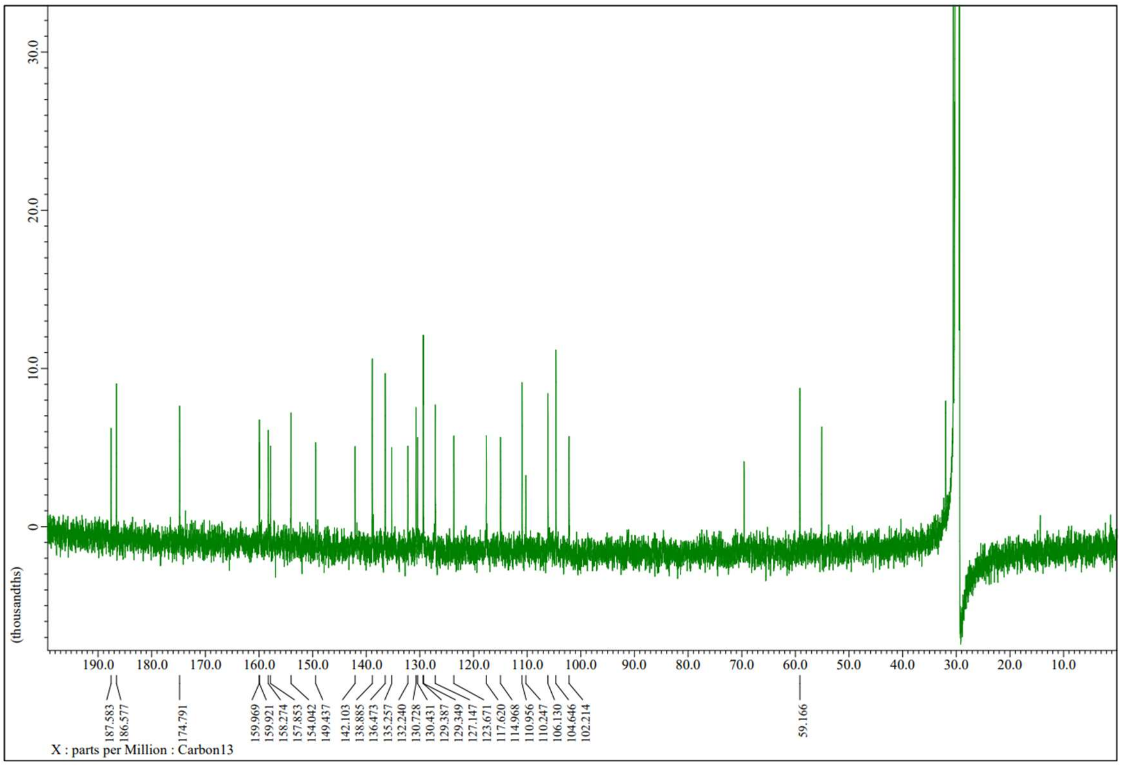

Supplementary Fig. 45. ^{1}H and ^{13}C NMR spectra of **9**.

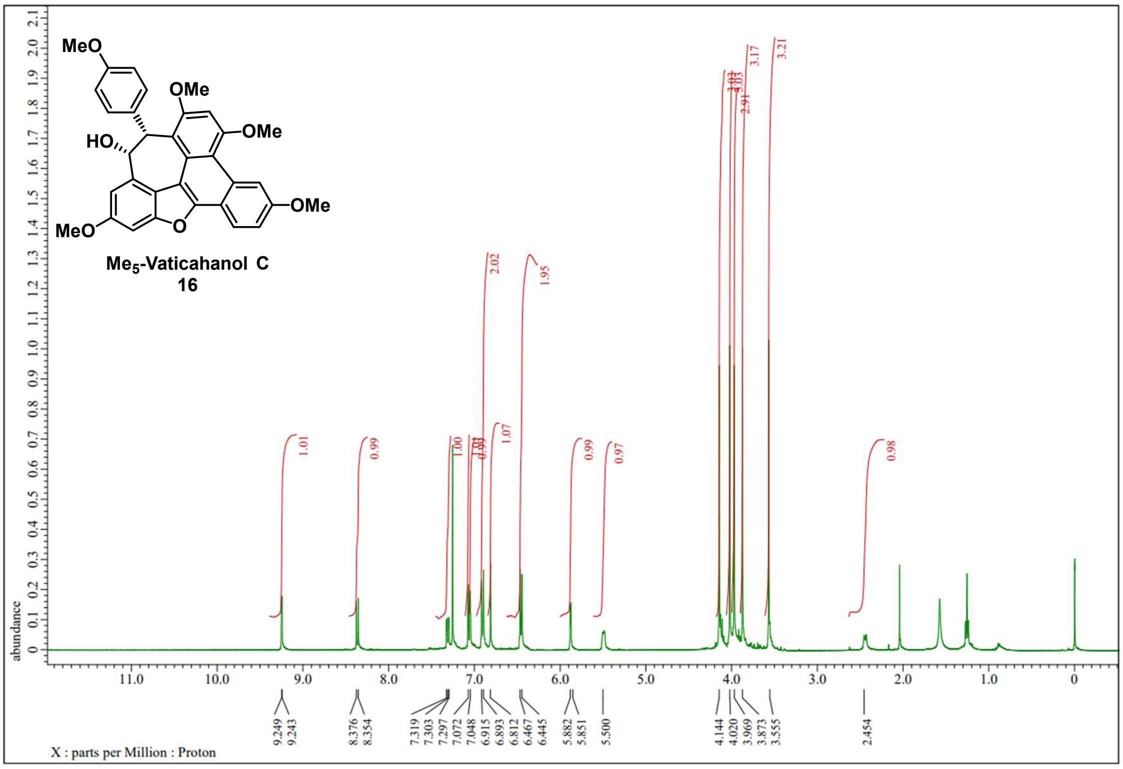
MeO
OMe
OMe
HO
MeO
O
OMe
Me5-Vaticahanol C
16
abundance
X : parts per Million : Proton

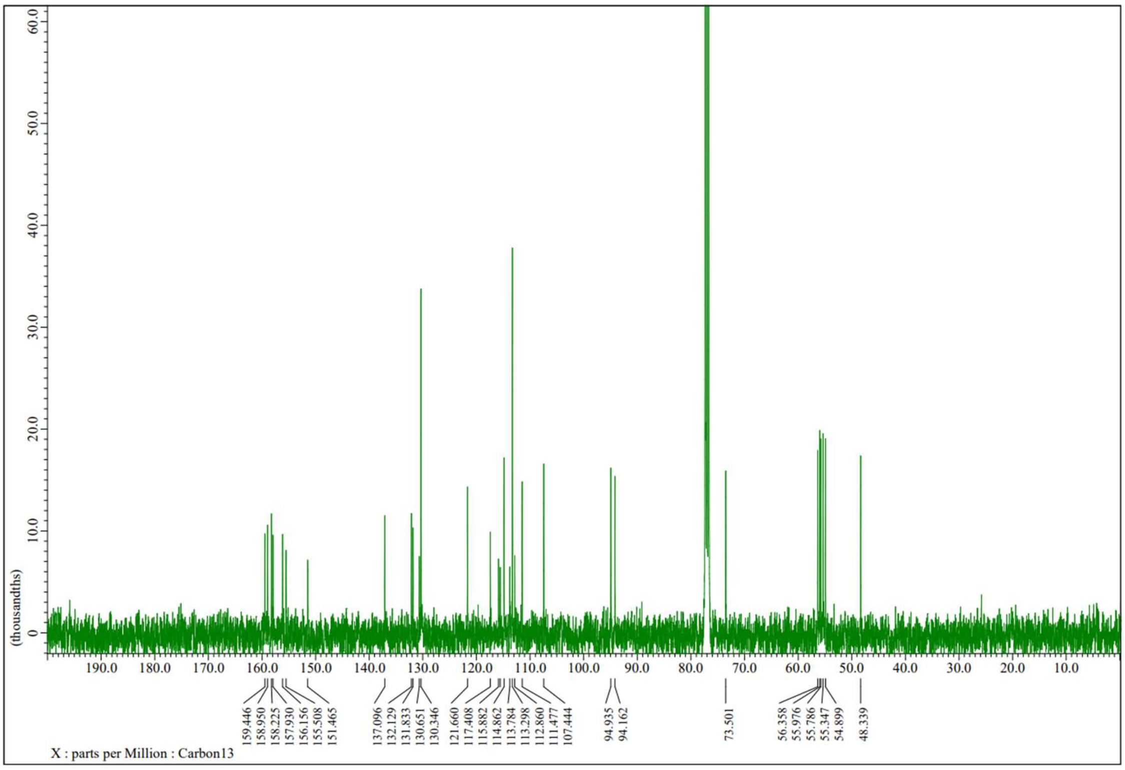

Supplementary Fig. 46. ^{1}H and ^{13}C NMR spectra of **16**.

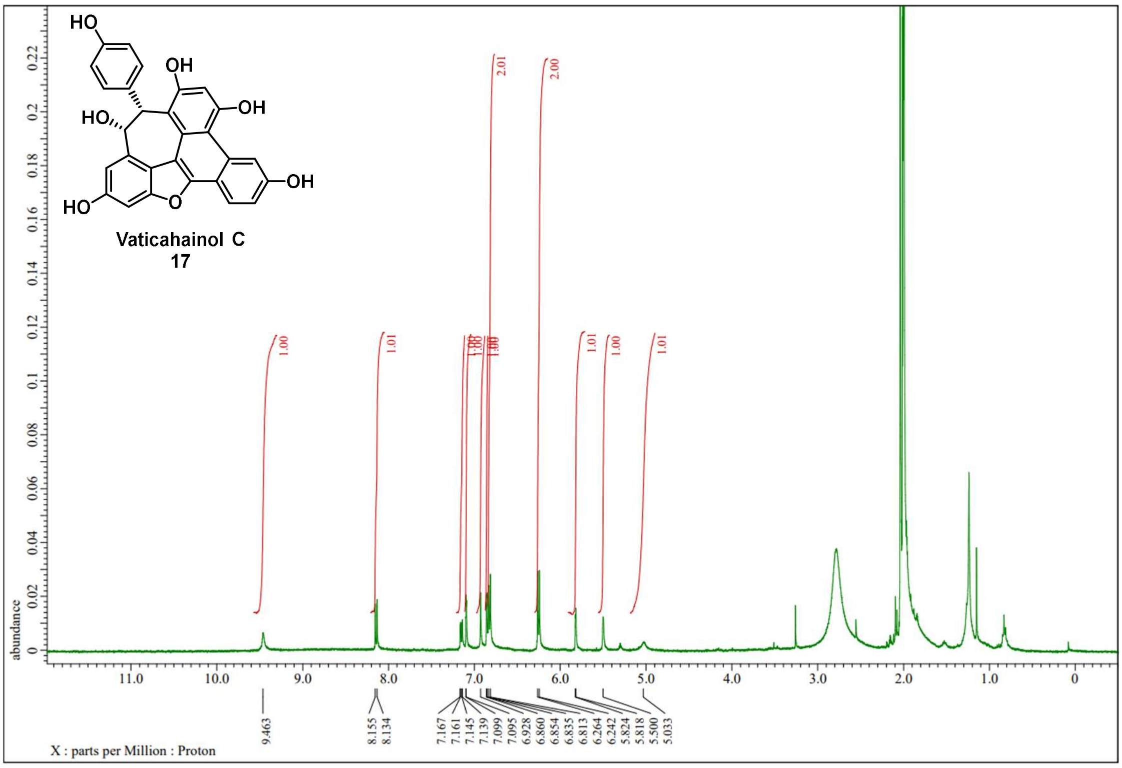

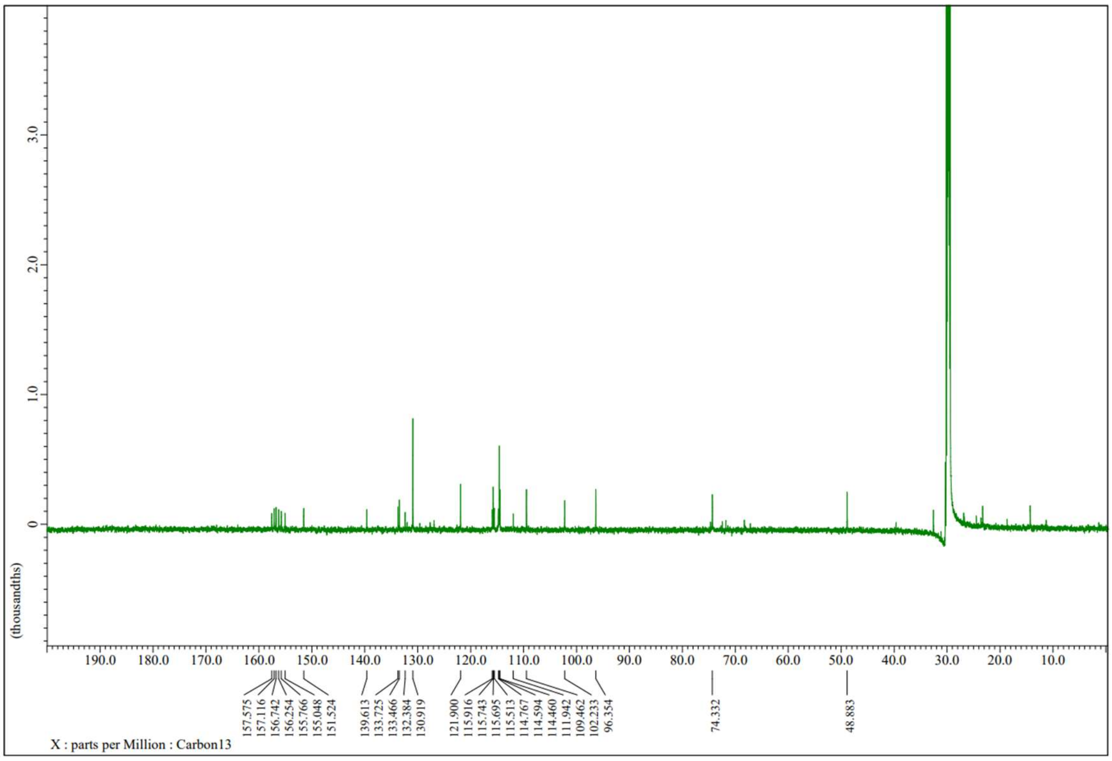

Supplementary Fig. 47. ^{1}H and ^{13}C NMR spectra of **17**.

7. Structural figures of X-ray crystallographic analysis

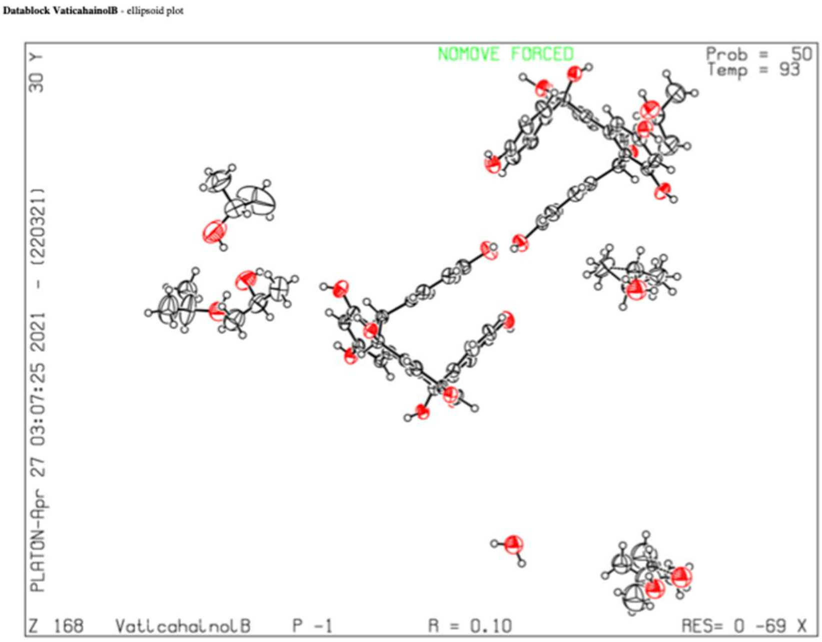

Supplementary Fig. 48. X-ray crystallographic analysis of **epi-6**.

Datablock 200720_adc_epiA_Me_tolhex - ellipsoid plot

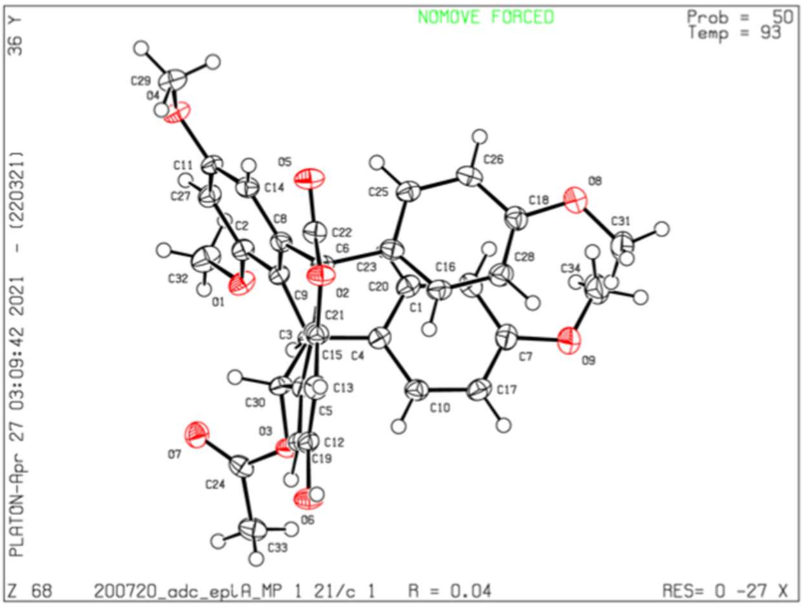

Supplementary Fig. 49. X-ray crystallographic analysis of **13**.

Datablock 201124_adc_MeVC_plus - ellipsoid plot

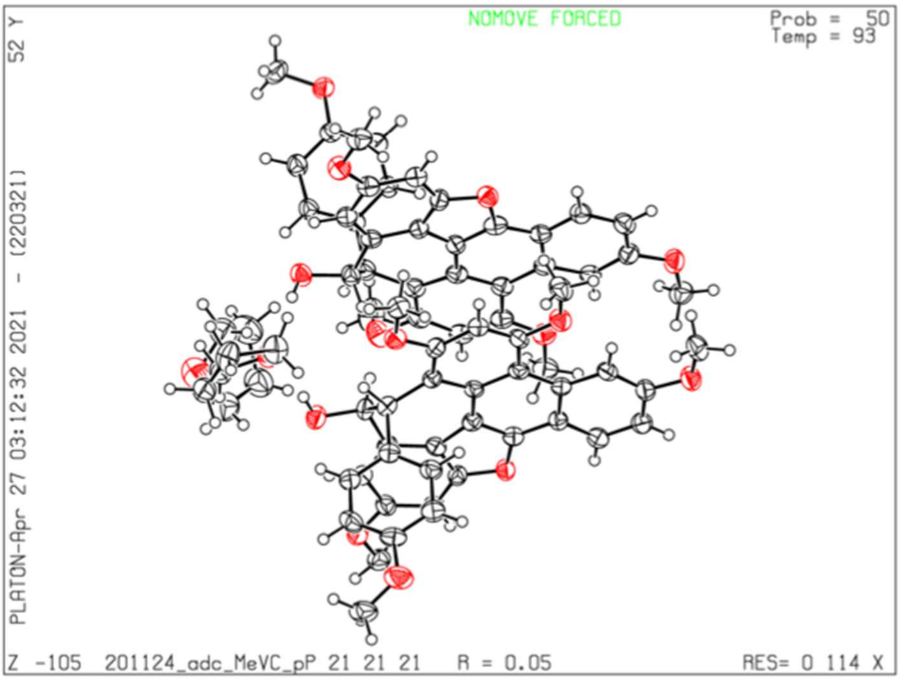

Supplementary Fig. 50. X-ray crystallographic analysis of (+)**-16**

Datablock 16minus_a - ellipsoid plot

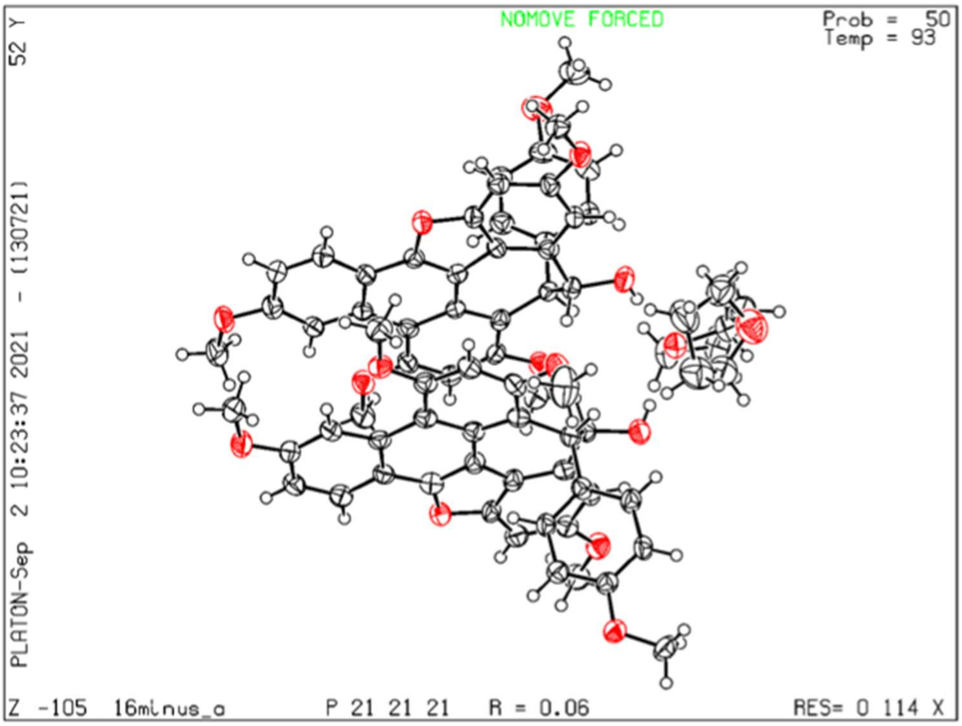

Supplementary Fig. 51. X-ray crystallographic analysis of (-)-**16**

II. Supplementary References

(1) Yao, C. S., Lin, M. & Wang Y. H. Synthesis of the Active Stilbenoids by Photooxidation Reaction of trans-ε-Viniferin. *Chin. J. Chem.* **22**, 1350-1355 (2004).

(2)Vo, Duc Duy and Elofsson, Mikael, Total synthesis of viniferifuran, resveratrol-piceatannol hybrid, angiopressin A and analogues. *Adv. Synth. Catal.* **358,** 4085–4492 (2016).

(3)Kim, I. & Choi, J. A versatile approach to oligostilbenoid natural sproducts – synthesis of permethylated analogues of viniferifuran, malibatol A, and shoreaphenol. *Org. Biomol. Chem.* **7,** 2788–2795 (2009).

(4) Dai, J. R., Hallock, Y. F., Cardellina, J. H. II & Boyd, M. R. HIV-inhibitory and cytotoxic oligostilbenes from the leaves of *Hopea malibato*. *J. Nat. Prod.* **61,** 351–353 (1998).

(5) Qin, Y. H. et al. Oligostilbenes from Vatica mangachapoi with xanthine oxidase and acetylcholinesterase inhibitory activities. *RSC Advances* **1**, 135–141 (2011).

(6) Abe, N. et al. Resveratrol derivatives from *Vatica albiramis*. *Chem. Pharm. Bull.* **59,** 452-457 (2011).

(7) Ge, H. M. et al. Hopeahinol A: An acetylcholinesterase inhibitor from *Hopea hainanensis*. *Chem. Eur. J.* **14,** 376–381 (2008).
