# Supplementary material for: Computation-guided asymmetric total syntheses of resveratrol dimers
Source: Nat Commun. 2022 Jan 10;13:152. doi: 10.1038/s41467-021-27546-4 (PMC8748746; doi:10.1038/s41467-021-27546-4)
Supplement: Supplementary file 3 — Source Data [file 41467_2021_27546_MOESM3_ESM.docx]

**Supplementary Data file**

**Computation-Guided Asymmetric Total Syntheses Resveratrol Dimers**

Masaya Nakajima*^1^, Yusuke Adachi^1^, Tetsuhiro Nemoto*^1^

^1^Graduate School of Pharmaceutical Sciences, Chiba University, 1-8-1, Inohana, Chuo-ku, Chiba 260-8675, Japan.

E-mail: m.nakajima@chiba-u.jp (MN), tnemoto@faculty.chiba-u.jp (TN)

**Cartesian Coordinates and Energies**

SM.log

Energy (E) = -1605.16768673 Hartree

Enthalpy (H) = -1604.711781 Hartree

Gibbs free energy (G) = -1604.799127 Hartree

Charge = 0, Spin = 1

C 3.067992 2.455425 1.979705

C 1.7968 2.986656 1.793844

C 0.81872 2.074337 1.428561

C 1.034602 0.708599 1.266519

C 2.331894 0.20218 1.418612

C 3.335591 1.087244 1.783646

H 1.597034 4.044483 1.913368

H 4.353471 0.730257 1.89743

C -0.241986 0.136518 0.891874

C -1.10267 1.192917 0.799738

O -0.478943 2.379045 1.14431

C -2.489729 1.335582 0.354772

C -3.358535 2.229363 0.992671

C -2.962219 0.601214 -0.739394

C -4.669901 2.373726 0.563868

H -3.006507 2.811712 1.837924

C -4.273814 0.7327 -1.17079

H -2.29256 -0.074715 -1.261782

C -5.132121 1.619172 -0.515837

H -5.345727 3.061675 1.060386

H -4.634354 0.160594 -2.020492

C -0.545817 -1.30099 0.749698

C -1.803964 -1.765228 1.150133

C 0.421715 -2.192424 0.253105

C -2.125282 -3.110322 1.018835

H -2.5331 -1.081544 1.571256

C 0.064554 -3.546906 0.14466

C -1.194565 -4.010961 0.511763

H -1.448108 -5.062037 0.415128

C 2.601028 -1.251931 1.121349

H 2.239967 -1.853365 1.967077

C 1.823378 -1.75874 -0.136175

H 2.35865 -2.669335 -0.420975

C 1.867152 -0.819574 -1.34146

C 3.03575 -0.11955 -1.667137

C 0.762361 -0.650645 -2.178438

C 3.092474 0.732673 -2.762455

H 3.91817 -0.236555 -1.048298

C 0.803041 0.195522 -3.283257

H -0.159908 -1.183526 -1.970083

C 1.970312 0.896922 -3.574233

H 4.003187 1.274642 -2.996381

H -0.072704 0.314033 -3.915642

O 4.000981 -1.435192 0.992018

O 2.07305 1.752576 -4.638428

O 1.001046 -4.413208 -0.344525

O -3.342845 -3.607816 1.385441

O -6.429869 1.789404 -0.89508

H 1.231928 1.775603 -5.110505

H 0.633661 -5.304894 -0.358029

H -3.901294 -2.8842 1.695111

H -6.624787 1.206553 -1.63921

H 4.162438 -2.384315 0.953394

O 4.060088 3.324165 2.341804

H 4.891905 2.842259 2.420956

SM2.log

Energy (E) = -1605.15621420 Hartree

Enthalpy (H) = -1604.700574 Hartree

Gibbs free energy (G) = -1604.788329 Hartree

Charge = 0, Spin = 1

C 0.067931 -4.69327 -0.025609

C 1.335287 -4.206472 0.279976

C 1.484676 -2.831569 0.19714

C 0.477202 -1.94806 -0.186485

C -0.809171 -2.449863 -0.425742

C -0.990767 -3.825589 -0.350747

H 2.141875 -4.86588 0.576713

H -1.981721 -4.24546 -0.507718

C 1.072106 -0.624887 -0.173313

C 2.350958 -0.80382 0.275646

O 2.621794 -2.141889 0.495553

C 3.45086 0.103456 0.601

C 4.781474 -0.289773 0.406803

C 3.196517 1.370591 1.139912

C 5.828618 0.560596 0.728755

H 4.998902 -1.269227 -0.005954

C 4.236606 2.230963 1.457464

H 2.173391 1.68445 1.318019

C 5.557935 1.826325 1.252068

H 6.858761 0.257577 0.575374

H 4.030728 3.211259 1.877105

C 0.466738 0.574329 -0.789303

C 1.301351 1.430282 -1.51799

C -0.929063 0.765098 -0.759448

C 0.758884 2.497472 -2.219499

H 2.369648 1.249053 -1.564696

C -1.44581 1.813588 -1.543991

C -0.6165 2.687896 -2.241889

H -1.043812 3.498154 -2.824654

C -2.004656 -1.537767 -0.631566

C -1.800651 -0.178459 0.071671

O -2.795225 1.971498 -1.631871

O 1.526688 3.370479 -2.936213

O 6.62023 2.625662 1.548824

H -2.986406 2.73651 -2.187892

H 2.45464 3.119866 -2.852177

H 6.303382 3.462928 1.909564

O -0.11335 -6.046435 0.043548

H -1.028782 -6.259234 -0.174566

C -3.08903 0.426037 0.614802

C -4.36007 0.073421 0.156101

C -3.004276 1.360576 1.64792

C -5.50763 0.630048 0.708333

H -4.46642 -0.628969 -0.66442

C -4.140977 1.933781 2.208723

H -2.026717 1.649923 2.025491

C -5.400201 1.564749 1.737222

H -6.490909 0.351071 0.343549

H -4.054314 2.657804 3.014187

O -2.29194 -1.301659 -2.006077

H -2.517247 -2.152296 -2.399938

H -2.859638 -2.040621 -0.162962

H -1.213433 -0.405034 0.967885

O -6.559386 2.085554 2.247752

H -6.342454 2.724355 2.937239

cpdI.log

Energy (E) = -1624.69460174 Hartree

Enthalpy (H) = -1624.351139 Hartree

Gibbs free energy (G) = -1624.423596 Hartree

Charge = 0, Spin = 2

C -0.362056 3.404304 0.218452

C -3.285969 -0.428341 0.373094

C 0.52768 -3.353313 0.185209

C 3.460656 0.472606 0.064708

C 2.666602 1.604343 0.138028

C 3.177353 2.948688 0.086926

C 2.103075 3.774693 0.128377

C 0.939238 2.932538 0.206169

N 1.298991 1.611233 0.226544

C -1.499052 2.610501 0.242755

C -2.844727 3.116387 0.255138

C -3.662279 2.034276 0.309279

C -2.811346 0.874768 0.325804

N -1.495314 1.24635 0.278185

C -2.506519 -1.575875 0.356466

C -3.015378 -2.923312 0.425034

C -1.938294 -3.745642 0.37368

C -0.775723 -2.899032 0.271052

N -1.147135 -1.581611 0.257828

C 1.662249 -2.555685 0.099597

C 3.000991 -3.074144 0.009621

C 3.833244 -2.001406 -0.024734

C 2.996203 -0.835147 0.047206

N 1.681389 -1.188843 0.114502

H -0.503794 4.479848 0.19499

H -4.361989 -0.558982 0.427723

H 0.680767 -4.427789 0.187326

H 4.533729 0.622041 0.00196

H 4.226652 3.201087 0.016571

H 2.074631 4.855414 0.099489

H -3.105586 4.16555 0.229386

H -4.74292 2.000035 0.336161

H -4.063045 -3.180299 0.503134

H -1.908338 -4.826512 0.400471

H 3.248883 -4.12652 -0.019338

H 4.91262 -1.979986 -0.088301

Fe 0.101378 0.053521 0.290099

S -0.019648 -0.282517 -2.002437

C -1.66305 0.152028 -2.611809

H -1.659032 0.015247 -3.696252

H -2.416706 -0.520285 -2.19009

H -1.925546 1.183526 -2.369395

O 0.038162 -0.197869 1.930357

CP1.log

Energy (E) = -3229.88447924 Hartree

Enthalpy (H) = -3229.081386 Hartree

Gibbs free energy (G) = -3229.219109 Hartree

Charge = 0, Spin = 2

C 0.872048 -3.55963 -2.446639

C 0.025019 -2.551317 -2.935971

C 0.312539 -1.28944 -2.495774

C 1.346781 -0.955808 -1.600698

C 2.215019 -1.984222 -1.154398

C 1.95477 -3.267267 -1.581582

H -0.799208 -2.775045 -3.600936

H 2.612001 -4.079628 -1.28283

C 1.217899 0.423367 -1.32543

C 0.145482 0.866951 -2.157434

O -0.376243 -0.162 -2.85142

C -0.46196 2.14344 -2.376973

C 0.273101 3.340363 -2.273156

C -1.841919 2.20394 -2.673129

C -0.349458 4.558842 -2.448549

H 1.338357 3.307057 -2.07455

C -2.471779 3.419828 -2.820982

H -2.418843 1.288026 -2.739617

C -1.726802 4.604722 -2.710153

H 0.216997 5.482423 -2.385421

H -3.537322 3.477108 -3.013287

C 1.842005 1.140116 -0.210444

C 1.077215 2.102818 0.465839

C 3.114167 0.745019 0.259681

C 1.583533 2.707133 1.608395

H 0.074735 2.349546 0.139828

C 3.539509 1.303537 1.476565

C 2.805481 2.297908 2.123167

H 3.175428 2.732499 3.046816

C 3.452479 -1.69878 -0.322678

C 3.945525 -0.257192 -0.539188

O 4.690933 0.858433 2.03946

O 0.912134 3.68199 2.278506

O -2.387531 5.76614 -2.867159

H 4.8432 1.331113 2.866457

H 0.039415 3.800472 1.882019

H -1.779092 6.510702 -2.770484

O 0.625714 -4.806641 -2.864044

H 1.241756 -5.431712 -2.458483

C -0.086633 -2.89159 1.612119

C -3.788244 -2.640603 -1.47511

C -4.667049 1.875501 -0.013257

C -1.141967 1.513341 3.265305

C -0.527281 0.290123 3.069296

C 0.649005 -0.147694 3.782912

C 0.959966 -1.375827 3.302025

C -0.032612 -1.692033 2.301726

N -0.91821 -0.663067 2.175211

C -1.049773 -3.222451 0.671547

C -1.098938 -4.488139 -0.017995

C -2.141356 -4.420282 -0.883104

C -2.725957 -3.112093 -0.723439

N -2.060198 -2.412927 0.242949

C -4.347734 -1.37821 -1.345325

C -5.40647 -0.869981 -2.182777

C -5.645642 0.402632 -1.777091

C -4.73825 0.666763 -0.687377

N -3.97045 -0.433345 -0.437146

C -3.792904 2.158584 1.023895

C -3.744287 3.420706 1.719027

C -2.767872 3.311492 2.655949

C -2.21309 1.986963 2.521564

N -2.856997 1.305012 1.533615

H 0.678592 -3.630624 1.82671

H -4.202735 -3.30028 -2.230267

H -5.347262 2.662127 -0.323228

H -0.720162 2.173863 4.014545

H 1.156306 0.433109 4.541809

H 1.776986 -2.027078 3.581557

H -0.401259 -5.299792 0.140624

H -2.487436 -5.164886 -1.587456

H -5.883293 -1.429892 -2.976418

H -6.362715 1.114551 -2.164178

H -4.384123 4.265693 1.501465

H -2.429017 4.047877 3.3728

Fe -2.416933 -0.531343 0.837866

S -3.920749 -1.362784 2.577461

C -4.746127 -2.842159 1.904132

H -5.44719 -3.233027 2.647934

H -5.308997 -2.605657 0.996412

H -4.026851 -3.631157 1.664995

O -1.431381 0.093398 -0.302054

C 5.454292 -0.094262 -0.414368

C 6.051722 1.021749 -1.002731

C 6.280554 -1.015491 0.232268

C 7.426484 1.225235 -0.948402

H 5.42996 1.752064 -1.514672

C 7.657571 -0.832427 0.289843

H 5.850457 -1.88152 0.725369

C 8.234957 0.291858 -0.29975

H 7.873348 2.099718 -1.413259

H 8.29235 -1.552163 0.796701

O 9.593706 0.428737 -0.213544

H 9.860457 1.245449 -0.652184

H 3.742591 -0.039998 -1.593479

H 4.227163 -2.38745 -0.682133

O 3.22833 -1.904462 1.062027

H 2.935231 -2.814708 1.18826

TS1.log

Energy (E) = -3229.88952188 Hartree

Enthalpy (H) = -3229.079240 Hartree

Gibbs free energy (G) = -3229.215233 Hartree

Charge = 0, Spin = 2

Imaginary frequency: -239.0931

C 1.116057 -3.823022 -2.233471

C 0.152668 -2.909788 -2.677774

C 0.293623 -1.632834 -2.204861

C 1.314705 -1.190571 -1.344655

C 2.304874 -2.121654 -0.951123

C 2.18041 -3.42332 -1.397112

H -0.661325 -3.210302 -3.32469

H 2.934211 -4.158128 -1.126744

C 1.063318 0.180112 -1.077804

C -0.130284 0.490481 -1.824995

O -0.540801 -0.597561 -2.521844

C -0.646257 1.755183 -2.337999

C 0.168588 2.885124 -2.459999

C -1.987194 1.826678 -2.752428

C -0.344002 4.068658 -2.970403

H 1.211694 2.842246 -2.167274

C -2.507167 3.004784 -3.25084

H -2.626871 0.954923 -2.660052

C -1.68389 4.133354 -3.361029

H 0.291142 4.943309 -3.071471

H -3.546073 3.069886 -3.555454

C 1.716183 1.022511 -0.078564

C 0.968018 2.047953 0.519946

C 3.033324 0.732243 0.352152

C 1.504988 2.77694 1.570711

H -0.05202 2.235778 0.21086

C 3.491308 1.416179 1.489815

C 2.75673 2.449253 2.072516

H 3.152434 2.978479 2.934035

C 3.525567 -1.716457 -0.150134

C 3.897711 -0.251765 -0.435684

O 4.679362 1.055452 2.040831

O 0.83096 3.796502 2.172291

O -2.240531 5.263579 -3.85958

H 4.859341 1.618241 2.8031

H -0.064457 3.843369 1.813113

H -1.577091 5.964028 -3.90115

O 0.986338 -5.095056 -2.654845

H 1.700184 -5.640129 -2.299552

C -0.019299 -2.69778 1.727579

C -3.605403 -2.564087 -1.498974

C -4.767809 1.872947 0.004368

C -1.24413 1.692321 3.296078

C -0.569983 0.502125 3.101683

C 0.616543 0.121246 3.828915

C 0.976541 -1.102697 3.373126

C 0.004439 -1.473983 2.373269

N -0.914299 -0.478389 2.215727

C -0.962655 -3.068451 0.783799

C -1.013399 -4.368617 0.166441

C -2.018707 -4.330658 -0.742818

C -2.582322 -3.007383 -0.681957

N -1.942855 -2.265332 0.273693

C -4.202949 -1.316536 -1.399694

C -5.25082 -0.843979 -2.268813

C -5.581908 0.399762 -1.837557

C -4.7365 0.684526 -0.705714

N -3.911462 -0.375289 -0.457517

C -3.936409 2.18699 1.066614

C -3.973895 3.438207 1.780549

C -2.987951 3.38497 2.712299

C -2.346836 2.103028 2.561363

N -2.947024 1.390562 1.567979

H 0.746704 -3.421263 1.98544

H -3.97514 -3.243394 -2.259595

H -5.492174 2.620094 -0.302647

H -0.857725 2.372917 4.046477

H 1.094174 0.733213 4.582402

H 1.812728 -1.719758 3.671973

H -0.33902 -5.18204 0.398291

H -2.350808 -5.106441 -1.419539

H -5.664786 -1.408824 -3.093454

H -6.325581 1.07925 -2.232357

H -4.669601 4.240693 1.574277

H -2.6971 4.133776 3.437044

Fe -2.380735 -0.40643 0.846222

S -3.874334 -1.272777 2.522503

C -4.587512 -2.812422 1.866145

H -5.307192 -3.192861 2.59728

H -5.115133 -2.637187 0.924954

H -3.823894 -3.577862 1.704207

O -1.427659 0.384027 -0.262818

C 5.394965 0.023255 -0.395509

C 5.887117 1.125259 -1.096616

C 6.311282 -0.788094 0.276888

C 7.246154 1.420286 -1.128647

H 5.193984 1.769795 -1.631666

C 7.673794 -0.513468 0.249508

H 5.963145 -1.638378 0.855111

C 8.145128 0.594774 -0.454245

H 7.611332 2.281482 -1.681484

H 8.37914 -1.148512 0.775915

O 9.494077 0.825212 -0.451929

H 9.681063 1.615451 -0.972651

H 3.6314 -0.092188 -1.486212

H 4.347293 -2.356267 -0.494859

O 3.342333 -1.884365 1.247056

H 3.100824 -2.804452 1.40601

INT1.log

Energy (E) = -3229.96440011 Hartree

Enthalpy (H) = -3229.161240 Hartree

Gibbs free energy (G) = -3229.297267 Hartree

Charge = 0, Spin = 2

C 0.948888 -3.751659 -2.291556

C 0.025252 -2.804443 -2.70518

C 0.082141 -1.571341 -2.077711

C 1.092993 -1.238719 -1.101343

C 2.079709 -2.229397 -0.759324

C 1.984409 -3.454061 -1.338979

H -0.745949 -3.046056 -3.424565

H 2.716561 -4.225492 -1.118157

C 0.852865 0.037751 -0.667574

C -0.435594 0.569443 -1.342406

O -0.762267 -0.604427 -2.303214

C -0.132655 1.741238 -2.274317

C 0.978038 1.734864 -3.119608

C -0.977184 2.849358 -2.274668

C 1.24308 2.813742 -3.952998

H 1.651623 0.881601 -3.13349

C -0.724462 3.935426 -3.103847

H -1.837063 2.85864 -1.612542

C 0.388649 3.919225 -3.945373

H 2.110002 2.805868 -4.607789

H -1.379574 4.800465 -3.105519

C 1.586141 0.860962 0.282478

C 0.902835 1.963693 0.817059

C 2.914565 0.554459 0.680351

C 1.493864 2.737121 1.803693

H -0.112653 2.17395 0.507726

C 3.434257 1.301911 1.749604

C 2.745942 2.390945 2.287624

H 3.188997 2.960669 3.099082

C 3.245647 -1.91307 0.153635

C 3.729788 -0.476758 -0.100024

O 4.633975 0.956507 2.284363

O 0.873904 3.816462 2.354776

O 0.598634 5.010399 -4.737039

H 4.857836 1.576681 2.988194

H -0.018956 3.890701 1.992665

H 1.394045 4.877318 -5.266842

O 0.859371 -4.965181 -2.824483

H 1.53591 -5.558765 -2.469693

C -0.333504 -2.490158 1.864299

C -3.653867 -2.498545 -1.633897

C -4.894308 1.99934 -0.381718

C -1.617778 1.976008 3.159406

C -0.951137 0.767188 3.072045

C 0.150887 0.389869 3.922855

C 0.528878 -0.854183 3.540388

C -0.351886 -1.242056 2.464148

N -1.226937 -0.232463 2.181468

C -1.176155 -2.893902 0.841186

C -1.176939 -4.221795 0.278413

C -2.105956 -4.22497 -0.709226

C -2.671208 -2.899592 -0.747935

N -2.097369 -2.108 0.210372

C -4.262393 -1.251036 -1.613897

C -5.289346 -0.831233 -2.533194

C -5.639848 0.43011 -2.173348

C -4.82721 0.777543 -1.03433

N -3.998836 -0.260939 -0.71308

C -4.122716 2.375873 0.707211

C -4.210557 3.661675 1.355936

C -3.294698 3.655219 2.358029

C -2.646263 2.367725 2.313389

N -3.16756 1.608821 1.309671

H 0.388718 -3.211713 2.230785

H -3.989806 -3.213841 -2.377263

H -5.608756 2.723967 -0.759277

H -1.293617 2.67747 3.920581

H 0.56937 1.018605 4.697537

H 1.322339 -1.4737 3.935978

H -0.528889 -5.025255 0.602763

H -2.386219 -5.031071 -1.37407

H -5.676252 -1.43828 -3.340933

H -6.376614 1.082645 -2.623153

H -4.889549 4.452055 1.064065

H -3.058774 4.438235 3.06659

Fe -2.642483 -0.256006 0.761242

S -4.154564 -1.031105 2.261154

C -4.933068 -2.520231 1.571779

H -5.700982 -2.849886 2.277472

H -5.411036 -2.311194 0.611837

H -4.211277 -3.330071 1.440193

O -1.447118 0.772783 -0.542027

C 5.241652 -0.328154 -0.025652

C 5.850319 0.691302 -0.758247

C 6.05949 -1.182153 0.717677

C 7.230178 0.86881 -0.748886

H 5.234408 1.364633 -1.34929

C 7.439872 -1.024711 0.732965

H 5.618113 -1.97158 1.31848

C 8.029584 0.005905 7.8e-05

H 7.687751 1.667506 -1.326136

H 8.068843 -1.690192 1.31547

O 9.391743 0.12258 0.050307

H 9.667993 0.871127 -0.491993

H 3.495818 -0.277741 -1.153046

H 4.04794 -2.607061 -0.120999

O 2.927184 -2.061717 1.525514

H 2.691632 -2.98483 1.675949

CP1'.log

Energy (E) = -3229.89806249 Hartree

Enthalpy (H) = -3229.094598 Hartree

Gibbs free energy (G) = -3229.232851 Hartree

Charge = 0, Spin = 2

C -1.936002 3.63308 1.518386

C -1.328742 2.63611 2.302794

C -1.333659 1.388255 1.747861

C -1.867596 1.069376 0.483983

C -2.50227 2.08829 -0.272074

C -2.530214 3.353518 0.262654

H -0.888128 2.860881 3.265387

H -3.013286 4.163009 -0.272229

C -1.638911 -0.300858 0.276943

C -1.059092 -0.768799 1.488861

O -0.847738 0.257627 2.342311

C -0.75557 -2.074994 1.987528

C -1.468845 -3.20981 1.550809

C 0.246348 -2.229134 2.970038

C -1.176417 -4.45847 2.0594

H -2.274841 -3.102285 0.834026

C 0.553591 -3.476759 3.466052

H 0.799139 -1.362038 3.313484

C -0.154661 -4.599763 3.009825

H -1.734747 -5.331719 1.736977

H 1.337624 -3.609105 4.203004

C -1.8384 -1.061847 -0.958823

C -0.92036 -2.070348 -1.27372

C -2.910367 -0.754435 -1.818669

C -1.087902 -2.817572 -2.433167

H -0.059723 -2.251582 -0.640444

C -3.048132 -1.532191 -2.977361

C -2.156339 -2.558717 -3.283353

H -2.280235 -3.142622 -4.190116

C -3.14233 1.740597 -1.586587

H -2.331828 1.627551 -2.320411

C -3.889331 0.372178 -1.546869

H -4.5565 0.416111 -2.411868

C -4.76155 0.152011 -0.311456

C -5.526442 1.199349 0.217678

C -4.851929 -1.094106 0.312147

C -6.332838 1.017191 1.33384

H -5.490152 2.177504 -0.249163

C -5.658108 -1.294203 1.429664

H -4.284909 -1.936224 -0.072

C -6.40062 -0.234809 1.946086

H -6.91682 1.838816 1.735991

H -5.709556 -2.27269 1.899243

O -3.981097 2.809797 -1.9739

O -7.210289 -0.366125 3.040202

O -4.091871 -1.253409 -3.807529

O -0.22914 -3.811269 -2.788419

O 0.186534 -5.793544 3.528609

H -7.177169 -1.278308 3.352307

H -4.072639 -1.849304 -4.565997

H 0.508773 -3.837987 -2.165318

H -0.358117 -6.493528 3.145445

H -4.248857 2.650055 -2.885645

O -1.936425 4.871721 2.02025

H -2.362297 5.484851 1.405525

C 0.433319 2.666021 -1.758005

C 3.112959 2.620728 2.257969

C 4.89989 -1.681414 0.990733

C 2.135796 -1.680401 -2.967803

C 1.410128 -0.502064 -2.960043

C 0.463035 -0.131438 -3.983623

C -0.022638 1.086448 -3.639804

C 0.630053 1.45908 -2.406347

N 1.486736 0.474203 -2.010923

C 1.056823 3.043112 -0.578442

C 0.834745 4.305963 0.080133

C 1.566837 4.284365 1.222118

C 2.245922 3.013267 1.252734

N 1.929425 2.282519 0.14257

C 3.834055 1.436657 2.258699

C 4.760386 1.051009 3.294481

C 5.271795 -0.151831 2.930418

C 4.653763 -0.501538 1.675025

N 3.792602 0.482223 1.284966

C 4.294904 -2.052978 -0.198669

C 4.55395 -3.295586 -0.883818

C 3.77384 -3.298015 -1.994189

C 3.040274 -2.056158 -1.986359

N 3.372378 -1.319887 -0.888341

H -0.268418 3.365583 -2.200308

H 3.257703 3.299414 3.09194

H 5.614402 -2.372539 1.425917

H 1.971058 -2.367795 -3.790097

H 0.217673 -0.743355 -4.841378

H -0.747843 1.699547 -4.158913

H 0.180328 5.083726 -0.29059

H 1.650681 5.043464 1.988367

H 4.976252 1.641365 4.175175

H 5.997238 -0.765245 3.448372

H 5.245915 -4.052747 -0.539351

H 3.686717 -4.056163 -2.761323

Fe 2.585085 0.444122 -0.321965

S 4.392857 1.449959 -1.63084

C 4.909952 2.958007 -0.747483

H 5.741419 3.422368 -1.286521

H 5.247208 2.729256 0.267741

H 4.096193 3.686561 -0.682362

O 1.407929 -0.297675 0.54355

TS1'.log

Energy (E) = -3229.89343560 Hartree

Enthalpy (H) = -3229.092201 Hartree

Gibbs free energy (G) = -3229.229075 Hartree

Charge = 0, Spin = 2

Imaginary frequency: -200.5374

C -2.189198 3.571652 1.505334

C -1.410926 2.655493 2.222835

C -1.259996 1.430801 1.62741

C -1.836969 1.050526 0.405053

C -2.653797 1.980966 -0.276704

C -2.810712 3.231524 0.283164

H -0.939171 2.918836 3.160247

H -3.432498 3.971849 -0.207699

C -1.424291 -0.276454 0.146623

C -0.596708 -0.632965 1.26589

O -0.541797 0.391038 2.152404

C -0.235617 -1.932824 1.819539

C -1.012531 -3.069151 1.57336

C 0.895657 -2.036827 2.645214

C -0.659635 -4.292129 2.125224

H -1.905591 -2.998869 0.961893

C 1.260894 -3.253716 3.186463

H 1.501232 -1.157679 2.840115

C 0.481779 -4.388727 2.92543

H -1.265774 -5.173677 1.94059

H 2.145965 -3.343409 3.807005

C -1.655122 -1.077146 -1.053909

C -0.6945 -2.029589 -1.422013

C -2.813455 -0.879592 -1.835602

C -0.876809 -2.792674 -2.568031

H 0.21601 -2.138421 -0.845697

C -2.963689 -1.673881 -2.981324

C -2.011351 -2.621003 -3.352102

H -2.147546 -3.217731 -4.248762

C -3.342077 1.568175 -1.547911

H -2.586916 1.570701 -2.347095

C -3.899455 0.11643 -1.475193

H -4.632461 0.077842 -2.285562

C -4.635553 -0.231187 -0.180731

C -5.444943 0.711857 0.46519

C -4.550837 -1.504785 0.386321

C -6.117504 0.407572 1.642257

H -5.549693 1.706092 0.045377

C -5.221901 -1.82739 1.562621

H -3.94811 -2.271699 -0.089811

C -6.003186 -0.866287 2.199659

H -6.734008 1.151838 2.136154

H -5.134767 -2.823801 1.987319

O -4.345138 2.520093 -1.84673

O -6.676673 -1.118357 3.362995

O -4.086789 -1.495691 -3.734395

O 0.029207 -3.724441 -2.973702

O 0.881001 -5.558367 3.482117

H -6.517286 -2.031949 3.628918

H -4.071311 -2.099242 -4.486537

H 0.811333 -3.685354 -2.407086

H 0.263348 -6.261177 3.244047

H -4.679129 2.319905 -2.72804

O -2.324707 4.801512 2.038802

H -2.877322 5.351857 1.468735

C 0.649267 3.514501 -0.695042

C 3.250055 1.440172 2.802441

C 4.375055 -2.156084 -0.209864

C 1.35835 -0.337397 -3.508609

C 0.853898 0.840441 -2.989821

C -0.069285 1.698482 -3.688865

C -0.24562 2.796597 -2.911703

C 0.555063 2.599423 -1.72959

N 1.202896 1.400761 -1.792316

C 1.350703 3.300188 0.477894

C 1.368735 4.222812 1.584239

C 2.052213 3.616702 2.586575

C 2.477767 2.335139 2.083401

N 2.045022 2.168162 0.797128

C 3.78433 0.271477 2.282027

C 4.604781 -0.652619 3.025962

C 4.938761 -1.650455 2.169594

C 4.313986 -1.339644 0.907046

N 3.632067 -0.161723 0.998833

C 3.68257 -1.942951 -1.390962

C 3.695387 -2.854832 -2.507081

C 2.84035 -2.349447 -3.433264

C 2.306629 -1.130274 -2.879901

N 2.845372 -0.896093 -1.650698

H 0.100729 4.445166 -0.791893

H 3.481867 1.687554 3.832814

H 4.977031 -3.056104 -0.139458

H 0.996283 -0.658104 -4.47954

H -0.502244 1.473733 -4.654551

H -0.862279 3.666467 -3.094962

H 0.879231 5.187509 1.582668

H 2.265659 3.984295 3.581423

H 4.876972 -0.530814 4.065922

H 5.542931 -2.528747 2.35439

H 4.283979 -3.761547 -2.550728

H 2.571856 -2.752443 -4.400446

Fe 2.371855 0.593417 -0.378082

S 4.242672 1.741303 -1.360479

C 5.000155 2.723759 -0.028942

H 5.906444 3.194265 -0.421835

H 5.280996 2.092286 0.818072

H 4.330095 3.511184 0.326888

O 1.185066 -0.365289 0.279344

INT1'.log

Energy (E) = -3229.98266895 Hartree

Enthalpy (H) = -3229.179342 Hartree

Gibbs free energy (G) = -3229.314288 Hartree

Charge = 0, Spin = 2

C -2.028386 3.770372 1.071773

C -1.231911 2.957775 1.869418

C -0.92836 1.707836 1.36227

C -1.464116 1.230516 0.111472

C -2.2777 2.109285 -0.680784

C -2.544488 3.351097 -0.199959

H -0.833201 3.310628 2.811314

H -3.15075 4.048073 -0.766775

C -1.029516 -0.050957 -0.076156

C -0.073696 -0.433264 1.081104

O -0.156883 0.839858 1.958647

C -0.683541 -1.52364 1.964901

C -1.946906 -1.376889 2.540107

C 0.023645 -2.704239 2.182959

C -2.497726 -2.387684 3.317396

H -2.521 -0.468375 2.382816

C -0.513763 -3.723458 2.959707

H 1.005123 -2.823931 1.735967

C -1.778501 -3.566774 3.527437

H -3.481151 -2.265179 3.762616

H 0.035852 -4.64323 3.130947

C -1.376658 -1.029335 -1.100401

C -0.557538 -2.163851 -1.200916

C -2.530909 -0.904149 -1.910963

C -0.849892 -3.163481 -2.116749

H 0.336085 -2.238104 -0.596515

C -2.817483 -1.959514 -2.788554

C -1.984865 -3.069392 -2.911257

H -2.221247 -3.858136 -3.619073

C -2.786373 1.61234 -1.997133

H -1.910854 1.438141 -2.640315

C -3.511173 0.248645 -1.846873

H -4.117876 0.179035 -2.75383

C -4.468203 0.170872 -0.657051

C -5.414067 1.184554 -0.449439

C -4.455077 -0.896099 0.241301

C -6.297112 1.144753 0.620821

H -5.452154 2.025225 -1.133546

C -5.337389 -0.95292 1.318013

H -3.742929 -1.705524 0.118839

C -6.260387 0.070991 1.512751

H -7.021301 1.938688 0.772667

H -5.303067 -1.792376 2.007467

O -3.609926 2.604987 -2.570474

O -7.150034 0.075055 2.550886

O -3.955708 -1.870831 -3.533359

O -0.055549 -4.25738 -2.274708

O -2.266795 -4.59521 4.279024

H -7.022954 -0.719411 3.083521

H -4.034758 -2.65133 -4.094207

H 0.727784 -4.166732 -1.716265

H -3.143023 -4.363011 4.609022

H -3.854155 2.303678 -3.452338

O -2.298047 4.988622 1.529561

H -2.859165 5.480424 0.913816

C 0.767079 2.254823 -2.286276

C 2.746378 2.811098 2.077247

C 4.573573 -1.648744 1.754712

C 2.504455 -2.237756 -2.56412

C 1.812616 -1.070449 -2.835478

C 1.024757 -0.857588 -4.02443

C 0.527908 0.401062 -3.940291

C 1.014956 0.956558 -2.700034

N 1.774999 0.036722 -2.035272

C 1.204799 2.806126 -1.092516

C 0.944117 4.167417 -0.693412

C 1.484642 4.321118 0.540414

C 2.080114 3.055598 0.890686

N 1.901934 2.149457 -0.119307

C 3.406194 1.626781 2.375148

C 4.124689 1.382472 3.600241

C 4.64901 0.134373 3.499957

C 4.247204 -0.382347 2.215065

N 3.49552 0.544488 1.549511

C 4.177739 -2.19178 0.54175

C 4.506134 -3.528348 0.108055

C 3.923606 -3.697631 -1.106308

C 3.240872 -2.463889 -1.409884

N 3.411345 -1.566153 -0.398785

H 0.175041 2.88512 -2.941993

H 2.783192 3.609018 2.81157

H 5.178556 -2.272369 2.405174

H 2.446774 -3.039699 -3.292608

H 0.883575 -1.592953 -4.805353

H -0.106822 0.928793 -4.640038

H 0.399364 4.887164 -1.289927

H 1.482671 5.195298 1.177748

H 4.205013 2.08823 4.41643

H 5.251135 -0.406755 4.21798

H 5.101585 -4.229951 0.677387

H 3.938054 -4.56672 -1.750756

Fe 2.66954 0.301287 -0.258726

S 4.540432 1.047444 -1.295221

C 4.995348 2.643998 -0.557859

H 5.92867 2.972108 -1.024358

H 5.15892 2.552907 0.518561

H 4.230779 3.403893 -0.737612

O 1.163202 -0.660663 0.729411

CP1_04.log

Energy (E) = -3229.91154017 Hartree

Enthalpy (H) = -3229.108434 Hartree

Gibbs free energy (G) = -3229.248655 Hartree

Charge = 0, Spin = 4

C -1.92372 3.693126 1.588002

C -1.407785 2.671397 2.377587

C -1.452916 1.405681 1.814237

C -1.94537 1.126813 0.541188

C -2.509326 2.162301 -0.214327

C -2.486165 3.441566 0.323191

H -1.000551 2.8618 3.36314

H -2.911256 4.270729 -0.231996

C -1.774798 -0.295879 0.344455

C -1.274628 -0.770785 1.522456

O -1.05575 0.253412 2.423431

C -0.969402 -2.111988 2.018837

C -1.79997 -3.191368 1.695509

C 0.147259 -2.340871 2.833152

C -1.510803 -4.471536 2.145398

H -2.684119 -3.024467 1.087924

C 0.441162 -3.616466 3.293293

H 0.797958 -1.514406 3.098927

C -0.384321 -4.687344 2.943308

H -2.159786 -5.304473 1.890627

H 1.30908 -3.796369 3.918889

C -1.945656 -1.018949 -0.9284

C -1.049052 -2.046562 -1.240413

C -2.956981 -0.648559 -1.83228

C -1.179696 -2.74562 -2.433533

H -0.233206 -2.285968 -0.566723

C -3.053896 -1.371928 -3.032363

C -2.189301 -2.420967 -3.33346

H -2.28109 -2.964226 -4.268802

C -3.136988 1.855995 -1.55095

H -2.33103 1.750178 -2.288906

C -3.912587 0.501202 -1.566427

H -4.552951 0.578981 -2.449815

C -4.834874 0.271859 -0.369488

C -5.555522 1.329482 0.199686

C -5.025445 -1.001467 0.17199

C -6.412762 1.130529 1.274921

H -5.443753 2.329677 -0.203661

C -5.883816 -1.219547 1.246526

H -4.493781 -1.850703 -0.245723

C -6.578912 -0.149193 1.804598

H -6.960157 1.961347 1.708742

H -6.011201 -2.219769 1.651786

O -3.959561 2.948883 -1.925593

O -7.435599 -0.29628 2.861875

O -4.038299 -1.015298 -3.911

O -0.327789 -3.752745 -2.782964

O -0.04902 -5.921581 3.413105

H -7.465212 -1.22558 3.118612

H -3.997277 -1.584296 -4.68863

H 0.361849 -3.834711 -2.111814

H -0.690122 -6.571096 3.099529

H -4.230402 2.802651 -2.838458

O -1.869586 4.961611 2.094652

H -2.246583 5.575247 1.452926

C 0.467453 2.600105 -1.646663

C 3.468761 2.646201 2.126669

C 4.945193 -1.806733 1.009678

C 2.15987 -1.729016 -2.92572

C 1.451976 -0.537863 -2.907894

C 0.492509 -0.16414 -3.90846

C -0.005795 1.042408 -3.535115

C 0.662087 1.401471 -2.314356

N 1.546534 0.427536 -1.946412

C 1.144746 2.983463 -0.506959

C 0.966648 4.251702 0.146054

C 1.807529 4.263594 1.21046

C 2.497397 3.003009 1.202565

N 2.089478 2.236405 0.149708

C 4.157581 1.450624 2.118756

C 5.139037 1.076112 3.102022

C 5.542536 -0.18067 2.79658

C 4.819248 -0.578186 1.618585

N 3.988601 0.439863 1.203913

C 4.286857 -2.178413 -0.15355

C 4.480013 -3.437262 -0.82053

C 3.712964 -3.406088 -1.939751

C 3.04387 -2.133711 -1.945863

N 3.406209 -1.398142 -0.8463

H -0.263389 3.290158 -2.054617

H 3.699072 3.355126 2.914412

H 5.626467 -2.523591 1.454958

H 1.975562 -2.409409 -3.749467

H 0.241287 -0.767112 -4.770138

H -0.746619 1.654583 -4.03167

H 0.273382 5.014876 -0.180077

H 1.961579 5.040774 1.946456

H 5.452752 1.710039 3.920281

H 6.263196 -0.804977 3.307237

H 5.132068 -4.223572 -0.46506

H 3.594592 -4.161789 -2.704535

Fe 2.635187 0.358155 -0.263555

S 4.743561 1.269564 -1.440495

C 4.914626 3.006373 -1.005357

H 5.761805 3.425588 -1.554089

H 5.134664 3.090729 0.065972

H 4.00294 3.56638 -1.222567

O 1.479807 -0.315557 0.633153

TS1_04.log

Energy (E) = -3229.88755204 Hartree

Enthalpy (H) = -3229.086771 Hartree

Gibbs free energy (G) = -3229.224560 Hartree

Charge = 0, Spin = 4

Imaginary frequency: -605.3662

C -2.109285 3.673252 1.550222

C -1.35184 2.737202 2.249262

C -1.214431 1.50838 1.636681

C -1.78183 1.163268 0.405285

C -2.563548 2.11816 -0.266496

C -2.71324 3.365545 0.316872

H -0.883371 2.974532 3.196228

H -3.312993 4.123939 -0.174357

C -1.399455 -0.192118 0.142112

C -0.597384 -0.578579 1.238907

O -0.527048 0.4553 2.156886

C -0.427637 -1.908462 1.858587

C -1.393782 -2.901607 1.683689

C 0.667823 -2.17185 2.69199

C -1.262114 -4.140539 2.298739

H -2.265272 -2.706522 1.068186

C 0.805485 -3.401936 3.315368

H 1.423228 -1.408277 2.84385

C -0.158185 -4.3945 3.113727

H -2.018288 -4.907111 2.15617

H 1.65529 -3.608501 3.95739

C -1.672753 -0.987926 -1.058766

C -0.781487 -2.016221 -1.392911

C -2.79508 -0.725796 -1.873885

C -0.984299 -2.778263 -2.534874

H 0.101818 -2.18247 -0.788361

C -2.965468 -1.517092 -3.020233

C -2.07603 -2.534361 -3.359306

H -2.227885 -3.127146 -4.255983

C -3.224451 1.75254 -1.567962

H -2.450395 1.740834 -2.348487

C -3.840167 0.323122 -1.541645

H -4.544842 0.326672 -2.377875

C -4.64146 -0.010874 -0.281943

C -5.410281 0.96018 0.372403

C -4.662628 -1.306052 0.240415

C -6.13818 0.661786 1.517951

H -5.438543 1.972222 -0.015107

C -5.389033 -1.62342 1.384951

H -4.098809 -2.094343 -0.248364

C -6.123098 -0.633991 2.034423

H -6.720049 1.428474 2.019622

H -5.381382 -2.637447 1.77572

O -4.187009 2.743506 -1.883962

O -6.844425 -0.879562 3.170939

O -4.052703 -1.266357 -3.809787

O -0.124988 -3.771774 -2.905372

O 0.026904 -5.587892 3.744134

H -6.738308 -1.806227 3.41761

H -4.058088 -1.880586 -4.553159

H 0.622944 -3.790256 -2.294401

H -0.69939 -6.185164 3.526905

H -4.516408 2.552794 -2.768883

O -2.23716 4.908288 2.108917

H -2.779184 5.465956 1.537721

C 0.370379 2.765404 -1.729124

C 2.949731 2.47353 2.332093

C 4.681913 -1.814931 0.950774

C 2.151372 -1.491714 -3.138801

C 1.405948 -0.321157 -3.078951

C 0.474516 0.085235 -4.094745

C -0.042553 1.276098 -3.692131

C 0.579637 1.587915 -2.434638

N 1.449835 0.600053 -2.072096

C 0.956799 3.072684 -0.516814

C 0.794665 4.329199 0.166273

C 1.50288 4.237473 1.319147

C 2.098658 2.92712 1.330564

N 1.763898 2.233143 0.206754

C 3.64918 1.282569 2.300236

C 4.514404 0.819585 3.355555

C 4.998897 -0.384903 2.967282

C 4.440199 -0.663392 1.669097

N 3.628581 0.375625 1.270164

C 4.14868 -2.096431 -0.302346

C 4.429136 -3.292049 -1.055916

C 3.726072 -3.194023 -2.213024

C 3.013363 -1.942889 -2.156484

N 3.29162 -1.289629 -0.986049

H -0.302028 3.497789 -2.163847

H 3.092867 3.114625 3.195555

H 5.34285 -2.552812 1.393264

H 2.016747 -2.120972 -4.012129

H 0.256393 -0.481812 -4.989663

H -0.769826 1.903715 -4.18981

H 0.197875 5.153477 -0.200164

H 1.622296 4.972484 2.10354

H 4.703688 1.362678 4.271787

H 5.67423 -1.045225 3.494674

H 5.0844 -4.086759 -0.725325

H 3.676943 -3.890906 -3.039115

Fe 2.423065 0.411152 -0.329948

S 4.587546 1.465351 -1.190037

C 4.672414 3.16538 -0.607109

H 5.557735 3.644538 -1.032497

H 4.781798 3.165394 0.484356

H 3.772956 3.726545 -0.868915

O 1.247471 -0.569929 0.436332

INT1_04.log

Energy (E) = -3229.94859292 Hartree

Enthalpy (H) = -3229.149015 Hartree

Gibbs free energy (G) = -3229.284851 Hartree

Charge = 0, Spin = 4

C -2.095616 3.78927 1.134122

C -1.24289 2.965416 1.87864

C -0.955884 1.727394 1.345365

C -1.521902 1.254085 0.138336

C -2.351363 2.117211 -0.602003

C -2.634707 3.377775 -0.093389

H -0.803538 3.30842 2.807734

H -3.275247 4.057256 -0.644906

C -1.045299 -0.055665 -0.081765

C -0.052534 -0.381751 1.047827

O -0.118206 0.838151 1.908131

C -0.567231 -1.518128 1.943228

C -1.830676 -1.429239 2.531386

C 0.195738 -2.661287 2.172475

C -2.328993 -2.457051 3.322239

H -2.4461 -0.549456 2.36655

C -0.287781 -3.699217 2.96274

H 1.178791 -2.741942 1.720336

C -1.554593 -3.599916 3.536345

H -3.314892 -2.37768 3.772579

H 0.306256 -4.590644 3.137048

C -1.400683 -1.02338 -1.091605

C -0.590311 -2.171486 -1.217831

C -2.543455 -0.888376 -1.928468

C -0.864067 -3.137897 -2.17033

H 0.296239 -2.269882 -0.605358

C -2.789844 -1.897968 -2.86894

C -1.963694 -3.011658 -3.012521

H -2.177409 -3.771115 -3.758197

C -2.87867 1.634479 -1.92147

H -2.024166 1.509628 -2.603012

C -3.552371 0.235849 -1.808175

H -4.181591 0.177496 -2.700787

C -4.482151 0.097777 -0.602484

C -5.462838 1.069628 -0.357799

C -4.415137 -0.98331 0.276732

C -6.326721 0.976778 0.725039

H -5.543475 1.921122 -1.024466

C -5.27585 -1.093646 1.36765

H -3.674309 -1.761312 0.125704

C -6.234241 -0.11019 1.596046

H -7.07859 1.739158 0.902787

H -5.196932 -1.943018 2.041347

O -3.756117 2.610684 -2.455215

O -7.108132 -0.159404 2.648286

O -3.901546 -1.774725 -3.658893

O -0.065503 -4.235641 -2.336586

O -1.991079 -4.647788 4.297504

H -6.939482 -0.956891 3.164177

H -3.956322 -2.537143 -4.246184

H 0.664186 -4.193078 -1.705705

H -2.879711 -4.458769 4.621308

H -4.013754 2.313686 -3.334429

O -2.360545 5.022267 1.651568

H -2.955044 5.499096 1.059713

C 0.669857 2.226531 -2.265713

C 2.776881 2.861346 2.011262

C 4.527383 -1.629837 1.767013

C 2.545592 -2.198856 -2.58101

C 1.808917 -1.041339 -2.846967

C 1.00237 -0.858975 -4.024429

C 0.451211 0.377493 -3.923646

C 0.935828 0.939245 -2.690214

N 1.748153 0.04698 -2.042229

C 1.135373 2.809748 -1.08219

C 0.882196 4.176499 -0.709284

C 1.463603 4.354467 0.503934

C 2.066884 3.096213 0.852784

N 1.859142 2.16773 -0.136346

C 3.438657 1.668716 2.328846

C 4.157146 1.442927 3.55199

C 4.645665 0.176051 3.482971

C 4.226472 -0.356521 2.215916

N 3.493132 0.576814 1.526656

C 4.157504 -2.180537 0.537081

C 4.506076 -3.509765 0.104691

C 3.961541 -3.666549 -1.129733

C 3.279757 -2.43454 -1.431238

N 3.420463 -1.543105 -0.404598

H 0.04885 2.842931 -2.908056

H 2.842849 3.670371 2.731859

H 5.111794 -2.259374 2.43087

H 2.504121 -2.991932 -3.320604

H 0.88261 -1.596444 -4.80602

H -0.215114 0.884156 -4.608367

H 0.31809 4.881383 -1.304212

H 1.485091 5.239215 1.125099

H 4.259977 2.168383 4.347295

H 5.237034 -0.363468 4.210159

H 5.089661 -4.21237 0.683804

H 3.998512 -4.52568 -1.785526

Fe 2.611384 0.302731 -0.244014

S 4.529145 1.046764 -1.272879

C 4.825328 2.774102 -0.794392

H 5.75854 3.090182 -1.268804

H 4.943398 2.873075 0.288182

H 4.023628 3.437084 -1.129015

O 1.207955 -0.626302 0.661304

CP1'_04.log

Energy (E) = -3229.88461944 Hartree

Enthalpy (H) = -3229.081831 Hartree

Gibbs free energy (G) = -3229.219905 Hartree

Charge = 0, Spin = 4

C -0.905826 -3.57489 2.438821

C -0.041801 -2.583103 2.930668

C -0.309805 -1.314591 2.49752

C -1.343851 -0.955985 1.612276

C -2.227398 -1.969542 1.161148

C -1.985424 -3.259586 1.578006

H 0.783087 -2.823218 3.589016

H -2.654606 -4.059833 1.273203

C -1.192476 0.42335 1.344978

C -0.099743 0.839492 2.166534

O 0.406484 -0.204472 2.849975

C 0.543926 2.098061 2.391959

C -0.152806 3.317907 2.301823

C 1.92689 2.111986 2.67834

C 0.510481 4.514873 2.480756

H -1.219596 3.32034 2.108729

C 2.597045 3.305498 2.830214

H 2.474888 1.177496 2.734738

C 1.890298 4.514539 2.732661

H -0.025913 5.456843 2.42668

H 3.665689 3.325472 3.013324

C -1.811893 1.156438 0.237204

C -1.03823 2.114965 -0.435135

C -3.088479 0.778419 -0.235527

C -1.536186 2.727476 -1.576947

H -0.033008 2.348575 -0.108789

C -3.505996 1.343907 -1.451926

C -2.760515 2.331052 -2.096127

H -3.124723 2.771492 -3.019277

C -3.458041 -1.663347 0.328201

C -3.932993 -0.217662 0.556336

O -4.661472 0.911643 -2.016731

O -0.854633 3.698762 -2.242114

O 2.591187 5.652508 2.891463

H -4.806263 1.384846 -2.844728

H 0.017381 3.809603 -1.842081

H 2.00754 6.417697 2.805581

O -0.677422 -4.828204 2.847461

H -1.3053 -5.440998 2.44159

C 0.080553 -2.927557 -1.665875

C 3.689593 -2.603482 1.52023

C 4.607379 1.881892 -0.012125

C 1.117197 1.480217 -3.323024

C 0.505728 0.255693 -3.124915

C -0.668319 -0.188147 -3.838604

C -0.969612 -1.420065 -3.361103

C 0.024421 -1.730688 -2.360453

N 0.903357 -0.696545 -2.232797

C 1.028867 -3.243505 -0.705285

C 1.073428 -4.502291 -0.002484

C 2.089386 -4.413424 0.891651

C 2.662349 -3.099596 0.736313

N 2.01892 -2.419121 -0.257594

C 4.240025 -1.337223 1.38971

C 5.266964 -0.806119 2.252103

C 5.522315 0.455219 1.821289

C 4.654313 0.690633 0.693837

N 3.892751 -0.414613 0.446845

C 3.754349 2.146045 -1.071575

C 3.717031 3.397526 -1.786148

C 2.747149 3.278532 -2.728612

C 2.187142 1.957704 -2.580309

N 2.821657 1.287134 -1.578144

H -0.67513 -3.674047 -1.888431

H 4.08341 -3.246297 2.300544

H 5.282589 2.673389 0.296311

H 0.696013 2.137214 -4.075807

H -1.179928 0.390748 -4.596083

H -1.782334 -2.076572 -3.641429

H 0.389725 -5.323477 -0.172346

H 2.422202 -5.146114 1.614608

H 5.715849 -1.345409 3.07574

H 6.226627 1.176736 2.214013

H 4.357445 4.243653 -1.57484

H 2.41572 4.006106 -3.457796

Fe 2.376836 -0.542642 -0.867846

S 3.921419 -1.390318 -2.560622

C 4.737575 -2.853569 -1.841565

H 5.47025 -3.244255 -2.554329

H 5.262994 -2.600922 -0.915899

H 4.018499 -3.648074 -1.620759

O 1.343748 0.081665 0.229014

C -5.439528 -0.033629 0.434796

C -6.022135 1.083677 1.035421

C -6.278111 -0.937855 -0.219926

C -7.394515 1.304496 0.985809

H -5.390565 1.800901 1.553846

C -7.652795 -0.737482 -0.273006

H -5.8596 -1.803933 -0.722879

C -8.215359 0.387527 0.329444

H -7.830001 2.179469 1.460442

H -8.297298 -1.444135 -0.78595

O -9.572566 0.541256 0.247747

H -9.828522 1.355924 0.69639

H -3.726218 -0.01151 1.612042

H -4.242513 -2.345934 0.677779

O -3.228483 -1.860456 -1.057284

H -2.93578 -2.770297 -1.187846

TS1'_04.log

Energy (E) = -3229.88081216 Hartree

Enthalpy (H) = -3229.078218 Hartree

Gibbs free energy (G) = -3229.214727 Hartree

Charge = 0, Spin = 4

Imaginary frequency: -293.5929

C 1.128112 -3.867582 -2.16882

C 0.165501 -2.965521 -2.638243

C 0.315735 -1.673062 -2.212528

C 1.344162 -1.203721 -1.375758

C 2.331008 -2.125544 -0.954848

C 2.198081 -3.442391 -1.35369

H -0.656774 -3.285378 -3.265265

H 2.948849 -4.170571 -1.058047

C 1.0886 0.172204 -1.136355

C -0.142848 0.445785 -1.846496

O -0.526486 -0.648511 -2.549729

C -0.713612 1.697063 -2.345578

C 0.061321 2.847907 -2.516034

C -2.074136 1.727438 -2.690153

C -0.513265 4.015194 -2.99918

H 1.119492 2.83622 -2.279536

C -2.656112 2.888337 -3.159935

H -2.680547 0.836761 -2.563842

C -1.875072 4.040987 -3.311545

H 0.088548 4.908583 -3.135496

H -3.712401 2.921044 -3.404563

C 1.73091 1.030362 -0.145832

C 0.97684 2.061272 0.438264

C 3.047926 0.75172 0.29396

C 1.514786 2.816205 1.470402

H -0.044412 2.242778 0.129188

C 3.499328 1.451865 1.424347

C 2.763747 2.496081 1.98465

H 3.157571 3.043065 2.835889

C 3.550963 -1.701996 -0.162725

C 3.918953 -0.241791 -0.473951

O 4.683957 1.099205 1.987652

O 0.847585 3.856618 2.042581

O -2.496347 5.155187 -3.77022

H 4.862151 1.677333 2.738793

H -0.037244 3.920306 1.660799

H -1.864509 5.882595 -3.828627

O 0.990155 -5.154102 -2.543575

H 1.701149 -5.690201 -2.169372

C -0.022782 -2.731552 1.753189

C -3.677764 -2.604374 -1.388937

C -4.677941 1.918006 -0.01546

C -1.132277 1.719585 3.249931

C -0.505323 0.494584 3.097379

C 0.680579 0.100965 3.815478

C 1.007608 -1.139178 3.375341

C 0.01381 -1.507729 2.398341

N -0.890773 -0.495778 2.243762

C -0.978044 -3.107314 0.823236

C -1.035485 -4.408082 0.206114

C -2.068664 -4.377588 -0.672428

C -2.636014 -3.055692 -0.594653

N -1.976623 -2.312844 0.340033

C -4.243132 -1.344743 -1.300305

C -5.311461 -0.87994 -2.147233

C -5.60176 0.385194 -1.751532

C -4.709772 0.693903 -0.663275

N -3.899558 -0.372938 -0.400791

C -3.799497 2.248438 1.000899

C -3.788666 3.521009 1.678749

C -2.802406 3.458823 2.607729

C -2.205345 2.151125 2.490913

N -2.828336 1.434997 1.510085

H 0.748817 -3.45321 2.000307

H -4.081329 -3.287218 -2.128957

H -5.389708 2.672692 -0.332518

H -0.721587 2.408139 3.979721

H 1.185021 0.716898 4.54794

H 1.83698 -1.767139 3.670657

H -0.349854 -5.217393 0.41867

H -2.41668 -5.155915 -1.338182

H -5.764729 -1.464584 -2.936666

H -6.343174 1.066938 -2.146537

H -4.460266 4.339185 1.455158

H -2.484275 4.215204 3.312949

Fe -2.361157 -0.412233 0.874663

S -3.755802 -1.274111 2.601644

C -5.039389 -2.292287 1.811693

H -5.688899 -2.68883 2.597699

H -5.650594 -1.707486 1.119602

H -4.602653 -3.13526 1.269912

O -1.315457 0.187081 -0.281052

C 5.414956 0.039879 -0.431669

C 5.905869 1.133566 -1.146512

C 6.331453 -0.757161 0.257433

C 7.263691 1.434515 -1.175483

H 5.212711 1.766945 -1.694668

C 7.692794 -0.476423 0.233595

H 5.984242 -1.600235 0.846602

C 8.162761 0.623601 -0.483777

H 7.627953 2.289238 -1.73887

H 8.3981 -1.10006 0.773537

O 9.510478 0.861187 -0.477229

H 9.695932 1.644269 -1.009198

H 3.656028 -0.101972 -1.52815

H 4.374671 -2.346181 -0.494555

O 3.366941 -1.843956 1.237849

H 3.122379 -2.760122 1.41395

INT1'_04.log

Energy (E) = -3229.92869966 Hartree

Enthalpy (H) = -3229.127911 Hartree

Gibbs free energy (G) = -3229.268508 Hartree

Charge = 0, Spin = 4

C -0.901683 -3.595992 2.582545

C 0.002253 -2.574492 2.900711

C -0.134927 -1.388065 2.217282

C -1.143301 -1.151043 1.249444

C -2.067836 -2.18272 0.98177

C -1.926251 -3.395889 1.648177

H 0.792975 -2.732054 3.624455

H -2.63607 -4.198375 1.461469

C -0.943731 0.151253 0.735665

C 0.315021 0.722772 1.404813

O 0.673746 -0.325007 2.394756

C 0.020623 1.98555 2.214881

C -1.078637 2.025001 3.075067

C 0.832112 3.113709 2.110917

C -1.366209 3.163421 3.816923

H -1.724987 1.155762 3.165942

C 0.555816 4.262148 2.845655

H 1.685767 3.095433 1.441198

C -0.545918 4.288338 3.700048

H -2.224219 3.18806 4.483191

H 1.184941 5.142497 2.762203

C -1.651428 0.847683 -0.310516

C -1.012358 1.93581 -0.940787

C -2.942367 0.430881 -0.745407

C -1.585981 2.539072 -2.047029

H -0.031018 2.253775 -0.610907

C -3.432146 1.000159 -1.930703

C -2.781286 2.058535 -2.567377

H -3.196124 2.492736 -3.471948

C -3.246652 -1.979319 0.057599

C -3.751542 -0.526658 0.128619

O -4.57742 0.506974 -2.481477

O -0.989784 3.581805 -2.700046

O -0.78098 5.438372 4.39864

H -4.806524 1.035116 -3.255198

H -0.128913 3.754014 -2.298402

H -1.573833 5.328268 4.937069

O -0.737335 -4.78269 3.228953

H -1.406922 -5.41065 2.931038

C 0.358142 -2.625914 -1.563619

C 3.850389 -2.269444 1.745356

C 4.899093 2.16771 0.150884

C 1.570649 1.706256 -3.310078

C 0.932443 0.502444 -3.0925

C -0.170777 0.031633 -3.881282

C -0.538951 -1.167833 -3.365678

C 0.353844 -1.441668 -2.275316

N 1.23688 -0.405238 -2.107555

C 1.257416 -2.920807 -0.560201

C 1.327786 -4.18804 0.107318

C 2.314865 -4.090424 1.033614

C 2.84485 -2.761032 0.938177

N 2.203113 -2.059816 -0.055378

C 4.406376 -1.009602 1.612054

C 5.471342 -0.513421 2.435779

C 5.767796 0.732493 1.984641

C 4.886415 0.995951 0.882771

N 4.059903 -0.079686 0.666599

C 4.088175 2.416211 -0.941817

C 4.139218 3.617083 -1.727248

C 3.214846 3.478985 -2.713297

C 2.595283 2.198192 -2.521179

N 3.142843 1.559465 -1.441175

H -0.369376 -3.383073 -1.831199

H 4.245197 -2.919383 2.518242

H 5.607271 2.936119 0.44075

H 1.223502 2.321184 -4.132516

H -0.599424 0.573965 -4.712875

H -1.33217 -1.829591 -3.68443

H 0.684807 -5.029021 -0.113474

H 2.658375 -4.833687 1.740041

H 5.923526 -1.066842 3.247583

H 6.515534 1.425273 2.346403

H 4.80862 4.444746 -1.535489

H 2.959105 4.168868 -3.506126

Fe 2.491149 -0.141203 -0.577709

S 4.270568 -1.823673 -2.360215

C 5.632906 -2.285762 -1.283927

H 6.394645 -2.773269 -1.903198

H 6.0667 -1.424006 -0.774672

H 5.291412 -3.024484 -0.551999

O 1.36388 0.909811 0.576078

C -5.264223 -0.401858 0.006481

C -5.889246 0.716226 0.561249

C -6.071223 -1.364682 -0.604044

C -7.269604 0.882207 0.50838

H -5.284363 1.478409 1.046491

C -7.452791 -1.220087 -0.660423

H -5.619705 -2.235644 -1.068918

C -8.056716 -0.09255 -0.104327

H -7.737063 1.759107 0.948008

H -8.071114 -1.972853 -1.139062

O -9.420068 0.004899 -0.186157

H -9.706795 0.826619 0.229883

H -3.541184 -0.206084 1.15567

H -4.043637 -2.642039 0.417178

O -2.957332 -2.293138 -1.301937

H -2.67598 -3.214662 -1.330825

INT1_2.log

Energy (E) = -3229.96795091 Hartree

Enthalpy (H) = -3229.164448 Hartree

Gibbs free energy (G) = -3229.299381 Hartree

Charge = 0, Spin = 2

C -1.10812 3.934558 -2.003109

C -0.165569 3.045163 -2.497297

C -0.183937 1.770274 -1.960202

C -1.164922 1.345883 -0.98787

C -2.174984 2.281109 -0.563749

C -2.119281 3.544403 -1.058547

H 0.583697 3.356952 -3.212813

H -2.867132 4.279923 -0.776872

C -0.873241 0.055758 -0.637532

C 0.39928 -0.400338 -1.389233

O 0.671764 0.837222 -2.27362

C 0.06365 -1.51612 -2.380335

C -1.049061 -1.437662 -3.223012

C 0.868649 -2.650517 -2.429283

C -1.35128 -2.470245 -4.098648

H -1.694519 -0.563303 -3.200251

C 0.577841 -3.693323 -3.303138

H 1.729619 -2.71918 -1.772542

C -0.535776 -3.604322 -4.138046

H -2.21508 -2.413216 -4.752607

H 1.20902 -4.577008 -3.335982

C -1.557423 -0.843513 0.281666

C -0.818391 -1.932443 0.762291

C -2.896402 -0.615679 0.701342

C -1.362946 -2.769781 1.725999

H 0.200692 -2.085891 0.431298

C -3.372635 -1.431735 1.741554

C -2.624995 -2.503846 2.233007

H -3.031637 -3.12693 3.024201

C -3.325368 1.86422 0.332637

C -3.756544 0.424183 -0.018974

O -4.585892 -1.172332 2.289907

O -0.689692 -3.837815 2.23078

O -0.877143 -4.595752 -5.010186

H -4.781684 -1.835535 2.96244

H 0.203228 -3.859525 1.861918

H -0.246904 -5.321727 -4.9295

O -1.066352 5.182941 -2.452475

H -1.762446 5.72217 -2.051825

C 3.697448 2.702676 -1.453256

C 4.829475 -1.931029 -0.697903

C 1.787446 -1.9873 3.046502

C 0.253583 2.445802 1.914611

C 1.10085 2.918472 0.925424

C 1.110812 4.278132 0.447824

C 2.097406 4.355329 -0.479945

C 2.678986 3.038869 -0.57589

N 2.060416 2.182687 0.291187

C 4.283098 1.449416 -1.556932

C 5.285841 1.105035 -2.534936

C 5.586166 -0.204419 -2.341909

C 4.777425 -0.654492 -1.236585

N 3.993575 0.366056 -0.779023

C 4.112552 -2.342746 0.414255

C 4.229246 -3.644819 1.024191

C 3.402126 -3.644924 2.100067

C 2.768179 -2.348137 2.134267

N 3.222722 -1.574154 1.108142

C 1.071291 -0.80076 3.01946

C -0.016585 -0.502126 3.917105

C -0.479095 0.726011 3.573052

C 0.337955 1.181707 2.474319

N 1.25709 0.227905 2.141264

H 4.058995 3.478836 -2.12015

H 5.503364 -2.64597 -1.158302

H 1.521793 -2.712867 3.808026

H -0.505796 3.119074 2.296577

H 0.435532 5.05253 0.786884

H 2.406185 5.207301 -1.071069

H 5.687481 1.790837 -3.269281

H 6.290714 -0.824669 -2.880094

H 4.869538 -4.438077 0.661559

H 3.212825 -4.439587 2.809698

H -0.367158 -1.164251 4.697607

H -1.280724 1.298359 4.022447

Fe 2.652845 0.300624 0.698842

S 4.131853 1.173241 2.177971

C 5.658755 0.199818 2.0208

H 6.403204 0.632012 2.695635

H 5.499551 -0.844651 2.29993

H 6.049927 0.239079 1.001143

O 1.4566 -0.643206 -0.657747

C -5.260438 0.209931 0.045352

C -5.830213 -0.775093 -0.762106

C -6.109803 0.973158 0.850016

C -7.202412 -1.005333 -0.769244

H -5.189215 -1.378076 -1.400518

C -7.483055 0.762751 0.849859

H -5.699129 1.731269 1.509607

C -8.033374 -0.23093 0.039489

H -7.630019 -1.775655 -1.405088

H -8.136748 1.358509 1.478739

O -9.390047 -0.401852 0.075509

H -9.636124 -1.111754 -0.529411

H -3.514325 0.300193 -1.081439

H -4.150598 2.543563 0.108913

O -3.050352 2.050352 1.708269

H -2.460724 1.339108 1.994765

TS2.log

Energy (E) = -3229.95714488 Hartree

Enthalpy (H) = -3229.148627 Hartree

Gibbs free energy (G) = -3229.283544 Hartree

Charge = 0, Spin = 2

Imaginary frequency: -325.3834

C -1.056781 3.54763 -2.486685

C -0.105137 2.600511 -2.861252

C -0.18726 1.373646 -2.235995

C -1.171663 1.055434 -1.275278

C -2.16147 2.012398 -0.958186

C -2.088885 3.245188 -1.567561

H 0.679787 2.836125 -3.568763

H -2.845752 3.997094 -1.36375

C -0.894675 -0.259258 -0.814343

C 0.405226 -0.656979 -1.453343

O 0.670465 0.364282 -2.460367

C 0.697979 -1.993584 -2.065567

C 0.083798 -2.361747 -3.265155

C 1.584644 -2.874263 -1.455388

C 0.356372 -3.590725 -3.847217

H -0.611323 -1.681148 -3.748211

C 1.873922 -4.105392 -2.032038

H 2.049412 -2.595156 -0.5174

C 1.258737 -4.464334 -3.231498

H -0.117004 -3.885778 -4.777692

H 2.573805 -4.784525 -1.553353

C -1.638548 -1.071055 0.167293

C -0.944495 -2.093167 0.826057

C -2.986564 -0.777001 0.490024

C -1.547148 -2.79856 1.856554

H 0.093843 -2.285577 0.601029

C -3.530463 -1.460786 1.595295

C -2.832052 -2.468554 2.258505

H -3.285325 -2.981076 3.101603

C -3.351736 1.673594 -0.081445

C -3.793925 0.21589 -0.351147

O -4.764744 -1.116149 2.040351

O -0.905879 -3.794757 2.525201

O 1.4982 -5.656108 -3.847459

H -5.007965 -1.68771 2.777959

H 0.019855 -3.817415 2.250027

H 2.136799 -6.162234 -3.330403

O -0.960443 4.763953 -3.051976

H -1.653157 5.348666 -2.71748

C 2.207968 3.604178 -0.744943

C 4.663231 -0.487114 -1.472911

C 2.758528 -2.271999 2.588101

C -0.196536 1.526595 2.8813

C 0.22061 2.43345 1.919998

C -0.350037 3.745952 1.743467

C 0.355942 4.345323 0.753353

C 1.342789 3.390247 0.312146

N 1.244155 2.236067 1.038285

C 3.07059 2.652628 -1.268248

C 3.848421 2.847404 -2.465166

C 4.502614 1.681353 -2.69963

C 4.140383 0.784335 -1.633209

N 3.261274 1.392315 -0.778778

C 4.393934 -1.31178 -0.393059

C 4.983289 -2.609248 -0.192436

C 4.470215 -3.09382 0.968097

C 3.556998 -2.096708 1.466292

N 3.534132 -1.016538 0.6287

C 1.785129 -1.386613 3.019043

C 0.881498 -1.651364 4.112856

C 0.022704 -0.60371 4.169175

C 0.418521 0.309007 3.124911

N 1.494013 -0.181847 2.44407

H 2.156468 4.563778 -1.248659

H 5.357952 -0.847752 -2.2241

H 2.875372 -3.196894 3.14362

H -1.03297 1.807265 3.513604

H -1.185978 4.131541 2.310995

H 0.224165 5.330742 0.326447

H 3.865729 3.762391 -3.042383

H 5.178605 1.433329 -3.507074

H 5.693124 -3.071683 -0.865175

H 4.665921 -4.041774 1.451591

H 0.906011 -2.54193 4.726261

H -0.806444 -0.440811 4.844653

Fe 2.436614 0.634248 0.865105

S 3.952125 1.578696 2.217149

C 5.563158 1.402469 1.39681

H 6.318872 1.831505 2.060873

H 5.808452 0.352459 1.221838

H 5.58728 1.939621 0.445758

O 0.932623 -0.351742 -0.247752

C -5.303774 0.026766 -0.341809

C -5.850627 -1.020103 -1.084534

C -6.179511 0.868674 0.348067

C -7.224317 -1.236699 -1.136318

H -5.190231 -1.685328 -1.635332

C -7.554136 0.672411 0.302157

H -5.790387 1.678619 0.957162

C -8.080998 -0.386031 -0.4389

H -7.632202 -2.057156 -1.720299

H -8.227546 1.328497 0.844331

O -9.44062 -0.54081 -0.446827

H -9.671648 -1.309413 -0.982077

H -3.511324 0.010924 -1.388585

H -4.159664 2.344308 -0.381371

O -3.133033 1.952052 1.289807

H -2.454842 1.34421 1.616041

INT2.log

Energy (E) = -3229.96499009 Hartree

Enthalpy (H) = -3229.160763 Hartree

Gibbs free energy (G) = -3229.296128 Hartree

Charge = 0, Spin = 2

C -1.034053 3.403661 -2.664171

C -0.132366 2.407783 -3.042451

C -0.180586 1.236419 -2.314791

C -1.032566 1.028553 -1.231067

C -2.008341 1.98151 -0.945683

C -1.983236 3.177808 -1.659127

H 0.562721 2.55116 -3.860767

H -2.732902 3.938521 -1.458476

C -0.702729 -0.301 -0.661854

C 0.343411 -0.822437 -1.614844

O 0.571586 0.130055 -2.621496

C 0.646023 -2.205066 -2.050934

C 0.100742 -2.673901 -3.250175

C 1.468165 -3.041988 -1.300787

C 0.372745 -3.960103 -3.689601

H -0.539193 -2.027997 -3.843022

C 1.750591 -4.331337 -1.733533

H 1.898419 -2.6842 -0.373715

C 1.201199 -4.792895 -2.930477

H -0.047329 -4.33271 -4.617628

H 2.399578 -4.975171 -1.146981

C -1.519294 -1.075694 0.329585

C -0.829881 -2.034421 1.072035

C -2.875805 -0.782169 0.588055

C -1.464322 -2.713911 2.10306

H 0.219471 -2.218176 0.89199

C -3.451828 -1.421437 1.705754

C -2.770673 -2.394235 2.435743

H -3.252475 -2.882247 3.277458

C -3.192804 1.66622 -0.048852

C -3.643923 0.200093 -0.301504

O -4.706395 -1.07272 2.094431

O -0.834632 -3.673981 2.835669

O 1.437414 -6.044857 -3.409997

H -4.953835 -1.59588 2.8661

H 0.094925 -3.712895 2.576033

H 2.025694 -6.517866 -2.8087

O -0.977878 4.579225 -3.347517

H -1.661157 5.17741 -3.020446

C 2.07851 3.658137 -0.846646

C 4.418923 -0.489894 -1.61706

C 2.791627 -2.113412 2.626144

C -0.157477 1.686704 2.941113

C 0.220862 2.566901 1.941269

C -0.338128 3.884159 1.770185

C 0.323134 4.453924 0.733576

C 1.268724 3.475705 0.25708

N 1.191749 2.332405 1.00855

C 2.894037 2.680722 -1.39598

C 3.59586 2.830219 -2.643285

C 4.209814 1.644729 -2.890492

C 3.902122 0.782722 -1.780902

N 3.088494 1.4278 -0.886262

C 4.215224 -1.275153 -0.495485

C 4.831702 -2.555478 -0.277299

C 4.405941 -2.990335 0.937268

C 3.514013 -1.982826 1.44849

N 3.416444 -0.941623 0.566026

C 1.833842 -1.2194 3.068121

C 1.000146 -1.438816 4.223823

C 0.133997 -0.397618 4.282228

C 0.459717 0.470655 3.179415

N 1.492202 -0.046938 2.449166

H 2.01474 4.608486 -1.365967

H 5.062855 -0.879887 -2.398157

H 2.956575 -3.007483 3.218537

H -0.954157 1.992075 3.611646

H -1.134938 4.293797 2.375757

H 0.186266 5.434606 0.297751

H 3.592671 3.730288 -3.24354

H 4.826599 1.362761 -3.733344

H 5.500001 -3.04137 -0.975426

H 4.646295 -3.912609 1.44925

H 1.071882 -2.298771 4.875889

H -0.654489 -0.209102 4.998337

Fe 2.3829 0.73799 0.838104

S 3.973125 1.696051 2.06765

C 5.53845 1.380635 1.203568

H 6.330946 1.858819 1.786326

H 5.748857 0.310998 1.137229

H 5.533098 1.812064 0.200282

O 0.738067 -0.371103 -0.355088

C -5.155685 0.027993 -0.334387

C -5.692418 -1.028762 -1.070777

C -6.043136 0.894013 0.30906

C -7.066242 -1.231498 -1.160482

H -5.023314 -1.713433 -1.586133

C -7.418214 0.71162 0.225302

H -5.664131 1.712461 0.91321

C -7.934523 -0.356821 -0.508608

H -7.465292 -2.060312 -1.738821

H -8.100134 1.386924 0.732149

O -9.295418 -0.49711 -0.555409

H -9.519217 -1.277382 -1.076761

H -3.333009 -0.03264 -1.324226

H -3.9985 2.333272 -0.363223

O -2.996737 1.959296 1.322941

H -2.320932 1.358312 1.665942

INT1'_2.log

Energy (E) = -3229.98278193 Hartree

Enthalpy (H) = -3229.179346 Hartree

Gibbs free energy (G) = -3229.314054 Hartree

Charge = 0, Spin = 2

C -2.040753 3.759359 1.090837

C -1.240573 2.945644 1.883604

C -0.93366 1.699107 1.370147

C -1.469937 1.225725 0.118056

C -2.286916 2.105779 -0.669292

C -2.556943 3.344466 -0.182324

H -0.841705 3.295369 2.8266

H -3.166151 4.042186 -0.745059

C -1.032201 -0.053708 -0.075807

C -0.072917 -0.438222 1.077838

O -0.158213 0.831011 1.961118

C -0.676515 -1.533409 1.959773

C -1.939867 -1.392699 2.540372

C 0.035934 -2.709552 2.17428

C -2.481484 -2.404497 3.319998

H -2.51815 -0.48658 2.384711

C -0.493607 -3.731919 2.955143

H 1.015962 -2.825963 1.723254

C -1.755172 -3.579729 3.529886

H -3.461734 -2.294523 3.772311

H 0.068946 -4.646726 3.119483

C -1.378318 -1.028444 -1.103872

C -0.556198 -2.160208 -1.210756

C -2.534123 -0.902764 -1.912197

C -0.847087 -3.156448 -2.130722

H 0.338603 -2.234559 -0.60812

C -2.818941 -1.954862 -2.794303

C -1.983471 -3.061909 -2.923144

H -2.218557 -3.847987 -3.634336

C -2.795539 1.613485 -1.987399

H -1.920271 1.444106 -2.632158

C -3.517086 0.247401 -1.84221

H -4.1246 0.180222 -2.748796

C -4.47265 0.163109 -0.651725

C -5.423741 1.171594 -0.442783

C -4.452557 -0.903839 0.246507

C -6.305129 1.126909 0.628622

H -5.467113 2.012181 -1.126675

C -5.332847 -0.965415 1.324625

H -3.736411 -1.709548 0.122935

C -6.261168 0.053457 1.520607

H -7.033463 1.91685 0.781473

H -5.292534 -1.804428 2.01427

O -3.621897 2.606751 -2.555559

O -7.149396 0.052817 2.559934

O -3.958127 -1.86567 -3.537549

O -0.049834 -4.247339 -2.29486

O -2.327493 -4.545736 4.304578

H -7.016617 -0.740365 3.093099

H -4.035415 -2.643328 -4.102588

H 0.734114 -4.156749 -1.737292

H -1.722467 -5.293618 4.3799

H -3.865196 2.30949 -3.43905

O -2.313823 4.974549 1.554627

H -2.876919 5.46748 0.94159

C 0.75617 2.268091 -2.277007

C 2.739561 2.807166 2.086825

C 4.579922 -1.645304 1.738281

C 2.504304 -2.218728 -2.579554

C 1.809045 -1.051881 -2.84413

C 1.018771 -0.835167 -4.030795

C 0.519092 0.421886 -3.939824

C 1.006769 0.972545 -2.697642

N 1.770099 0.051353 -2.038591

C 1.194017 2.814427 -1.081018

C 0.92999 4.17287 -0.674443

C 1.471632 4.321613 0.55949

C 2.071133 3.055956 0.902357

N 1.894251 2.154654 -0.112159

C 3.403223 1.623247 2.377659

C 4.124393 1.374768 3.600345

C 4.652576 0.128928 3.492655

C 4.250344 -0.38233 2.205695

N 3.494604 0.545571 1.546216

C 4.183513 -2.183396 0.523285

C 4.515079 -3.516758 0.082183

C 3.930546 -3.681716 -1.131823

C 3.243508 -2.448526 -1.427818

N 3.413322 -1.555401 -0.412517

H 0.161557 2.90016 -2.928674

H 2.774964 3.601312 2.825298

H 5.188069 -2.270334 2.384429

H 2.447475 -3.01723 -3.311878

H 0.878074 -1.567094 -4.815029

H -0.118008 0.95148 -4.636

H 0.382433 4.894186 -1.266473

H 1.46799 5.192418 1.201419

H 4.203698 2.076462 4.420131

H 5.257599 -0.41402 4.206862

H 5.113851 -4.219404 0.646733

H 3.946377 -4.547489 -1.780691

Fe 2.666576 0.30943 -0.262121

S 4.533733 1.065958 -1.29772

C 4.985243 2.660069 -0.552952

H 5.917467 2.992719 -1.018426

H 5.149741 2.564149 0.522905

H 4.218741 3.418994 -0.728526

O 1.163884 -0.660208 0.722363

TS2'.log

Energy (E) = -3229.97439036 Hartree

Enthalpy (H) = -3229.165494 Hartree

Gibbs free energy (G) = -3229.301815 Hartree

Charge = 0, Spin = 2

Imaginary frequency: -330.6139

C -2.066962 3.409163 1.568997

C -1.316098 2.472508 2.282723

C -1.054172 1.286664 1.632884

C -1.525371 1.003248 0.334085

C -2.297785 1.957023 -0.355394

C -2.567379 3.152255 0.271615

H -0.935391 2.691766 3.271669

H -3.171456 3.906963 -0.218232

C -1.099829 -0.301722 0.003779

C -0.182613 -0.727663 1.105072

O -0.333531 0.277419 2.153792

C -0.207061 -2.079488 1.749161

C -1.351736 -2.503575 2.427764

C 0.898906 -2.918823 1.67325

C -1.392041 -3.756257 3.021098

H -2.218646 -1.85035 2.486601

C 0.874045 -4.173985 2.271213

H 1.782151 -2.593718 1.13298

C -0.274554 -4.5938 2.943949

H -2.277751 -4.099253 3.545345

H 1.738049 -4.829884 2.213167

C -1.453876 -1.149626 -1.143395

C -0.60414 -2.215887 -1.46738

C -2.648687 -0.933907 -1.862141

C -0.920782 -3.069032 -2.513213

H 0.336888 -2.337409 -0.948762

C -2.966569 -1.863437 -2.868598

C -2.114846 -2.910274 -3.206956

H -2.370071 -3.587732 -4.016035

C -2.858134 1.585373 -1.694935

H -2.018546 1.45524 -2.391848

C -3.606347 0.217518 -1.625317

H -4.26829 0.242241 -2.494369

C -4.489935 0.05824 -0.390887

C -5.494456 1.000749 -0.134415

C -4.3352 -0.987089 0.520859

C -6.299204 0.915891 0.993809

H -5.640371 1.823871 -0.825848

C -5.135036 -1.088109 1.656966

H -3.580311 -1.749672 0.358905

C -6.118751 -0.132038 1.898326

H -7.071941 1.654341 1.18189

H -4.993982 -1.910538 2.35333

O -3.69159 2.63276 -2.145648

O -6.935725 -0.174559 2.993739

O -4.148449 -1.696221 -3.526753

O -0.093262 -4.080232 -2.896587

O -0.366022 -5.814754 3.542514

H -6.711527 -0.945972 3.527913

H -4.244813 -2.379717 -4.200588

H 0.735735 -4.020792 -2.401678

H 0.460244 -6.297588 3.419176

H -3.943454 2.432148 -3.053594

O -2.303391 4.584299 2.177932

H -2.8266 5.159436 1.604283

C 0.823063 3.563337 -0.947809

C 2.980798 1.592056 2.890113

C 4.241163 -2.186833 0.161964

C 1.544956 -0.490987 -3.462349

C 1.101519 0.763342 -3.079852

C 0.311119 1.632045 -3.914013

C 0.138166 2.785767 -3.219157

C 0.807222 2.61037 -1.954722

N 1.384893 1.374897 -1.889681

C 1.376127 3.381683 0.307961

C 1.314656 4.361669 1.363294

C 1.882599 3.7937 2.456363

C 2.308256 2.475047 2.061348

N 1.99509 2.245558 0.751212

C 3.508006 0.380719 2.474417

C 4.287553 -0.496309 3.311434

C 4.680199 -1.532834 2.529289

C 4.126864 -1.297754 1.218637

N 3.41882 -0.128056 1.208303

C 3.614254 -2.048859 -1.067825

C 3.621068 -3.061868 -2.094535

C 2.845833 -2.594253 -3.107001

C 2.375607 -1.294309 -2.69828

N 2.857083 -0.982443 -1.459787

H 0.328989 4.508926 -1.14544

H 3.145197 1.888963 3.920472

H 4.816395 -3.091234 0.331409

H 1.234499 -0.863561 -4.432875

H -0.048668 1.374368 -4.901277

H -0.404312 3.676358 -3.507551

H 0.863183 5.339911 1.264799

H 2.011416 4.20846 3.447193

H 4.498455 -0.318249 4.357501

H 5.278231 -2.394196 2.795986

H 4.150328 -4.002983 -2.025274

H 2.60039 -3.068456 -4.048005

Fe 2.473389 0.65857 -0.360469

S 4.322328 1.636395 -1.159499

C 5.11274 2.491889 0.234058

H 6.042668 2.933282 -0.135475

H 5.353933 1.79703 1.041772

H 4.478128 3.2904 0.625612

O 0.803939 -0.45452 0.223904

INT2'.log

Energy (E) = -3229.98260471 Hartree

Enthalpy (H) = -3229.179138 Hartree

Gibbs free energy (G) = -3229.316215 Hartree

Charge = 0, Spin = 2

C -2.267424 3.263946 1.589723

C -1.544406 2.31697 2.317829

C -1.178738 1.179011 1.630102

C -1.466345 0.962432 0.286668

C -2.264394 1.871434 -0.394608

C -2.653425 3.034367 0.262218

H -1.288843 2.475635 3.357892

H -3.273103 3.7651 -0.244918

C -0.883038 -0.341582 -0.094802

C -0.304364 -0.834232 1.202664

O -0.54061 0.111593 2.212118

C -0.164335 -2.211891 1.722463

C -1.181914 -2.743939 2.519914

C 0.962248 -2.979244 1.439751

C -1.072698 -4.029408 3.027572

H -2.062082 -2.147678 2.742711

C 1.083184 -4.266707 1.946822

H 1.750152 -2.56963 0.818522

C 0.064501 -4.792245 2.742983

H -1.856507 -4.454294 3.645343

H 1.965238 -4.861945 1.729369

C -1.284236 -1.199628 -1.251099

C -0.381417 -2.193418 -1.635874

C -2.511036 -1.039856 -1.919065

C -0.690726 -3.054811 -2.680174

H 0.582307 -2.271446 -1.149938

C -2.809984 -1.965044 -2.936982

C -1.918594 -2.960933 -3.322974

H -2.166838 -3.642229 -4.130972

C -2.789326 1.481095 -1.747172

H -1.949097 1.375829 -2.44515

C -3.497845 0.085227 -1.669017

H -4.173409 0.089656 -2.528022

C -4.359326 -0.092169 -0.424284

C -5.453805 0.756406 -0.215405

C -4.091346 -1.055062 0.55039

C -6.242304 0.659105 0.923799

H -5.68532 1.516271 -0.954864

C -4.872664 -1.168194 1.697674

H -3.260243 -1.742638 0.427455

C -5.951504 -0.307718 1.887623

H -7.08763 1.323001 1.07388

H -4.645866 -1.927483 2.441654

O -3.663352 2.493218 -2.205487

O -6.758453 -0.371214 2.990083

O -4.019355 -1.845422 -3.558587

O 0.178713 -4.01331 -3.110089

O 0.127195 -6.045889 3.269201

H -6.462069 -1.092379 3.55812

H -4.094148 -2.511323 -4.252331

H 1.032108 -3.88536 -2.673974

H 0.956914 -6.462316 3.0057

H -3.910332 2.273264 -3.110095

O -2.608102 4.408167 2.243974

H -3.114668 4.979207 1.653813

C 0.823119 3.819844 -0.404208

C 2.807806 1.115918 3.057675

C 4.119962 -2.093674 -0.297872

C 1.380337 0.197635 -3.541692

C 0.971667 1.380453 -2.952614

C 0.213296 2.401206 -3.628121

C 0.094203 3.436965 -2.758686

C 0.763836 3.039295 -1.547795

N 1.284976 1.78232 -1.680256

C 1.349466 3.404167 0.805215

C 1.279953 4.175099 2.020788

C 1.783061 3.393074 3.007829

C 2.187955 2.156668 2.388661

N 1.918817 2.183666 1.048023

C 3.331617 -0.005013 2.435299

C 4.094788 -1.022943 3.109392

C 4.522096 -1.886262 2.15362

C 3.996347 -1.410537 0.899959

N 3.272705 -0.264236 1.09272

C 3.476668 -1.746415 -1.475817

C 3.478214 -2.561503 -2.663932

C 2.682433 -1.932245 -3.567697

C 2.210527 -0.729313 -2.933838

N 2.702041 -0.634969 -1.661938

H 0.37128 4.805305 -0.444006

H 2.943121 1.21006 4.129787

H 4.705663 -3.006986 -0.295786

H 1.057885 -0.000755 -4.558333

H -0.163131 2.317039 -4.638845

H -0.408283 4.384723 -2.898532

H 0.86209 5.17049 2.090856

H 1.883749 3.611848 4.06247

H 4.277108 -1.046843 4.175423

H 5.122217 -2.779347 2.266436

H 4.017106 -3.493809 -2.76855

H 2.42425 -2.238152 -4.572591

Fe 2.38996 0.821416 -0.320331

S 4.252482 1.871725 -0.927684

C 5.049656 2.421723 0.608497

H 5.999324 2.888616 0.332125

H 5.255319 1.579641 1.272949

H 4.437662 3.15724 1.135281

O 0.556358 -0.378207 0.196341

INT1_2_04.log

Energy (E) = -3229.94672149 Hartree

Enthalpy (H) = -3229.145216 Hartree

Gibbs free energy (G) = -3229.284131 Hartree

Charge = 0, Spin = 4

C 1.168441 3.949879 2.014991

C 0.209624 3.077862 2.501545

C 0.218582 1.789984 1.982149

C 1.195591 1.348499 1.010609

C 2.200011 2.28253 0.567055

C 2.168897 3.544113 1.066027

H -0.534902 3.398673 3.218346

H 2.917827 4.272359 0.768977

C 0.916969 0.044096 0.694508

C -0.330812 -0.430622 1.480687

O -0.639003 0.872275 2.314348

C 0.07177 -1.457836 2.541132

C 1.161217 -1.25807 3.394552

C -0.653312 -2.64167 2.640184

C 1.512993 -2.216471 4.333693

H 1.748602 -0.345664 3.334709

C -0.311425 -3.612024 3.576752

H -1.493163 -2.803947 1.972817

C 0.773724 -3.398782 4.426665

H 2.356134 -2.062891 4.998982

H -0.881666 -4.53386 3.649713

C 1.590763 -0.865495 -0.225565

C 0.860068 -1.982821 -0.658061

C 2.90739 -0.619622 -0.701605

C 1.389842 -2.830161 -1.620356

H -0.140363 -2.157378 -0.284859

C 3.359411 -1.441013 -1.748462

C 2.621047 -2.540768 -2.187733

H 3.010449 -3.169401 -2.983449

C 3.30691 1.871775 -0.379894

C 3.777532 0.443559 -0.031164

O 4.539703 -1.160096 -2.355151

O 0.73398 -3.935957 -2.062655

O 1.160638 -4.310189 5.364985

H 4.718609 -1.828441 -3.027315

H -0.158704 -3.948166 -1.693422

H 0.573929 -5.07546 5.330472

O 1.152727 5.200018 2.46735

H 1.862954 5.722302 2.069292

C -2.869338 3.258131 0.855348

C -5.169934 -0.994028 1.271257

C -2.597307 -2.759536 -2.439857

C -0.171882 1.430514 -2.728711

C -0.662112 2.305833 -1.756436

C -0.258718 3.686756 -1.592308

C -1.028562 4.205915 -0.598958

C -1.900223 3.143122 -0.142785

N -1.63467 2.010271 -0.85001

C -3.746638 2.26289 1.298399

C -4.744071 2.421749 2.33667

C -5.3778 1.224994 2.462144

C -4.778016 0.327708 1.496487

N -3.794281 0.989516 0.82238

C -4.680391 -1.876115 0.300736

C -5.136899 -3.2315 0.082381

C -4.417373 -3.726874 -0.962615

C -3.517169 -2.677178 -1.3877

N -3.705789 -1.58205 -0.603384

C -1.711988 -1.767858 -2.868958

C -0.791746 -1.890292 -3.981896

C -0.098225 -0.723896 -4.050894

C -0.596898 0.123286 -2.986098

N -1.559862 -0.541642 -2.29362

H -2.955392 4.227618 1.33733

H -5.963792 -1.373531 1.908269

H -2.575264 -3.695549 -2.991043

H 0.592572 1.826032 -3.392183

H 0.51472 4.177958 -2.167735

H -1.010624 5.20929 -0.193571

H -4.923295 3.335288 2.888687

H -6.182485 0.96078 3.135907

H -5.907914 -3.727287 0.65775

H -4.482434 -4.709304 -1.412106

H -0.694782 -2.765709 -4.610259

H 0.678203 -0.445135 -4.751214

Fe -2.65611 0.205968 -0.702345

S -4.273222 1.003791 -2.352896

C -5.892506 0.983811 -1.549321

H -6.627363 1.233175 -2.322604

H -6.12923 -0.010168 -1.163346

H -5.959722 1.720155 -0.746272

O -1.382475 -0.737261 0.785245

C 5.280854 0.250388 -0.152202

C 5.894869 -0.720684 0.639712

C 6.088047 1.017884 -0.995119

C 7.268875 -0.934252 0.59381

H 5.287498 -1.326412 1.307728

C 7.462811 0.823803 -1.048577

H 5.642589 1.765686 -1.643892

C 8.05728 -0.157274 -0.254163

H 7.730647 -1.69429 1.218081

H 8.083455 1.422171 -1.707744

O 9.413274 -0.313389 -0.344242

H 9.690924 -1.022561 0.247813

H 3.580546 0.32817 1.041713

H 4.132848 2.566673 -0.215977

O 2.94488 2.037711 -1.738566

H 2.280357 1.370024 -1.958721

TS2_04.log

Energy (E) = -3229.94266402 Hartree

Enthalpy (H) = -3229.141198 Hartree

Gibbs free energy (G) = -3229.280734 Hartree

Charge = 0, Spin = 4

Imaginary frequency: -336.06

C -0.954052 3.335586 -2.7937

C -0.064702 2.32044 -3.142503

C -0.201054 1.127859 -2.461482

C -1.180699 0.912782 -1.463137

C -2.114253 1.936056 -1.177742

C -1.987031 3.131461 -1.847888

H 0.718789 2.48164 -3.872206

H -2.698535 3.933047 -1.669938

C -0.941038 -0.373415 -0.91812

C 0.332652 -0.863104 -1.53756

O 0.59931 0.067889 -2.647464

C 0.570934 -2.255711 -2.039644

C -0.175524 -2.769078 -3.101703

C 1.555649 -3.043064 -1.451726

C 0.061149 -4.053141 -3.570585

H -0.947454 -2.160678 -3.564717

C 1.808921 -4.328282 -1.915868

H 2.12038 -2.647777 -0.615077

C 1.059787 -4.833964 -2.979311

H -0.51493 -4.463577 -4.393162

H 2.581847 -4.936866 -1.454671

C -1.661919 -1.065943 0.162082

C -0.977586 -2.06335 0.868515

C -2.975901 -0.681667 0.525238

C -1.554617 -2.647252 1.986261

H 0.035412 -2.330364 0.603008

C -3.4822 -1.228369 1.720412

C -2.793376 -2.210386 2.430912

H -3.217023 -2.621649 3.342097

C -3.305471 1.713598 -0.262381

C -3.791408 0.252068 -0.373328

O -4.665244 -0.771729 2.20244

O -0.929707 -3.619326 2.703302

O 1.254847 -6.085956 -3.481813

H -4.895603 -1.25838 3.002957

H -0.026136 -3.726015 2.378931

H 1.97379 -6.516746 -3.003645

O -0.798416 4.520372 -3.408188

H -1.448097 5.157975 -3.083845

C 2.277485 3.503526 -1.06429

C 4.766609 -0.630975 -1.271529

C 2.569117 -2.140069 2.754166

C -0.197492 1.81509 2.718992

C 0.25472 2.619642 1.687008

C -0.2937 3.913029 1.375484

C 0.417897 4.396889 0.327044

C 1.394117 3.39541 -0.009449

N 1.282341 2.319967 0.834946

C 3.16255 2.509427 -1.450731

C 3.992595 2.585107 -2.620821

C 4.671746 1.410667 -2.694862

C 4.265847 0.62539 -1.562316

N 3.342852 1.311304 -0.816235

C 4.410337 -1.381258 -0.164596

C 4.926155 -2.691721 0.122818

C 4.316192 -3.11056 1.262572

C 3.423465 -2.057856 1.665125

N 3.503759 -1.007724 0.79193

C 1.633819 -1.180837 3.093339

C 0.697112 -1.321546 4.176404

C -0.102967 -0.226406 4.146375

C 0.358481 0.593562 3.057605

N 1.422757 0.003014 2.433775

H 2.237219 4.404938 -1.666034

H 5.494386 -1.054437 -1.955217

H 2.612701 -3.037528 3.361941

H -1.029577 2.175195 3.315052

H -1.125613 4.365198 1.897521

H 0.296987 5.332849 -0.201628

H 4.027577 3.432401 -3.292424

H 5.387387 1.087128 -3.438711

H 5.655274 -3.209697 -0.485597

H 4.435928 -4.047086 1.790448

H 0.658924 -2.171072 4.844237

H -0.935398 0.024041 4.789983

Fe 2.479818 0.716229 0.885591

S 4.164816 1.815177 2.320295

C 5.721684 1.532285 1.412971

H 6.55264 1.943187 1.992959

H 5.901764 0.464364 1.262593

H 5.705211 2.023891 0.436315

O 0.942982 -0.472449 -0.408255

C -5.303479 0.099333 -0.283835

C -5.894762 -1.025437 -0.860887

C -6.139741 1.044539 0.314644

C -7.272805 -1.215608 -0.840504

H -5.266059 -1.773701 -1.337169

C -7.518965 0.875588 0.337309

H -5.716766 1.919969 0.797102

C -8.090252 -0.25887 -0.239385

H -7.714804 -2.097287 -1.296286

H -8.161162 1.614072 0.806472

O -9.452207 -0.380331 -0.187852

H -9.715855 -1.204984 -0.613274

H -3.558412 -0.056187 -1.397503

H -4.098074 2.372121 -0.624632

O -3.067831 2.123947 1.071952

H -2.415052 1.523151 1.457775

INT2_04.log

Energy (E) = -3229.94889404 Hartree

Enthalpy (H) = -3229.147542 Hartree

Gibbs free energy (G) = -3229.287023 Hartree

Charge = 0, Spin = 4

C 1.066594 3.283429 2.803009

C 0.188319 2.267558 3.183783

C 0.23876 1.107609 2.437301

C 1.074853 0.930191 1.335721

C 2.023181 1.906374 1.039915

C 1.995927 3.090564 1.773184

H -0.494737 2.390083 4.015525

H 2.724201 3.869351 1.563584

C 0.744579 -0.388818 0.741679

C -0.286782 -0.935924 1.691665

O -0.500447 -0.011379 2.732235

C -0.611323 -2.328208 2.078539

C -0.018858 -2.88835 3.213552

C -1.520757 -3.080056 1.338172

C -0.326984 -4.184594 3.597224

H 0.687348 -2.307686 3.798847

C -1.839529 -4.378279 1.715161

H -1.984336 -2.647416 0.459743

C -1.239363 -4.933217 2.84654

H 0.130442 -4.63048 4.473855

H -2.553686 -4.957071 1.136431

C 1.546037 -1.132244 -0.285001

C 0.84693 -2.082982 -1.029394

C 2.891591 -0.81648 -0.573779

C 1.460252 -2.731715 -2.092431

H -0.193371 -2.289064 -0.819389

C 3.44653 -1.431095 -1.71592

C 2.755326 -2.393517 -2.450138

H 3.219996 -2.859437 -3.313683

C 3.178845 1.628286 0.095995

C 3.66787 0.166638 0.308976

O 4.690284 -1.06763 -2.125469

O 0.819644 -3.678856 -2.832268

O -1.504019 -6.200468 3.267493

H 4.924462 -1.576808 -2.910445

H -0.102882 -3.731041 -2.550982

H -2.137474 -6.615101 2.669069

O 1.002763 4.449054 3.503278

H 1.662773 5.06671 3.164872

C -2.041344 3.603028 1.123217

C -4.595867 -0.504776 1.403007

C -2.579617 -2.064199 -2.717033

C 0.127981 1.947211 -2.874586

C -0.248602 2.787931 -1.823997

C 0.32113 4.088997 -1.547667

C -0.294655 4.558348 -0.431366

C -1.239442 3.544157 -0.015125

N -1.187794 2.491663 -0.882549

C -2.918221 2.619247 1.587935

C -3.698628 2.693012 2.799958

C -4.406919 1.533006 2.88536

C -4.064306 0.747706 1.723291

N -3.155173 1.429017 0.966749

C -4.31634 -1.284922 0.279476

C -4.891723 -2.580649 -0.002473

C -4.334258 -3.015154 -1.164894

C -3.412863 -1.987658 -1.597692

N -3.432001 -0.955077 -0.705983

C -1.634324 -1.121867 -3.12567

C -0.746212 -1.259282 -4.258012

C 0.031874 -0.144405 -4.285505

C -0.389244 0.686426 -3.179655

N -1.392658 0.063648 -2.49712

H -1.949777 4.496592 1.733641

H -5.314558 -0.915648 2.106091

H -2.66076 -2.963043 -3.321454

H 0.913388 2.316202 -3.528084

H 1.099545 4.559087 -2.133511

H -0.123848 5.492424 0.087435

H -3.695606 3.528292 3.487856

H -5.101308 1.227046 3.656644

H -5.61837 -3.083476 0.621872

H -4.512423 -3.945765 -1.687438

H -0.721468 -2.110802 -4.924486

H 0.818166 0.1081 -4.984441

Fe -2.428015 0.838066 -0.875525

S -4.151771 1.952008 -2.070222

C -5.697573 1.164871 -1.545944

H -6.520011 1.760692 -1.954326

H -5.778522 0.14179 -1.918034

H -5.787027 1.167596 -0.456435

O -0.698718 -0.444542 0.456677

C 5.183577 0.030404 0.293078

C 5.770443 -1.015178 1.00678

C 6.027782 0.922585 -0.372729

C 7.151497 -1.182041 1.053228

H 5.135923 -1.719491 1.539187

C 7.408823 0.776382 -0.332314

H 5.608752 1.733993 -0.959678

C 7.975528 -0.281078 0.37998

H 7.590179 -2.002128 1.615091

H 8.056895 1.471969 -0.85588

O 9.340522 -0.383948 0.384035

H 9.601605 -1.157884 0.897164

H 3.393096 -0.089295 1.336436

H 3.983236 2.307236 0.387123

O 2.919912 1.936782 -1.262388

H 2.247746 1.322158 -1.587834

INT1'_2_04.log

Energy (E) = -3229.97213389 Hartree

Enthalpy (H) = -3229.169212 Hartree

Gibbs free energy (G) = -3229.308189 Hartree

Charge = 0, Spin = 4

C -1.784797 3.808152 1.229729

C -1.1324 2.893882 2.044059

C -0.933712 1.627935 1.516896

C -1.434234 1.247266 0.214811

C -2.123628 2.226339 -0.581525

C -2.283501 3.47528 -0.073979

H -0.750378 3.177796 3.015838

H -2.79217 4.244132 -0.643784

C -1.088807 -0.054259 -0.01386

C -0.238667 -0.587151 1.166779

O -0.293491 0.675083 2.127878

C -0.986925 -1.661794 1.954176

C -2.282836 -1.46044 2.437503

C -0.373124 -2.892618 2.166625

C -2.952087 -2.466134 3.119737

H -2.787567 -0.511635 2.279833

C -1.031055 -3.910716 2.84958

H 0.631322 -3.053073 1.788577

C -2.324124 -3.697046 3.326844

H -3.958466 -2.310679 3.49476

H -0.546771 -4.869571 3.011958

C -1.414667 -0.936797 -1.130146

C -0.640917 -2.098782 -1.272334

C -2.499987 -0.686099 -2.002397

C -0.909798 -3.000176 -2.291781

H 0.201741 -2.269653 -0.615148

C -2.764037 -1.642236 -2.993823

C -1.975599 -2.779117 -3.154376

H -2.190754 -3.490603 -3.945773

C -2.626858 1.833074 -1.936285

H -1.745738 1.647807 -2.567449

C -3.428004 0.506669 -1.895232

H -4.004493 0.522484 -2.824147

C -4.430071 0.397159 -0.745364

C -5.248616 1.485008 -0.411303

C -4.593761 -0.782698 -0.019063

C -6.175155 1.405868 0.619442

H -5.156004 2.412876 -0.964825

C -5.525371 -0.880853 1.012188

H -3.983024 -1.65101 -0.244227

C -6.31563 0.2177 1.338941

H -6.797102 2.258439 0.872758

H -5.630048 -1.809614 1.566377

O -3.373878 2.906653 -2.469256

O -7.241659 0.188973 2.344626

O -3.833171 -1.426341 -3.811042

O -0.158006 -4.11712 -2.491733

O -3.0226 -4.656239 4.000426

H -7.23558 -0.681068 2.7616

H -3.905214 -2.145831 -4.449315

H 0.587201 -4.117245 -1.876783

H -2.481867 -5.452075 4.071226

H -3.598299 2.681922 -3.378823

O -1.935678 5.043435 1.697821

H -2.387597 5.60632 1.053847

C 0.909777 2.444727 -2.047739

C 2.948669 2.493604 2.330451

C 4.49509 -2.028727 1.638502

C 2.387329 -2.100979 -2.707607

C 1.765187 -0.878665 -2.875537

C 0.981288 -0.52706 -4.030084

C 0.557009 0.747716 -3.841061

C 1.082152 1.175676 -2.570293

N 1.799392 0.165041 -1.989856

C 1.388427 2.858087 -0.816375

C 1.210901 4.184448 -0.291494

C 1.767905 4.193862 0.946158

C 2.293714 2.875451 1.175561

N 2.058264 2.075119 0.086221

C 3.534359 1.251764 2.524281

C 4.227612 0.862354 3.723233

C 4.672184 -0.4051 3.522482

C 4.247278 -0.789089 2.202674

N 3.56046 0.236409 1.610932

C 4.063461 -2.431141 0.385506

C 4.301069 -3.737293 -0.167091

C 3.708555 -3.758688 -1.389768

C 3.110337 -2.464964 -1.581667

N 3.339465 -1.671395 -0.493684

H 0.347666 3.161824 -2.636448

H 3.031574 3.222424 3.129403

H 5.05651 -2.745134 2.228856

H 2.276283 -2.837122 -3.496009

H 0.791494 -1.188757 -4.864452

H -0.052053 1.363635 -4.48934

H 0.702687 4.984818 -0.812226

H 1.819536 5.005353 1.659513

H 4.346553 1.492848 4.5942

H 5.233673 -1.040587 4.194232

H 4.847939 -4.52443 0.334552

H 3.665185 -4.566251 -2.108433

Fe 2.747694 0.226685 -0.223257

S 4.872862 1.03947 -1.271902

C 5.282614 2.5454 -0.328081

H 6.233535 2.948606 -0.688099

H 5.385712 2.326095 0.73842

H 4.514916 3.315006 -0.450415

O 0.998659 -0.847242 0.882806

TS2'_04.log

Energy (E) = -3229.94230968 Hartree

Enthalpy (H) = -3229.149757 Hartree

Gibbs free energy (G) = -3229.289696 Hartree

Charge = 0, Spin = 4

Imaginary frequency: -341.2138

C -1.873119 3.395025 1.741552

C -1.214846 2.378643 2.437157

C -1.029654 1.197691 1.751152

C -1.47293 1.005038 0.425244

C -2.151947 2.040184 -0.24543

C -2.356581 3.225267 0.423766

H -0.849226 2.531412 3.444396

H -2.88699 4.042178 -0.051268

C -1.126837 -0.310904 0.049403

C -0.288912 -0.854567 1.158139

O -0.414831 0.113855 2.254792

C -0.432377 -2.231058 1.731539

C -1.635379 -2.623933 2.32187

C 0.631409 -3.125521 1.675433

C -1.775927 -3.899986 2.847489

H -2.468557 -1.927054 2.366098

C 0.505416 -4.404635 2.205463

H 1.562157 -2.822708 1.204795

C -0.700792 -4.792548 2.790741

H -2.707495 -4.218447 3.303294

H 1.335699 -5.103877 2.162488

C -1.479833 -1.071118 -1.158662

C -0.67891 -2.164924 -1.513483

C -2.630892 -0.745413 -1.906505

C -1.002727 -2.938158 -2.61796

H 0.230052 -2.37346 -0.965647

C -2.961861 -1.597454 -2.97534

C -2.157985 -2.671249 -3.343597

H -2.417828 -3.286689 -4.199473

C -2.685858 1.767705 -1.618998

H -1.830868 1.610537 -2.290752

C -3.523996 0.451686 -1.644129

H -4.148916 0.561257 -2.533816

C -4.462059 0.291688 -0.451094

C -5.401035 1.29341 -0.172024

C -4.421831 -0.813156 0.400412

C -6.251738 1.207076 0.921625

H -5.457447 2.163965 -0.816757

C -5.269491 -0.9166 1.501292

H -3.720224 -1.62087 0.218109

C -6.186279 0.097901 1.766848

H -6.972084 1.99202 1.128493

H -5.217614 -1.786341 2.150882

O -3.426886 2.889629 -2.051218

O -7.044878 0.058636 2.830186

O -4.105463 -1.324588 -3.665147

O -0.221553 -3.975162 -3.028603

O -0.88734 -6.034688 3.320605

H -6.901988 -0.7561 3.326658

H -4.212669 -1.964432 -4.378972

H 0.600853 -3.977796 -2.518799

H -0.075917 -6.548801 3.227174

H -3.651466 2.747668 -2.97725

O -2.036848 4.562475 2.388272

H -2.491774 5.196914 1.819245

C 0.897921 3.564201 -0.847374

C 3.17788 1.481166 2.892198

C 4.07545 -2.435216 0.182181

C 1.609553 -0.436387 -3.487193

C 1.174053 0.833373 -3.10215

C 0.444668 1.760777 -3.937947

C 0.258774 2.89217 -3.202824

C 0.86334 2.655528 -1.910447

N 1.398228 1.404935 -1.883601

C 1.461141 3.36342 0.412729

C 1.47321 4.329317 1.491417

C 2.093672 3.736848 2.545714

C 2.474484 2.407258 2.117269

N 2.070514 2.216498 0.829369

C 3.647529 0.223636 2.497891

C 4.381284 -0.704807 3.331366

C 4.64343 -1.799627 2.566901

C 4.072588 -1.548115 1.261006

N 3.485712 -0.318193 1.259366

C 3.489137 -2.244169 -1.074305

C 3.432404 -3.233328 -2.130663

C 2.744138 -2.670238 -3.161355

C 2.371913 -1.336865 -2.737231

N 2.846421 -1.114484 -1.480698

H 0.42784 4.528009 -1.018764

H 3.404253 1.781296 3.911146

H 4.559493 -3.393631 0.3466

H 1.322439 -0.762952 -4.482609

H 0.127954 1.563131 -4.953678

H -0.250394 3.802288 -3.491807

H 1.043634 5.321012 1.437586

H 2.284494 4.147825 3.528373

H 4.648913 -0.531449 4.365483

H 5.169784 -2.701972 2.849696

H 3.865539 -4.2236 -2.076627

H 2.496525 -3.107322 -4.119844

Fe 2.505639 0.574113 -0.328434

S 4.556007 1.565303 -1.110024

C 5.175749 2.61387 0.226781

H 6.175349 2.946681 -0.072705

H 5.271541 2.049227 1.157115

H 4.54315 3.489001 0.386317

O 0.761661 -0.604834 0.354643

INT2'_04.log

Energy (E) = -3229.96621170 Hartree

Enthalpy (H) = -3229.165121 Hartree

Gibbs free energy (G) = -3229.306413 Hartree

Charge = 0, Spin = 4

C -0.679708 3.379175 2.047807

C -0.65488 2.154378 2.720066

C -0.837712 1.031546 1.937115

C -0.969998 1.074622 0.551969

C -1.072109 2.302093 -0.087285

C -0.923356 3.458557 0.670794

H -0.52343 2.100996 3.793824

H -0.990098 4.431817 0.197761

C -1.044214 -0.320568 0.065273

C -1.031816 -1.114684 1.334386

O -0.955327 -0.241769 2.440221

C -1.673326 -2.410263 1.649184

C -2.981368 -2.428891 2.140901

C -0.988622 -3.609514 1.462872

C -3.600875 -3.6331 2.438652

H -3.515645 -1.494718 2.289132

C -1.600555 -4.821421 1.759152

H 0.0297 -3.595841 1.084959

C -2.908391 -4.832831 2.248263

H -4.616798 -3.660414 2.817785

H -1.068435 -5.757319 1.615995

C -1.574903 -0.781103 -1.254079

C -1.199229 -2.061305 -1.668114

C -2.414117 0.018745 -2.050285

C -1.669679 -2.572785 -2.871105

H -0.514599 -2.650953 -1.071205

C -2.899585 -0.55023 -3.241881

C -2.53753 -1.827042 -3.658296

H -2.911055 -2.231368 -4.593921

C -1.469543 2.314814 -1.535632

H -0.682189 1.837139 -2.12984

C -2.767612 1.464673 -1.7553

H -3.188504 1.868969 -2.679073

C -3.812712 1.655901 -0.663068

C -4.34106 2.932111 -0.430784

C -4.252599 0.614069 0.154894

C -5.255873 3.165808 0.587539

H -4.016999 3.761775 -1.05087

C -5.171778 0.829744 1.179057

H -3.879164 -0.394075 0.004511

C -5.671011 2.110841 1.401848

H -5.654598 4.160358 0.760118

H -5.498211 0.002646 1.804255

O -1.630962 3.654128 -1.958521

O -6.568661 2.388274 2.395849

O -3.743937 0.205355 -4.00314

O -1.310184 -3.807997 -3.323174

O -3.560123 -5.987615 2.558442

H -6.763371 1.577152 2.880214

H -3.974473 -0.280925 -4.80342

H -0.625536 -4.167692 -2.744462

H -2.974877 -6.739251 2.40467

H -1.710751 3.641584 -2.918539

O -0.488146 4.496868 2.801261

H -0.522233 5.276417 2.23364

C 2.212805 3.422664 -0.678481

C 3.162117 0.556476 3.103081

C 2.769293 -3.344291 0.261385

C 1.64862 -0.489123 -3.481919

C 1.677971 0.83992 -3.054165

C 1.403744 1.990773 -3.882709

C 1.577615 3.0908 -3.099718

C 1.949051 2.615152 -1.787907

N 1.992975 1.251445 -1.788933

C 2.532729 3.001277 0.611783

C 2.749746 3.866175 1.750309

C 2.994664 3.058882 2.816046

C 2.938955 1.69766 2.331223

N 2.649477 1.698064 0.997567

C 3.161976 -0.775704 2.674624

C 3.355168 -1.93041 3.521861

C 3.246926 -3.026362 2.722673

C 2.990173 -2.544719 1.384642

N 2.953234 -1.181209 1.390962

C 2.452608 -2.921804 -1.032961

C 2.148628 -3.79418 -2.146555

C 1.839671 -2.991366 -3.200623

C 1.946828 -1.62829 -2.730961

N 2.333997 -1.621521 -1.424076

H 2.136743 4.494881 -0.833198

H 3.358504 0.721187 4.158576

H 2.817527 -4.418097 0.418544

H 1.354914 -0.655889 -4.514268

H 1.120071 1.948092 -4.925875

H 1.460633 4.131632 -3.370379

H 2.701074 4.946632 1.724247

H 3.193841 3.342134 3.841012

H 3.537849 -1.893003 4.587599

H 3.32479 -4.069019 3.001149

H 2.167934 -4.875499 -2.109776

H 1.542951 -3.280395 -4.199887

Fe 2.725678 0.051585 -0.258657

S 5.033854 0.252648 -0.738287

C 5.784154 0.962704 0.754481

H 6.870881 0.901863 0.642145

H 5.503609 0.391098 1.643357

H 5.499016 2.008196 0.890505

O 0.114612 -1.080815 0.543992

P450_III.log

Energy (E) = -1549.62053179 Hartree

Enthalpy (H) = -1549.280088 Hartree

Gibbs free energy (G) = -1549.351816 Hartree

Charge = 0, Spin = 2

C -0.07628 -3.390536 0.172801

C 3.313757 0.008408 0.63433

C -0.092989 3.390599 0.17192

C -3.510516 -0.008377 0.290957

C -2.862129 -1.229462 0.272636

C -3.534204 -2.499263 0.207485

C -2.567105 -3.448326 0.141375

C -1.305912 -2.757323 0.183023

N -1.500294 -1.402044 0.270986

C 1.142159 -2.74877 0.297828

C 2.406224 -3.432289 0.360151

C 3.357967 -2.480128 0.527504

C 2.675447 -1.215023 0.551539

N 1.319859 -1.393158 0.42373

C 2.669318 1.228663 0.551473

C 3.3456 2.497093 0.527268

C 2.38921 3.444558 0.359689

C 1.128543 2.754841 0.297367

N 1.312876 1.400104 0.42359

C -1.319518 2.751371 0.182006

C -2.584102 3.436148 0.140207

C -3.546536 2.482371 0.206623

C -2.868211 1.215926 0.272066

N -1.507263 1.395158 0.270218

H -0.068098 -4.472563 0.093075

H 4.394637 0.011139 0.726233

H -0.090064 4.472637 0.091971

H -4.595448 -0.011101 0.287079

H -4.608704 -2.624291 0.207766

H -2.674818 -4.52299 0.079384

H 2.525684 -4.505264 0.290828

H 4.428965 -2.599983 0.621002

H 4.415996 2.62221 0.62079

H 2.503417 4.51809 0.290151

H -2.697123 4.510255 0.077976

H -4.621634 2.602116 0.206948

Fe -0.078518 -3.3e-05 0.172933

S -0.003913 -0.000257 -2.059701

C 1.753941 0.000102 -2.510626

H 1.802968 0.002986 -3.603463

H 2.262436 0.890891 -2.135759

H 2.261638 -0.893088 -2.140425

Epoxy1.log

Energy (E) = -1680.33801218 Hartree

Enthalpy (H) = -1679.877321 Hartree

Gibbs free energy (G) = -1679.966992 Hartree

Charge = 0, Spin = 1

C 2.402089 3.392358 1.506194

C 1.190387 3.64806 0.858537

C 0.436473 2.539891 0.532015

C 0.831658 1.229738 0.798348

C 2.052503 0.988123 1.419605

C 2.828525 2.08888 1.783172

H 0.869356 4.658148 0.635586

H 3.788716 1.938487 2.263778

C -0.223867 0.342766 0.259096

C -1.208885 1.282148 -0.335701

O -0.785856 2.607868 -0.090684

C -2.678264 1.182812 -0.524561

C -3.208639 0.319987 -1.482263

C -3.541962 1.920974 0.288222

C -4.583016 0.188511 -1.629172

H -2.541859 -0.262252 -2.108258

C -4.916384 1.796115 0.149634

H -3.137974 2.59345 1.037513

C -5.43989 0.925806 -0.809589

H -4.995813 -0.484171 -2.375093

H -5.593592 2.363881 0.778476

C -0.470025 -1.029051 0.79054

C -1.766388 -1.41787 1.125078

C 0.62053 -1.893904 0.966924

C -1.996768 -2.701015 1.612469

H -2.59831 -0.731871 1.024785

C 0.351509 -3.178535 1.466332

C -0.941551 -3.589272 1.778663

H -1.126185 -4.589483 2.158542

C 2.532711 -0.42101 1.697642

H 2.112592 -0.725863 2.666905

C 2.056655 -1.486224 0.675453

H 2.669854 -2.359908 0.91666

C 2.305866 -1.176163 -0.799378

C 3.235 -0.225075 -1.231766

C 1.62115 -1.90112 -1.778825

C 3.453848 0.013313 -2.584363

H 3.806276 0.345058 -0.508892

C 1.831554 -1.679825 -3.134805

H 0.894275 -2.65103 -1.481374

C 2.748864 -0.712369 -3.542255

H 4.171414 0.761784 -2.904475

H 1.282226 -2.252247 -3.877104

O 3.947842 -0.392861 1.797422

O 2.995454 -0.438635 -4.860784

O 1.405805 -4.027685 1.636303

O -3.244643 -3.134025 1.949991

O -6.796343 0.835744 -0.901283

O -0.3693 0.55849 -1.198531

H 2.444749 -1.006286 -5.413784

H 1.087729 -4.867505 1.988515

H -3.884635 -2.437248 1.758761

H -7.033986 0.202954 -1.590401

H 4.224988 -1.239787 2.164335

O 3.153732 4.475467 1.849066

H 3.96455 4.18069 2.282042

Epoxy1'.log

Energy (E) = -1680.34034840 Hartree

Enthalpy (H) = -1679.879128 Hartree

Gibbs free energy (G) = -1679.967152 Hartree

Charge = 0, Spin = 1

C 3.281168 1.961094 1.838961

C 2.049804 2.618402 1.791478

C 0.94048 1.813077 1.626718

C 0.998274 0.423313 1.567763

C 2.235403 -0.206871 1.552344

C 3.379746 0.570738 1.699353

H 1.982135 3.698143 1.841665

H 4.359304 0.106898 1.681299

C -0.387063 -0.075668 1.410339

C -1.187246 1.183345 1.327558

O -0.329216 2.298479 1.433872

C -2.408367 1.469333 0.539484

C -2.268603 1.91833 -0.775229

C -3.683912 1.294194 1.074249

C -3.387897 2.179808 -1.552227

H -1.27684 2.060893 -1.194587

C -4.81054 1.553522 0.305845

H -3.796899 0.950997 2.097498

C -4.662369 1.993777 -1.010693

H -3.280017 2.523038 -2.576658

H -5.806697 1.419154 0.713119

C -0.802326 -1.384539 0.815941

C -2.128714 -1.774406 1.02292

C 0.066284 -2.171567 0.039062

C -2.625731 -2.922537 0.419713

H -2.775902 -1.187356 1.661682

C -0.485469 -3.30881 -0.5822

C -1.810003 -3.689382 -0.403003

H -2.201315 -4.578415 -0.8876

C 2.283256 -1.669909 1.211375

H 1.747813 -2.243144 1.979926

C 1.553412 -1.929555 -0.14935

H 1.966846 -2.884644 -0.482803

C 1.895168 -0.900861 -1.219536

C 3.201606 -0.826035 -1.716857

C 0.960788 0.011478 -1.711997

C 3.568536 0.131751 -2.653161

H 3.949359 -1.524305 -1.355658

C 1.309843 0.976542 -2.652292

H -0.068271 -0.021764 -1.368461

C 2.620051 1.041719 -3.121487

H 4.584954 0.180876 -3.030071

H 0.565044 1.676038 -3.022572

O 3.634872 -2.082576 1.162444

O 3.028707 1.967149 -4.042909

O 0.338486 -4.046085 -1.382546

O -3.912215 -3.340971 0.594513

O -5.796111 2.227851 -1.728442

O -1.245036 0.41963 2.502375

H 2.281527 2.528009 -4.283837

H -0.145675 -4.804922 -1.729218

H -4.373971 -2.728238 1.180172

H -5.558893 2.525112 -2.615686

H 3.640526 -3.035879 1.022359

O 4.387693 2.740718 1.988823

H 5.176062 2.184257 1.973958

INT2_2.log

Energy (E) = -1909.61893290 Hartree

Enthalpy (H) = -1909.074155 Hartree

Gibbs free energy (G) = -1909.179640 Hartree

Charge = 0, Spin = 1

C 2.900384 1.731097 2.037032

C 1.868455 2.47994 1.459383

C 0.776754 1.766156 1.013276

C 0.65969 0.383783 1.114955

C 1.670657 -0.349511 1.734662

C 2.78686 0.340826 2.196658

H 1.93637 3.553455 1.339907

H 3.607871 -0.192998 2.658228

C -0.580722 0.002989 0.408476

C -1.131425 1.300945 -0.068572

O -0.303253 2.345228 0.390336

C -2.519137 1.757581 -0.331706

C -3.208837 1.315961 -1.459455

C -3.154054 2.612064 0.572782

C -4.518317 1.718732 -1.686636

H -2.722377 0.644878 -2.158399

C -4.461518 3.019248 0.354948

H -2.624546 2.958056 1.454201

C -5.146819 2.571766 -0.777321

H -5.055805 1.376305 -2.56599

H -4.962516 3.681529 1.052586

C -1.313187 -1.273201 0.649322

C -2.699106 -1.245192 0.800532

C -0.594706 -2.479012 0.706044

C -3.399068 -2.434854 0.977149

H -3.241556 -0.308003 0.793431

C -1.337606 -3.659929 0.874504

C -2.723567 -3.648304 1.003554

H -3.273091 -4.575183 1.136398

C 1.537179 -1.845686 1.898639

H 0.839208 -2.022728 2.729269

C 0.923385 -2.563754 0.662479

H 1.163646 -3.616428 0.83774

C 1.545653 -2.199443 -0.683369

C 2.918406 -1.940918 -0.799746

C 0.787991 -2.19384 -1.855048

C 3.505018 -1.669688 -2.029784

H 3.54358 -1.95662 0.085206

C 1.359715 -1.923374 -3.095384

H -0.277277 -2.395542 -1.808675

C 2.722825 -1.656086 -3.185104

H 4.56839 -1.466214 -2.103406

H 0.745937 -1.916916 -3.991737

O 2.804392 -2.377076 2.245855

O 3.344773 -1.374381 -4.3701

O -0.654506 -4.840004 0.910031

O -4.752841 -2.463138 1.130717

O -6.428841 3.001286 -0.942849

O -0.510948 0.461156 -1.012249

H 2.698004 -1.40446 -5.085662

H -1.276567 -5.565799 1.038919

H -5.097755 -1.56365 1.072343

H -6.793919 2.627609 -1.75432

H 2.671393 -3.298928 2.493131

O 4.041144 2.318448 2.467205

O 2.154119 1.313087 -1.811485

H 2.653657 0.684395 -1.275233

H 1.22998 1.09115 -1.608036

H 2.696249 2.965155 -1.348935

O 3.029239 3.883762 -1.260725

H 3.695088 3.957658 -1.952817

H 4.141783 3.201393 2.021977

O 4.333372 4.54807 1.046833

H 3.875676 4.278342 0.215196

H 3.75595 5.222247 1.422499

TS3.log

Energy (E) = -1909.60918220 Hartree

Enthalpy (H) = -1909.063346 Hartree

Gibbs free energy (G) = -1909.167343 Hartree

Charge = 0, Spin = 1

Imaginary frequency: -320.8779

C 2.74686 1.907856 1.96602

C 1.727994 2.599058 1.300248

C 0.651715 1.847244 0.883452

C 0.534982 0.462699 1.126084

C 1.555938 -0.208365 1.836011

C 2.638394 0.525056 2.261422

H 1.807259 3.653593 1.072751

H 3.456069 0.050943 2.788443

C -0.669376 0.03428 0.524907

C -1.17243 1.191063 -0.288111

O -0.376828 2.339044 0.172137

C -2.594659 1.656132 -0.404166

C -3.323689 1.375179 -1.555384

C -3.211787 2.333115 0.650567

C -4.655157 1.760541 -1.66213

H -2.847242 0.838792 -2.368486

C -4.54108 2.716552 0.559872

H -2.650192 2.5537 1.553119

C -5.265906 2.428585 -0.600418

H -5.224037 1.541376 -2.560978

H -5.029992 3.238224 1.375625

C -1.310998 -1.281068 0.609076

C -2.710117 -1.334615 0.569601

C -0.541168 -2.456637 0.722312

C -3.359903 -2.561629 0.616661

H -3.299563 -0.428782 0.521759

C -1.234542 -3.678064 0.730523

C -2.623996 -3.738433 0.683544

H -3.132982 -4.697225 0.704021

C 1.431667 -1.685642 2.094931

H 0.646195 -1.8194 2.853207

C 0.972381 -2.482936 0.844144

H 1.227193 -3.516479 1.095129

C 1.710093 -2.155948 -0.452434

C 3.078803 -1.856126 -0.450073

C 1.065138 -2.223784 -1.688149

C 3.770439 -1.614114 -1.631104

H 3.617883 -1.815918 0.48939

C 1.744386 -1.99039 -2.880157

H 0.005764 -2.455134 -1.732336

C 3.101607 -1.680347 -2.853662

H 4.829194 -1.377251 -1.614299

H 1.219729 -2.04195 -3.830082

O 2.66103 -2.160837 2.608663

O 3.822823 -1.430527 -3.987774

O -0.497854 -4.82259 0.784653

O -4.716535 -2.67109 0.59414

O -6.569908 2.824407 -0.638738

O -0.620161 0.563645 -1.352207

H 3.242772 -1.505496 -4.7551

H -1.086721 -5.586532 0.76907

H -5.110838 -1.792465 0.527943

H -6.958927 2.567673 -1.483769

H 2.525313 -3.074504 2.883353

O 3.875499 2.514326 2.340637

O 2.022653 1.258474 -1.846678

H 2.479625 0.648193 -1.254277

H 1.073709 1.059783 -1.695657

H 2.622315 2.906346 -1.501933

O 3.012014 3.808007 -1.482568

H 3.735172 3.759942 -2.117132

H 3.974357 3.38461 1.855649

O 4.188317 4.640602 0.834433

H 3.77341 4.318533 -0.002299

H 3.6026 5.346163 1.13177

INT3.log

Energy (E) = -1909.61441157 Hartree

Enthalpy (H) = -1909.070791 Hartree

Gibbs free energy (G) = -1909.177541 Hartree

Charge = 0, Spin = 1

C 1.986372 2.522439 1.677984

C 0.907627 2.954811 0.91324

C -0.005358 1.992547 0.515236

C 0.116261 0.60347 0.909461

C 1.170611 0.233249 1.820318

C 2.070153 1.176633 2.188779

H 0.826893 3.974512 0.56385

H 2.897183 0.9399 2.845304

C -0.819302 -0.124801 0.235858

C -1.628764 0.793658 -0.720897

O -0.993487 2.224931 -0.288569

C -3.090431 0.99979 -0.351824

C -4.042879 1.07909 -1.363223

C -3.503246 1.11218 0.978457

C -5.387931 1.264606 -1.062698

H -3.72532 0.984186 -2.396011

C -4.841105 1.296072 1.294213

H -2.772935 1.05314 1.781061

C -5.787806 1.372244 0.269211

H -6.128914 1.323798 -1.854982

H -5.166014 1.380638 2.325772

C -1.012581 -1.575963 0.151569

C -2.231321 -2.025541 -0.387147

C -0.006459 -2.507116 0.500166

C -2.465028 -3.378613 -0.580436

H -3.009816 -1.324579 -0.656212

C -0.264998 -3.864848 0.241026

C -1.476151 -4.306288 -0.277539

H -1.650359 -5.364184 -0.447895

C 1.238214 -1.183974 2.294826

H 0.301587 -1.408188 2.826224

C 1.340723 -2.179882 1.109477

H 1.672292 -3.103499 1.590304

C 2.419976 -1.80524 0.102876

C 3.7643 -1.970483 0.455858

C 2.13742 -1.276194 -1.157458

C 4.792108 -1.609811 -0.405084

H 4.011561 -2.388182 1.426316

C 3.156838 -0.912813 -2.034497

H 1.110417 -1.136241 -1.481092

C 4.488401 -1.076324 -1.65753

H 5.830372 -1.745053 -0.120413

H 2.915481 -0.497148 -3.008583

O 2.336164 -1.309303 3.173244

O 5.530662 -0.740671 -2.475485

O 0.726039 -4.757017 0.519378

O -3.638672 -3.855253 -1.078772

O -7.09245 1.549096 0.628101

O -1.340898 0.579444 -1.963416

H 5.184198 -0.395706 -3.307283

H 0.414884 -5.650685 0.331752

H -4.238466 -3.119722 -1.253872

H -7.639177 1.581395 -0.166536

H 2.31794 -2.200834 3.53875

O 2.998368 3.312861 1.972277

O 1.143404 1.659897 -2.431492

H 1.682704 1.230203 -1.75713

H 0.242558 1.283197 -2.284171

H 1.440548 3.39728 -2.03342

O 1.681049 4.34502 -1.944626

H 2.255451 4.512473 -2.699311

H 2.984018 4.134239 1.382521

O 3.031663 5.25053 0.252201

H 2.581236 4.877201 -0.54409

H 2.45307 5.968182 0.535095

INT2'_2.log

Energy (E) = -1909.61670949 Hartree

Enthalpy (H) = -1909.071550 Hartree

Gibbs free energy (G) = -1909.178535 Hartree

Charge = 0, Spin = 1

C 3.751263 0.552423 0.139407

C 2.839752 1.506271 -0.328506

C 1.515086 1.28989 -0.011389

C 1.070018 0.211971 0.743957

C 1.968773 -0.783966 1.108612

C 3.313216 -0.603047 0.808181

H 3.157597 2.372026 -0.894718

H 4.045268 -1.349184 1.090681

C -0.377007 0.39753 0.976993

C -0.681677 1.640996 0.210053

O 0.48935 2.11161 -0.415032

C -1.916544 2.018551 -0.514709

C -2.080091 1.583639 -1.831058

C -2.91012 2.791062 0.084662

C -3.226683 1.909506 -2.540911

H -1.306321 0.984005 -2.301917

C -4.060752 3.122998 -0.617099

H -2.784277 3.132245 1.107168

C -4.22 2.680664 -1.931868

H -3.358759 1.568254 -3.563381

H -4.839517 3.723245 -0.159414

C -1.369218 -0.665503 1.322168

C -2.623926 -0.238938 1.766282

C -1.083939 -2.033879 1.168015

C -3.628102 -1.164248 2.019116

H -2.821104 0.814251 1.921717

C -2.137118 -2.936932 1.410338

C -3.395349 -2.520591 1.829093

H -4.184061 -3.242494 2.01623

C 1.416391 -2.051177 1.698923

H 0.974647 -1.832732 2.680674

C 0.268214 -2.618128 0.798004

H 0.220947 -3.673495 1.077828

C 0.58708 -2.567209 -0.692025

C 1.669092 -3.30062 -1.193758

C -0.140642 -1.788767 -1.593539

C 2.025036 -3.245682 -2.534985

H 2.25261 -3.9168 -0.517864

C 0.199845 -1.724031 -2.941819

H -0.997203 -1.214943 -1.254273

C 1.290447 -2.450057 -3.414946

H 2.867742 -3.816967 -2.910601

H -0.381812 -1.109968 -3.624318

O 2.471977 -2.980205 1.848038

O 1.679104 -2.425901 -4.726714

O -1.881628 -4.263897 1.219608

O -4.866291 -0.791506 2.4512

O -5.366681 3.0297 -2.578638

O -0.62313 1.715974 1.61329

H 1.091163 -1.842055 -5.220948

H -2.66323 -4.778521 1.453502

H -4.897775 0.169894 2.528605

H -5.353867 2.669815 -3.474291

H 2.117339 -3.747468 2.310353

O 5.081648 0.692544 -0.066914

O 1.958837 2.647752 2.827946

H 2.419683 1.806052 2.726777

H 1.085709 2.474542 2.441213

H 2.902945 3.706258 1.698456

O 3.403899 4.347999 1.151694

H 3.809991 4.938469 1.795236

H 5.2818 1.650979 -0.235139

O 5.516003 3.313803 -0.254058

H 4.761316 3.662811 0.276654

H 5.31619 3.592465 -1.154746

TS3'.log

Energy (E) = -1909.60336588 Hartree

Enthalpy (H) = -1909.060008 Hartree

Gibbs free energy (G) = -1909.165853 Hartree

Charge = 0, Spin = 1

Imaginary frequency: -356.8481

C 3.787371 0.415086 -0.008047

C 2.916172 1.401667 -0.486308

C 1.587167 1.257363 -0.153551

C 1.099054 0.175054 0.604091

C 1.981576 -0.843194 1.017444

C 3.313279 -0.719476 0.699142

H 3.273992 2.257249 -1.042852

H 4.029757 -1.474388 0.996185

C -0.281046 0.398029 0.817165

C -0.56937 1.757879 0.286256

O 0.625204 2.143971 -0.473147

C -1.774003 2.103766 -0.533378

C -1.995573 1.444503 -1.742392

C -2.679068 3.069067 -0.101601

C -3.112973 1.738683 -2.511356

H -1.291528 0.69009 -2.083705

C -3.798898 3.376533 -0.863786

H -2.509937 3.575695 0.843301

C -4.017759 2.707818 -2.069306

H -3.290624 1.221509 -3.449839

H -4.51073 4.125834 -0.534424

C -1.302764 -0.504525 1.367507

C -2.513382 0.04807 1.806689

C -1.106678 -1.901229 1.355401

C -3.548804 -0.779169 2.215604

H -2.633362 1.121818 1.861505

C -2.197702 -2.70353 1.736417

C -3.401786 -2.160991 2.168629

H -4.219756 -2.805624 2.474469

C 1.405232 -2.038704 1.718478

H 1.040315 -1.719095 2.705052

C 0.174696 -2.600075 0.942021

H 0.08537 -3.625489 1.309558

C 0.374057 -2.681901 -0.570331

C 1.490918 -3.347152 -1.092236

C -0.521569 -2.112653 -1.476317

C 1.71545 -3.425348 -2.459851

H 2.204703 -3.80107 -0.413607

C -0.312969 -2.184015 -2.851601

H -1.406489 -1.596302 -1.118808

C 0.811375 -2.839458 -3.346872

H 2.587138 -3.940013 -2.85082

H -1.024247 -1.730999 -3.536928

O 2.422799 -3.006104 1.87915

O 1.076027 -2.943725 -4.684909

O -2.035553 -4.05546 1.67036

O -4.737195 -0.290562 2.666763

O -5.13557 3.03699 -2.778039

O -0.503367 2.15124 1.585825

H 0.375293 -2.505772 -5.182926

H -2.835855 -4.492867 1.98517

H -4.715044 0.674059 2.645165

H -5.175239 2.504311 -3.581877

H 2.062237 -3.720669 2.415793

O 5.106641 0.498246 -0.199457

O 2.056941 2.697226 2.58892

H 2.458773 1.820977 2.566377

H 1.160545 2.553172 2.219652

H 3.114529 3.630199 1.474712

O 3.705426 4.223895 0.961607

H 4.174645 4.725274 1.637028

H 5.355448 1.438657 -0.434084

O 5.682132 3.041788 -0.510693

H 4.974449 3.450016 0.044168

H 5.475727 3.330788 -1.406786

INT3'.log

Energy (E) = -1909.61562414 Hartree

Enthalpy (H) = -1909.072466 Hartree

Gibbs free energy (G) = -1909.180120 Hartree

Charge = 0, Spin = 1

C 3.828972 -0.622883 -0.295284

C 3.259817 0.541963 -0.801797

C 1.968632 0.834495 -0.398032

C 1.210385 -0.056363 0.458445

C 1.79693 -1.312447 0.856925

C 3.070856 -1.575099 0.477968

H 3.824627 1.236055 -1.408143

H 3.568815 -2.490132 0.771497

C 0.052654 0.568313 0.808711

C 0.023061 2.011372 0.242996

O 1.345128 1.931645 -0.695409

C -1.100874 2.230456 -0.754864

C -1.335669 1.328355 -1.793621

C -1.937065 3.335258 -0.617927

C -2.387442 1.519282 -2.679541

H -0.692807 0.461586 -1.91695

C -2.992712 3.542337 -1.49717

H -1.75785 4.036407 0.190252

C -3.21925 2.631805 -2.529637

H -2.566239 0.81288 -3.485541

H -3.647552 4.401091 -1.393006

C -1.063551 0.088755 1.622334

C -1.908138 1.053386 2.194071

C -1.337437 -1.288323 1.770667

C -3.030899 0.661271 2.908314

H -1.672576 2.105956 2.095946

C -2.499415 -1.642435 2.474277

C -3.336593 -0.688382 3.043835

H -4.22464 -0.992187 3.589475

C 0.985666 -2.258873 1.693179

H 0.93 -1.830751 2.705487

C -0.47606 -2.393159 1.192034

H -0.824192 -3.319431 1.657503

C -0.636211 -2.56327 -0.318965

C 0.275873 -3.325289 -1.060457

C -1.719422 -2.007568 -1.000664

C 0.128708 -3.501512 -2.429782

H 1.118882 -3.788921 -0.56073

C -1.886922 -2.182939 -2.371868

H -2.450151 -1.410484 -0.464583

C -0.955642 -2.925799 -3.092821

H 0.848519 -4.086097 -2.993415

H -2.735027 -1.733252 -2.880958

O 1.656672 -3.501916 1.743155

O -1.054655 -3.125538 -4.442547

O -2.794191 -2.968603 2.581233

O -3.876762 1.556244 3.489351

O -4.269028 2.875691 -3.367115

O 0.226951 2.927588 1.134895

H -1.843451 -2.680235 -4.775377

H -3.605885 -3.077265 3.091313

H -3.57106 2.453225 3.305357

H -4.316658 2.178227 -4.032115

H 1.194874 -4.055821 2.382567

O 5.098214 -0.921524 -0.484262

O 2.736836 2.53533 2.142102

H 2.774756 1.587389 2.308928

H 1.818911 2.684725 1.805399

H 4.063057 2.833484 0.960666

O 4.836112 3.109144 0.420403

H 5.428836 3.523882 1.056005

H 5.606704 -0.105066 -0.79398

O 6.347886 1.296725 -0.962246

H 5.807175 1.927918 -0.427885

H 6.22811 1.592295 -1.872153

INT3_2.log

Energy (E) = -1909.61441172 Hartree

Enthalpy (H) = -1909.070787 Hartree

Gibbs free energy (G) = -1909.177523 Hartree

Charge = 0, Spin = 1

C -1.985739 2.523016 -1.677808

C -0.906987 2.955148 -0.913057

C 0.005853 1.992704 -0.514939

C -0.116074 0.60359 -0.909183

C -1.170498 0.233622 -1.820112

C -2.069816 1.177196 -2.188582

H -0.826038 3.974817 -0.563624

H -2.896866 0.94069 -2.845163

C 0.819233 -0.124999 -0.235594

C 1.629118 0.792922 0.721262

O 0.993847 2.224973 0.288886

C 3.090684 0.999083 0.351948

C 4.043168 1.079041 1.363256

C 3.503411 1.110804 -0.978426

C 5.388183 1.264567 1.062565

H 3.725676 0.984659 2.396115

C 4.841225 1.294711 -1.294341

H 2.773069 1.0512 -1.780956

C 5.78797 1.371545 -0.26942

H 6.129201 1.324272 1.854776

H 5.166083 1.378772 -2.325957

C 1.012162 -1.576248 -0.151411

C 2.230766 -2.026137 0.387361

C 0.005912 -2.507172 -0.50024

C 2.464195 -3.379283 0.580506

H 3.009398 -1.325375 0.65655

C 0.26419 -3.864989 -0.241315

C 1.475193 -4.306729 0.277338

H 1.649186 -5.364678 0.447579

C -1.23835 -1.183542 -2.294772

H -0.301716 -1.407878 -2.826109

C -1.341166 -2.179594 -1.109562

H -1.672882 -3.103086 -1.590531

C -2.420425 -1.804891 -0.102999

C -3.764747 -1.969631 -0.456192

C -2.137864 -1.276352 1.157557

C -4.792555 -1.608939 0.404748

H -4.012013 -2.386925 -1.426826

C -3.157278 -0.912992 2.034605

H -1.110846 -1.136785 1.481327

C -4.488843 -1.075978 1.65741

H -5.830823 -1.743752 0.119889

H -2.915911 -0.497695 3.008842

O -2.336232 -1.308553 -3.173318

O -5.531111 -0.740323 2.475356

O -0.726969 -4.756949 -0.519943

O 3.637693 -3.856223 1.078901

O 7.092577 1.548321 -0.628471

O 1.341315 0.578884 1.963678

H -5.184657 -0.395552 3.307239

H -0.415863 -5.650728 -0.332753

H 4.237331 -3.120784 1.254912

H 7.63931 1.581346 0.166131

H -2.318513 -2.200254 -3.538438

O -2.99754 3.313681 -1.972281

O -1.143632 1.659485 2.431406

H -1.682492 1.229995 1.756564

H -0.242747 1.282911 2.284404

H -1.439311 3.398033 2.033445

O -1.678663 4.346028 1.944952

H -2.256 4.512973 2.697516

H -2.982932 4.135185 -1.382743

O -3.030441 5.251664 -0.252234

H -2.579674 4.878223 0.543726

H -2.452243 5.969676 -0.535005

TS4.log

Energy (E) = -1909.60563552 Hartree

Enthalpy (H) = -1909.066423 Hartree

Gibbs free energy (G) = -1909.169121 Hartree

Charge = 0, Spin = 1

Imaginary frequency: -565.5422

C -2.018725 2.528986 -1.773812

C -0.875289 2.96911 -1.046109

C 0.013136 2.024078 -0.622154

C -0.107622 0.615318 -0.963603

C -1.164539 0.219096 -1.869837

C -2.065853 1.145595 -2.258459

H -0.794527 3.99995 -0.72541

H -2.891929 0.884923 -2.908729

C 0.820297 -0.081818 -0.261859

C 1.608174 0.889397 0.662897

O 1.022082 2.258793 0.184552

C 3.088352 1.045245 0.320819

C 4.027315 1.138153 1.343195

C 3.52615 1.109621 -1.004723

C 5.381293 1.287973 1.059434

H 3.692445 1.081359 2.373291

C 4.872416 1.256778 -1.304832

H 2.806825 1.039749 -1.816365

C 5.804682 1.346073 -0.267965

H 6.110445 1.357547 1.861949

H 5.214803 1.302913 -2.333296

C 1.01858 -1.534312 -0.125995

C 2.229562 -1.96435 0.442381

C 0.025535 -2.481729 -0.464956

C 2.468248 -3.311232 0.674

H 2.998789 -1.252021 0.707854

C 0.289458 -3.831999 -0.172277

C 1.493294 -4.254808 0.377244

H 1.671675 -5.306883 0.576363

C -1.212298 -1.211873 -2.306128

H -0.267292 -1.442334 -2.819878

C -1.31794 -2.177646 -1.095662

H -1.642383 -3.115593 -1.553337

C -2.405302 -1.782766 -0.105554

C -3.74834 -1.922675 -0.474693

C -2.129148 -1.266471 1.16117

C -4.779759 -1.548981 0.376411

H -3.991548 -2.32996 -1.450434

C -3.152422 -0.890541 2.029126

H -1.102816 -1.145933 1.494854

C -4.481978 -1.028468 1.635693

H -5.816784 -1.664473 0.078731

H -2.915965 -0.486391 3.009339

O -2.297004 -1.375874 -3.196419

O -5.528064 -0.679753 2.443782

O -0.692035 -4.738193 -0.446639

O 3.63788 -3.766882 1.204587

O 7.119022 1.486044 -0.612642

O 1.31007 0.712764 1.925265

H -5.184987 -0.347993 3.282293

H -0.375984 -5.624665 -0.235372

H 4.231764 -3.023266 1.363704

H 7.655254 1.531068 0.18834

H -2.27332 -2.283255 -3.519958

O -3.008082 3.289589 -1.986377

O -1.147221 1.693883 2.304684

H -1.67303 1.200498 1.663245

H -0.218546 1.346 2.175112

H -1.433405 3.22246 1.947499

O -1.656414 4.199062 1.815108

H -2.009293 4.508853 2.659138

H -3.162071 4.228075 -0.749769

O -3.288248 4.734017 0.124891

H -2.491484 4.427245 0.938946

H -3.156186 5.669989 -0.076962

PD1_2.log

Energy (E) = -1909.63171862 Hartree

Enthalpy (H) = -1909.087093 Hartree

Gibbs free energy (G) = -1909.193867 Hartree

Charge = 0, Spin = 1

C -2.005587 2.613346 -1.881739

C -0.985278 3.005784 -0.942073

C -0.156674 2.04266 -0.482583

C -0.200458 0.655801 -0.910779

C -1.159709 0.287968 -1.933192

C -1.994518 1.236488 -2.401964

H -0.954017 4.020105 -0.565888

H -2.737553 1.001755 -3.154403

C 0.724954 -0.038035 -0.205526

C 1.448637 0.956516 0.72911

O 0.792382 2.232598 0.437645

C 2.921992 1.164872 0.432831

C 3.852561 1.261296 1.46398

C 3.352659 1.306305 -0.888893

C 5.196717 1.483317 1.185322

H 3.531257 1.154005 2.493192

C 4.689175 1.531069 -1.178831

H 2.638367 1.236868 -1.704276

C 5.616579 1.618775 -0.137527

H 5.921058 1.55424 1.991624

H 5.027079 1.637976 -2.203897

C 1.026868 -1.475565 -0.177261

C 2.248635 -1.878889 0.384267

C 0.11197 -2.443352 -0.649687

C 2.579973 -3.224754 0.462369

H 2.959733 -1.153349 0.755032

C 0.472565 -3.795468 -0.525988

C 1.692619 -4.192246 0.009916

H 1.94832 -5.244543 0.083559

C -1.173061 -1.135405 -2.400709

H -0.213686 -1.328642 -2.903308

C -1.25927 -2.131857 -1.214721

H -1.603322 -3.057309 -1.684294

C -2.285836 -1.780556 -0.136698

C -3.537773 -1.252922 -0.479925

C -2.039455 -2.032018 1.21456

C -4.492151 -0.961612 0.485182

H -3.772417 -1.06636 -1.521473

C -2.992083 -1.758308 2.193687

H -1.085334 -2.444654 1.52539

C -4.219845 -1.211672 1.830326

H -5.4536 -0.543755 0.204925

H -2.773653 -1.955153 3.239219

O -2.231301 -1.301623 -3.322767

O -5.189584 -0.905428 2.744415

O -0.42943 -4.728351 -0.944307

O 3.763414 -3.655957 0.980736

O 6.920012 1.836125 -0.471082

O 1.248847 0.660502 2.059022

H -4.867317 -1.115518 3.629112

H -0.066391 -5.612553 -0.813115

H 4.296595 -2.896271 1.24527

H 7.452906 1.875367 0.332723

H -2.165339 -2.193146 -3.6827

O -2.907285 3.40047 -2.244966

O -1.381842 0.992147 2.364783

H -1.772533 0.508466 1.626581

H 0.268676 0.7168 2.226525

H -1.569722 1.941434 2.178734

O -1.895742 3.66095 2.070709

H -2.406498 3.83056 2.869631

H -3.312323 4.399971 -0.775627

O -3.492539 4.812847 0.09326

H -2.482649 3.956684 1.342433

H -2.988338 5.633578 0.064739

INT3'_2.log

Energy (E) = -1909.61606952 Hartree

Enthalpy (H) = -1909.072440 Hartree

Gibbs free energy (G) = -1909.178692 Hartree

Charge = 0, Spin = 1

C 3.812575 0.608446 0.975811

C 3.316726 -0.679636 0.800392

C 2.067613 -0.793174 0.213249

C 1.283257 0.369555 -0.162141

C 1.761051 1.681196 0.207646

C 2.997712 1.777656 0.753399

H 3.909392 -1.555791 1.02353

H 3.413722 2.73675 1.034159

C 0.234836 -0.051621 -0.920466

C 0.260553 -1.59048 -1.092194

O 1.520838 -1.920224 -0.114677

C -0.913367 -2.252716 -0.386462

C -1.243637 -1.939023 0.933743

C -1.6938 -3.178707 -1.074639

C -2.324036 -2.544179 1.561217

H -0.654783 -1.213875 1.4897

C -2.781115 -3.789724 -0.46225

H -1.443376 -3.420903 -2.101951

C -3.095091 -3.473542 0.859831

H -2.573511 -2.29616 2.589468

H -3.391433 -4.510473 -0.996294

C -0.803878 0.736896 -1.582307

C -1.418647 0.180117 -2.714092

C -1.191529 2.002708 -1.097536

C -2.405443 0.888512 -3.385502

H -1.101942 -0.78969 -3.078263

C -2.207147 2.677764 -1.792538

C -2.805206 2.138871 -2.927761

H -3.585643 2.684218 -3.449347

C 0.892922 2.895271 0.000824

H 1.035191 3.221953 -1.040198

C -0.61988 2.628456 0.162137

H -1.049175 3.632187 0.221378

C -1.06935 1.879682 1.418196

C -0.251323 1.669973 2.531483

C -2.380643 1.396895 1.480075

C -0.70231 0.956969 3.638804

H 0.76076 2.056429 2.550167

C -2.853564 0.702664 2.585491

H -3.052089 1.551094 0.640943

C -2.003229 0.459658 3.66272

H -0.047956 0.782551 4.486563

H -3.871858 0.324953 2.603309

O 1.343661 3.906684 0.882471

O -2.394818 -0.261961 4.757887

O -2.606674 3.887598 -1.308727

O -3.021422 0.404325 -4.499593

O -4.169959 -4.098556 1.423175

O 0.573338 -2.013107 -2.273675

H -3.312585 -0.537589 4.643836

H -3.326342 4.233893 -1.849919

H -2.661517 -0.4661 -4.710445

H -4.27538 -3.789587 2.331113

H 0.946224 4.736284 0.595543

O 5.05034 0.838227 1.362369

O 3.073333 -1.047488 -2.791184

H 3.021101 -0.135067 -2.486799

H 2.164023 -1.404111 -2.63481

H 4.374985 -1.793236 -1.80584

O 5.154574 -2.248921 -1.417572

H 5.810587 -2.225434 -2.122425

H 5.611315 0.003473 1.251713

O 6.453238 -1.252429 0.768334

H 5.980723 -1.577117 -0.036725

H 6.323681 -1.952774 1.418346

TS4'.log

Energy (E) = -1909.60718915 Hartree

Enthalpy (H) = -1909.068869 Hartree

Gibbs free energy (G) = -1909.171013 Hartree

Charge = 0, Spin = 1

Imaginary frequency: -503.7129

C 3.825597 -0.722642 -1.110581

C 3.242377 0.576603 -1.174024

C 2.045179 0.762646 -0.546365

C 1.312742 -0.322199 0.093084

C 1.802659 -1.67713 -0.065278

C 3.008355 -1.849665 -0.648576

H 3.803945 1.409594 -1.577944

H 3.432047 -2.838527 -0.776471

C 0.307881 0.219474 0.820021

C 0.325809 1.763259 0.689298

O 1.456459 1.925983 -0.382572

C -0.931409 2.269758 -0.010956

C -1.335064 1.745944 -1.240717

C -1.711302 3.256704 0.586203

C -2.485944 2.202399 -1.869444

H -0.748371 0.96692 -1.72134

C -2.868886 3.721833 -0.02728

H -1.402519 3.664054 1.542996

C -3.25575 3.194852 -1.259511

H -2.790839 1.786873 -2.826169

H -3.47796 4.490234 0.437506

C -0.668867 -0.446493 1.687381

C -1.213034 0.294279 2.74582

C -1.062517 -1.780604 1.463377

C -2.14073 -0.290603 3.597638

H -0.886382 1.31426 2.909828

C -2.009752 -2.332284 2.340268

C -2.544554 -1.606011 3.39992

H -3.274619 -2.057362 4.064406

C 0.966362 -2.841561 0.404127

H 1.177141 -2.985268 1.474456

C -0.559203 -2.615883 0.299244

H -0.974147 -3.616946 0.445833

C -1.0993 -2.10853 -1.038638

C -0.354989 -2.099286 -2.221285

C -2.420647 -1.655005 -1.103239

C -0.885892 -1.601777 -3.408129

H 0.660953 -2.476101 -2.230736

C -2.973346 -1.176034 -2.283708

H -3.035561 -1.658987 -0.208415

C -2.194559 -1.125428 -3.438272

H -0.288233 -1.579279 -4.313534

H -3.997967 -0.815957 -2.304296

O 1.375598 -3.990425 -0.316709

O -2.665524 -0.61389 -4.617743

O -2.41082 -3.616724 2.113736

O -2.691949 0.381385 4.64838

O -4.400104 3.680474 -1.826871

O 0.694433 2.404196 1.770456

H -3.576181 -0.320379 -4.4926

H -3.072587 -3.867292 2.769362

H -2.3394 1.279479 4.673853

H -4.546332 3.23909 -2.67236

H 0.981652 -4.756025 0.116242

O 5.034098 -0.931286 -1.423759

O 3.134262 1.51979 2.347693

H 3.069342 0.569576 2.199127

H 2.212591 1.864026 2.155091

H 4.220562 2.0817 1.319068

O 4.927431 2.519884 0.744631

H 5.494493 3.01267 1.351279

H 5.858072 0.310022 -0.98797

O 6.355746 1.100957 -0.580866

H 5.638789 1.753148 0.078207

H 6.657172 1.648028 -1.31871

PD1'_2.log

Energy (E) = -1909.63511222 Hartree

Enthalpy (H) = -1909.089836 Hartree

Gibbs free energy (G) = -1909.195560 Hartree

Charge = 0, Spin = 1

C 3.832658 0.796214 1.100343

C 3.283929 -0.537618 1.110445

C 2.105738 -0.726387 0.477657

C 1.344276 0.333299 -0.16101

C 1.813166 1.6982 -0.02087

C 3.002819 1.896988 0.582891

H 3.853286 -1.357808 1.528344

H 3.401538 2.896084 0.711026

C 0.325102 -0.237712 -0.842361

C 0.366359 -1.756515 -0.586255

O 1.503923 -1.910382 0.31982

C -0.879708 -2.238662 0.128023

C -1.176581 -1.745175 1.399348

C -1.751695 -3.147981 -0.468133

C -2.313399 -2.162867 2.076514

H -0.520617 -1.018502 1.871806

C -2.895065 -3.57068 0.19806

H -1.538155 -3.532345 -1.458622

C -3.175435 -3.081088 1.474095

H -2.539737 -1.768545 3.063136

H -3.575267 -4.280146 -0.260866

C -0.690066 0.381777 -1.698517

C -1.263553 -0.397739 -2.712619

C -1.080846 1.722299 -1.509329

C -2.218072 0.155341 -3.556865

H -0.949378 -1.423536 -2.858872

C -2.050258 2.24259 -2.381059

C -2.615224 1.477461 -3.396626

H -3.365307 1.904395 -4.054886

C 0.960671 2.8357 -0.522196

H 1.152607 2.937116 -1.60086

C -0.560901 2.595117 -0.380448

H -0.990346 3.587033 -0.546231

C -1.065748 2.117468 0.982642

C -0.308832 2.190644 2.155306

C -2.365767 1.611722 1.083745

C -0.804131 1.72126 3.36863

H 0.688761 2.612582 2.137241

C -2.883692 1.160637 2.290552

H -2.990951 1.551459 0.198198

C -2.090258 1.190842 3.435566

H -0.195535 1.761942 4.266071

H -3.891151 0.75746 2.339317

O 1.370732 4.014624 0.14505

O -2.524613 0.706275 4.640151

O -2.443795 3.534495 -2.192943

O -2.800227 -0.554626 -4.56363

O -4.308432 -3.528976 2.087038

O 0.620505 -2.519572 -1.703834

H -3.420549 0.362847 4.53678

H -3.113524 3.765464 -2.847908

H -2.453737 -1.455446 -4.563552

H -4.382631 -3.123601 2.95954

H 0.96156 4.758863 -0.310122

O 4.987142 1.040113 1.514444

O 2.904853 -1.513771 -2.646411

H 2.829053 -0.571272 -2.4591

H 1.447436 -2.160587 -2.127235

H 3.567658 -1.841531 -1.993454

O 4.719443 -2.608476 -0.930442

H 5.326493 -3.042108 -1.539909

H 6.002738 -0.400691 1.08242

O 6.457494 -1.188255 0.719842

H 5.29474 -2.02 -0.395889

H 6.431879 -1.821508 1.445779

PD1.log

Energy (E) = -1680.35096914 Hartree

Enthalpy (H) = -1679.890252 Hartree

Gibbs free energy (G) = -1679.980610 Hartree

Charge = 0, Spin = 1

C 1.675645 3.541017 1.23562

C 0.655506 3.549696 0.203001

C 0.046692 2.381609 -0.086168

C 0.337947 1.121216 0.576759

C 1.38142 1.098443 1.584659

C 2.000029 2.254528 1.889092

H 0.416975 4.473523 -0.309604

H 2.789184 2.284712 2.630877

C -0.476894 0.169941 0.067684

C -1.36601 0.831609 -0.997928

O -0.905186 2.215707 -1.018681

C -2.849258 0.876663 -0.681431

C -3.805967 0.537939 -1.637398

C -3.269071 1.288837 0.584328

C -5.160116 0.599831 -1.334548

H -3.496747 0.212653 -2.623434

C -4.618944 1.355402 0.897511

H -2.538329 1.558183 1.341481

C -5.569545 1.007349 -0.064901

H -5.906806 0.333195 -2.07469

H -4.939756 1.673079 1.885056

C -0.517848 -1.280115 0.305405

C -1.696886 -1.973102 -0.003701

C 0.622052 -1.977831 0.759222

C -1.758428 -3.352563 0.142354

H -2.577142 -1.448674 -0.351489

C 0.525743 -3.374188 0.87383

C -0.646804 -4.061453 0.579908

H -0.695407 -5.140874 0.682918

C 1.732474 -0.224155 2.197785

H 0.881222 -0.5499 2.813218

C 1.938765 -1.315644 1.105785

H 2.539104 -2.077447 1.60936

C 2.741883 -0.844065 -0.10656

C 3.979393 -0.215356 0.081372

C 2.299253 -1.020431 -1.418338

C 4.736563 0.235221 -0.991578

H 4.352508 -0.066606 1.088724

C 3.046639 -0.576026 -2.506127

H 1.350317 -1.509986 -1.61318

C 4.268275 0.057869 -2.29369

H 5.691795 0.724203 -0.830364

H 2.680109 -0.721613 -3.518429

O 2.87203 -0.057713 3.016418

O 2.284335 4.57054 1.56769

O 5.046635 0.519711 -3.319459

O 1.634346 -4.054183 1.282821

O -2.885384 -4.064394 -0.138277

O -6.908457 1.045219 0.184695

O -1.170899 0.27529 -2.251587

H -0.219566 0.184099 -2.400583

H 4.612047 0.326934 -4.159173

H 1.432659 -4.996452 1.332727

H -3.575672 -3.46462 -0.447105

H -7.058883 1.336338 1.092471

H 3.007982 -0.88561 3.490558

PD1'.log

Energy (E) = -1680.35529575 Hartree

Enthalpy (H) = -1679.894054 Hartree

Gibbs free energy (G) = -1679.983750 Hartree

Charge = 0, Spin = 1

C 3.922848 1.037247 1.88998

C 2.709407 1.474296 2.554145

C 1.561282 0.844964 2.232934

C 1.456584 -0.235549 1.264931

C 2.655304 -0.63232 0.549008

C 3.81593 -0.029719 0.873006

H 2.752002 2.270772 3.286916

H 4.734356 -0.284224 0.35784

C 0.176564 -0.672078 1.251584

C -0.631194 0.208591 2.222138

O 0.358616 1.121534 2.7639

C -1.6914 1.017975 1.501384

C -3.049965 0.760494 1.681715

C -1.301483 2.016536 0.608115

C -4.005568 1.496282 0.99309

H -3.368832 -0.017815 2.364998

C -2.247425 2.761154 -0.08191

H -0.24806 2.21673 0.432922

C -3.605294 2.499896 0.110382

H -5.063323 1.300413 1.132257

H -1.935546 3.530818 -0.78163

C -0.448955 -1.745864 0.468625

C -1.601887 -2.354682 0.984373

C 0.080396 -2.148971 -0.773739

C -2.230807 -3.369774 0.275167

H -2.000525 -2.053756 1.944519

C -0.585677 -3.176198 -1.461647

C -1.726872 -3.786389 -0.95079

H -2.224918 -4.576787 -1.503527

C 2.546867 -1.663604 -0.541619

H 2.452459 -2.647118 -0.057839

C 1.283541 -1.493116 -1.423804

H 1.510582 -2.0904 -2.310913

C 0.976286 -0.075182 -1.910067

C -0.324389 0.259121 -2.302985

C 1.960544 0.909033 -2.033179

C -0.644728 1.527891 -2.766622

H -1.115598 -0.480705 -2.233718

C 1.651246 2.193337 -2.472834

H 2.990139 0.683832 -1.782542

C 0.341731 2.509387 -2.828869

H -1.662053 1.773425 -3.052366

H 2.428227 2.949698 -2.540008

O 3.735621 -1.625863 -1.308236

O 5.028902 1.542899 2.13889

O -0.028455 3.760545 -3.242903

O -0.083181 -3.554587 -2.671509

O -3.349973 -3.995534 0.736417

O -4.582318 3.191868 -0.541918

O -1.206232 -0.467768 3.28766

H -0.551921 -1.076475 3.656071

H 0.741881 4.3413 -3.221687

H -0.62103 -4.266168 -3.039229

H -3.60699 -3.611572 1.583896

H -4.179513 3.851318 -1.119882

H 3.727495 -2.39458 -1.889567

INT4.log

Energy (E) = -1833.65811582 Hartree

Enthalpy (H) = -1833.128535 Hartree

Gibbs free energy (G) = -1833.233135 Hartree

Charge = 1, Spin = 1

C 1.730744 -3.107914 -1.146137

C 0.666071 -3.19535 -0.234091

C -0.022334 -2.033355 -0.002524

C 0.296942 -0.780628 -0.633756

C 1.388766 -0.732798 -1.568007

C 2.077872 -1.874936 -1.805313

H 0.415166 -4.124464 0.260528

H 2.910334 -1.896206 -2.496331

C -0.554428 0.169973 -0.151246

C -1.53596 -0.533654 0.802313

O -1.049001 -1.923459 0.824345

C -2.974992 -0.613706 0.305901

C -4.019843 -0.097767 1.072668

C -3.266566 -1.212115 -0.921674

C -5.333664 -0.185443 0.633305

H -3.809391 0.391598 2.01743

C -4.575379 -1.303136 -1.37251

H -2.471926 -1.616174 -1.541979

C -5.614647 -0.792173 -0.590806

H -6.146299 0.213836 1.230177

H -4.796693 -1.770664 -2.32727

C -0.543305 1.618924 -0.309762

C -1.707398 2.322228 0.043292

C 0.628287 2.315215 -0.693129

C -1.724656 3.708392 0.02589

H -2.611633 1.797068 0.317228

C 0.582815 3.71908 -0.645384

C -0.573225 4.412935 -0.305655

H -0.579637 5.498415 -0.296727

C 1.714517 0.578721 -2.207202

H 0.849721 0.881916 -2.815855

C 1.932236 1.684722 -1.137295

H 2.458005 2.466543 -1.690853

C 2.85743 1.252095 -0.00545

C 4.217267 1.060761 -0.279181

C 2.413871 1.006081 1.294754

C 5.100074 0.623721 0.698533

H 4.591209 1.253451 -1.279145

C 3.286891 0.571018 2.287958

H 1.371771 1.152195 1.559229

C 4.633393 0.374127 1.98953

H 6.152115 0.480359 0.474905

H 2.922181 0.386347 3.294392

O 2.849652 0.407405 -3.02545

O 2.473653 -4.139055 -1.453006

O 5.53975 -0.052581 2.919887

O 1.72482 4.39282 -0.94995

O -2.832139 4.431712 0.34045

O -6.917604 -0.855895 -0.977867

O -1.374349 0.00602 2.052044

H -1.875307 -0.554497 2.703499

H 5.094983 -0.173535 3.767538

H 1.565818 5.34347 -0.902054

H -3.556705 3.835444 0.567263

H -6.98043 -1.308435 -1.827927

H 2.977114 1.225836 -3.518137

O -2.674674 -1.650492 3.691434

H -3.611483 -1.486137 3.528608

H -2.502352 -2.480168 3.229329

O 1.790064 -6.324434 -0.234364

H 2.192474 -4.978701 -0.966221

H 1.873352 -6.163454 0.713782

H 0.841554 -6.440473 -0.371734

TS5.log

Energy (E) = -1833.62985698 Hartree

Enthalpy (H) = -1833.088766 Hartree

Gibbs free energy (G) = -1833.189793 Hartree

Charge = 1, Spin = 1

Imaginary frequency: -294.9921

C 0.81888 -3.510213 -0.13935

C -0.145037 -3.275303 0.848924

C -0.605298 -1.976576 0.93672

C -0.147286 -0.933423 0.143901

C 0.709606 -1.210868 -0.919905

C 1.215732 -2.491749 -1.033142

H -0.522159 -4.067649 1.483249

H 1.895717 -2.747385 -1.836067

C -0.905908 0.270339 0.484702

C -1.891778 -0.229913 1.432279

O -1.644309 -1.567893 1.733923

C -2.557474 0.010898 -0.101336

C -3.41355 1.138006 -0.188503

C -2.787705 -1.077188 -0.980118

C -4.460277 1.164355 -1.074676

H -3.265506 1.990579 0.461374

C -3.843107 -1.063692 -1.856117

H -2.135597 -1.942337 -0.977866

C -4.687823 0.059224 -1.914097

H -5.118056 2.02302 -1.137535

H -4.019356 -1.904013 -2.518945

C -0.359699 1.664393 0.408335

C -0.936234 2.619972 1.25981

C 0.733056 2.012437 -0.413476

C -0.47841 3.928642 1.279303

H -1.729146 2.342042 1.940195

C 1.168048 3.356055 -0.353366

C 0.573488 4.310152 0.458462

H 0.938713 5.33208 0.46962

C 0.938696 -0.119252 -1.921123

H -0.048644 0.184315 -2.303136

C 1.601634 1.126792 -1.297412

H 1.848275 1.743739 -2.16698

C 2.920526 0.769414 -0.624816

C 4.095705 0.720535 -1.379635

C 2.99697 0.438764 0.729396

C 5.307202 0.346971 -0.811419

H 4.063376 0.981904 -2.433261

C 4.201099 0.062321 1.315334

H 2.10503 0.477116 1.347473

C 5.36044 0.013739 0.541951

H 6.215522 0.315751 -1.404195

H 4.243551 -0.19074 2.371001

O 1.729566 -0.629917 -2.973784

O 1.37361 -4.721166 -0.319161

O 6.574777 -0.345361 1.05969

O 2.230205 3.697295 -1.134767

O -1.018794 4.876775 2.09347

O -5.715227 0.133448 -2.759432

O -2.499912 0.4236 2.412848

H -3.443642 0.071969 2.491067

H 6.47417 -0.548006 1.99766

H 2.438459 4.63009 -1.002559

H -1.735059 4.486437 2.609258

H -5.783808 -0.673945 -3.288874

H 1.819017 0.065275 -3.635199

O -4.902782 -0.533568 2.359896

H -5.228886 -0.220843 1.505836

H -4.761872 -1.479273 2.225578

O 0.401233 -6.532024 1.394762

H 1.007783 -5.377079 0.3278

H 0.542001 -6.157685 2.272784

H -0.555809 -6.493861 1.278409

PD2_2.log

Energy (E) = -1833.66253123 Hartree

Enthalpy (H) = -1833.132477 Hartree

Gibbs free energy (G) = -1833.234895 Hartree

Charge = 1, Spin = 1

C 1.870393 -2.860246 -0.576132

C 1.054338 -3.026503 0.554748

C 0.067583 -2.08045 0.713871

C -0.152768 -1.021323 -0.133527

C 0.66349 -0.859845 -1.238975

C 1.674413 -1.787251 -1.459959

H 1.190753 -3.846594 1.248517

H 2.345066 -1.680146 -2.303942

C -1.319229 -0.200655 0.347992

C -1.704278 -1.057512 1.575203

O -0.867668 -2.088 1.75545

C -2.516383 -0.24611 -0.625606

C -3.262312 0.888525 -0.944707

C -2.875157 -1.465546 -1.207455

C -4.334624 0.812705 -1.82541

H -3.01017 1.850163 -0.513075

C -3.947833 -1.555981 -2.083223

H -2.308937 -2.364883 -0.981616

C -4.682056 -0.410292 -2.396165

H -4.906583 1.699536 -2.076502

H -4.21513 -2.509742 -2.528713

C -0.945004 1.246099 0.735323

C -1.665567 1.817423 1.790206

C -0.007284 2.012316 0.013692

C -1.463825 3.139467 2.163439

H -2.414519 1.241993 2.318624

C 0.158275 3.355428 0.425265

C -0.5453 3.921383 1.47845

H -0.382721 4.956881 1.760142

C 0.480549 0.406419 -2.016978

H -0.583411 0.53459 -2.259833

C 0.9166 1.609021 -1.137078

H 0.905241 2.457093 -1.829638

C 2.355756 1.439329 -0.6673

C 3.413458 1.828239 -1.49274

C 2.665723 0.844445 0.55736

C 4.73715 1.622963 -1.121561

H 3.199026 2.301196 -2.446662

C 3.983718 0.630262 0.945471

H 1.86634 0.535835 1.22469

C 5.024001 1.017231 0.101255

H 5.552423 1.930008 -1.768527

H 4.207557 0.162818 1.9004

O 1.24584 0.339898 -3.202236

O 2.87954 -3.713726 -0.85656

O 6.34012 0.831186 0.427037

O 1.062616 4.107202 -0.26386

O -2.144399 3.72135 3.191009

O -5.742191 -0.430588 -3.253132

O -2.67471 -0.928841 2.305229

H -2.864865 -1.926168 3.349463

H 6.393978 0.398596 1.287712

H 1.066654 5.004074 0.091499

H -2.745363 3.077777 3.586228

H -5.879619 -1.329903 -3.574852

H 1.133339 1.173087 -3.673637

O -3.034274 -2.635377 4.097809

H -3.3504 -3.456758 3.685865

H -2.191981 -2.835682 4.539947

O 3.050259 -5.674146 0.982276

H 2.933732 -4.426722 -0.173864

H 3.100777 -5.216715 1.830157

H 2.174784 -6.079252 0.9907

PD2.log

Energy (E) = -1680.38012412 Hartree

Enthalpy (H) = -1679.919325 Hartree

Gibbs free energy (G) = -1680.009439 Hartree

Charge = 0, Spin = 1

C 1.455595 3.324121 -0.815638

C 0.717132 2.967905 -1.950888

C -0.147129 1.906728 -1.791441

C -0.307064 1.209486 -0.613368

C 0.436174 1.564811 0.495254

C 1.32294 2.632569 0.393897

H 0.824349 3.502218 -2.886504

H 1.934182 2.914041 1.245356

C -1.333718 0.124575 -0.786253

C -1.727624 0.395573 -2.266633

O -0.980098 1.416476 -2.789737

C -2.597449 0.37886 0.062575

C -3.236297 -0.632661 0.779991

C -3.133462 1.669365 0.113996

C -4.373943 -0.366755 1.533983

H -2.848319 -1.644622 0.763731

C -4.271801 1.946943 0.857323

H -2.657182 2.480051 -0.430461

C -4.896019 0.924321 1.574037

H -4.861597 -1.157032 2.094724

H -4.676373 2.954366 0.888356

C -0.780129 -1.300586 -0.578423

C -1.35731 -2.310759 -1.356415

C 0.176702 -1.621756 0.406329

C -0.99265 -3.640089 -1.195084

H -2.120977 -2.06767 -2.084103

C 0.514895 -2.988914 0.536077

C -0.046729 -3.99055 -0.242672

H 0.244328 -5.027385 -0.106681

C 0.330535 0.6603 1.690049

H -0.72994 0.496352 1.92436

C 0.959828 -0.709729 1.353588

H 0.977576 -1.227247 2.317029

C 2.409883 -0.553833 0.906758

C 2.753517 -0.385155 -0.438041

C 3.437388 -0.536673 1.850746

C 4.071993 -0.189832 -0.829508

H 1.978247 -0.40401 -1.1985

C 4.763974 -0.344213 1.477621

H 3.199898 -0.676953 2.901129

C 5.081998 -0.165675 0.132149

H 4.328319 -0.056418 -1.875453

H 5.552565 -0.33683 2.225009

O 1.004439 1.157583 2.829971

O 2.317726 4.369497 -0.952549

O 6.366628 0.02891 -0.298073

O 1.44248 -3.310484 1.482303

O -1.537929 -4.640801 -1.943736

O -6.013823 1.136412 2.327183

O -2.593993 -0.110485 -2.930994

H 6.95765 0.026648 0.464504

H 1.567648 -4.266727 1.48936

H -2.175519 -4.263403 -2.562062

H -6.271944 2.064192 2.263026

H 0.573413 1.980915 3.087471

H 2.772855 4.522406 -0.115284

INT4'.log

Energy (E) = -1833.66367070 Hartree

Enthalpy (H) = -1833.134371 Hartree

Gibbs free energy (G) = -1833.236288 Hartree

Charge = 1, Spin = 1

C 4.022166 0.870554 -0.500882

C 3.457258 -0.128322 -1.309045

C 2.092416 -0.097623 -1.428521

C 1.253645 0.882625 -0.78944

C 1.864694 1.857199 0.07607

C 3.216657 1.841788 0.190585

H 4.065527 -0.871285 -1.807931

H 3.729793 2.538534 0.840393

C -0.030392 0.661152 -1.199913

C -0.033254 -0.662445 -1.980401

O 1.388961 -0.956981 -2.151025

C -0.629053 -1.736459 -1.070705

C 0.171342 -2.514901 -0.234566

C -2.015784 -1.90789 -1.038085

C -0.39659 -3.457548 0.609938

H 1.248832 -2.397995 -0.233897

C -2.593162 -2.84201 -0.190802

H -2.65259 -1.310612 -1.68196

C -1.782301 -3.610968 0.645835

H 0.229589 -4.052741 1.267214

H -3.669229 -2.972824 -0.159186

C -1.23466 1.440192 -0.925671

C -2.256264 1.440291 -1.887455

C -1.358394 2.187116 0.264113

C -3.405107 2.19181 -1.676202

H -2.144998 0.873606 -2.802149

C -2.544076 2.91273 0.450089

C -3.55579 2.925679 -0.505802

H -4.461161 3.500737 -0.338257

C 1.005161 2.816879 0.851546

H 0.71433 3.619571 0.157166

C -0.314304 2.189407 1.363648

H -0.664285 2.908847 2.108789

C -0.212074 0.835347 2.067436

C 0.963098 0.382384 2.669711

C -1.351218 0.02783 2.177904

C 1.015477 -0.846369 3.324551

H 1.860944 0.988573 2.638611

C -1.321896 -1.185269 2.849664

H -2.285045 0.345796 1.724871

C -0.12818 -1.635584 3.410651

H 1.94529 -1.189591 3.769401

H -2.20912 -1.805638 2.918287

O 1.782279 3.356036 1.901571

O -0.136828 -2.860277 4.018229

O -2.684783 3.607283 1.611998

O -4.419718 2.241986 -2.581127

O -2.394083 -4.485622 1.490004

O -0.605882 -0.573649 -3.215822

H -0.797961 -1.491344 -3.546545

H 0.746812 -3.053919 4.353234

H -3.541972 4.049834 1.622154

H -4.197005 1.697544 -3.346567

H -1.731735 -4.899762 2.057567

H 1.298668 4.108758 2.260444

O 5.312339 0.966798 -0.305536

H 5.83391 0.252079 -0.793002

O 6.673528 -0.862324 -1.539833

H 7.078737 -1.416037 -0.861566

H 7.417212 -0.429979 -1.976854

O -1.05899 -3.075955 -4.023205

H -1.779417 -3.354853 -3.44401

H -0.286207 -3.521233 -3.653735

TS5'.log

Energy (E) = -1833.63948382 Hartree

Enthalpy (H) = -1833.103960 Hartree

Gibbs free energy (G) = -1833.203389 Hartree

Charge = 1, Spin = 1

Imaginary frequency: -160.8513

C 3.979149 1.015444 0.550879

C 3.649134 0.671736 -0.764049

C 2.304617 0.710423 -1.068192

C 1.295402 1.031894 -0.166084

C 1.636314 1.371608 1.146546

C 2.983643 1.366659 1.4812

H 4.399002 0.399742 -1.496011

H 3.293184 1.62969 2.484719

C 0.013266 0.906233 -0.880409

C 0.410827 0.513999 -2.225178

O 1.79244 0.404005 -2.3069

C -0.287198 -0.716191 -1.314542

C 0.526997 -1.761425 -0.808144

C -1.607761 -1.027622 -1.739087

C 0.068387 -3.051095 -0.777825

H 1.524151 -1.556211 -0.437256

C -2.076727 -2.312315 -1.696249

H -2.248774 -0.241755 -2.121218

C -1.243994 -3.333525 -1.200292

H 0.68647 -3.849772 -0.38252

H -3.083426 -2.55596 -2.013588

C -1.204596 1.671212 -0.483526

C -1.933462 2.328755 -1.475172

C -1.584462 1.720068 0.867489

C -3.083502 3.032707 -1.131781

H -1.616575 2.292895 -2.510033

C -2.756693 2.43594 1.170245

C -3.505438 3.081186 0.190379

H -4.406269 3.62607 0.455191

C 0.590545 1.802979 2.149494

H 0.406016 2.872173 1.971627

C -0.786226 1.110094 2.011249

H -1.307847 1.426475 2.919437

C -0.794234 -0.416135 2.052324

C 0.27745 -1.170114 2.538385

C -1.961349 -1.102785 1.700309

C 0.19573 -2.554941 2.657767

H 1.193545 -0.68083 2.845864

C -2.060472 -2.482319 1.818082

H -2.813959 -0.54837 1.318455

C -0.973089 -3.21569 2.290659

H 1.045159 -3.122388 3.027533

H -2.966461 -3.002156 1.523937

O 1.127882 1.63832 3.450726

O -1.102739 -4.576486 2.352816

O -3.151077 2.479291 2.474059

O -3.832903 3.692273 -2.057332

O -1.757611 -4.55817 -1.131975

O -0.181985 0.774755 -3.382833

H -0.114339 -0.045747 -3.961626

H -0.288404 -4.956553 2.703741

H -3.955948 3.005582 2.550101

H -3.440656 3.571125 -2.930985

H -1.124403 -5.172988 -0.734562

H 0.551658 2.111293 4.0611

O 5.253518 1.03024 0.986483

H 5.87755 0.773644 0.261162

O 6.997152 0.356923 -0.938083

H 7.42322 -0.446756 -0.61779

H 7.686146 1.029331 -0.878811

O -0.003332 -1.540194 -4.577587

H -0.867449 -1.93517 -4.403275

H 0.584096 -1.991696 -3.95705

PD2'_2.log

Energy (E) = -1833.65878310 Hartree

Enthalpy (H) = -1833.129342 Hartree

Gibbs free energy (G) = -1833.230341 Hartree

Charge = 1, Spin = 1

C 3.747695 -1.248557 1.238933

C 3.797963 -0.565382 0.016085

C 2.65072 0.10891 -0.324208

C 1.476575 0.149032 0.407279

C 1.474166 -0.452391 1.665844

C 2.61264 -1.160946 2.053543

H 4.682103 -0.557341 -0.609089

H 2.642625 -1.638768 3.024583

C 0.449759 0.913647 -0.400007

C 1.366806 1.500479 -1.482119

O 2.562412 0.899307 -1.473822

C -0.429608 0.009973 -1.315219

C -0.10744 -1.322027 -1.561505

C -1.504273 0.581815 -2.002049

C -0.862486 -2.083273 -2.446427

H 0.724519 -1.789875 -1.046714

C -2.26444 -0.166945 -2.886565

H -1.7612 1.623357 -1.839221

C -1.948519 -1.509041 -3.104608

H -0.619903 -3.12843 -2.614634

H -3.108542 0.27444 -3.405354

C -0.410029 1.893831 0.396748

C -0.497896 3.221458 -0.017455

C -1.155023 1.42748 1.499025

C -1.348018 4.108134 0.638673

H 0.072154 3.580978 -0.864094

C -2.018176 2.352017 2.117888

C -2.124826 3.675332 1.701326

H -2.799027 4.36133 2.204423

C 0.382828 -0.191272 2.680503

H 0.665503 0.75586 3.163942

C -1.0407 0.043724 2.138331

H -1.625515 0.125821 3.059055

C -1.685076 -1.109638 1.376814

C -1.18251 -2.411564 1.401097

C -2.891505 -0.892543 0.703513

C -1.84502 -3.457568 0.76281

H -0.261331 -2.630012 1.926928

C -3.563354 -1.922011 0.059718

H -3.308619 0.109753 0.665122

C -3.034514 -3.21258 0.083475

H -1.431904 -4.462298 0.785462

H -4.489192 -1.734443 -0.474337

O 0.411468 -1.221155 3.65507

O -3.720848 -4.194351 -0.580633

O -2.773837 1.912843 3.166171

O -1.45736 5.413376 0.263632

O -2.735994 -2.21283 -3.967109

O 1.099457 2.310085 -2.358861

H 2.119833 2.586333 -3.350649

H -3.244245 -5.027983 -0.488245

H -3.307164 2.642579 3.503614

H -0.8666 5.580071 -0.481115

H -2.429287 -3.127059 -4.005296

H -0.131747 -0.929167 4.395317

O 4.790952 -1.971373 1.699054

H 5.54733 -1.938197 1.061742

O 6.873602 -1.889188 0.003667

H 7.071365 -2.810289 -0.202326

H 7.616399 -1.603386 0.548302

O 2.844801 2.78774 -4.079426

H 3.321905 1.96354 -4.274395

H 3.488874 3.416986 -3.713607

PD2'.log

Energy (E) = -1680.37651693 Hartree

Enthalpy (H) = -1679.916280 Hartree

Gibbs free energy (G) = -1680.004463 Hartree

Charge = 0, Spin = 1

C -2.306181 3.806592 0.012921

C -2.331584 3.301218 -1.289731

C -1.903458 2.001984 -1.427272

C -1.436771 1.192734 -0.400213

C -1.507561 1.681355 0.902321

C -1.928342 3.002236 1.088533

H -2.683093 3.890957 -2.127059

H -1.990789 3.404439 2.09314

C -0.976448 -0.112151 -1.012481

C -1.573231 0.031833 -2.432038

O -1.944375 1.330532 -2.641264

C 0.532814 -0.128624 -1.388316

C 1.300305 1.034736 -1.415796

C 1.109098 -1.321021 -1.830087

C 2.623604 1.005617 -1.83697

H 0.877688 1.976888 -1.084061

C 2.430619 -1.363942 -2.25276

H 0.526458 -2.236313 -1.837201

C 3.195031 -0.19686 -2.24938

H 3.225199 1.908201 -1.833229

H 2.872945 -2.300681 -2.578999

C -1.357146 -1.370184 -0.230642

C -2.023801 -2.404395 -0.88181

C -0.968258 -1.500285 1.120571

C -2.297422 -3.59758 -0.217365

H -2.321417 -2.315576 -1.91692

C -1.243521 -2.729105 1.746292

C -1.896596 -3.774694 1.096956

H -2.093312 -4.706755 1.620132

C -1.345783 0.78828 2.112722

H -2.334496 0.328936 2.261022

C -0.368174 -0.39614 1.989948

H -0.385393 -0.812293 3.001562

C 1.097202 -0.056247 1.740455

C 1.620091 1.228492 1.909514

C 1.991775 -1.082778 1.428177

C 2.978312 1.485798 1.751564

H 0.966811 2.051507 2.171407

C 3.350287 -0.843522 1.263546

H 1.618859 -2.093629 1.289873

C 3.847141 0.450546 1.418188

H 3.368378 2.491234 1.872652

H 4.023679 -1.655094 1.002188

O -1.052519 1.598695 3.239901

O 5.171804 0.754929 1.248342

O -0.84564 -2.887102 3.042975

O -2.955058 -4.565712 -0.916156

O 4.505575 -0.177692 -2.63198

O -1.635974 -0.761728 -3.335642

H 5.649488 -0.041399 0.987086

H -1.108605 -3.761328 3.354005

H -3.080329 -5.337683 -0.350914

H 4.776004 -1.067507 -2.889125

H -1.177639 1.054138 4.024916

O -2.708641 5.095582 0.183344

H -2.683812 5.318569 1.122272

PD1_3.log

Energy (E) = -1756.77948020 Hartree

Enthalpy (H) = -1756.290623 Hartree

Gibbs free energy (G) = -1756.385131 Hartree

Charge = 0, Spin = 1

C 1.153513 3.847466 1.112984

C 0.197111 3.68812 0.036313

C -0.261652 2.444159 -0.218271

C 0.149417 1.251228 0.508279

C 1.115372 1.404493 1.579815

C 1.568341 2.641971 1.859442

H -0.129165 4.553177 -0.528015

H 2.285877 2.801497 2.655311

C -0.534663 0.193096 0.012697

C -1.482442 0.712796 -1.086605

O -1.15313 2.138256 -1.170047

C -2.959366 0.640229 -0.735364

C -3.906528 0.299977 -1.700536

C -3.389398 0.956896 0.553944

C -5.258685 0.264416 -1.384633

H -3.58769 0.051309 -2.705748

C -4.73776 0.927137 0.88086

H -2.668401 1.224834 1.320521

C -5.677092 0.578026 -0.091451

H -5.996774 -0.004283 -2.132711

H -5.065316 1.169876 1.887374

C -0.470392 -1.234115 0.353802

C -1.537761 -2.052827 -0.046137

C 0.630466 -1.792797 1.038826

C -1.536803 -3.40965 0.245896

H -2.385996 -1.644292 -0.578421

C 0.5979 -3.171489 1.307921

C -0.46998 -3.977853 0.929555

H -0.465557 -5.040338 1.151361

C 1.542461 0.183028 2.342646

H 0.687434 -0.127613 2.961691

C 1.876545 -1.023096 1.432697

H 2.435498 -1.689338 2.095598

C 2.792145 -0.741838 0.239371

C 3.716651 0.308626 0.237384

C 2.790371 -1.599495 -0.864834

C 4.578295 0.518678 -0.833068

H 3.77814 0.976419 1.088134

C 3.659434 -1.414273 -1.938212

H 2.097486 -2.433776 -0.903833

C 4.553174 -0.343362 -1.927887

H 5.280224 1.346037 -0.822497

H 3.638714 -2.096712 -2.78307

O 2.629392 0.531486 3.178077

O 1.627455 4.954378 1.418385

O 5.418472 -0.099978 -2.955657

O 1.672694 -3.71612 1.945811

O -2.559925 -4.230971 -0.120326

O -7.013444 0.525554 0.171832

O -1.250376 0.136704 -2.313481

H -0.292387 0.272225 -2.5283

H 5.292273 -0.767187 -3.641412

H 1.530031 -4.663069 2.062833

H -3.227761 -3.718726 -0.593247

H -7.168221 0.769718 1.092684

H 2.791117 -0.212958 3.768411

O 1.32926 0.676518 -2.813766

H 1.838233 -0.074333 -2.475396

H 1.537015 1.383632 -2.190519

TS6.log

Energy (E) = -1756.75634359 Hartree

Enthalpy (H) = -1756.252964 Hartree

Gibbs free energy (G) = -1756.343731 Hartree

Charge = 0, Spin = 1

Imaginary frequency: -273.2006

C 1.391937 2.895677 2.571162

C 0.485267 3.157684 1.489956

C 0.064567 2.133283 0.689668

C 0.475917 0.735641 0.938478

C 1.546575 0.503196 1.909181

C 1.931348 1.526692 2.694326

H 0.141268 4.171657 1.318627

H 2.726127 1.397614 3.419311

C -0.244949 -0.146518 0.209972

C -1.251011 0.455639 -0.772908

O -0.762798 2.27124 -0.312162

C -2.682626 0.56693 -0.338645

C -3.681634 0.633289 -1.312061

C -3.048351 0.599431 1.010237

C -5.017703 0.734224 -0.954645

H -3.403305 0.600029 -2.359608

C -4.380639 0.700818 1.38173

H -2.287843 0.541499 1.783026

C -5.369109 0.770267 0.396381

H -5.796467 0.784186 -1.708154

H -4.661583 0.724733 2.430405

C -0.148909 -1.623747 0.193394

C -1.319926 -2.359988 -0.03197

C 1.084559 -2.281819 0.349896

C -1.272459 -3.748163 -0.080189

H -2.275968 -1.865588 -0.158766

C 1.097997 -3.682663 0.284146

C -0.064673 -4.417886 0.075605

H -0.033094 -5.501853 0.02856

C 2.273857 -0.824874 1.963793

H 1.70414 -1.505407 2.612378

C 2.374302 -1.523328 0.58161

H 3.172857 -2.260916 0.69599

C 2.705287 -0.599769 -0.590195

C 3.37652 0.613473 -0.413406

C 2.219169 -0.895878 -1.865493

C 3.403562 1.572151 -1.420553

H 3.826063 0.850042 0.543518

C 2.257692 0.039787 -2.891103

H 1.72732 -1.845248 -2.049576

C 2.788494 1.298554 -2.636086

H 3.85422 2.544334 -1.251793

H 1.810672 -0.177813 -3.856544

O 3.551699 -0.599482 2.533297

O 1.774651 3.778629 3.36555

O 2.618177 2.347569 -3.522817

O 2.301833 -4.308565 0.424948

O -2.387062 -4.506762 -0.28261

O -6.691806 0.869705 0.701576

O -0.992384 0.303321 -2.004217

H -0.352668 1.533201 -2.668418

H 2.443073 1.997213 -4.40725

H 2.185429 -5.261964 0.333157

H -3.153619 -3.928533 -0.379638

H -6.799525 0.896217 1.660516

H 3.939583 -1.464573 2.70665

O -0.086905 2.522779 -2.722918

H 0.859952 2.606907 -2.988863

H -0.200243 2.744185 -1.742399

INT5.log

Energy (E) = -1756.76069866 Hartree

Enthalpy (H) = -1756.272867 Hartree

Gibbs free energy (G) = -1756.368079 Hartree

Charge = 0, Spin = 1

C -0.604593 3.123521 2.405324

C -1.064641 3.182546 1.034986

C -0.751237 2.200508 0.159294

C -0.103724 0.941314 0.618882

C 0.644259 1.050874 1.885212

C 0.355231 2.060239 2.727825

H -1.594261 4.065923 0.696315

H 0.856238 2.138969 3.685493

C -0.413191 -0.232557 0.004131

C -1.37809 -0.269615 -1.174927

O -1.113633 2.328821 -1.126178

C -2.820898 -0.113979 -0.92696

C -3.702426 -0.137086 -2.018499

C -3.338873 0.051153 0.364496

C -5.062234 0.010567 -1.830858

H -3.303358 -0.26596 -3.018706

C -4.701866 0.196992 0.564387

H -2.677685 0.058477 1.224804

C -5.566438 0.181094 -0.534506

H -5.749784 -0.000361 -2.669359

H -5.103631 0.322597 1.564942

C 0.059396 -1.600397 0.338331

C -0.837519 -2.65781 0.115013

C 1.364065 -1.862739 0.782178

C -0.438731 -3.971037 0.323107

H -1.855564 -2.471191 -0.209484

C 1.742233 -3.204644 0.95986

C 0.859611 -4.25346 0.734186

H 1.174156 -5.282282 0.878012

C 1.703991 0.046904 2.299021

H 1.217403 -0.689738 2.953978

C 2.331948 -0.763696 1.151808

H 3.188527 -1.257314 1.620107

C 2.889281 0.010469 -0.037089

C 3.191052 1.373683 0.013513

C 3.19958 -0.681501 -1.211896

C 3.775311 2.026127 -1.070387

H 2.979626 1.946866 0.909153

C 3.79536 -0.050163 -2.29701

H 2.976742 -1.742023 -1.283651

C 4.088525 1.312234 -2.227447

H 3.999575 3.086475 -1.021673

H 4.030017 -0.608557 -3.198698

O 2.684777 0.751056 3.044267

O -0.919526 3.972647 3.253599

O 4.670126 1.991853 -3.25957

O 3.022317 -3.447225 1.359382

O -1.276236 -5.027889 0.13017

O -6.906215 0.324345 -0.406331

O -0.923246 -0.525682 -2.287083

H 0.706626 0.630375 -2.64407

H 4.822414 1.383267 -3.992829

H 3.161791 -4.398693 1.437951

H -2.132288 -4.708387 -0.180687

H -7.139344 0.443537 0.523416

H 3.238785 0.091518 3.477412

O 0.845333 1.590087 -2.68266

H 1.622425 1.747095 -2.127809

H -0.380291 2.028054 -1.741224

PD1'_3.log

Energy (E) = -1756.78050832 Hartree

Enthalpy (H) = -1756.291249 Hartree

Gibbs free energy (G) = -1756.387110 Hartree

Charge = 0, Spin = 1

C 3.167399 2.202921 2.112291

C 1.800759 2.689629 2.147513

C 0.815921 1.813922 1.858477

C 1.036689 0.420666 1.510426

C 2.402068 -0.065219 1.444296

C 3.398391 0.790916 1.740076

H 1.604184 3.723682 2.403014

H 4.433591 0.473733 1.698815

C -0.163705 -0.171227 1.318851

C -1.25075 0.894178 1.53495

O -0.494636 2.10273 1.835748

C -2.056313 1.157747 0.2706

C -1.468863 1.818585 -0.808979

C -3.367598 0.696361 0.145794

C -2.174497 2.026246 -1.986565

H -0.445818 2.174455 -0.739826

C -4.084378 0.89784 -1.026322

H -3.841273 0.17781 0.972006

C -3.486061 1.56135 -2.097645

H -1.709132 2.541159 -2.821735

H -5.104385 0.541668 -1.122011

C -0.489326 -1.5432 0.905606

C -1.749162 -2.052861 1.248995

C 0.409828 -2.311756 0.137573

C -2.13091 -3.318655 0.824778

H -2.422641 -1.472361 1.86562

C -0.026531 -3.57293 -0.300178

C -1.276692 -4.081118 0.037359

H -1.585416 -5.062901 -0.307895

C 2.622827 -1.489594 1.027021

H 2.250023 -2.134829 1.836101

C 1.803988 -1.856765 -0.243628

H 2.312519 -2.744389 -0.629724

C 1.834276 -0.811907 -1.358803

C 3.024641 -0.151878 -1.685536

C 0.701247 -0.517445 -2.120188

C 3.080312 0.778124 -2.716656

H 3.927056 -0.36462 -1.123619

C 0.743282 0.401412 -3.163774

H -0.242383 -1.005936 -1.898986

C 1.934194 1.05785 -3.461457

H 4.012797 1.285609 -2.948234

H -0.148048 0.621568 -3.741895

O 4.009619 -1.70409 0.855622

O 4.134489 2.936501 2.374822

O 1.925612 1.961046 -4.489892

O 0.823499 -4.296106 -1.084191

O -3.339413 -3.861335 1.144668

O -4.226786 1.729093 -3.229539

O -2.014644 0.579579 2.642387

H -2.698636 1.287513 2.744759

H 2.810333 2.3306 -4.59807

H 0.415873 -5.140414 -1.312029

H -3.838771 -3.238397 1.687208

H -3.694887 2.180142 -3.896371

H 4.139819 -2.64788 0.708856

O -3.725188 2.666978 2.803611

H -3.058236 3.354311 2.684421

H -4.163205 2.62515 1.944445

TS6'.log

Energy (E) = -1756.74076810 Hartree

Enthalpy (H) = -1756.256462 Hartree

Gibbs free energy (G) = -1756.349972 Hartree

Charge = 0, Spin = 1

Imaginary frequency: -271.1618

C 3.38291 2.855293 0.895095

C 2.01801 3.234165 1.124506

C 1.036353 2.286087 1.111945

C 1.336893 0.846966 0.929775

C 2.702099 0.487741 0.545536

C 3.652099 1.442449 0.567333

H 1.776516 4.277522 1.294951

H 4.671988 1.2091 0.286644

C 0.275543 0.05403 1.215938

C -0.988426 0.783194 1.665565

O -0.234605 2.534676 1.271467

C -2.12276 0.831353 0.696204

C -1.901466 0.892982 -0.682566

C -3.433411 0.823807 1.17647

C -2.966173 0.948499 -1.567897

H -0.887521 0.911794 -1.071191

C -4.508422 0.8747 0.301134

H -3.608678 0.769934 2.245727

C -4.274561 0.939855 -1.074216

H -2.790846 1.002758 -2.638701

H -5.529629 0.863491 0.666478

C 0.166792 -1.422163 1.201002

C -0.756906 -2.016287 2.075561

C 0.921285 -2.219851 0.3225

C -0.920198 -3.394972 2.092745

H -1.339704 -1.412263 2.759739

C 0.720594 -3.609748 0.360341

C -0.184552 -4.201792 1.233289

H -0.321359 -5.278436 1.242792

C 3.043992 -0.915443 0.098051

H 3.235876 -1.513236 1.001299

C 1.919235 -1.648792 -0.662206

H 2.429467 -2.508146 -1.105805

C 1.237115 -0.908449 -1.811726

C 1.71762 0.278203 -2.366875

C 0.08088 -1.46441 -2.373156

C 1.042443 0.919226 -3.404042

H 2.628843 0.729296 -1.992667

C -0.592209 -0.850737 -3.419417

H -0.314012 -2.394901 -1.976398

C -0.121654 0.360486 -3.925342

H 1.422155 1.853547 -3.807988

H -1.493145 -1.289609 -3.835222

O 4.227505 -0.84891 -0.680043

O 4.325951 3.671519 0.922159

O -0.842046 0.951688 -4.927837

O 1.44028 -4.375402 -0.509399

O -1.797407 -4.01198 2.934289

O -5.359399 0.992821 -1.893844

O -1.213365 0.827643 2.912738

H -1.641908 2.230917 3.289406

H -0.417411 1.780972 -5.179032

H 1.197822 -5.302316 -0.396474

H -2.247104 -3.347288 3.470651

H -5.067441 1.038428 -2.812681

H 4.543523 -1.752238 -0.793572

O -1.52943 3.25678 3.224726

H -0.906017 3.256825 2.398559

H -2.380018 3.644813 2.966146

INT5'.log

Energy (E) = -1756.75954353 Hartree

Enthalpy (H) = -1756.271584 Hartree

Gibbs free energy (G) = -1756.368440 Hartree

Charge = 0, Spin = 1

C 3.909999 2.387487 0.302607

C 2.621999 3.047009 0.344878

C 1.485946 2.312835 0.338908

C 1.509018 0.838011 0.465284

C 2.77705 0.19354 0.08689

C 3.899207 0.937427 0.061764

H 2.583351 4.130621 0.318915

H 4.852665 0.487219 -0.18931

C 0.448442 0.234705 1.062398

C -0.697518 1.073333 1.623819

O 0.263792 2.851191 0.253643

C -1.987476 1.050722 0.911649

C -2.060817 0.690142 -0.437673

C -3.156654 1.419818 1.588007

C -3.274198 0.695705 -1.104967

H -1.1586 0.425532 -0.979251

C -4.376982 1.414896 0.936496

H -3.100312 1.700363 2.634524

C -4.43686 1.05345 -0.414793

H -3.326892 0.43157 -2.157116

H -5.290864 1.686772 1.453267

C 0.265398 -1.207472 1.362933

C -0.456919 -1.534517 2.521958

C 0.722448 -2.220012 0.50399

C -0.725773 -2.861765 2.828852

H -0.800172 -0.764614 3.203028

C 0.420265 -3.551034 0.839675

C -0.296286 -3.879095 1.984263

H -0.519075 -4.915302 2.218375

C 2.869435 -1.271409 -0.299797

H 3.208418 -1.824795 0.587672

C 1.553822 -1.939522 -0.729777

H 1.873429 -2.915103 -1.105716

C 0.749023 -1.293089 -1.852676

C 1.141717 -0.151874 -2.552037

C -0.461978 -1.895192 -2.217367

C 0.33392 0.402502 -3.543652

H 2.087128 0.332154 -2.333229

C -1.270307 -1.366652 -3.212787

H -0.786514 -2.795189 -1.702709

C -0.879925 -0.198083 -3.867855

H 0.648048 1.303096 -4.063642

H -2.210035 -1.839803 -3.477944

O 3.852303 -1.372336 -1.319295

O 4.982109 3.008505 0.373356

O -1.725675 0.309843 -4.815745

O 0.847344 -4.526568 -0.011358

O -1.416638 -3.224936 3.945318

O -5.655843 1.069086 -1.008815

O -0.549885 1.60939 2.719575

H -0.441483 3.751187 2.697148

H -1.344021 1.116457 -5.182845

H 0.57932 -5.389216 0.327288

H -1.680638 -2.433412 4.430705

H -5.572798 0.806079 -1.934319

H 4.07433 -2.306422 -1.407142

O -0.173276 4.535253 2.193929

H 0.180282 3.661494 0.828396

H -1.00538 4.984688 2.003113

INT5_2.log

Energy (E) = -1680.32954075 Hartree

Enthalpy (H) = -1679.869388 Hartree

Gibbs free energy (G) = -1679.961681 Hartree

Charge = 0, Spin = 1

C -0.669258 -3.373925 2.005626

C 0.07834 -3.411833 0.77116

C 0.281948 -2.280667 0.057537

C -0.122283 -0.942699 0.543297

C -1.076455 -0.957018 1.668481

C -1.289587 -2.092773 2.35823

H 0.422279 -4.370366 0.396093

H -1.965468 -2.103051 3.20383

C 0.456043 0.173072 0.019239

C 1.494674 0.087532 -1.099116

O 0.869764 -2.290172 -1.146988

C 2.891362 -0.25464 -0.763973

C 3.847465 -0.271426 -1.787428

C 3.289743 -0.549805 0.546848

C 5.168013 -0.580465 -1.516159

H 3.542959 -0.041084 -2.802642

C 4.609042 -0.858335 0.830844

H 2.565673 -0.532235 1.355128

C 5.551264 -0.87504 -0.201791

H 5.910191 -0.595563 -2.308887

H 4.925442 -1.085151 1.842782

C 0.166651 1.604032 0.309915

C 1.241101 2.497685 0.239804

C -1.145157 2.079693 0.503257

C 1.022595 3.866984 0.344357

H 2.258554 2.149924 0.106219

C -1.337728 3.468775 0.522974

C -0.270579 4.360175 0.456161

H -0.462191 5.427961 0.489568

C -1.816471 0.29905 2.063839

H -1.129768 0.934193 2.640041

C -2.277019 1.138439 0.837134

H -3.099647 1.734993 1.246679

C -2.838268 0.324769 -0.323324

C -3.816371 -0.648102 -0.085606

C -2.412725 0.514905 -1.637813

C -4.341849 -1.413038 -1.118357

H -4.16582 -0.816827 0.927361

C -2.931632 -0.241336 -2.684707

H -1.647582 1.252102 -1.858887

C -3.898079 -1.210529 -2.425516

H -5.096586 -2.167725 -0.922716

H -2.583263 -0.082757 -3.701416

O -2.90603 -0.068372 2.887613

O -0.843414 -4.379824 2.711852

O -4.444141 -1.987271 -3.410286

O -2.572601 4.043426 0.63612

O 2.113359 4.680686 0.295152

O 6.830028 -1.181168 0.124997

O 1.142263 0.418563 -2.223991

H 1.22735 -3.167718 -1.339215

H -4.053984 -1.744708 -4.258772

H -3.260572 3.414505 0.390934

H 1.833356 5.60117 0.36885

H 7.382708 -1.155935 -0.66639

H -3.246139 0.740443 3.286202

INT5'_2.log

Energy (E) = -1680.33215304 Hartree

Enthalpy (H) = -1679.871903 Hartree

Gibbs free energy (G) = -1679.961846 Hartree

Charge = 0, Spin = 1

C -4.02174 -1.614457 1.7635

C -2.773208 -2.113521 2.292658

C -1.601325 -1.54746 1.923295

C -1.539141 -0.328443 1.084972

C -2.765623 -0.047357 0.31577

C -3.925041 -0.62278 0.685243

H -2.789247 -2.972811 2.955571

H -4.839786 -0.410032 0.145469

C -0.457762 0.488748 1.169429

C 0.677314 0.198839 2.14844

O -0.410995 -2.028255 2.305047

C 1.940171 -0.335218 1.604899

C 1.980352 -1.000451 0.375165

C 3.11795 -0.202639 2.351578

C 3.168825 -1.526835 -0.104198

H 1.071588 -1.131356 -0.203033

C 4.313618 -0.709114 1.875877

H 3.087482 0.312154 3.306068

C 4.339467 -1.376678 0.645444

H 3.194155 -2.060537 -1.049856

H 5.2337 -0.601371 2.439706

C -0.209838 1.773633 0.459101

C 0.479441 2.764103 1.169126

C -0.556017 1.97576 -0.891947

C 0.837796 3.955143 0.548096

H 0.736209 2.634782 2.21233

C -0.122977 3.164634 -1.50225

C 0.559889 4.151286 -0.796282

H 0.869421 5.05679 -1.308075

C -2.77012 0.851846 -0.907501

H -3.037011 1.864736 -0.575216

C -1.427732 0.996753 -1.650512

H -1.740664 1.489917 -2.577368

C -0.688986 -0.27042 -2.068825

C -1.173121 -1.56563 -1.874971

C 0.550858 -0.126643 -2.700045

C -0.422143 -2.679471 -2.240881

H -2.146051 -1.728856 -1.424981

C 1.304592 -1.225772 -3.087812

H 0.948847 0.867747 -2.883082

C 0.826354 -2.511496 -2.835286

H -0.798913 -3.681489 -2.064562

H 2.270289 -1.090499 -3.5662

O -3.774915 0.362558 -1.780684

O -5.124368 -2.048225 2.13116

O 1.543957 -3.632287 -3.15031

O -0.338304 3.45262 -2.820277

O 1.485723 4.884508 1.303684

O 5.53443 -1.861536 0.226248

O 0.546894 0.541796 3.316932

H -0.521886 -2.738935 2.95153

H 2.3838 -3.368875 -3.546137

H -0.612184 2.666688 -3.306154

H 1.694396 5.653067 0.758729

H 5.427097 -2.297425 -0.628678

H -3.98173 1.068697 -2.402961

TS7.log

Energy (E) = -1680.29787623 Hartree

Enthalpy (H) = -1679.838524 Hartree

Gibbs free energy (G) = -1679.926677 Hartree

Charge = 0, Spin = 1

Imaginary frequency: -37.6989

C -4.425683 -0.075065 1.416345

C -3.924802 1.156666 1.982427

C -2.876671 1.767592 1.386533

C -2.047645 1.096946 0.346118

C -2.795356 0.105496 -0.460868

C -3.880209 -0.462297 0.10369

H -4.509549 1.659007 2.746473

H -4.382007 -1.284509 -0.388541

C -0.699666 1.317167 0.259472

C 0.109249 1.619267 1.525669

O -2.587893 3.063895 1.552102

C 1.482017 1.033033 1.619438

C 1.839205 -0.194351 1.055833

C 2.428014 1.719726 2.39235

C 3.105283 -0.725136 1.250145

H 1.122275 -0.760592 0.476758

C 3.704627 1.214159 2.570743

H 2.159612 2.669506 2.842658

C 4.0454 -0.016174 2.000661

H 3.366734 -1.687597 0.819856

H 4.444748 1.755584 3.149911

C 0.119398 1.294824 -0.988956

C 0.954704 2.412611 -1.086334

C 0.068816 0.342191 -2.019475

C 1.761845 2.594325 -2.203159

H 0.983391 3.159918 -0.300459

C 0.914652 0.546132 -3.125895

C 1.751716 1.65564 -3.222895

H 2.383588 1.769004 -4.097767

C -2.329451 -0.397882 -1.813485

H -2.443662 0.447142 -2.507565

C -0.861718 -0.852702 -1.959681

H -0.88865 -1.293994 -2.961435

C -0.332053 -1.955327 -1.045064

C -0.937746 -2.381061 0.137739

C 0.888098 -2.548056 -1.395911

C -0.335754 -3.330154 0.960033

H -1.889306 -1.973138 0.449794

C 1.500379 -3.496981 -0.589839

H 1.386813 -2.25065 -2.313857

C 0.891842 -3.881819 0.606642

H -0.814499 -3.64118 1.882669

H 2.450951 -3.935583 -0.880259

O -3.190267 -1.452719 -2.200634

O -5.341638 -0.731857 1.929683

O 1.451859 -4.794627 1.455453

O 1.003983 -0.307937 -4.188279

O 2.547407 3.706739 -2.238143

O 5.305814 -0.478717 2.209247

O -0.316127 2.23574 2.490139

H -3.180071 3.449668 2.213277

H 2.297664 -5.089726 1.096693

H 0.430119 -1.074304 -4.084384

H 3.056737 3.713714 -3.057701

H 5.411587 -1.333914 1.77359

H -3.08253 -1.575112 -3.14991

MalibatolA_Conf1.log

Energy (E) = -1605.16768673 Hartree

Enthalpy (H) = -1604.711781 Hartree

Gibbs free energy (G) = -1604.799127 Hartree

Charge = 0, Spin = 1

C 3.067992 2.455425 1.979705

C 1.7968 2.986656 1.793844

C 0.81872 2.074337 1.428561

C 1.034602 0.708599 1.266519

C 2.331894 0.20218 1.418612

C 3.335591 1.087244 1.783646

H 1.597034 4.044483 1.913368

H 4.353471 0.730257 1.89743

C -0.241986 0.136518 0.891874

C -1.10267 1.192917 0.799738

O -0.478943 2.379045 1.14431

C -2.489729 1.335582 0.354772

C -3.358535 2.229363 0.992671

C -2.962219 0.601214 -0.739394

C -4.669901 2.373726 0.563868

H -3.006507 2.811712 1.837924

C -4.273814 0.7327 -1.17079

H -2.29256 -0.074715 -1.261782

C -5.132121 1.619172 -0.515837

H -5.345727 3.061675 1.060386

H -4.634354 0.160594 -2.020492

C -0.545817 -1.30099 0.749698

C -1.803964 -1.765228 1.150133

C 0.421715 -2.192424 0.253105

C -2.125282 -3.110322 1.018835

H -2.5331 -1.081544 1.571256

C 0.064554 -3.546906 0.14466

C -1.194565 -4.010961 0.511763

H -1.448108 -5.062037 0.415128

C 2.601028 -1.251931 1.121349

H 2.239967 -1.853365 1.967077

C 1.823378 -1.75874 -0.136175

H 2.35865 -2.669335 -0.420975

C 1.867152 -0.819574 -1.34146

C 3.03575 -0.11955 -1.667137

C 0.762361 -0.650645 -2.178438

C 3.092474 0.732673 -2.762455

H 3.91817 -0.236555 -1.048298

C 0.803041 0.195522 -3.283257

H -0.159908 -1.183526 -1.970083

C 1.970312 0.896922 -3.574233

H 4.003187 1.274642 -2.996381

H -0.072704 0.314033 -3.915642

O 4.000981 -1.435192 0.992018

O 2.07305 1.752576 -4.638428

O 1.001046 -4.413208 -0.344525

O -3.342845 -3.607816 1.385441

O -6.429869 1.789404 -0.89508

H 1.231928 1.775603 -5.110505

H 0.633661 -5.304894 -0.358029

H -3.901294 -2.8842 1.695111

H -6.624787 1.206553 -1.63921

H 4.162438 -2.384315 0.953394

O 4.060088 3.324165 2.341804

H 4.891905 2.842259 2.420956

MalibatolA_Conf2.log

Energy (E) = -1605.16773158 Hartree

Enthalpy (H) = -1604.711768 Hartree

Gibbs free energy (G) = -1604.799103 Hartree

Charge = 0, Spin = 1

C 3.043998 2.481257 1.979759

C 1.769201 3.002103 1.789297

C 0.799056 2.081344 1.424247

C 1.025661 0.716868 1.266394

C 2.326269 0.220742 1.423807

C 3.322402 1.114452 1.788727

H 1.561084 4.058747 1.905004

H 4.342555 0.765252 1.906354

C -0.245624 0.134308 0.889531

C -1.113976 1.183978 0.792579

O -0.500007 2.375551 1.135807

C -2.500915 1.316477 0.343918

C -2.966641 0.578421 -0.752711

C -3.375224 2.205581 0.977207

C -4.275379 0.704576 -1.189527

H -2.291275 -0.094689 -1.271377

C -4.686659 2.344807 0.54198

H -3.030193 2.79055 1.823515

C -5.140586 1.588607 -0.539507

H -4.636112 0.135244 -2.039402

H -5.361557 3.033764 1.041199

C -0.538654 -1.30561 0.750021

C -1.795388 -1.777318 1.146095

C 0.437075 -2.191757 0.260007

C -2.107171 -3.1249 1.017099

H -2.530862 -1.097523 1.562465

C 0.089306 -3.548782 0.153335

C -1.168128 -4.020487 0.516543

H -1.413975 -5.073548 0.42138

C 2.606819 -1.232441 1.132691

H 2.24709 -1.83329 1.979424

C 1.837143 -1.749587 -0.125517

H 2.379706 -2.657591 -0.404749

C 1.87866 -0.815134 -1.334541

C 3.042849 -0.10683 -1.658117

C 0.776398 -0.659478 -2.177406

C 3.097539 0.740632 -2.75724

H 3.923329 -0.213495 -1.03468

C 0.815205 0.181649 -3.286142

H -0.14232 -1.199135 -1.970894

C 1.977946 0.89131 -3.575183

H 4.004799 1.289033 -2.989594

H -0.058484 0.28957 -3.923246

O 4.008472 -1.406042 1.008881

O 2.078527 1.742412 -4.643233

O 1.033125 -4.410221 -0.330252

O -3.322922 -3.62963 1.379809

O -6.417143 1.679277 -1.007696

H 1.239816 1.755143 -5.119977

H 0.6699 -5.303567 -0.345706

H -3.88845 -2.908874 1.683306

H -6.908277 2.318431 -0.476758

H 4.17656 -2.354057 0.971992

O 4.028099 3.359367 2.341145

H 4.864371 2.885588 2.421982

MalibatolA_Conf3.log

Energy (E) = -1605.16770193 Hartree

Enthalpy (H) = -1604.711742 Hartree

Gibbs free energy (G) = -1604.799035 Hartree

Charge = 0, Spin = 1

C 3.058087 2.455762 1.987251

C 1.788796 2.988715 1.793784

C 0.810856 2.077116 1.426188

C 1.025149 0.710626 1.268607

C 2.320683 0.202239 1.42897

C 3.324163 1.086509 1.796395

H 1.59021 4.047163 1.909742

H 4.340751 0.727918 1.916621

C -0.250587 0.139745 0.889357

C -1.109128 1.197355 0.790819

O -0.484943 2.383254 1.135248

C -2.494293 1.341402 0.340339

C -3.364217 2.237023 0.974151

C -2.963862 0.606396 -0.754629

C -4.673881 2.382626 0.540547

H -3.014503 2.819786 1.820082

C -4.273729 0.73914 -1.190803

H -2.293337 -0.071054 -1.273907

C -5.133208 1.62749 -0.539981

H -5.350554 3.071984 1.033946

H -4.632052 0.166644 -2.041187

C -0.556221 -1.297558 0.748773

C -1.817318 -1.758647 1.143587

C 0.41218 -2.191853 0.259042

C -2.140614 -3.103342 1.013213

H -2.547277 -1.072887 1.559828

C 0.052695 -3.545818 0.15088

C -1.209159 -4.006718 0.512412

H -1.464329 -5.057432 0.41618

C 2.588187 -1.253418 1.138116

H 2.22029 -1.851168 1.983536

C 1.817241 -1.762394 -0.12265

H 2.351927 -2.675339 -0.400895

C 1.872168 -0.827539 -1.330878

C 0.771774 -0.655406 -2.175619

C 3.045267 -0.136165 -1.652198

C 0.823401 0.184592 -3.282913

H -0.154166 -1.182666 -1.969186

C 3.113258 0.711135 -2.752535

H 3.923868 -0.254705 -1.028248

C 1.996873 0.877076 -3.571195

H -0.043085 0.310102 -3.924142

H 4.032943 1.243771 -2.978829

O 3.988503 -1.440383 1.018431

O 2.003502 1.700005 -4.665594

O 0.989824 -4.414933 -0.332137

O -3.36097 -3.597838 1.374546

O -6.429354 1.798882 -0.924167

H 2.872306 2.111967 -4.744657

H 0.620326 -5.305736 -0.347275

H -3.919741 -2.872667 1.67999

H -6.622142 1.215526 -1.668478

H 4.147886 -2.389929 0.981444

O 4.050064 3.323751 2.351634

H 4.880308 2.840295 2.437589

MalibatolA_Conf4.log

Energy (E) = -1605.16737998 Hartree

Enthalpy (H) = -1604.711613 Hartree

Gibbs free energy (G) = -1604.799015 Hartree

Charge = 0, Spin = 1

C 3.068931 2.441669 1.994333

C 1.797899 2.9748 1.813063

C 0.819199 2.065588 1.441614

C 1.034182 0.700891 1.269766

C 2.331584 0.193042 1.416607

C 3.335925 1.075017 1.787251

H 1.598545 4.031732 1.940883

H 4.35385 0.716989 1.897164

C -0.243226 0.132004 0.892721

C -1.10337 1.189457 0.808673

O -0.478439 2.372948 1.160573

C -2.490491 1.336961 0.365659

C -3.355333 2.23301 1.005788

C -2.966445 0.607323 -0.73015

C -4.66603 2.384357 0.577423

H -3.000688 2.811843 1.852347

C -4.277424 0.745897 -1.161236

H -2.299984 -0.070359 -1.254323

C -5.131704 1.634692 -0.504167

H -5.338709 3.074088 1.075749

H -4.640556 0.177396 -2.01225

C -0.547824 -1.30481 0.742645

C -1.802066 -1.772179 1.146116

C 0.42024 -2.192915 0.236576

C -2.121235 -3.117887 1.012424

H -2.534689 -1.09727 1.571988

C 0.065223 -3.545722 0.122565

C -1.192512 -4.014163 0.495043

H -1.437387 -5.068162 0.393437

C 2.600235 -1.258747 1.107972

H 2.240151 -1.866619 1.949498

C 1.820819 -1.755724 -0.152403

H 2.355853 -2.663891 -0.445265

C 1.862722 -0.807092 -1.350192

C 3.031889 -0.107063 -1.673827

C 0.755821 -0.62924 -2.182445

C 3.087418 0.753033 -2.763001

H 3.915788 -0.230634 -1.058372

C 0.795148 0.225129 -3.280985

H -0.166939 -1.161727 -1.975207

C 1.963277 0.925837 -3.570268

H 3.998687 1.294635 -2.995613

H -0.082221 0.350565 -3.90977

O 4.00001 -1.44135 0.975511

O 2.065198 1.78891 -4.628534

O 0.999525 -4.409479 -0.375042

O -3.363572 -3.516212 1.414478

O -6.428667 1.8122 -0.883033

H 1.222912 1.817465 -5.098207

H 0.634164 -5.301934 -0.388274

H -3.448319 -4.469175 1.2895

H -6.626968 1.231267 -1.62775

H 4.161316 -2.390248 0.931101

O 4.06165 3.306967 2.363078

H 4.892438 2.823179 2.441734

MalibatolA_Conf5.log

Energy (E) = -1605.16752123 Hartree

Enthalpy (H) = -1604.711085 Hartree

Gibbs free energy (G) = -1604.799012 Hartree

Charge = 0, Spin = 1

C -2.513892 -3.228505 1.54779

C -1.228779 -3.570174 1.142962

C -0.364231 -2.504189 0.948003

C -0.699959 -1.166992 1.146746

C -2.007816 -0.842962 1.524726

C -2.899706 -1.886853 1.72746

H -0.935214 -4.60022 0.981752

H -3.921468 -1.674133 2.025334

C 0.486372 -0.398741 0.839131

C 1.415994 -1.311925 0.427028

O 0.922032 -2.60191 0.511489

C 2.75999 -1.215157 -0.148662

C 3.056455 -0.237937 -1.108344

C 3.76021 -2.119464 0.221813

C 4.322387 -0.149128 -1.664224

H 2.284875 0.457301 -1.423241

C 5.030772 -2.042848 -0.334168

H 3.547041 -2.887701 0.957942

C 5.315592 -1.051503 -1.274272

H 4.552083 0.606581 -2.40761

H 5.804676 -2.744563 -0.03755

C 0.652165 1.062405 0.9681

C 1.923222 1.549588 1.300949

C -0.424108 1.94816 0.768172

C 2.157387 2.915143 1.373451

H 2.734949 0.860585 1.505388

C -0.141046 3.327204 0.836783

C 1.127211 3.816088 1.126864

H 1.311168 4.884828 1.174972

C -2.41739 0.604197 1.676637

H -2.009763 0.987675 2.6182

C -1.874328 1.543159 0.550842

H -2.453746 2.451834 0.722344

C -2.238482 1.079018 -0.853654

C -1.338516 0.455109 -1.723863

C -3.545788 1.276676 -1.312658

C -1.729116 0.021703 -2.984918

H -0.306375 0.30557 -1.427358

C -3.956555 0.843054 -2.569132

H -4.261697 1.798349 -0.683406

C -3.044583 0.206621 -3.408774

H -1.018336 -0.458408 -3.649637

H -4.977066 1.009403 -2.902533

O -3.821917 0.740671 1.806171

O -3.379813 -0.240085 -4.657191

O -1.168696 4.19619 0.601456

O 3.381146 3.431672 1.687707

O 6.544271 -0.920253 -1.848631

H -4.30884 -0.042625 -4.826824

H -0.849042 5.101042 0.698722

H 4.008625 2.709949 1.817965

H 7.138899 -1.593645 -1.495568

H -4.232259 0.35036 1.023519

O -3.397189 -4.251758 1.752918

H -4.247956 -3.888873 2.027506

MalibatolA_Conf6.log

Energy (E) = -1605.16773707 Hartree

Enthalpy (H) = -1604.711760 Hartree

Gibbs free energy (G) = -1604.798913 Hartree

Charge = 0, Spin = 1

C 3.073926 2.451271 1.975242

C 1.804617 2.985288 1.786545

C 0.823766 2.072934 1.422487

C 1.037148 0.707497 1.265035

C 2.334841 0.198466 1.420154

C 3.338591 1.081669 1.78325

H 1.600349 4.044007 1.901551

H 4.357448 0.733341 1.901325

C -0.240085 0.136811 0.891214

C -1.098958 1.19451 0.795379

O -0.472588 2.38021 1.136671

C -2.485532 1.338303 0.349236

C -3.353914 2.23402 0.984969

C -2.958177 0.602251 -0.743745

C -4.664945 2.378755 0.555166

H -3.001853 2.817576 1.829384

C -4.26948 0.733939 -1.175988

H -2.288858 -0.075304 -1.264464

C -5.127335 1.622437 -0.523221

H -5.340437 3.06822 1.050036

H -4.630143 0.160395 -2.024675

C -0.546557 -1.300513 0.752432

C -1.80574 -1.761508 1.153345

C 0.419549 -2.194816 0.258305

C -2.129325 -3.106366 1.025101

H -2.533868 -1.075541 1.572478

C 0.060145 -3.548946 0.153

C -1.199957 -4.009874 0.520678

H -1.455354 -5.060718 0.426505

C 2.600106 -1.257026 1.126156

H 2.236248 -1.85562 1.97275

C 1.822169 -1.764419 -0.131075

H 2.355851 -2.676484 -0.414133

C 1.868173 -0.827456 -1.337981

C 3.037729 -0.129021 -1.663623

C 0.764413 -0.659014 -2.17643

C 3.09621 0.721583 -2.760126

H 3.919451 -0.24581 -1.043754

C 0.806888 0.185446 -3.282488

H -0.158497 -1.190851 -1.96822

C 1.974975 0.885576 -3.573227

H 4.007608 1.262458 -2.993913

H -0.068088 0.303644 -3.916004

O 3.999519 -1.444866 0.998478

O 2.079303 1.739781 -4.638472

O 0.995464 -4.417958 -0.333627

O -3.347965 -3.600857 1.392219

O -6.424809 1.792947 -0.903405

H 1.238712 1.762446 -5.111507

H 0.626864 -5.309179 -0.345237

H -3.905371 -2.875465 1.699564

H -6.619883 1.208565 -1.646295

H 4.157786 -2.394543 0.960088

O 4.140013 3.228676 2.334331

H 3.851085 4.144619 2.426223

MalibatolA_Conf7.log

Energy (E) = -1605.16777421 Hartree

Enthalpy (H) = -1604.711756 Hartree

Gibbs free energy (G) = -1604.798893 Hartree

Charge = 0, Spin = 1

C 3.047926 2.479558 1.974719

C 1.775257 3.002983 1.779601

C 0.802756 2.08165 1.415826

C 1.027179 0.717331 1.264114

C 2.328079 0.218824 1.426374

C 3.323735 1.111216 1.789462

H 1.562372 4.060545 1.889611

H 4.344834 0.771403 1.912814

C -0.244454 0.135571 0.887961

C -1.111174 1.186259 0.78588

O -0.49488 2.377881 1.124814

C -2.49771 1.319233 0.336022

C -2.963639 0.578914 -0.758985

C -3.371762 2.210221 0.967028

C -4.272245 0.704659 -1.196383

H -2.288575 -0.095887 -1.275843

C -4.683025 2.349166 0.531112

H -3.026716 2.796819 1.812214

C -5.137139 1.590638 -0.54865

H -4.633074 0.13338 -2.044915

H -5.357729 3.039547 1.028626

C -0.539793 -1.304364 0.752897

C -1.797783 -1.772679 1.148973

C 0.434967 -2.193648 0.266671

C -2.111655 -3.120145 1.023785

H -2.532587 -1.090386 1.562399

C 0.084992 -3.550413 0.163643

C -1.173638 -4.018843 0.526936

H -1.421205 -5.07175 0.434671

C 2.605001 -1.236117 1.140348

H 2.241492 -1.833187 1.988199

C 1.836444 -1.755127 -0.117842

H 2.377699 -2.664914 -0.393739

C 1.881725 -0.824145 -1.329408

C 3.047251 -0.117709 -1.65232

C 0.781473 -0.669859 -2.175164

C 3.104916 0.727054 -2.753376

H 3.926314 -0.223514 -1.026753

C 0.823309 0.168457 -3.285918

H -0.138043 -1.208415 -1.96933

C 1.987161 0.876629 -3.574035

H 4.013089 1.274195 -2.985138

H -0.048842 0.275419 -3.925298

O 4.006263 -1.414611 1.019941

O 2.090483 1.725289 -4.64382

O 1.027886 -4.414903 -0.316345

O -3.328659 -3.621752 1.386647

O -6.41363 1.68078 -1.01724

H 1.252978 1.736645 -5.122719

H 0.663451 -5.307811 -0.329411

H -3.893291 -2.899122 1.687304

H -6.904598 2.321146 -0.487609

H 4.171009 -2.363233 0.98341

O 4.106614 3.266866 2.334142

H 3.809874 4.18083 2.420768

MalibatolA_Conf8.log

Energy (E) = -1605.16773237 Hartree

Enthalpy (H) = -1604.711693 Hartree

Gibbs free energy (G) = -1604.798862 Hartree

Charge = 0, Spin = 1

C 3.033697 2.480005 1.988273

C 1.761151 3.003155 1.789436

C 0.790945 2.083561 1.421466

C 1.015488 0.718249 1.268128

C 2.313964 0.219562 1.434693

C 3.310072 1.112008 1.802641

H 1.55465 4.06051 1.901522

H 4.328677 0.760753 1.927427

C -0.255004 0.137393 0.886037

C -1.120826 1.188567 0.782616

O -0.506044 2.379713 1.125943

C -2.50586 1.322775 0.328473

C -2.96857 0.58493 -0.769543

C -3.381423 2.2129 0.958599

C -4.275675 0.712351 -1.210873

H -2.292214 -0.089085 -1.285706

C -4.691245 2.353341 0.518902

H -3.038728 2.797595 1.806052

C -5.142188 1.597404 -0.564022

H -4.634036 0.143207 -2.061874

H -5.367177 3.042996 1.015744

C -0.550233 -1.302198 0.747572

C -1.810233 -1.770475 1.137347

C 0.426328 -2.191383 0.2647

C -2.124335 -3.117561 1.008921

H -2.546462 -1.088506 1.548745

C 0.075858 -3.547769 0.157896

C -1.18464 -4.016018 0.514817

H -1.432372 -5.068644 0.419766

C 2.59228 -1.235348 1.150389

H 2.22441 -1.832233 1.996432

C 1.830322 -1.75399 -0.111904

H 2.372158 -2.664414 -0.384461

C 1.884844 -0.823209 -1.323235

C 0.787682 -0.662046 -2.174278

C 3.054826 -0.125082 -1.641249

C 0.839256 0.173928 -3.2846

H -0.135543 -1.195098 -1.970648

C 3.122665 0.718362 -2.744587

H 3.931012 -0.235143 -1.012366

C 2.009428 0.873349 -3.569638

H -0.024738 0.290806 -3.930806

H 4.039949 1.256207 -2.968284

O 3.994312 -1.413594 1.037762

O 2.016042 1.691915 -4.667303

O 1.020273 -4.412155 -0.319303

O -3.343114 -3.619064 1.365887

O -6.417059 1.689204 -1.036567

H 2.881938 2.110608 -4.742715

H 0.654964 -5.304646 -0.336077

H -3.908799 -2.896705 1.665276

H -6.909165 2.329495 -0.507907

H 4.159718 -2.362209 1.003972

O 4.017842 3.356837 2.352742

H 4.852285 2.881091 2.440741

MalibatolA_Conf9.log

Energy (E) = -1605.16739125 Hartree

Enthalpy (H) = -1604.711551 Hartree

Gibbs free energy (G) = -1604.798853 Hartree

Charge = 0, Spin = 1

C 3.060942 2.44161 2.001201

C 1.791754 2.976679 1.813113

C 0.812925 2.068346 1.439742

C 1.02611 0.702857 1.272052

C 2.321853 0.192867 1.426084

C 3.326206 1.073891 1.798826

H 1.593729 4.034254 1.937663

H 4.342912 0.714159 1.914408

C -0.250672 0.135387 0.890941

C -1.108566 1.194216 0.800848

O -0.482955 2.377353 1.152546

C -2.493849 1.343188 0.352538

C -3.359704 2.241207 0.988534

C -2.967008 0.612774 -0.743951

C -4.668731 2.39376 0.555468

H -3.007269 2.82058 1.835647

C -4.276277 0.752578 -1.179714

H -2.29971 -0.06646 -1.265024

C -5.131644 1.643371 -0.526803

H -5.342184 3.084999 1.050645

H -4.63727 0.183605 -2.031329

C -0.557385 -1.301169 0.742776

C -1.814465 -1.765097 1.141391

C 0.41111 -2.192379 0.243071

C -2.135984 -3.110305 1.008733

H -2.547644 -1.087825 1.562477

C 0.053527 -3.544633 0.129724

C -1.206879 -4.009652 0.497381

H -1.453712 -5.063256 0.396455

C 2.58882 -1.260369 1.122912

H 2.223086 -1.86497 1.964397

C 1.814703 -1.759405 -0.139875

H 2.348834 -2.669969 -0.426859

C 1.86606 -0.8152 -1.340908

C 0.762977 -0.636363 -2.180665

C 3.038385 -0.121874 -1.660881

C 0.811397 0.211465 -3.282099

H -0.162487 -1.164699 -1.974813

C 3.103263 0.733126 -2.75542

H 3.918915 -0.245234 -1.040601

C 1.984318 0.905312 -3.56933

H -0.057156 0.342117 -3.919492

H 4.022407 1.267072 -2.980883

O 3.988855 -1.446286 0.998242

O 1.987768 1.735742 -4.658071

O 0.988185 -4.411369 -0.362069

O -3.381038 -3.505108 1.405798

O -6.42704 1.822054 -0.910493

H 2.856741 2.147173 -4.737983

H 0.620445 -5.302823 -0.377157

H -3.467741 -4.4579 1.280955

H -6.623302 1.240626 -1.655377

H 4.148202 -2.395499 0.95337

O 4.053846 3.30592 2.371931

H 4.883101 2.820468 2.456298

MalibatolA_Conf10.log

Energy (E) = -1605.16743357 Hartree

Enthalpy (H) = -1604.711596 Hartree

Gibbs free energy (G) = -1604.798852 Hartree

Charge = 0, Spin = 1

C 3.075758 2.435531 1.989594

C 1.806703 2.971927 1.806076

C 0.824681 2.063038 1.436456

C 1.036584 0.698538 1.269101

C 2.334182 0.187508 1.418788

C 3.33908 1.067199 1.787153

H 1.603464 4.029951 1.928995

H 4.35778 0.717168 1.901429

C -0.241653 0.131642 0.892618

C -1.099553 1.190784 0.805042

O -0.471544 2.373545 1.153967

C -2.486056 1.340095 0.360644

C -3.350302 2.23806 0.99886

C -2.962185 0.60945 -0.734432

C -4.660526 2.390408 0.5693

H -2.995602 2.817575 1.844939

C -4.27273 0.748868 -1.166579

H -2.296188 -0.069837 -1.257119

C -5.126401 1.639651 -0.511438

H -5.332749 3.081629 1.066179

H -4.635998 0.179463 -2.016937

C -0.549398 -1.304792 0.74493

C -1.80501 -1.768656 1.148151

C 0.416939 -2.195813 0.240754

C -2.127126 -3.113829 1.016162

H -2.536463 -1.091405 1.572302

C 0.059035 -3.548019 0.128614

C -1.200017 -4.013027 0.500965

H -1.447284 -5.066608 0.40083

C 2.598245 -1.265757 1.113153

H 2.234577 -1.87088 1.955167

C 1.818812 -1.762267 -0.147559

H 2.352001 -2.671938 -0.439073

C 1.863924 -0.81496 -1.346284

C 3.034927 -0.117749 -1.669386

C 0.758142 -0.635221 -2.17962

C 3.093151 0.741837 -2.758833

H 3.918056 -0.242925 -1.053155

C 0.80018 0.218604 -3.278489

H -0.165919 -1.165668 -1.972931

C 1.969975 0.916851 -3.566964

H 4.005768 1.281405 -2.990885

H -0.076385 0.345637 -3.908078

O 3.997416 -1.453757 0.982903

O 2.074405 1.779602 -4.625286

O 0.991881 -4.414542 -0.366919

O -3.370744 -3.508789 1.417606

O -6.422957 1.818054 -0.891412

H 1.232445 1.809821 -5.095431

H 0.624797 -5.30631 -0.379291

H -3.457719 -4.461601 1.293027

H -6.621504 1.236009 -1.635197

H 4.15496 -2.403281 0.938144

O 4.142906 3.209333 2.353396

H 3.854889 4.124815 2.452367

MalibatolA_Conf11.log

Energy (E) = -1605.16776104 Hartree

Enthalpy (H) = -1604.711733 Hartree

Gibbs free energy (G) = -1604.798852 Hartree

Charge = 0, Spin = 1

C 3.064288 2.451554 1.982825

C 1.796952 2.987426 1.786442

C 0.816186 2.075904 1.420013

C 1.027849 0.709705 1.266953

C 2.32371 0.198612 1.430295

C 3.327313 1.080914 1.795942

H 1.594025 4.046793 1.897817

H 4.344825 0.730908 1.920509

C -0.248607 0.140342 0.888514

C -1.105234 1.199313 0.786399

O -0.478299 2.384729 1.12758

C -2.489906 1.344407 0.334724

C -3.359772 2.241265 0.966797

C -2.959219 0.60813 -0.759524

C -4.669119 2.386958 0.532164

H -3.010265 2.82492 1.81221

C -4.268809 0.740792 -1.196577

H -2.288667 -0.070333 -1.27744

C -5.128226 1.630458 -0.547497

H -5.345778 3.0773 1.024205

H -4.626957 0.167195 -2.046298

C -0.557061 -1.296732 0.751478

C -1.819289 -1.754347 1.146703

C 0.409841 -2.194098 0.264489

C -2.144981 -3.098796 1.019649

H -2.548184 -1.066156 1.560784

C 0.048031 -3.547697 0.159824

C -1.214911 -4.005241 0.52186

H -1.472053 -5.055714 0.428345

C 2.587217 -1.258464 1.142859

H 2.216575 -1.853201 1.989235

C 1.81585 -1.768124 -0.117481

H 2.348863 -2.68255 -0.394016

C 1.87298 -0.8356 -1.327373

C 0.773586 -0.663935 -2.173504

C 3.047127 -0.146104 -1.648846

C 0.827039 0.174152 -3.282116

H -0.15303 -1.190022 -1.967072

C 3.116945 0.699355 -2.750553

H 3.925064 -0.264444 -1.023924

C 2.001432 0.865109 -3.570419

H -0.038733 0.29939 -3.924368

H 4.037392 1.230698 -2.976803

O 3.986963 -1.450169 1.024698

O 2.009589 1.686397 -4.666066

O 0.984002 -4.419673 -0.320266

O -3.366511 -3.590063 1.381439

O -6.424145 1.801806 -0.932586

H 2.878915 2.097184 -4.745327

H 0.613394 -5.310064 -0.332979

H -3.924241 -2.862944 1.684088

H -6.616753 1.217526 -1.676218

H 4.143075 -2.400323 0.989248

O 4.130058 3.227957 2.345103

H 3.842017 4.14442 2.434556

MalibatolA_Conf12.log

Energy (E) = -1605.16760362 Hartree

Enthalpy (H) = -1604.711125 Hartree

Gibbs free energy (G) = -1604.798789 Hartree

Charge = 0, Spin = 1

C -2.533075 -3.187793 1.580416

C -1.248499 -3.540387 1.18556

C -0.377298 -2.479427 0.982448

C -0.707874 -1.140019 1.164982

C -2.016977 -0.805848 1.536167

C -2.913488 -1.842363 1.746193

H -0.952249 -4.572306 1.033651

H -3.936312 -1.633584 2.037669

C 0.48178 -0.380436 0.849505

C 1.40883 -1.302262 0.450544

O 0.908953 -2.588857 0.550301

C 2.753505 -1.219005 -0.125719

C 3.054628 -0.2559 -1.098152

C 3.749258 -2.123522 0.256196

C 4.320655 -0.181218 -1.655882

H 2.286626 0.439507 -1.421391

C 5.019897 -2.061051 -0.301472

H 3.532467 -2.880816 1.002545

C 5.309201 -1.083856 -1.254872

H 4.553997 0.563413 -2.409245

H 5.790347 -2.762893 0.003786

C 0.652528 1.081883 0.956136

C 1.924766 1.569747 1.28333

C -0.419993 1.968039 0.738631

C 2.164112 2.935466 1.332507

H 2.733373 0.881247 1.50153

C -0.131312 3.346896 0.782321

C 1.138217 3.835872 1.066869

H 1.326344 4.904568 1.095933

C -2.417886 0.645553 1.669331

H -2.005045 1.039907 2.60414

C -1.87177 1.564943 0.528208

H -2.447966 2.478396 0.684959

C -2.238782 1.077009 -0.867199

C -3.549383 1.264885 -1.325771

C -1.342459 0.436828 -1.726684

C -3.965131 0.802388 -2.568394

H -4.261856 1.800473 -0.704391

C -1.738844 -0.026041 -2.977281

H -0.308677 0.294512 -1.432151

C -3.056904 0.145491 -3.396802

H -4.984372 0.956011 -2.907348

H -1.024323 -0.520216 -3.629743

O -3.82104 0.79229 1.800526

O -3.504508 -0.296387 -4.611356

O -1.154549 4.215456 0.527349

O 3.389084 3.452719 1.640882

O 6.538025 -0.967002 -1.832008

H -2.778538 -0.728655 -5.077184

H -0.83082 5.120613 0.606552

H 4.013537 2.731079 1.785357

H 7.129422 -1.638058 -1.469291

H -4.235994 0.387999 1.027459

O -3.500031 -4.12466 1.815909

H -3.133658 -5.0049 1.667648

MalibatolA_Conf13.log

Energy (E) = -1605.16743144 Hartree

Enthalpy (H) = -1604.711550 Hartree

Gibbs free energy (G) = -1604.798787 Hartree

Charge = 0, Spin = 1

C 3.047329 2.463518 1.996876

C 1.772662 2.986977 1.812929

C 0.801291 2.070255 1.440898

C 1.026541 0.706998 1.270897

C 2.327293 0.2088 1.420825

C 3.324677 1.098505 1.79204

H 1.565418 4.04261 1.93895

H 4.344927 0.747823 1.90418

C -0.246024 0.128594 0.891784

C -1.113601 1.179683 0.80499

O -0.49791 2.367959 1.157044

C -2.500468 1.317682 0.358033

C -2.968734 0.585881 -0.741687

C -3.371378 2.208087 0.994194

C -4.276502 0.719734 -1.17911

H -2.296021 -0.088202 -1.262534

C -4.681829 2.354878 0.558521

H -3.024406 2.788358 1.842935

C -5.138231 1.605169 -0.526452

H -4.639104 0.155323 -2.031468

H -5.354093 3.044863 1.059912

C -0.540426 -1.310311 0.742622

C -1.792691 -1.785396 1.143144

C 0.43496 -2.192594 0.240438

C -2.102549 -3.133431 1.010657

H -2.530851 -1.114837 1.566357

C 0.088947 -3.547712 0.126755

C -1.166615 -4.024095 0.496622

H -1.404065 -5.079848 0.395558

C 2.606732 -1.241596 1.11519

H 2.249177 -1.850449 1.95711

C 1.833508 -1.746539 -0.145835

H 2.375322 -2.65176 -0.435415

C 1.871681 -0.800519 -1.345829

C 3.036338 -0.092336 -1.66798

C 0.766012 -0.633889 -2.182069

C 3.088628 0.764646 -2.759782

H 3.919231 -0.207064 -1.049387

C 0.802206 0.217186 -3.283264

H -0.15327 -1.172838 -1.976123

C 1.96584 0.925823 -3.571329

H 3.996432 1.31248 -2.991348

H -0.074091 0.333603 -3.915269

O 4.008036 -1.414381 0.986037

O 2.064454 1.78591 -4.632348

O 1.029766 -4.406029 -0.367897

O -3.343102 -3.539254 1.410682

O -6.413858 1.703737 -0.99581

H 1.223911 1.805215 -5.105625

H 0.668837 -5.300247 -0.38282

H -3.421062 -4.493153 1.288509

H -6.902851 2.342812 -0.462807

H 4.175956 -2.362103 0.941561

O 4.032534 3.337327 2.365799

H 4.867673 2.861283 2.445174

MalibatolA_Conf14.log

Energy (E) = -1605.16745389 Hartree

Enthalpy (H) = -1604.711550 Hartree

Gibbs free energy (G) = -1604.798719 Hartree

Charge = 0, Spin = 1

C 3.068478 2.435625 1.99604

C 1.80136 2.974137 1.805581

C 0.819099 2.066284 1.434015

C 1.029008 0.700967 1.270842

C 2.324838 0.18765 1.427712

C 3.329899 1.066239 1.798248

H 1.599667 4.032845 1.925126

H 4.347303 0.714338 1.918252

C -0.248768 0.135622 0.890557

C -1.104269 1.196218 0.797026

O -0.475367 2.378601 1.145545

C -2.488954 1.346881 0.347412

C -3.35462 2.246018 0.982012

C -2.96187 0.615792 -0.748751

C -4.663244 2.399151 0.547819

H -3.002363 2.825777 1.828952

C -4.270779 0.756013 -1.185503

H -2.294623 -0.064408 -1.26864

C -5.125959 1.648006 -0.534005

H -5.336611 3.09127 1.041887

H -4.631635 0.186404 -2.036755

C -0.558968 -1.300518 0.74529

C -1.817556 -1.760475 1.143691

C 0.407638 -2.19507 0.247972

C -2.142304 -3.105129 1.013249

H -2.549416 -1.080587 1.562837

C 0.046966 -3.546702 0.137184

C -1.214931 -4.007831 0.504697

H -1.464409 -5.060994 0.405689

C 2.586998 -1.26716 1.127946

H 2.217994 -1.868653 1.97025

C 1.812461 -1.766087 -0.134763

H 2.344666 -2.678205 -0.420327

C 1.866682 -0.823536 -1.336927

C 0.764644 -0.643407 -2.177802

C 3.040686 -0.13301 -1.65672

C 0.81558 0.203338 -3.279918

H -0.162004 -1.169804 -1.972251

C 3.108084 0.72095 -2.75199

H 3.92053 -0.257515 -1.035681

C 1.990081 0.89471 -3.566812

H -0.052213 0.335071 -3.918128

H 4.028507 1.252818 -2.977147

O 3.986384 -1.458685 1.00504

O 1.995786 1.72438 -4.656149

O 0.980075 -4.416563 -0.352013

O -3.388821 -3.496094 1.40954

O -6.421037 1.827111 -0.918677

H 2.865779 2.133622 -4.736163

H 0.610689 -5.30738 -0.365327

H -3.477871 -4.448782 1.285576

H -6.61724 1.244998 -1.663049

H 4.142 -2.408675 0.96341

O 4.135598 3.208198 2.362627

H 3.848622 4.124224 2.459567

MalibatolA_Conf15.log

Energy (E) = -1605.16742778 Hartree

Enthalpy (H) = -1604.711494 Hartree

Gibbs free energy (G) = -1604.798710 Hartree

Charge = 0, Spin = 1

C 3.039343 2.461155 2.004969

C 1.766964 2.987444 1.81347

C 0.795106 2.072295 1.438753

C 1.017781 0.708128 1.272783

C 2.316377 0.206884 1.430838

C 3.314148 1.094912 1.804908

H 1.561685 4.043845 1.936255

H 4.332818 0.74172 1.923414

C -0.254308 0.131965 0.888801

C -1.118998 1.184947 0.795981

O -0.502066 2.372487 1.14827

C -2.50397 1.324919 0.343741

C -2.969299 0.593227 -0.757292

C -3.376073 2.216342 0.976795

C -4.27547 0.728262 -1.199062

H -2.295557 -0.081655 -1.275742

C -4.684971 2.364256 0.53681

H -3.031343 2.796421 1.826582

C -5.138484 1.614701 -0.549475

H -4.635786 0.164009 -2.052491

H -5.3582 3.054968 1.035885

C -0.55153 -1.30649 0.740955

C -1.80709 -1.777605 1.135855

C 0.42409 -2.192268 0.245434

C -2.119861 -3.125005 1.004119

H -2.545665 -1.104428 1.554115

C 0.074919 -3.546653 0.132027

C -1.183728 -4.019076 0.496401

H -1.423553 -5.074342 0.395832

C 2.593203 -1.245194 1.131108

H 2.228354 -1.850408 1.97252

C 1.826282 -1.751233 -0.133361

H 2.366975 -2.658957 -0.417006

C 1.876037 -0.80884 -1.335804

C 0.775052 -0.638429 -2.180037

C 3.045392 -0.109229 -1.652855

C 0.822605 0.207257 -3.283147

H -0.14799 -1.171921 -1.976605

C 3.109313 0.743736 -2.749071

H 3.924335 -0.226003 -1.029051

C 1.992512 0.907367 -3.567622

H -0.0443 0.331206 -3.924112

H 4.026261 1.282369 -2.972292

O 3.994768 -1.422786 1.011446

O 1.99524 1.735241 -4.658307

O 1.015925 -4.408306 -0.356469

O -3.363495 -3.526845 1.398532

O -6.412498 1.714263 -1.022992

H 2.861801 2.152289 -4.735281

H 0.652279 -5.301398 -0.373316

H -3.443535 -4.480629 1.276872

H -6.902393 2.354522 -0.492258

H 4.159922 -2.371157 0.97042

O 4.02506 3.333194 2.376782

H 4.858354 2.854915 2.462003

MalibatolA_Conf16.log

Energy (E) = -1605.16747666 Hartree

Enthalpy (H) = -1604.711555 Hartree

Gibbs free energy (G) = -1604.798666 Hartree

Charge = 0, Spin = 1

C 3.054039 2.457001 1.992301

C 1.781624 2.984165 1.805574

C 0.806776 2.067872 1.435284

C 1.028622 0.70469 1.270133

C 2.329374 0.202929 1.423538

C 3.327349 1.090209 1.792665

H 1.570701 4.04094 1.926359

H 4.348255 0.74755 1.909615

C -0.244799 0.128489 0.891323

C -1.109893 1.18145 0.800684

O -0.490908 2.368952 1.149634

C -2.496145 1.321301 0.352326

C -2.964459 0.588789 -0.746909

C -3.366681 2.213081 0.987036

C -4.271887 0.723222 -1.185198

H -2.292062 -0.086511 -1.266591

C -4.676768 2.360583 0.550393

H -3.019769 2.793778 1.83552

C -5.133242 1.610046 -0.533966

H -4.634503 0.158086 -2.037076

H -5.348762 3.051594 1.050735

C -0.542585 -1.310015 0.74444

C -1.796431 -1.781408 1.144359

C 0.431048 -2.195387 0.244338

C -2.109448 -3.12887 1.013434

H -2.533418 -1.108391 1.565698

C 0.081887 -3.549834 0.13227

C -1.175177 -4.022583 0.501687

H -1.415172 -5.077886 0.401939

C 2.60388 -1.249104 1.121269

H 2.241903 -1.854975 1.963493

C 1.831215 -1.753339 -0.140556

H 2.371122 -2.660187 -0.428534

C 1.873565 -0.808554 -1.341364

C 3.040574 -0.103729 -1.662404

C 0.769403 -0.639317 -2.179057

C 3.096397 0.752949 -2.754278

H 3.922412 -0.220609 -1.042708

C 0.809155 0.211422 -3.280397

H -0.151535 -1.175816 -1.974092

C 1.974932 0.917079 -3.567062

H 4.005922 1.298354 -2.984833

H -0.066055 0.330021 -3.91351

O 4.004619 -1.427988 0.995246

O 2.076868 1.777033 -4.627913

O 1.021171 -4.411073 -0.360234

O -3.351433 -3.531195 1.412578

O -6.408613 1.709025 -1.004044

H 1.236758 1.79863 -5.10185

H 0.658347 -5.304544 -0.374367

H -3.431688 -4.48495 1.290754

H -6.897452 2.348872 -0.471833

H 4.168369 -2.376469 0.951209

O 4.114891 3.239119 2.356738

H 3.819932 4.152699 2.452857

MalibatolA_Conf17.log

Energy (E) = -1605.16506417 Hartree

Enthalpy (H) = -1604.709480 Hartree

Gibbs free energy (G) = -1604.798634 Hartree

Charge = 0, Spin = 1

C 3.028698 2.48174 1.954285

C 1.766287 3.01807 1.727717

C 0.783156 2.100354 1.38328

C 0.991586 0.729793 1.27937

C 2.279461 0.216239 1.478472

C 3.286417 1.103232 1.820681

H 1.569663 4.08196 1.800753

H 4.300143 0.752866 1.971623

C -0.278454 0.149436 0.903797

C -1.132671 1.205419 0.756842

O -0.507401 2.400931 1.067057

C -2.510192 1.340593 0.280045

C -2.971039 0.566212 -0.793429

C -3.379166 2.269912 0.86081

C -4.269239 0.697993 -1.258833

H -2.300494 -0.140673 -1.27167

C -4.680025 2.41496 0.396372

H -3.038314 2.882967 1.688709

C -5.128724 1.624276 -0.662278

H -4.625449 0.100669 -2.091225

H -5.350464 3.135668 0.85503

C -0.587064 -1.289274 0.780805

C -1.848799 -1.732254 1.186971

C 0.369184 -2.196078 0.278702

C -2.198762 -3.07046 1.047627

H -2.56166 -1.035586 1.614817

C -0.035983 -3.53324 0.117178

C -1.299849 -3.9743 0.497462

H -1.573292 -5.014171 0.3596

C 2.516992 -1.25386 1.251447

H 2.084781 -1.812406 2.09275

C 1.797011 -1.781267 -0.038453

H 2.366844 -2.691628 -0.253265

C 1.932159 -0.874268 -1.260075

C 3.16149 -0.280004 -1.572519

C 0.857306 -0.621713 -2.113616

C 3.307733 0.54889 -2.676563

H 4.01955 -0.46285 -0.935483

C 0.987231 0.204051 -3.227287

H -0.109822 -1.071801 -1.913914

C 2.215302 0.796966 -3.508029

H 4.264704 1.007844 -2.90298

H 0.13452 0.390167 -3.874416

O 3.913645 -1.48627 1.209733

O 2.406865 1.624147 -4.581598

O 0.774961 -4.503056 -0.411206

O -3.419651 -3.547125 1.429076

O -6.394294 1.71972 -1.158807

H 1.578999 1.703978 -5.070356

H 1.495226 -4.105944 -0.913192

H -3.95647 -2.816377 1.759344

H -6.879035 2.39764 -0.671989

H 4.050131 -2.439891 1.234179

O 4.095633 3.26464 2.298525

H 3.812122 4.185155 2.354752

MalibatolA_Conf18.log

Energy (E) = -1605.16778393 Hartree

Enthalpy (H) = -1604.711687 Hartree

Gibbs free energy (G) = -1604.798629 Hartree

Charge = 0, Spin = 1

C 3.038047 2.477303 1.98372

C 1.767494 3.002971 1.781126

C 0.794883 2.082881 1.41444

C 1.017258 0.717837 1.266431

C 2.316144 0.216854 1.436953

C 3.311854 1.107922 1.803049

H 1.556209 4.061179 1.888003

H 4.331491 0.76613 1.932874

C -0.253629 0.13792 0.884964

C -1.117843 1.190144 0.777107

O -0.500757 2.381125 1.116794

C -2.502417 1.325098 0.32171

C -2.965312 0.585346 -0.774953

C -3.37763 2.217149 0.949584

C -4.272161 0.712761 -1.217069

H -2.289318 -0.09034 -1.289394

C -4.68717 2.357709 0.508998

H -3.034932 2.803228 1.796086

C -5.138287 1.599804 -0.572458

H -4.630593 0.141979 -2.066947

H -5.362797 3.04885 1.004186

C -0.551151 -1.301599 0.750204

C -1.812276 -1.766777 1.140019

C 0.424313 -2.193596 0.270357

C -2.128467 -3.113669 1.01465

H -2.547754 -1.082568 1.549012

C 0.071713 -3.549682 0.166575

C -1.189863 -4.01492 0.523588

H -1.439306 -5.067349 0.430947

C 2.590801 -1.2396 1.156864

H 2.219415 -1.833188 2.003747

C 1.829516 -1.759469 -0.105446

H 2.369997 -2.671448 -0.375424

C 1.887257 -0.831407 -1.318706

C 0.79148 -0.670261 -2.171527

C 3.058724 -0.135651 -1.636489

C 0.845666 0.163733 -3.283215

H -0.132742 -1.20169 -1.968189

C 3.129177 0.705843 -2.741162

H 3.933911 -0.245865 -1.006253

C 2.01717 0.861073 -3.567838

H -0.01729 0.280707 -3.930791

H 4.047467 1.24209 -2.964556

O 3.992374 -1.422608 1.047089

O 2.026194 1.678084 -4.666682

O 1.015143 -4.416808 -0.307622

O -3.34842 -3.612291 1.371672

O -6.412984 1.691455 -1.045627

H 2.893065 2.094722 -4.742127

H 0.648493 -5.308789 -0.322797

H -3.91315 -2.888255 1.668758

H -6.904843 2.333076 -0.518357

H 4.154503 -2.371784 1.01308

O 4.096615 3.263195 2.346676

H 3.801123 4.17779 2.430895

MalibatolA_Conf19.log

Energy (E) = -1605.16748292 Hartree

Enthalpy (H) = -1604.711512 Hartree

Gibbs free energy (G) = -1604.798617 Hartree

Charge = 0, Spin = 1

C 3.044646 2.456811 1.999669

C 1.774303 2.986037 1.805069

C 0.79941 2.070677 1.43252

C 1.019375 0.706644 1.272003

C 2.318204 0.202579 1.433843

C 3.316141 1.088853 1.805416

H 1.56487 4.04354 1.922057

H 4.33566 0.744368 1.928952

C -0.253315 0.131894 0.888649

C -1.116014 1.186273 0.791434

O -0.496332 2.373469 1.14011

C -2.500367 1.327656 0.337608

C -2.965741 0.594826 -0.762637

C -3.372047 2.220924 0.968646

C -4.271452 0.730548 -1.205607

H -2.292425 -0.081744 -1.279452

C -4.680452 2.369646 0.527373

H -3.027435 2.801764 1.817972

C -5.133995 1.61892 -0.55808

H -4.631716 0.16524 -2.058366

H -5.353356 3.061752 1.024959

C -0.553296 -1.306341 0.743522

C -1.810343 -1.774114 1.137637

C 0.420983 -2.194965 0.250456

C -2.125868 -3.121023 1.007481

H -2.548015 -1.098692 1.553836

C 0.069003 -3.548787 0.138623

C -1.191083 -4.017907 0.502268

H -1.433173 -5.072763 0.402872

C 2.590658 -1.251191 1.138278

H 2.22155 -1.853085 1.98028

C 1.824733 -1.757603 -0.126763

H 2.363886 -2.66697 -0.408035

C 1.878663 -0.817187 -1.330607

C 0.778948 -0.644163 -2.176

C 3.050483 -0.121537 -1.647342

C 0.82998 0.200509 -3.279727

H -0.145889 -1.174688 -1.972987

C 3.117902 0.73042 -2.744133

H 3.928468 -0.240399 -1.022579

C 2.002218 0.896897 -3.563662

H -0.035965 0.326565 -3.921583

H 4.036593 1.266235 -2.96698

O 3.991704 -1.434362 1.02209

O 2.008233 1.724201 -4.654809

O 1.008709 -4.413188 -0.347567

O -3.370903 -3.51964 1.400765

O -6.407605 1.719086 -1.032681

H 2.876417 2.137825 -4.731852

H 0.643439 -5.305646 -0.363407

H -3.453018 -4.473296 1.279485

H -6.897236 2.360536 -0.503145

H 4.153069 -2.383381 0.980795

O 4.105293 3.237812 2.367217

H 3.811416 4.152033 2.46053

MalibatolA_Conf20.log

Energy (E) = -1605.16501291 Hartree

Enthalpy (H) = -1604.709462 Hartree

Gibbs free energy (G) = -1604.798497 Hartree

Charge = 0, Spin = 1

C 3.028021 2.480391 1.954901

C 1.763394 3.015288 1.735527

C 0.780934 2.098499 1.393271

C 0.990051 0.727337 1.283751

C 2.277333 0.214787 1.477613

C 3.286123 1.102492 1.819395

H 1.572966 4.078822 1.811721

H 4.29832 0.741014 1.964952

C -0.279893 0.147469 0.906428

C -1.134871 1.203456 0.765051

O -0.510958 2.39841 1.08011

C -2.5127 1.339492 0.289345

C -2.971169 0.573462 -0.791114

C -3.383961 2.26172 0.877856

C -4.269397 0.706525 -1.256081

H -2.298581 -0.12702 -1.275772

C -4.684992 2.407821 0.414139

H -3.044762 2.868378 1.71114

C -5.131259 1.625529 -0.65176

H -4.623843 0.11603 -2.094085

H -5.357227 3.123023 0.878732

C -0.587398 -1.29089 0.776793

C -1.849704 -1.735755 1.179271

C 0.369618 -2.195496 0.271819

C -2.199555 -3.073318 1.033712

H -2.563301 -1.040767 1.608634

C -0.035186 -3.532132 0.104853

C -1.299547 -3.974819 0.481639

H -1.572545 -5.014233 0.339461

C 2.517459 -1.254076 1.24614

H 2.087642 -1.816368 2.086074

C 1.796846 -1.778322 -0.044907

H 2.3683 -2.686864 -0.263354

C 1.929829 -0.866768 -1.26333

C 3.158617 -0.270996 -1.57534

C 0.853598 -0.610823 -2.11407

C 3.302875 0.562898 -2.675904

H 4.01781 -0.456707 -0.940664

C 0.981561 0.21996 -3.224234

H -0.113017 -1.062339 -1.915151

C 2.208896 0.814694 -3.504213

H 4.259403 1.022944 -2.901994

H 0.127725 0.408721 -3.869115

O 3.914505 -1.483451 1.201675

O 2.398091 1.646767 -4.574316

O 0.776182 -4.500185 -0.426129

O -3.421117 -3.551526 1.410882

O -6.396663 1.722492 -1.148175

H 1.568384 1.730903 -5.059307

H 1.4996 -4.101726 -0.92244

H -3.958327 -2.822383 1.744115

H -6.88318 2.395272 -0.656022

H 4.053298 -2.436684 1.228446

O 4.024252 3.354743 2.290704

H 4.853857 2.871441 2.383317

MalibatolA_Conf21.log

Energy (E) = -1605.16758305 Hartree

Enthalpy (H) = -1604.711011 Hartree

Gibbs free energy (G) = -1604.798264 Hartree

Charge = 0, Spin = 1

C -2.534789 -3.193188 1.576718

C -1.250084 -3.545928 1.180074

C -0.379276 -2.486614 0.976642

C -0.708775 -1.145699 1.159916

C -2.016043 -0.810732 1.530185

C -2.914078 -1.847668 1.741023

H -0.961583 -4.579153 1.030489

H -3.93564 -1.62637 2.033226

C 0.481816 -0.386689 0.846413

C 1.407978 -1.308795 0.44673

O 0.907679 -2.595447 0.544518

C 2.753107 -1.223978 -0.128143

C 3.05475 -0.257304 -1.096894

C 3.74915 -2.128753 0.252345

C 4.321656 -0.179111 -1.652044

H 2.286501 0.438255 -1.419193

C 5.02074 -2.062725 -0.302757

H 3.531948 -2.888853 0.995707

C 5.310666 -1.081716 -1.252083

H 4.555449 0.568441 -2.402367

H 5.791511 -2.764619 0.001532

C 0.653699 1.075171 0.957702

C 1.925575 1.56087 1.289483

C -0.417739 1.963005 0.741581

C 2.165631 2.926231 1.345029

H 2.733294 0.870966 1.50649

C -0.128424 3.341493 0.792146

C 1.140788 3.828438 1.081458

H 1.329441 4.896881 1.115851

C -2.418497 0.640212 1.663934

H -2.009608 1.033482 2.600892

C -1.869321 1.561794 0.526354

H -2.445149 2.475289 0.684084

C -2.2335 1.078071 -0.871305

C -3.541848 1.271895 -1.333814

C -1.336663 0.43669 -1.729397

C -3.955083 0.814234 -2.579084

H -4.254475 1.808568 -0.713519

C -1.73046 -0.021291 -2.98257

H -0.30457 0.289255 -1.431485

C -3.046393 0.156406 -3.406224

H -4.972583 0.972529 -2.921104

H -1.015556 -0.516415 -3.633904

O -3.822363 0.785212 1.790624

O -3.491269 -0.280227 -4.623641

O -1.150702 4.211826 0.539335

O 3.390267 3.441359 1.658149

O 6.540477 -0.960967 -1.826239

H -2.765085 -0.713361 -5.088346

H -0.826687 5.116419 0.623621

H 4.013971 2.718651 1.800448

H 7.132306 -1.632162 -1.4645

H -4.234139 0.387533 1.012436

O -3.424335 -4.209504 1.789079

H -4.274869 -3.839254 2.054519

MalibatolA_Conf22.log

Energy (E) = -1605.16727563 Hartree

Enthalpy (H) = -1604.710831 Hartree

Gibbs free energy (G) = -1604.798244 Hartree

Charge = 0, Spin = 1

C -2.541308 -3.171503 1.603128

C -1.256868 -3.529996 1.213158

C -0.383089 -2.472503 1.002931

C -0.711103 -1.130977 1.17442

C -2.020395 -0.791081 1.539709

C -2.919441 -1.823976 1.756556

H -0.962533 -4.563747 1.070226

H -3.942463 -1.610698 2.044045

C 0.480847 -0.37644 0.85566

C 1.406442 -1.303116 0.464981

O 0.903588 -2.587961 0.573657

C 2.751037 -1.226938 -0.112305

C 3.053695 -0.270534 -1.090875

C 3.744128 -2.133084 0.272549

C 4.318445 -0.204538 -1.652531

H 2.287736 0.426218 -1.416085

C 5.013596 -2.079012 -0.288673

H 3.526146 -2.885153 1.023814

C 5.304213 -1.109013 -1.248945

H 4.552901 0.534687 -2.410857

H 5.782084 -2.782064 0.018774

C 0.655066 1.086243 0.952751

C 1.924834 1.573376 1.283056

C -0.416441 1.973448 0.723921

C 2.165671 2.939294 1.326648

H 2.73589 0.892292 1.509068

C -0.126386 3.350501 0.759128

C 1.143855 3.84004 1.048308

H 1.326669 4.911057 1.0684

C -2.419141 0.662102 1.658901

H -2.008371 1.064049 2.591406

C -1.867981 1.570552 0.511387

H -2.443566 2.486078 0.658005

C -2.230771 1.070717 -0.88087

C -3.53949 1.256166 -1.345666

C -1.332309 0.421765 -1.731509

C -3.951519 0.783234 -2.585602

H -4.253441 1.797771 -0.731225

C -1.724888 -0.05162 -2.979362

H -0.299929 0.28017 -1.431698

C -3.041241 0.117921 -3.405009

H -4.969413 0.934989 -2.929414

H -1.008713 -0.552626 -3.624768

O -3.822461 0.812335 1.784755

O -3.485253 -0.334347 -4.617081

O -1.145603 4.220075 0.49239

O 3.426682 3.34956 1.649567

O 6.531842 -1.001223 -1.83056

H -2.758393 -0.772255 -5.076193

H -0.820797 5.125161 0.567308

H 3.452806 4.313622 1.671698

H 7.121357 -1.672265 -1.464806

H -4.236104 0.400905 1.014774

O -3.510474 -4.104403 1.845462

H -3.145176 -4.986656 1.706961

MalibatolA_Conf23.log

Energy (E) = -1605.16691221 Hartree

Enthalpy (H) = -1604.711118 Hartree

Gibbs free energy (G) = -1604.798215 Hartree

Charge = 0, Spin = 1

C 3.080713 2.456999 1.949614

C 1.808434 2.989232 1.777653

C 0.824068 2.07521 1.427205

C 1.03588 0.708968 1.273764

C 2.335415 0.200198 1.41551

C 3.343314 1.087213 1.755699

H 1.605127 4.048252 1.891375

H 4.368964 0.74894 1.849864

C -0.242637 0.138382 0.902812

C -1.101099 1.19663 0.807811

O -0.473072 2.382402 1.146532

C -2.487169 1.344492 0.36095

C -3.351538 2.245528 0.994614

C -2.961771 0.611791 -0.733393

C -4.660366 2.399208 0.561218

H -2.997933 2.826832 1.839934

C -4.27092 0.752522 -1.169342

H -2.295575 -0.069687 -1.252928

C -5.12466 1.646722 -0.518964

H -5.332514 3.093124 1.054428

H -4.632971 0.181813 -2.01935

C -0.550185 -1.298697 0.7594

C -1.808121 -1.759469 1.158928

C 0.41642 -2.193674 0.262439

C -2.133108 -3.104039 1.027843

H -2.539857 -1.07994 1.578858

C 0.05533 -3.544844 0.14933

C -1.206457 -4.00623 0.517383

H -1.456177 -5.059214 0.417208

C 2.594825 -1.264621 1.127917

H 2.223032 -1.854171 1.975858

C 1.821381 -1.765853 -0.125239

H 2.356825 -2.675903 -0.405961

C 1.866276 -0.828219 -1.332107

C 3.022866 -0.101643 -1.641209

C 0.773639 -0.694233 -2.19095

C 3.079714 0.741552 -2.743503

H 3.896454 -0.187721 -1.004583

C 0.814596 0.142657 -3.302776

H -0.138973 -1.247815 -1.994192

C 1.969989 0.869461 -3.578584

H 3.980852 1.304369 -2.9648

H -0.051385 0.233888 -3.95287

O 3.973113 -1.551504 0.942708

O 2.072239 1.717118 -4.649164

O 0.987684 -4.414044 -0.342088

O -3.379286 -3.495058 1.42521

O -6.41971 1.82694 -0.903297

H 1.241044 1.712637 -5.139059

H 0.618485 -5.304933 -0.3553

H -3.468789 -4.447588 1.300362

H -6.617109 1.24393 -1.646643

H 4.405509 -1.504546 1.802758

O 4.151701 3.235866 2.290069

H 3.864648 4.152503 2.381136

MalibatolA_Conf24.log

Energy (E) = -1605.16756589 Hartree

Enthalpy (H) = -1604.710911 Hartree

Gibbs free energy (G) = -1604.798179 Hartree

Charge = 0, Spin = 1

C -2.547488 -3.173937 1.585474

C -1.262617 -3.531895 1.196491

C -0.387318 -2.47432 0.993214

C -0.714105 -1.133319 1.170677

C -2.023596 -0.793822 1.535658

C -2.924224 -1.826763 1.745494

H -0.969133 -4.565196 1.048662

H -3.947546 -1.613728 2.0321

C 0.479012 -0.378488 0.856863

C 1.404256 -1.304431 0.46314

O 0.899838 -2.589111 0.565424

C 2.749984 -1.228761 -0.111601

C 3.739635 -2.14302 0.269075

C 3.058649 -0.267613 -1.081203

C 5.008734 -2.09062 -0.288799

H 3.515886 -2.899195 1.014488

C 4.326389 -0.202737 -1.640419

H 2.2973 0.435223 -1.40404

C 5.306505 -1.115369 -1.242229

H 5.777636 -2.795112 0.00948

H 4.558039 0.544734 -2.393397

C 0.653546 1.083704 0.959409

C 1.925581 1.569076 1.290932

C -0.415478 1.972069 0.733794

C 2.168466 2.934306 1.336578

H 2.731321 0.879011 1.514779

C -0.123165 3.350251 0.774034

C 1.146378 3.836655 1.062911

H 1.337301 4.904924 1.089293

C -2.420728 0.659211 1.661486

H -2.010211 1.056286 2.596163

C -1.867404 1.572097 0.518525

H -2.441882 2.487724 0.6687

C -2.229586 1.078738 -0.876231

C -3.537535 1.268235 -1.341571

C -1.331451 0.431387 -1.728443

C -3.949222 0.800588 -2.583629

H -4.251157 1.808823 -0.725856

C -1.723688 -0.036681 -2.978397

H -0.299631 0.28681 -1.428138

C -3.039359 0.136663 -3.404611

H -4.96654 0.955352 -2.927801

H -1.007822 -0.536586 -3.625004

O -3.823908 0.810677 1.786898

O -3.482988 -0.310413 -4.618734

O -1.142637 4.220813 0.511029

O 3.393439 3.449195 1.64889

O 6.568857 -1.102942 -1.755331

H -2.75651 -0.748164 -5.078608

H -0.816628 5.125254 0.588964

H 4.014835 2.726248 1.799773

H 6.652399 -0.385814 -2.395999

H -4.237506 0.404197 1.014263

O -3.518406 -4.106881 1.820262

H -3.154734 -4.988714 1.674934

MalibatolA_Conf25.log

Energy (E) = -1605.16723016 Hartree

Enthalpy (H) = -1604.710690 Hartree

Gibbs free energy (G) = -1604.798097 Hartree

Charge = 0, Spin = 1

C -2.517034 -3.209865 1.570203

C -1.23167 -3.556493 1.172733

C -0.364043 -2.491898 0.973553

C -0.698409 -1.154306 1.162789

C -2.008396 -0.826119 1.5363

C -2.901537 -1.866427 1.742214

H -0.932259 -4.586733 1.015693

H -3.924837 -1.662012 2.035075

C 0.488875 -0.389795 0.849818

C 1.417834 -1.306962 0.444729

O 0.921808 -2.595485 0.539104

C 2.760488 -1.21775 -0.135326

C 3.055887 -0.250394 -1.10527

C 3.759014 -2.122676 0.238086

C 4.31899 -0.172411 -1.669205

H 2.28549 0.445216 -1.422261

C 5.026769 -2.056709 -0.325652

H 3.546644 -2.883328 0.982289

C 5.31037 -1.075654 -1.276751

H 4.547757 0.575371 -2.42085

H 5.799377 -2.758998 -0.026962

C 0.656288 1.07246 0.964058

C 1.925476 1.561833 1.293506

C -0.420579 1.957242 0.751734

C 2.160226 2.928241 1.351923

H 2.740906 0.882069 1.507339

C -0.13674 3.335197 0.801821

C 1.13269 3.827378 1.089913

H 1.310526 4.898943 1.12154

C -2.414249 0.623223 1.676644

H -2.003567 1.014037 2.61391

C -1.871475 1.550878 0.541061

H -2.449705 2.461793 0.704773

C -2.23937 1.072691 -0.85752

C -1.344051 0.432024 -1.720389

C -3.546074 1.271889 -1.317338

C -1.738831 -0.015647 -2.975124

H -0.312665 0.279551 -1.422417

C -3.960968 0.824194 -2.567532

H -4.25814 1.805314 -0.6936

C -3.053838 0.171281 -3.399708

H -1.031911 -0.50907 -3.634206

H -4.981039 0.991782 -2.901614

O -3.818093 0.764605 1.807083

O -3.393308 -0.290573 -4.641504

O -1.16167 4.202925 0.551199

O 3.421096 3.340606 1.67272

O 6.536219 -0.955713 -1.859818

H -4.321925 -0.091919 -4.812063

H -0.841377 5.108738 0.636202

H 3.442918 4.304463 1.70597

H 7.130186 -1.627372 -1.502368

H -4.230293 0.36573 1.029703

O -3.480826 -4.150968 1.802088

H -3.111748 -5.029305 1.649253

MalibatolA_Conf26.log

Energy (E) = -1605.16505090 Hartree

Enthalpy (H) = -1604.709472 Hartree

Gibbs free energy (G) = -1604.798077 Hartree

Charge = 0, Spin = 1

C 3.04745 2.46911 1.961186

C 1.78428 3.007447 1.743519

C 0.799928 2.092689 1.394893

C 1.007248 0.72284 1.279204

C 2.296423 0.207931 1.466266

C 3.304447 1.092072 1.813402

H 1.58785 4.070662 1.826074

H 4.318786 0.739816 1.955835

C -0.264874 0.146212 0.904835

C -1.118665 1.203931 0.768312

O -0.491423 2.396649 1.084968

C -2.496959 1.342877 0.295383

C -2.958786 0.57632 -0.783329

C -3.365193 2.268057 0.88374

C -4.257338 0.711762 -1.246671

H -2.28816 -0.125786 -1.268379

C -4.666426 2.416737 0.421402

H -3.023413 2.875123 1.715673

C -5.116078 1.63392 -0.642706

H -4.614266 0.121038 -2.083456

H -5.336249 3.134402 0.885701

C -0.576697 -1.291813 0.782859

C -1.837092 -1.732317 1.195802

C 0.376252 -2.200651 0.278445

C -2.188768 -3.070658 1.062008

H -2.547385 -1.03357 1.624551

C -0.029815 -3.53839 0.124606

C -1.292313 -3.977156 0.512433

H -1.566843 -5.017459 0.380048

C 2.537843 -1.259063 1.221779

H 2.125548 -1.827743 2.066273

C 1.798428 -1.78196 -0.05829

H 2.36774 -2.689444 -0.28706

C 1.907012 -0.870879 -1.279958

C 0.823162 -0.661116 -2.136497

C 3.112396 -0.23343 -1.593217

C 0.922633 0.164286 -3.251154

H -0.126238 -1.146667 -1.934646

C 3.228213 0.598585 -2.701009

H 3.977578 -0.382715 -0.956994

C 2.127701 0.80298 -3.533229

H 0.069282 0.320513 -3.903022

H 4.171605 1.090182 -2.922443

O 3.934984 -1.481906 1.150891

O 2.178287 1.612584 -4.635647

O 0.777278 -4.511331 -0.403713

O -3.408733 -3.54483 1.449355

O -6.381864 1.733361 -1.137739

H 3.063023 1.989324 -4.711814

H 1.500051 -4.117762 -0.904844

H -3.943248 -2.812442 1.779753

H -6.865464 2.409629 -0.647488

H 4.078165 -2.434591 1.172653

O 4.115582 3.24882 2.30878

H 3.832276 4.168791 2.37435

MalibatolA_Conf27.log

Energy (E) = -1605.16723656 Hartree

Enthalpy (H) = -1604.710711 Hartree

Gibbs free energy (G) = -1604.797993 Hartree

Charge = 0, Spin = 1

C -2.552661 -3.159678 1.608242

C -1.267922 -3.52228 1.223138

C -0.391176 -2.467366 1.012219

C -0.716428 -1.124573 1.178728

C -2.026132 -0.780639 1.538804

C -2.928154 -1.810799 1.756198

H -0.97543 -4.557096 1.084198

H -3.951573 -1.594266 2.03982

C 0.478257 -0.373769 0.861178

C 1.402746 -1.303848 0.475675

O 0.896264 -2.587097 0.58687

C 2.748779 -1.235018 -0.099035

C 3.734389 -2.15287 0.283471

C 3.060996 -0.278866 -1.072425

C 5.002841 -2.109053 -0.276572

H 3.507944 -2.905277 1.031862

C 4.328096 -0.222716 -1.634003

H 2.30283 0.426763 -1.396648

C 5.304094 -1.139043 -1.234221

H 5.768567 -2.816384 0.023158

H 4.562316 0.520834 -2.390048

C 0.654951 1.088911 0.95474

C 1.924194 1.574659 1.289027

C -0.414001 1.977423 0.719024

C 2.16729 2.940252 1.329868

H 2.733128 0.892657 1.519864

C -0.121675 3.354067 0.75188

C 1.148243 3.842086 1.04491

H 1.332909 4.912831 1.062924

C -2.422379 0.673724 1.651192

H -2.013762 1.078577 2.583378

C -1.86537 1.576385 0.501883

H -2.440037 2.493447 0.642287

C -2.223218 1.071204 -0.889734

C -3.530245 1.254962 -1.359925

C -1.321821 0.418911 -1.734683

C -3.937933 0.777155 -2.599424

H -4.246345 1.798931 -0.750086

C -1.710013 -0.059409 -2.982035

H -0.290552 0.278345 -1.43062

C -3.024847 0.108502 -3.412978

H -4.95461 0.927564 -2.947403

H -0.991596 -0.563072 -3.622871

O -3.825761 0.827281 1.772145

O -3.464673 -0.348623 -4.624763

O -1.138127 4.224827 0.478609

O 3.427738 3.349294 1.656579

O 6.565609 -1.135624 -1.749729

H -2.736372 -0.788767 -5.079427

H -0.811848 5.129442 0.552748

H 3.455192 4.31337 1.67651

H 6.652331 -0.421282 -2.393065

H -4.238188 0.412351 1.003411

O -3.524694 -4.089534 1.850717

H -3.161165 -4.97305 1.715731

MalibatolA_Conf28.log

Energy (E) = -1605.16491592 Hartree

Enthalpy (H) = -1604.709411 Hartree

Gibbs free energy (G) = -1604.797928 Hartree

Charge = 0, Spin = 1

C 3.042285 2.461764 1.964741

C 1.780339 3.002716 1.746562

C 0.794085 2.090024 1.39763

C 0.998714 0.71986 1.28193

C 2.286301 0.202002 1.471856

C 3.296283 1.083901 1.81858

H 1.586184 4.066367 1.828913

H 4.30956 0.729399 1.962944

C -0.273779 0.145699 0.905063

C -1.125608 1.204958 0.768859

O -0.496492 2.396465 1.087111

C -2.503696 1.347285 0.295974

C -2.966741 0.585275 -0.78536

C -3.370617 2.271587 0.887599

C -4.265124 0.724253 -1.24821

H -2.297515 -0.116678 -1.272526

C -4.671725 2.423665 0.425986

H -3.02798 2.875154 1.721722

C -5.122645 1.645168 -0.640719

H -4.623094 0.136624 -2.086733

H -5.340582 3.140401 0.893124

C -0.586861 -1.291671 0.775146

C -1.84627 -1.733387 1.184444

C 0.367173 -2.198595 0.264177

C -2.198823 -3.071049 1.043358

H -2.560667 -1.042374 1.615537

C -0.039977 -3.532994 0.100147

C -1.30409 -3.973859 0.486037

H -1.572465 -5.0159 0.344352

C 2.521784 -1.266213 1.230738

H 2.095203 -1.832063 2.069994

C 1.793261 -1.782807 -0.058968

H 2.361978 -2.691266 -0.285114

C 1.920514 -0.865996 -1.274018

C 3.145736 -0.263038 -1.586093

C 0.841446 -0.61238 -2.121878

C 3.283597 0.575915 -2.683708

H 4.007159 -0.44703 -0.9539

C 0.963022 0.223294 -3.229016

H -0.122483 -1.069515 -1.92267

C 2.186726 0.825599 -3.508808

H 4.237337 1.04175 -2.90973

H 0.106994 0.410309 -3.871516

O 3.918025 -1.498622 1.177299

O 2.369344 1.663275 -4.575744

O 0.764476 -4.504321 -0.434793

O -3.4435 -3.444457 1.460279

O -6.388545 1.747837 -1.135017

H 1.537828 1.746266 -5.057864

H 1.490418 -4.108947 -0.92995

H -3.556085 -4.391813 1.315644

H -6.872502 2.419905 -0.639379

H 4.054555 -2.452308 1.198618

O 4.112076 3.239288 2.312169

H 3.831221 4.160144 2.375815

MalibatolA_Conf29.log

Energy (E) = -1605.16695790 Hartree

Enthalpy (H) = -1604.711024 Hartree

Gibbs free energy (G) = -1604.797892 Hartree

Charge = 0, Spin = 1

C 3.058154 2.477006 1.954273

C 1.782516 2.99998 1.779098

C 0.805578 2.078664 1.427032

C 1.027581 0.713911 1.274736

C 2.330342 0.21451 1.41997

C 3.331019 1.109003 1.762007

H 1.571287 4.057593 1.891528

H 4.358814 0.778199 1.858967

C -0.245931 0.13405 0.900907

C -1.111819 1.185992 0.803705

O -0.493036 2.376364 1.143289

C -2.497725 1.324585 0.353313

C -2.965237 0.589896 -0.744806

C -3.367787 2.22005 0.983437

C -4.271328 0.726141 -1.186533

H -2.293168 -0.088007 -1.261452

C -4.676528 2.369366 0.543349

H -3.02145 2.802681 1.830834

C -5.132112 1.61701 -0.540079

H -4.633132 0.159592 -2.037816

H -5.34793 3.063531 1.040095

C -0.543092 -1.305084 0.757369

C -1.799009 -1.774096 1.153618

C 0.431013 -2.193765 0.263746

C -2.114407 -3.121036 1.023023

H -2.53646 -1.099249 1.571157

C 0.079158 -3.547261 0.15007

C -1.180362 -4.017092 0.515267

H -1.422354 -5.071884 0.415052

C 2.600591 -1.248783 1.134527

H 2.230452 -1.839963 1.982066

C 1.834146 -1.756717 -0.120217

H 2.376608 -2.663444 -0.398262

C 1.876133 -0.820212 -1.328078

C 3.028823 -0.086507 -1.63496

C 0.784992 -0.694465 -2.190052

C 3.083312 0.755313 -2.738427

H 3.901094 -0.165895 -0.99567

C 0.823672 0.140962 -3.30307

H -0.124603 -1.25357 -1.994988

C 1.975184 0.874621 -3.576914

H 3.981432 1.323555 -2.958126

H -0.041107 0.2256 -3.955651

O 3.9813 -1.526569 0.953396

O 2.075071 1.721004 -4.64874

O 1.018144 -4.410571 -0.339009

O -3.358647 -3.520178 1.418351

O -6.405972 1.718061 -1.0139

H 1.245672 1.709583 -5.141568

H 0.653444 -5.303257 -0.354429

H -3.440948 -4.473709 1.296224

H -6.894168 2.361573 -0.485537

H 4.411401 -1.474639 1.81432

O 4.122593 3.264016 2.296508

H 3.828206 4.178451 2.386227

MalibatolA_Conf30.log

Energy (E) = -1605.16499180 Hartree

Enthalpy (H) = -1604.709357 Hartree

Gibbs free energy (G) = -1604.797776 Hartree

Charge = 0, Spin = 1

C 3.045236 2.465705 1.949448

C 1.784922 3.01019 1.728765

C 0.795157 2.100411 1.388731

C 0.993484 0.727374 1.282783

C 2.27664 0.205036 1.477817

C 3.292513 1.085521 1.817022

H 1.602867 4.07533 1.802858

H 4.301763 0.716357 1.96376

C -0.280879 0.156315 0.907468

C -1.127709 1.218531 0.763488

O -0.494615 2.409504 1.07519

C -2.50489 1.362145 0.288181

C -3.373033 2.289486 0.877143

C -2.967346 0.595846 -0.788288

C -4.673844 2.437037 0.41773

H -3.028905 2.896788 1.707959

C -4.268242 0.730371 -1.249585

H -2.298711 -0.107966 -1.273546

C -5.125951 1.651763 -0.64409

H -5.348714 3.151711 0.876325

H -4.621091 0.134041 -2.085849

C -0.599008 -1.280022 0.780213

C -1.863991 -1.715397 1.184674

C 0.350672 -2.192145 0.274878

C -2.22394 -3.050368 1.0403

H -2.571953 -1.01495 1.61447

C -0.064361 -3.525803 0.108883

C -1.331587 -3.958844 0.487193

H -1.61253 -4.996255 0.345853

C 2.50433 -1.266384 1.25077

H 2.067512 -1.822577 2.091129

C 1.781647 -1.78725 -0.040691

H 2.345654 -2.701135 -0.256167

C 1.925509 -0.879168 -1.260363

C 3.163522 -0.304909 -1.576297

C 0.851368 -0.605059 -2.108025

C 3.319163 0.525275 -2.678069

H 4.021086 -0.504821 -0.943743

C 0.990511 0.222666 -3.219193

H -0.122279 -1.039807 -1.906181

C 2.227303 0.795348 -3.50344

H 4.282948 0.968186 -2.907502

H 0.138122 0.425848 -3.861572

O 3.899321 -1.508567 1.210274

O 2.428061 1.622634 -4.575113

O 0.739278 -4.500045 -0.422513

O -3.448647 -3.51894 1.419387

O -6.412884 1.826619 -1.056706

H 1.598974 1.719265 -5.058797

H 1.46577 -4.107118 -0.918773

H -3.978868 -2.785617 1.754653

H -6.599882 1.221804 -1.785329

H 4.029549 -2.462894 1.241529

O 4.04849 3.332519 2.28379

H 4.873474 2.841997 2.379766

MalibatolA_Conf31.log

Energy (E) = -1605.16505093 Hartree

Enthalpy (H) = -1604.709423 Hartree

Gibbs free energy (G) = -1604.797769 Hartree

Charge = 0, Spin = 1

C 3.050694 2.465204 1.946866

C 1.792313 3.01151 1.721333

C 0.801061 2.101308 1.380075

C 0.997995 0.728952 1.278516

C 2.281901 0.205101 1.476483

C 3.296756 1.084332 1.815385

H 1.604494 4.077062 1.79293

H 4.307625 0.725373 1.965503

C -0.276959 0.158287 0.905325

C -1.12275 1.220961 0.757464

O -0.487544 2.411938 1.064894

C -2.499784 1.36439 0.28182

C -3.365992 2.29725 0.864912

C -2.96441 0.59135 -0.788921

C -4.666857 2.443908 0.405298

H -3.020327 2.909598 1.691378

C -4.265416 0.724822 -1.250182

H -2.297469 -0.117492 -1.269222

C -5.121097 1.651969 -0.650641

H -5.340229 3.16287 0.859388

H -4.620028 0.123117 -2.081846

C -0.597191 -1.27817 0.78471

C -1.86207 -1.710295 1.192912

C 0.350864 -2.193492 0.282422

C -2.22345 -3.045578 1.055289

H -2.56865 -1.007196 1.620632

C -0.065656 -3.527477 0.123003

C -1.332795 -3.957492 0.505069

H -1.614986 -4.995256 0.368922

C 2.506821 -1.267379 1.252598

H 2.069781 -1.8204 2.095004

C 1.781769 -1.791057 -0.036394

H 2.344238 -2.706318 -0.250069

C 1.924328 -0.887236 -1.259386

C 3.160529 -0.309539 -1.576034

C 0.850379 -0.621241 -2.109955

C 3.314548 0.516145 -2.68144

H 4.017967 -0.503179 -0.941361

C 0.987969 0.201723 -3.224824

H -0.121875 -1.058817 -1.907431

C 2.22298 0.777913 -3.509826

H 4.276913 0.961858 -2.911404

H 0.135856 0.398477 -3.869583

O 3.901224 -1.512543 1.211184

O 2.4222 1.600995 -4.585057

O 0.736336 -4.504914 -0.405013

O -3.448071 -3.511111 1.438461

O -6.408053 1.826013 -1.063573

H 1.593669 1.691717 -5.070816

H 1.462314 -4.115027 -0.904445

H -3.976897 -2.775538 1.771002

H -6.596389 1.216589 -1.78801

H 4.029355 -2.467146 1.242846

O 4.124677 3.239609 2.288186

H 3.849622 4.162807 2.342664

MalibatolA_Conf32.log

Energy (E) = -1605.16498972 Hartree

Enthalpy (H) = -1604.709377 Hartree

Gibbs free energy (G) = -1604.797745 Hartree

Charge = 0, Spin = 1

C 3.044482 2.467802 1.963808

C 1.779611 3.004857 1.751122

C 0.796259 2.091112 1.403533

C 1.00439 0.720588 1.283051

C 2.292629 0.206632 1.466606

C 3.30211 1.091418 1.814353

H 1.589504 4.067733 1.836738

H 4.314803 0.728456 1.952706

C -0.267634 0.14418 0.907212

C -1.122054 1.201758 0.775071

O -0.496239 2.39415 1.095827

C -2.500619 1.341378 0.302985

C -2.961146 0.580747 -0.780427

C -3.370011 2.262027 0.896692

C -4.259689 0.717556 -1.243413

H -2.289483 -0.117572 -1.269489

C -4.671304 2.411881 0.434955

H -3.029056 2.864567 1.732247

C -5.11966 1.635029 -0.634094

H -4.615671 0.1316 -2.083944

H -5.342125 3.125965 0.90332

C -0.578175 -1.293544 0.778637

C -1.838663 -1.736738 1.188447

C 0.375705 -2.199498 0.270575

C -2.189584 -3.074587 1.047907

H -2.549774 -1.040404 1.619767

C -0.029563 -3.536728 0.110213

C -1.292155 -3.977994 0.494869

H -1.566045 -5.017779 0.357198

C 2.536135 -1.25924 1.218047

H 2.124696 -1.831223 2.060672

C 1.797788 -1.77863 -0.064018

H 2.368062 -2.684852 -0.29543

C 1.906423 -0.86286 -1.282181

C 0.821904 -0.647133 -2.136366

C 3.112598 -0.226174 -1.594055

C 0.921527 0.183241 -3.247307

H -0.128137 -1.131873 -1.935636

C 3.228602 0.610737 -2.698115

H 3.978236 -0.379957 -0.959519

C 2.127388 0.821048 -3.527962

H 0.067636 0.34413 -3.897324

H 4.172591 1.101689 -2.918486

O 3.933728 -1.47944 1.146848

O 2.177903 1.63569 -4.626632

O 0.778446 -4.50682 -0.421958

O -3.409672 -3.551255 1.431769

O -6.385339 1.736106 -1.128966

H 3.063378 2.010665 -4.702965

H 1.50143 -4.11064 -0.920711

H -3.945076 -2.820788 1.764995

H -6.870286 2.407956 -0.633988

H 4.078783 -2.431883 1.166908

O 4.041483 3.33874 2.306276

H 4.870286 2.853653 2.396763

MalibatolA_Conf33.log

Energy (E) = -1605.16748926 Hartree

Enthalpy (H) = -1604.710713 Hartree

Gibbs free energy (G) = -1604.797658 Hartree

Charge = 0, Spin = 1

C -2.533033 -3.212698 1.552228

C -1.24729 -3.561206 1.155252

C -0.37714 -2.499581 0.961406

C -0.707925 -1.16036 1.154722

C -2.01644 -0.829454 1.524335

C -2.913951 -1.868797 1.725513

H -0.957491 -4.592975 0.99831

H -3.936409 -1.65051 2.016854

C 0.482966 -0.398141 0.849875

C 1.410161 -1.316404 0.443642

O 0.910448 -2.604071 0.530429

C 2.754586 -1.227409 -0.132182

C 3.750218 -2.14013 0.236233

C 3.055885 -0.252948 -1.09078

C 5.018253 -2.073161 -0.322627

H 3.532094 -2.906573 0.97278

C 4.322361 -0.173575 -1.650807

H 2.289651 0.448624 -1.404595

C 5.308589 -1.084719 -1.264691

H 5.791921 -2.776423 -0.033929

H 4.548458 0.584183 -2.395167

C 0.653932 1.062783 0.974343

C 1.924945 1.546149 1.312805

C -0.417592 1.951921 0.764361

C 2.164035 2.911075 1.381249

H 2.732776 0.854734 1.524502

C -0.129404 3.330102 0.828682

C 1.13899 3.815097 1.124656

H 1.326893 4.883288 1.169531

C -2.420871 0.619977 1.667987

H -2.016338 1.005942 2.609867

C -1.868151 1.551564 0.540696

H -2.445046 2.463229 0.704688

C -2.226227 1.08205 -0.863586

C -1.32461 0.447684 -1.724528

C -3.529412 1.284776 -1.331865

C -1.709862 0.00953 -2.985545

H -0.295587 0.292603 -1.42003

C -3.934927 0.846441 -2.588443

H -4.246158 1.813995 -0.709918

C -3.021507 0.199908 -3.418791

H -0.99792 -0.478954 -3.642896

H -4.952412 1.016698 -2.92902

O -3.825424 0.762896 1.790013

O -3.35124 -0.252068 -4.666807

O -1.151933 4.202169 0.582806

O 3.387941 3.424006 1.700772

O 6.569909 -1.057656 -1.779639

H -4.278496 -0.052176 -4.843188

H -0.82879 5.106131 0.676738

H 4.011593 2.70024 1.837794

H 6.646476 -0.332606 -2.412242

H -4.23384 0.371243 1.007024

O -3.421923 -4.231455 1.755552

H -4.273065 -3.864105 2.023037

MalibatolA_Conf34.log

Energy (E) = -1605.16485863 Hartree

Enthalpy (H) = -1604.709333 Hartree

Gibbs free energy (G) = -1604.797657 Hartree

Charge = 0, Spin = 1

C 3.039933 2.458255 1.96947

C 1.775954 2.997809 1.757935

C 0.79087 2.086347 1.409019

C 0.996799 0.715767 1.286188

C 2.283797 0.199073 1.470809

C 3.295082 1.08144 1.818996

H 1.587849 4.060909 1.845221

H 4.306954 0.716451 1.957976

C -0.275699 0.141955 0.907928

C -1.128598 1.20095 0.778128

O -0.501123 2.39187 1.101922

C -2.506974 1.344422 0.306339

C -2.969462 0.588145 -0.779225

C -3.374257 2.265331 0.902733

C -4.267708 0.729373 -1.241756

H -2.299834 -0.110819 -1.270115

C -4.675275 2.419485 0.441659

H -3.03192 2.8646 1.740068

C -5.125644 1.646744 -0.629494

H -4.625225 0.146394 -2.083709

H -5.344496 3.133536 0.912385

C -0.587005 -1.295102 0.770287

C -1.84567 -1.740535 1.177849

C 0.367832 -2.198105 0.253694

C -2.19688 -3.077749 1.029345

H -2.560581 -1.052721 1.613175

C -0.038017 -3.532021 0.082508

C -1.301513 -3.976376 0.466383

H -1.568883 -5.017851 0.318795

C 2.522128 -1.26748 1.222712

H 2.098313 -1.838471 2.059825

C 1.79322 -1.779083 -0.068644

H 2.363144 -2.685633 -0.299406

C 1.918388 -0.855842 -1.279033

C 3.14325 -0.251771 -1.590369

C 0.837501 -0.596396 -2.12281

C 3.279017 0.594024 -2.682976

H 4.006034 -0.440256 -0.96135

C 0.956926 0.246229 -3.224863

H -0.126203 -1.054255 -1.924215

C 2.180235 0.849825 -3.503651

H 4.232468 1.060799 -2.908288

H 0.099464 0.438006 -3.864034

O 3.918977 -1.495831 1.16618

O 2.360636 1.694425 -4.56545

O 0.767085 -4.499543 -0.458247

O -3.440899 -3.454947 1.444827

O -6.391337 1.751899 -1.123704

H 1.527487 1.782501 -5.043872

H 1.493046 -4.100702 -0.950553

H -3.552112 -4.401779 1.295697

H -6.875741 2.420981 -0.624467

H 4.058354 -2.449151 1.184931

O 4.038671 3.327013 2.312491

H 4.866424 2.840113 2.40281

MalibatolA_Conf35.log

Energy (E) = -1605.16725699 Hartree

Enthalpy (H) = -1604.710668 Hartree

Gibbs free energy (G) = -1604.797646 Hartree

Charge = 0, Spin = 1

C -2.542824 -3.175797 1.599873

C -1.257884 -3.534518 1.209412

C -0.384314 -2.478773 0.999261

C -0.711279 -1.135683 1.170815

C -2.01885 -0.794909 1.534666

C -2.919649 -1.828141 1.751895

H -0.971291 -4.569541 1.068864

H -3.941487 -1.60221 2.039504

C 0.481554 -0.381827 0.853461

C 1.406307 -1.309001 0.462635

O 0.903119 -2.593883 0.57031

C 2.75126 -1.231831 -0.113585

C 3.05438 -0.272817 -1.089469

C 3.744557 -2.138112 0.270411

C 4.319877 -0.204129 -1.649018

H 2.288222 0.423942 -1.414176

C 5.014856 -2.081275 -0.288686

H 3.526181 -2.892289 1.019435

C 5.306032 -1.108407 -1.245918

H 4.554744 0.537223 -2.405138

H 5.783594 -2.784297 0.01818

C 0.656722 1.080553 0.953703

C 1.926307 1.565976 1.287321

C -0.413829 1.969174 0.725744

C 2.167905 2.931598 1.335418

H 2.736586 0.883711 1.512571

C -0.123056 3.345967 0.765871

C 1.147002 3.833829 1.058502

H 1.330382 4.904678 1.082311

C -2.419108 0.657893 1.65407

H -2.012041 1.059135 2.588453

C -1.865325 1.568098 0.509512

H -2.440406 2.483792 0.657009

C -2.226179 1.071653 -0.884491

C -3.532991 1.262492 -1.35248

C -1.327763 0.420886 -1.733819

C -3.943464 0.792838 -2.594217

H -4.246628 1.805949 -0.739275

C -1.718729 -0.04917 -2.983391

H -0.296711 0.275085 -1.431529

C -3.03339 0.12556 -3.412222

H -4.959903 0.948724 -2.940477

H -1.002616 -0.551699 -3.627681

O -3.823109 0.80631 1.775701

O -3.475706 -0.323418 -4.626092

O -1.141362 4.217042 0.500557

O 3.428647 3.340234 1.661347

O 6.534505 -0.99749 -1.825043

H -2.749158 -0.76328 -5.083852

H -0.816154 5.121658 0.579282

H 3.455141 4.304222 1.686353

H 7.124457 -1.668353 -1.45969

H -4.233472 0.402105 1.000123

O -3.435132 -4.188436 1.818308

H -4.285894 -3.81419 2.077382

MalibatolA_Conf36.log

Energy (E) = -1605.16718941 Hartree

Enthalpy (H) = -1604.710578 Hartree

Gibbs free energy (G) = -1604.797635 Hartree

Charge = 0, Spin = 1

C -2.520532 -3.212876 1.569856

C -1.23524 -3.560352 1.170557

C -0.367933 -2.497725 0.969538

C -0.70104 -1.15835 1.157946

C -2.009175 -0.828693 1.530034

C -2.903858 -1.869076 1.738366

H -0.943664 -4.592187 1.017379

H -3.925927 -1.65175 2.031798

C 0.487439 -0.394978 0.846578

C 1.415463 -1.312994 0.441976

O 0.918658 -2.601389 0.535442

C 2.758836 -1.223171 -0.136156

C 3.055981 -0.252355 -1.102114

C 3.756758 -2.129264 0.23607

C 4.320266 -0.171844 -1.662938

H 2.286088 0.44414 -1.41836

C 5.025748 -2.060728 -0.324576

H 3.543044 -2.892648 0.977067

C 5.311188 -1.075955 -1.271322

H 4.550434 0.578796 -2.411297

H 5.797959 -2.763764 -0.02665

C 0.656279 1.066808 0.965009

C 1.924953 1.553809 1.299953

C -0.41923 1.953383 0.753213

C 2.160478 2.919774 1.365092

H 2.739218 0.872499 1.513294

C -0.134755 3.330914 0.810814

C 1.134187 3.820828 1.104702

H 1.312592 4.892119 1.142079

C -2.4166 0.620397 1.668763

H -2.010498 1.011312 2.607935

C -1.86964 1.548745 0.536052

H -2.447871 2.45983 0.69867

C -2.232333 1.072983 -0.864826

C -1.333714 0.434348 -1.725758

C -3.537086 1.273698 -1.32955

C -1.723404 -0.009828 -2.983295

H -0.303645 0.280657 -1.424027

C -3.947004 0.829372 -2.582603

H -4.251574 1.805977 -0.707596

C -3.036561 0.178599 -3.41288

H -1.013834 -0.501601 -3.640754

H -4.965644 0.998137 -2.920453

O -3.821224 0.760189 1.793766

O -3.370713 -0.279602 -4.657457

O -1.15847 4.200581 0.561917

O 3.420735 3.329973 1.690947

O 6.53835 -0.953072 -1.850898

H -4.298485 -0.080266 -4.831714

H -0.837742 5.105738 0.652102

H 3.442811 4.293657 1.72881

H 7.132019 -1.625425 -1.494291

H -4.230112 0.365301 1.012622

O -3.406645 -4.232626 1.780409

H -4.257514 -3.865749 2.049356

MalibatolA_Conf37.log

Energy (E) = -1605.16754538 Hartree

Enthalpy (H) = -1604.710763 Hartree

Gibbs free energy (G) = -1604.797632 Hartree

Charge = 0, Spin = 1

C -2.549109 -3.179446 1.581907

C -1.264053 -3.537377 1.191108

C -0.389275 -2.481377 0.987435

C -0.715128 -1.138888 1.165394

C -2.022791 -0.798744 1.529535

C -2.924797 -1.832204 1.7404

H -0.978174 -4.57192 1.045665

H -3.946746 -1.606716 2.027973

C 0.478839 -0.384548 0.853391

C 1.40333 -1.310754 0.45923

O 0.898602 -2.595534 0.559734

C 2.749638 -1.233611 -0.11385

C 3.739611 -2.147761 0.266177

C 3.058929 -0.269381 -1.080219

C 5.009833 -2.091916 -0.288824

H 3.515346 -2.906342 1.008978

C 4.327699 -0.201153 -1.636598

H 2.297315 0.433372 -1.402597

C 5.308308 -1.113385 -1.238676

H 5.779124 -2.796132 0.00909

H 4.559919 0.548847 -2.386883

C 0.654395 1.077204 0.960556

C 1.926102 1.560505 1.29642

C -0.413667 1.967193 0.736707

C 2.169551 2.925397 1.348586

H 2.731049 0.869075 1.518864

C -0.120891 3.345059 0.784072

C 1.148329 3.829506 1.077476

H 1.339665 4.897551 1.109363

C -2.421603 0.653833 1.655981

H -2.014996 1.049852 2.592768

C -1.86533 1.568941 0.516568

H -2.439603 2.484539 0.667606

C -2.224401 1.079669 -0.880501

C -3.530543 1.273153 -1.349296

C -1.324968 0.43311 -1.731956

C -3.939351 0.81014 -2.594063

H -4.244987 1.813558 -0.734352

C -1.714258 -0.030237 -2.984553

H -0.294397 0.285429 -1.428967

C -3.028233 0.147105 -3.414354

H -4.955273 0.968066 -2.940909

H -0.997357 -0.529475 -3.630536

O -3.825491 0.803381 1.777042

O -3.468767 -0.295042 -4.631366

O -1.139585 4.217361 0.523783

O 3.394196 3.438258 1.665374

O 6.57182 -1.097294 -1.748716

H -2.741529 -0.732333 -5.090509

H -0.813217 5.121263 0.60632

H 4.015081 2.714286 1.813377

H 6.655554 -0.378098 -2.387038

H -4.235908 0.403093 0.999481

O -3.442597 -4.192311 1.794116

H -4.293305 -3.818665 2.054238

MalibatolA_Conf38.log

Energy (E) = -1605.16483392 Hartree

Enthalpy (H) = -1604.709279 Hartree

Gibbs free energy (G) = -1604.797585 Hartree

Charge = 0, Spin = 1

C 3.052203 2.45003 1.976935

C 1.787852 2.990766 1.770637

C 0.802431 2.081339 1.417529

C 1.007968 0.711385 1.286255

C 2.295997 0.194185 1.462349

C 3.307483 1.074648 1.815189

H 1.599475 4.053178 1.865388

H 4.319939 0.708998 1.948115

C -0.265955 0.139844 0.909279

C -1.1187 1.199791 0.786374

O -0.490059 2.388852 1.114462

C -2.497486 1.345761 0.316972

C -2.961705 0.591868 -0.769563

C -3.362871 2.267228 0.915215

C -4.259775 0.736314 -1.231532

H -2.293209 -0.107119 -1.261991

C -4.663714 2.424544 0.454705

H -3.01913 2.864638 1.753301

C -5.115709 1.654574 -0.617772

H -4.618526 0.155594 -2.074516

H -5.331404 3.139213 0.926679

C -0.579308 -1.296827 0.7733

C -1.836421 -1.741047 1.186808

C 0.373231 -2.20094 0.254779

C -2.188158 -3.078678 1.042993

H -2.549563 -1.052182 1.623402

C -0.032447 -3.535618 0.089838

C -1.294434 -3.978952 0.480132

H -1.562073 -5.020972 0.336921

C 2.537646 -1.269718 1.200577

H 2.129315 -1.84862 2.039973

C 1.793941 -1.778148 -0.082857

H 2.363267 -2.682431 -0.324217

C 1.898013 -0.852329 -1.293739

C 0.809919 -0.628709 -2.141279

C 3.103269 -0.21387 -1.605538

C 0.905215 0.210799 -3.245702

H -0.139508 -1.114542 -1.94026

C 3.215076 0.63194 -2.703222

H 3.971507 -0.37342 -0.975989

C 2.110365 0.849919 -3.526437

H 0.048537 0.377834 -3.890482

H 4.158444 1.124042 -2.923725

O 3.934837 -1.490143 1.121846

O 2.156696 1.673508 -4.618621

O 0.770294 -4.504939 -0.450972

O -3.430786 -3.454673 1.463672

O -6.38112 1.763306 -1.111925

H 3.042538 2.047251 -4.696847

H 1.496792 -4.108154 -0.944131

H -3.543132 -4.401646 1.316278

H -6.863401 2.434408 -0.61334

H 4.079483 -2.442736 1.136611

O 4.051213 3.316313 2.325455

H 4.878483 2.828276 2.414144

MalibatolA_Conf39.log

Energy (E) = -1605.16490165 Hartree

Enthalpy (H) = -1604.709299 Hartree

Gibbs free energy (G) = -1604.797472 Hartree

Charge = 0, Spin = 1

C 3.05573 2.451222 1.972564

C 1.793163 2.993527 1.76131

C 0.806249 2.08321 1.408299

C 1.01043 0.713732 1.282713

C 2.299144 0.19518 1.462945

C 3.309647 1.074825 1.81427

H 1.599012 4.05652 1.851759

H 4.32352 0.719242 1.951729

C -0.26371 0.142281 0.907047

C -1.115359 1.202693 0.779136

O -0.484962 2.392019 1.102754

C -2.493805 1.34822 0.30865

C -2.958357 0.590387 -0.775006

C -3.358894 2.272278 0.903315

C -4.256425 0.733425 -1.237442

H -2.29014 -0.110707 -1.264803

C -4.659668 2.428384 0.442133

H -3.01501 2.872658 1.739222

C -5.112029 1.654342 -0.627235

H -4.615414 0.149464 -2.078085

H -5.327081 3.145093 0.911395

C -0.578923 -1.294585 0.777441

C -1.83672 -1.735361 1.192512

C 0.372724 -2.202197 0.263586

C -2.189891 -3.073272 1.054968

H -2.549277 -1.043618 1.625496

C -0.034305 -3.537215 0.104889

C -1.296915 -3.977283 0.496893

H -1.565647 -5.019708 0.358739

C 2.538054 -1.27018 1.206493

H 2.127863 -1.844991 2.047846

C 1.793993 -1.782394 -0.07525

H 2.36208 -2.68833 -0.313245

C 1.89939 -0.861705 -1.289932

C 0.81256 -0.64307 -2.140405

C 3.104622 -0.223683 -1.602647

C 0.908981 0.191377 -3.248566

H -0.136773 -1.128841 -1.938768

C 3.217511 0.617164 -2.704049

H 3.971983 -0.379564 -0.970981

C 2.114039 0.8303 -3.530164

H 0.053282 0.354534 -3.89564

H 4.160837 1.109045 -2.925185

O 3.934736 -1.494063 1.129342

O 2.161559 1.648934 -4.62605

O 0.767616 -4.509987 -0.430937

O -3.433181 -3.44575 1.476864

O -6.377497 1.761496 -1.121688

H 3.047195 2.023173 -4.704143

H 1.494826 -4.116365 -0.925594

H -3.5468 -4.393189 1.333576

H -6.859476 2.434734 -0.625708

H 4.076993 -2.446988 1.145816

O 4.126184 3.226326 2.323464

H 3.844984 4.146433 2.395774

MalibatolA_Conf40.log

Energy (E) = -1605.16489170 Hartree

Enthalpy (H) = -1604.709296 Hartree

Gibbs free energy (G) = -1604.797461 Hartree

Charge = 0, Spin = 1

C 3.061073 2.446979 1.957745

C 1.803023 2.997005 1.739562

C 0.809457 2.091099 1.393656

C 1.003617 0.719166 1.281043

C 2.287393 0.191958 1.470943

C 3.304481 1.06688 1.814397

H 1.617063 4.06226 1.819897

H 4.315092 0.704771 1.958656

C -0.273428 0.153617 0.906912

C -1.117431 1.218941 0.768914

O -0.4792 2.406503 1.083706

C -2.49504 1.369198 0.297102

C -3.357856 2.299559 0.889115

C -2.963091 0.606866 -0.779759

C -4.658738 2.454016 0.432172

H -3.009504 2.903777 1.720421

C -4.264156 0.748242 -1.238593

H -2.298578 -0.099124 -1.267502

C -5.116497 1.672614 -0.630056

H -5.32951 3.170964 0.893245

H -4.621304 0.154792 -2.075068

C -0.597034 -1.281723 0.779318

C -1.859197 -1.713897 1.190276

C 0.349646 -2.196105 0.267949

C -2.222161 -3.048771 1.049587

H -2.568042 -1.017399 1.621652

C -0.067615 -3.527592 0.10511

C -1.334774 -3.958707 0.492026

H -1.611327 -4.998689 0.350899

C 2.511018 -1.278637 1.233464

H 2.079202 -1.838983 2.073719

C 1.778933 -1.791936 -0.05565

H 2.340419 -2.705391 -0.279773

C 1.914654 -0.878262 -1.272084

C 3.147618 -0.29373 -1.588731

C 0.836584 -0.609162 -2.116441

C 3.294351 0.542004 -2.687624

H 4.008229 -0.489801 -0.959085

C 0.966833 0.223863 -3.224627

H -0.133308 -1.051939 -1.913871

C 2.198539 0.807291 -3.509252

H 4.254208 0.993199 -2.917397

H 0.111468 0.423214 -3.864276

O 3.905322 -1.522752 1.181621

O 2.390184 1.640829 -4.577807

O 0.729152 -4.505238 -0.429795

O -3.469718 -3.412078 1.466921

O -6.403438 1.85451 -1.039928

H 1.558718 1.734509 -5.058031

H 1.458574 -4.115696 -0.924488

H -3.590783 -4.358161 1.320914

H -6.595612 1.250609 -1.767936

H 4.034164 -2.477442 1.206446

O 4.137185 3.216947 2.302504

H 3.863749 4.140122 2.364952

MalibatolA_Conf41.log

Energy (E) = -1605.16721655 Hartree

Enthalpy (H) = -1604.710542 Hartree

Gibbs free energy (G) = -1604.797409 Hartree

Charge = 0, Spin = 1

C -2.553986 -3.164118 1.604815

C -1.268752 -3.526848 1.219129

C -0.392229 -2.473662 1.008392

C -0.716434 -1.129325 1.175053

C -2.024381 -0.784593 1.533893

C -2.928125 -1.815129 1.751639

H -0.983991 -4.562883 1.082373

H -3.950265 -1.585972 2.035582

C 0.479077 -0.379172 0.858765

C 1.402724 -1.309735 0.473102

O 0.895938 -2.593032 0.583278

C 2.749149 -1.239895 -0.100494

C 3.735253 -2.157205 0.281959

C 3.061554 -0.281748 -1.071886

C 5.004601 -2.110559 -0.275847

H 3.508608 -2.91118 1.028702

C 4.329456 -0.222878 -1.631295

H 2.302954 0.423419 -1.396091

C 5.306142 -1.138337 -1.231169

H 5.770863 -2.817261 0.023983

H 4.563911 0.522331 -2.385635

C 0.656747 1.083203 0.95538

C 1.925881 1.567278 1.292643

C -0.411322 1.973118 0.720866

C 2.169683 2.932587 1.338017

H 2.734095 0.884122 1.522583

C -0.11834 3.349516 0.758629

C 1.151429 3.835896 1.05484

H 1.336596 4.906486 1.076656

C -2.422112 0.669401 1.646715

H -2.017063 1.073455 2.580753

C -1.862674 1.573867 0.500329

H -2.436797 2.491112 0.641726

C -2.218873 1.072086 -0.892976

C -3.524207 1.260901 -1.365897

C -1.317643 0.418318 -1.736991

C -3.930633 0.786398 -2.607117

H -4.239933 1.806482 -0.757026

C -1.70452 -0.056617 -2.986

H -0.287542 0.273837 -1.430876

C -3.017832 0.116196 -3.419685

H -4.946013 0.940688 -2.957176

H -0.986255 -0.561505 -3.626042

O -3.826146 0.821131 1.763595

O -3.456188 -0.337505 -4.633234

O -1.134037 4.22173 0.487185

O 3.429919 3.340017 1.667454

O 6.568625 -1.131809 -1.744168

H -2.728162 -0.779183 -5.086877

H -0.807425 5.125907 0.565073

H 3.457768 4.304025 1.690191

H 6.655425 -0.415864 -2.385714

H -4.23528 0.413569 0.989141

O -3.449248 -4.174047 1.823598

H -4.300211 -3.79714 2.07815

MalibatolA_Conf42.log

Energy (E) = -1605.16451887 Hartree

Enthalpy (H) = -1604.708891 Hartree

Gibbs free energy (G) = -1604.797366 Hartree

Charge = 0, Spin = 1

C 3.046279 2.498247 1.913358

C 1.78147 3.034489 1.700195

C 0.792743 2.115812 1.373976

C 0.996854 0.743835 1.281463

C 2.285924 0.229363 1.469748

C 3.299426 1.118419 1.78481

H 1.587513 4.099266 1.766588

H 4.318918 0.772031 1.911879

C -0.275305 0.163392 0.911796

C -1.127207 1.220828 0.760226

O -0.49825 2.417069 1.061115

C -2.504515 1.358387 0.283168

C -2.963642 0.590214 -0.795412

C -3.37359 2.285469 0.867105

C -4.260424 0.726601 -1.263494

H -2.292248 -0.113933 -1.276421

C -4.673193 2.434847 0.400309

H -3.033783 2.893543 1.69913

C -5.120012 1.650928 -0.664108

H -4.615384 0.134692 -2.100275

H -5.34379 3.153898 0.861338

C -0.589249 -1.275588 0.797785

C -1.853451 -1.709216 1.20087

C 0.365939 -2.19283 0.307279

C -2.210291 -3.046834 1.06966

H -2.569401 -1.011853 1.61882

C -0.044832 -3.526868 0.151681

C -1.314417 -3.959024 0.530066

H -1.586063 -5.001193 0.395799

C 2.514439 -1.250787 1.259812

H 2.077892 -1.791706 2.109052

C 1.795596 -1.786026 -0.016119

H 2.36836 -2.696534 -0.218364

C 1.921344 -0.89404 -1.250426

C 0.857657 -0.724134 -2.140128

C 3.123101 -0.241321 -1.544752

C 0.973505 0.076539 -3.271239

H -0.088364 -1.22156 -1.951308

C 3.25503 0.566209 -2.668591

H 3.973309 -0.357066 -0.881351

C 2.174666 0.730072 -3.535637

H 0.135949 0.202286 -3.949648

H 4.195004 1.070538 -2.87518

O 3.889431 -1.586642 1.160342

O 2.241753 1.514165 -4.655296

O 0.760226 -4.505858 -0.367663

O -3.46074 -3.410203 1.478132

O -6.383923 1.751414 -1.163951

H 3.11987 1.909175 -4.713305

H 1.495978 -4.117502 -0.853925

H -3.577872 -4.357652 1.338043

H -6.868314 2.428862 -0.676145

H 4.276473 -1.513933 2.040203

O 4.11945 3.281705 2.235708

H 3.839313 4.203544 2.287554

MalibatolA_Conf43.log

Energy (E) = -1605.16482602 Hartree

Enthalpy (H) = -1604.709230 Hartree

Gibbs free energy (G) = -1604.797336 Hartree

Charge = 0, Spin = 1

C 3.057404 2.4456 1.961721

C 1.797497 2.994089 1.748893

C 0.805428 2.088949 1.403196

C 1.000995 0.716472 1.284809

C 2.284148 0.190625 1.470474

C 3.302184 1.066478 1.815216

H 1.617265 4.058775 1.833304

H 4.311296 0.69436 1.955305

C -0.275753 0.150806 0.909167

C -1.120894 1.215708 0.776277

O -0.48455 2.403015 1.095787

C -2.498878 1.36647 0.305653

C -3.362177 2.293578 0.902043

C -2.966452 0.608927 -0.774769

C -4.663151 2.449353 0.4459

H -3.014104 2.894228 1.736036

C -4.267564 0.751777 -1.233001

H -2.301488 -0.094212 -1.265984

C -5.120436 1.672777 -0.620075

H -5.334295 3.163781 0.910322

H -4.624268 0.162169 -2.072367

C -0.597362 -1.284352 0.774817

C -1.859006 -1.720063 1.183659

C 0.350383 -2.195174 0.258895

C -2.220508 -3.054609 1.036316

H -2.568692 -1.026446 1.618285

C -0.065449 -3.526295 0.089489

C -1.332135 -3.960737 0.474177

H -1.607498 -5.000309 0.327795

C 2.510754 -1.278538 1.227551

H 2.080873 -1.843281 2.065789

C 1.779148 -1.78796 -0.063346

H 2.342027 -2.699695 -0.290955

C 1.913624 -0.868891 -1.275859

C 3.146677 -0.284161 -1.591876

C 0.834294 -0.59416 -2.116726

C 3.292276 0.557267 -2.686549

H 4.008209 -0.484538 -0.964853

C 0.963368 0.244673 -3.220655

H -0.13571 -1.036906 -1.914674

C 2.195105 0.82838 -3.50449

H 4.252202 1.008597 -2.915775

H 0.106935 0.448519 -3.857445

O 3.905696 -1.518973 1.173926

O 2.385468 1.667564 -4.56882

O 0.732389 -4.500378 -0.45029

O -3.467606 -3.421479 1.451856

O -6.407449 1.856049 -1.02905

H 1.55271 1.766007 -5.045873

H 1.461431 -4.107502 -0.942866

H -3.587448 -4.366985 1.30116

H -6.599393 1.255753 -1.76008

H 4.037118 -2.473354 1.196578

O 4.062955 3.307374 2.302338

H 4.886482 2.813744 2.394881

MalibatolA_Conf44.log

Energy (E) = -1605.16504144 Hartree

Enthalpy (H) = -1604.709324 Hartree

Gibbs free energy (G) = -1604.797278 Hartree

Charge = 0, Spin = 1

C 3.071679 2.448583 1.956364

C 1.812598 2.997369 1.740801

C 0.820071 2.090842 1.393868

C 1.01594 0.719296 1.278002

C 2.301177 0.193792 1.462418

C 3.317073 1.069515 1.807807

H 1.625109 4.062139 1.823957

H 4.328641 0.708586 1.948282

C -0.261479 0.152957 0.906418

C -1.106806 1.21757 0.771102

O -0.469476 2.405312 1.08622

C -2.484959 1.365623 0.300591

C -3.349848 2.293188 0.89395

C -2.951299 0.603125 -0.77694

C -4.651214 2.444814 0.437484

H -3.002646 2.897536 1.725648

C -4.252883 0.741561 -1.235151

H -2.284582 -0.09985 -1.266071

C -5.107251 1.663361 -0.625514

H -5.323582 3.159863 0.899172

H -4.60864 0.148405 -2.072423

C -0.585161 -1.282623 0.785738

C -1.847692 -1.713148 1.202584

C 0.359085 -2.199136 0.278894

C -2.21027 -3.048671 1.070294

H -2.551241 -1.008792 1.633251

C -0.057948 -3.533697 0.126555

C -1.322794 -3.962386 0.518079

H -1.605869 -5.000545 0.386883

C 2.530308 -1.275016 1.216985

H 2.115702 -1.840688 2.062356

C 1.783709 -1.791874 -0.061364

H 2.345063 -2.703999 -0.291414

C 1.896675 -0.881559 -1.283211

C 0.81253 -0.663226 -2.137225

C 3.106171 -0.253204 -1.59901

C 0.915683 0.161986 -3.251669

H -0.140024 -1.141762 -1.933512

C 3.225763 0.57849 -2.706654

H 3.971668 -0.409399 -0.964884

C 2.12486 0.791787 -3.536145

H 0.062032 0.325123 -3.901447

H 4.172319 1.06313 -2.929931

O 3.925518 -1.508792 1.142551

O 2.178694 1.601755 -4.638142

O 0.739926 -4.513121 -0.403778

O -3.432885 -3.512669 1.461685

O -6.394615 1.842613 -1.034991

H 3.066023 1.971827 -4.716661

H 1.465305 -4.125522 -0.905814

H -3.959162 -2.775939 1.795699

H -6.584617 1.240883 -1.765374

H 4.061607 -2.462518 1.164994

O 4.146931 3.219159 2.30245

H 3.871831 4.141541 2.369034

MalibatolA_Conf45.log

Energy (E) = -1605.16497293 Hartree

Enthalpy (H) = -1604.709253 Hartree

Gibbs free energy (G) = -1604.797200 Hartree

Charge = 0, Spin = 1

C 3.067717 2.446826 1.961115

C 1.806879 2.994119 1.750518

C 0.815922 2.088437 1.403354

C 1.013195 0.716382 1.281592

C 2.297746 0.192203 1.462083

C 3.314517 1.068771 1.809215

H 1.625134 4.058302 1.837962

H 4.324596 0.697916 1.945675

C -0.263928 0.149984 0.908304

C -1.110353 1.214198 0.778125

O -0.474878 2.401643 1.098108

C -2.488923 1.362867 0.308956

C -3.353988 2.287524 0.906582

C -2.955191 0.604814 -0.771725

C -4.655541 2.44052 0.451193

H -3.00674 2.888527 1.740673

C -4.256923 0.74478 -1.229048

H -2.288323 -0.09575 -1.264092

C -5.111502 1.66358 -0.615149

H -5.328066 3.153304 0.916137

H -4.612612 0.155198 -2.068861

C -0.585668 -1.285422 0.780635

C -1.847813 -1.719492 1.195008

C 0.359763 -2.198328 0.269292

C -2.208902 -3.054727 1.055799

H -2.552361 -1.018034 1.628776

C -0.055704 -3.53263 0.110417

C -1.320181 -3.964665 0.499399

H -1.602056 -5.002469 0.362895

C 2.529734 -1.275141 1.211127

H 2.116679 -1.84515 2.054278

C 1.783876 -1.787806 -0.069343

H 2.346695 -2.698085 -0.303129

C 1.895841 -0.871677 -1.286932

C 0.810411 -0.646662 -2.137523

C 3.105826 -0.243679 -1.601674

C 0.912832 0.184568 -3.247554

H -0.142586 -1.124632 -1.934619

C 3.22475 0.593897 -2.704923

H 3.97221 -0.404844 -0.970002

C 2.122539 0.8138 -3.530987

H 0.058148 0.352924 -3.894631

H 4.171702 1.078137 -2.927434

O 3.925503 -1.505478 1.135454

O 2.175473 1.629785 -4.628554

O 0.743346 -4.508623 -0.424417

O -3.431135 -3.522176 1.444224

O -6.399014 1.844228 -1.02345

H 3.063418 1.99829 -4.707557

H 1.468778 -4.117878 -0.9239

H -3.958662 -2.787848 1.781524

H -6.589078 1.245878 -1.75658

H 4.063947 -2.458905 1.155907

O 4.072131 3.308972 2.304127

H 4.896188 2.816077 2.395921

MalibatolA_Conf46.log

Energy (E) = -1605.16715756 Hartree

Enthalpy (H) = -1604.710331 Hartree

Gibbs free energy (G) = -1604.797135 Hartree

Charge = 0, Spin = 1

C -2.539006 -3.195226 1.578001

C -1.253226 -3.549347 1.186199

C -0.381001 -2.490911 0.984311

C -0.709698 -1.149456 1.165373

C -2.018379 -0.813168 1.529543

C -2.917949 -1.849198 1.738535

H -0.964899 -4.582923 1.038722

H -3.940531 -1.626586 2.02611

C 0.482948 -0.391975 0.855585

C 1.408973 -1.315261 0.458326

O 0.906817 -2.601348 0.555772

C 2.753555 -1.23442 -0.118164

C 3.74488 -2.150619 0.253222

C 3.058558 -0.266561 -1.082255

C 5.012189 -2.093655 -0.308327

H 3.523905 -2.912076 0.994062

C 4.324338 -0.197339 -1.64521

H 2.295701 0.437655 -1.398377

C 5.306223 -1.11199 -1.256279

H 5.782478 -2.799645 -0.017265

H 4.553147 0.555225 -2.393984

C 0.656404 1.069737 0.968544

C 1.924916 1.553744 1.308353

C -0.41487 1.959043 0.74694

C 2.164681 2.919169 1.369085

H 2.735736 0.870401 1.528338

C -0.126098 3.335836 0.800474

C 1.142853 3.822607 1.099446

H 1.324754 4.893436 1.133424

C -2.421423 0.638049 1.658273

H -2.01851 1.032912 2.597161

C -1.865211 1.557905 0.523014

H -2.44178 2.471638 0.676446

C -2.22027 1.074472 -0.877261

C -1.316381 0.432855 -1.730382

C -3.522857 1.270988 -1.3498

C -1.699015 -0.01858 -2.98748

H -0.287651 0.28229 -1.422694

C -3.925824 0.819318 -2.602501

H -4.241244 1.805607 -0.734397

C -3.010278 0.165346 -3.424697

H -0.985373 -0.512826 -3.638665

H -4.943026 0.984666 -2.946303

O -3.826133 0.78343 1.775849

O -3.337061 -0.300486 -4.668421

O -1.145274 4.20801 0.54203

O 3.424552 3.326689 1.699797

O 6.566718 -1.09528 -1.773882

H -4.264487 -0.104963 -4.848717

H -0.821535 5.112334 0.629714

H 3.449398 4.290456 1.733744

H 6.64711 -0.373578 -2.409804

H -4.233116 0.384115 0.995973

O -3.430009 -4.210631 1.788891

H -4.281142 -3.839398 2.051017

MalibatolA_Conf47.log

Energy (E) = -1605.16519078 Hartree

Enthalpy (H) = -1604.709037 Hartree

Gibbs free energy (G) = -1604.797063 Hartree

Charge = 0, Spin = 1

C -2.463456 -3.326376 1.407913

C -1.1773 -3.641475 0.984283

C -0.317255 -2.563456 0.842529

C -0.659484 -1.240559 1.110462

C -1.965962 -0.942245 1.509275

C -2.854697 -1.99744 1.658247

H -0.880228 -4.660898 0.770628

H -3.876116 -1.804137 1.97013

C 0.518995 -0.448982 0.838299

C 1.453516 -1.333937 0.376875

O 0.968204 -2.630176 0.398607

C 2.792912 -1.202352 -0.202747

C 3.794548 -2.132407 0.100036

C 3.08221 -0.17067 -1.103452

C 5.05664 -2.028171 -0.466758

H 3.585757 -2.942748 0.79087

C 4.342517 -0.053949 -1.670432

H 2.310963 0.546327 -1.36557

C 5.334542 -0.984143 -1.350617

H 5.834744 -2.745573 -0.229295

H 4.559579 0.748085 -2.369771

C 0.67403 1.00799 1.020764

C 1.937519 1.487952 1.378329

C -0.404141 1.900205 0.828843

C 2.174858 2.853202 1.471999

H 2.749793 0.801038 1.581573

C -0.10635 3.275653 0.87255

C 1.159095 3.757624 1.191239

H 1.333757 4.82832 1.218282

C -2.368268 0.493663 1.74743

H -1.93348 0.827349 2.695716

C -1.861881 1.49478 0.65715

H -2.449615 2.377143 0.926356

C -2.287238 1.118095 -0.754809

C -1.440138 0.477767 -1.665799

C -3.589992 1.40851 -1.17408

C -1.880259 0.116423 -2.932452

H -0.413074 0.258678 -1.394443

C -4.048702 1.049426 -2.437727

H -4.262582 1.939013 -0.505112

C -3.191665 0.393891 -3.319043

H -1.21332 -0.378931 -3.630457

H -5.064043 1.286065 -2.74298

O -3.767595 0.627727 1.917586

O -3.578782 0.014953 -4.57403

O -1.046507 4.243865 0.627185

O 3.430433 3.2548 1.824561

O 6.589646 -0.921702 -1.87767

H -4.501891 0.26164 -4.708424

H -1.759253 3.891274 0.082291

H 3.454256 4.218139 1.871468

H 6.653642 -0.160591 -2.467791

H -4.196935 0.307086 1.113495

O -3.341337 -4.363232 1.561411

H -4.192013 -4.021282 1.861977

MalibatolA_Conf48.log

Energy (E) = -1605.16480706 Hartree

Enthalpy (H) = -1604.709135 Hartree

Gibbs free energy (G) = -1604.797062 Hartree

Charge = 0, Spin = 1

C 3.074641 2.432162 1.970776

C 1.81437 2.982681 1.765387

C 0.821473 2.080639 1.414194

C 1.016217 0.708972 1.284058

C 2.300465 0.181923 1.458535

C 3.319122 1.054821 1.809372

H 1.63432 4.04655 1.859893

H 4.328927 0.681645 1.941459

C -0.262649 0.146774 0.91013

C -1.107378 1.213145 0.787276

O -0.469194 2.397775 1.112812

C -2.486203 1.367695 0.320831

C -3.34683 2.294097 0.922128

C -2.956696 0.61505 -0.761779

C -4.648082 2.454114 0.468339

H -2.996342 2.890972 1.757818

C -4.258148 0.762172 -1.217685

H -2.29333 -0.086937 -1.256846

C -5.108293 1.68265 -0.600116

H -5.31719 3.168172 0.936262

H -4.617045 0.176687 -2.058985

C -0.587206 -1.287714 0.776504

C -1.846736 -1.722024 1.193084

C 0.357249 -2.199574 0.256787

C -2.209231 -3.056876 1.050572

H -2.553787 -1.027364 1.63036

C -0.058843 -3.531368 0.094014

C -1.323525 -3.964621 0.486958

H -1.599606 -5.004625 0.345077

C 2.530492 -1.283957 1.197367

H 2.119636 -1.859178 2.03805

C 1.780373 -1.787789 -0.08415

H 2.342307 -2.696589 -0.325845

C 1.888998 -0.863599 -1.295876

C 0.800516 -0.630894 -2.140455

C 3.098718 -0.235323 -1.611065

C 0.899762 0.207928 -3.245029

H -0.15238 -1.108824 -1.936955

C 3.21459 0.609627 -2.709012

H 3.967301 -0.402172 -0.983884

C 2.109379 0.837098 -3.529014

H 0.042696 0.382317 -3.887341

H 4.161411 1.093916 -2.932009

O 3.925868 -1.514904 1.115837

O 2.159173 1.660582 -4.621126

O 0.735376 -4.507422 -0.447286

O -3.454385 -3.422406 1.473112

O -6.395408 1.870545 -1.006665

H 3.047686 2.027311 -4.702083

H 1.464533 -4.116982 -0.941589

H -3.575507 -4.368162 1.325

H -6.589668 1.274302 -1.740382

H 4.063673 -2.468511 1.131507

O 4.080996 3.290309 2.318249

H 4.903686 2.794786 2.408244

MalibatolA_Conf49.log

Energy (E) = -1605.16542900 Hartree

Enthalpy (H) = -1604.709318 Hartree

Gibbs free energy (G) = -1604.797049 Hartree

Charge = 0, Spin = 1

C -2.418424 -3.366189 1.375499

C -1.132199 -3.661959 0.938476

C -0.285644 -2.572747 0.802112

C -0.640756 -1.256552 1.086142

C -1.946726 -0.977784 1.500339

C -2.822355 -2.04469 1.644281

H -0.824969 -4.675632 0.712213

H -3.84312 -1.866788 1.967292

C 0.527198 -0.449152 0.814278

C 1.469089 -1.319449 0.340049

O 0.99794 -2.620838 0.350491

C 2.809163 -1.169701 -0.234147

C 3.089759 -0.138019 -1.139989

C 3.822038 -2.079879 0.083408

C 4.351841 -0.00436 -1.695931

H 2.30844 0.564088 -1.412578

C 5.088824 -1.957992 -0.472819

H 3.621628 -2.889129 0.777993

C 5.357237 -0.915304 -1.360692

H 4.568891 0.793124 -2.398363

H 5.871832 -2.665618 -0.217076

C 0.668463 1.007291 1.010128

C 1.93466 1.496379 1.352168

C -0.419756 1.890351 0.846444

C 2.161534 2.862338 1.455587

H 2.752121 0.807616 1.533739

C -0.133418 3.269677 0.903106

C 1.131407 3.759642 1.204343

H 1.302821 4.829233 1.244075

C -2.362645 0.4497 1.76435

H -1.925215 0.772107 2.715344

C -1.876283 1.474989 0.68791

H -2.468127 2.34714 0.980739

C -2.317617 1.118823 -0.724232

C -1.480552 0.491889 -1.653591

C -3.626724 1.410002 -1.122541

C -1.936594 0.143383 -2.918087

H -0.449336 0.273079 -1.398242

C -4.101276 1.063759 -2.384029

H -4.292042 1.930056 -0.438278

C -3.254211 0.420217 -3.283683

H -1.277765 -0.342173 -3.630531

H -5.121502 1.300121 -2.672587

O -3.762315 0.565121 1.945097

O -3.656783 0.052122 -4.537008

O -1.088308 4.230767 0.688096

O 3.381795 3.377036 1.784967

O 6.580912 -0.740805 -1.934365

H -4.583346 0.293602 -4.656023

H -1.808748 3.877355 0.154244

H 4.011907 2.655387 1.902251

H 7.182526 -1.430884 -1.628143

H -4.192053 0.258196 1.135692

O -3.282797 -4.414877 1.524621

H -4.1337 -4.087054 1.839763

MalibatolA_Conf50.log

Energy (E) = -1605.16488186 Hartree

Enthalpy (H) = -1604.709145 Hartree

Gibbs free energy (G) = -1604.796997 Hartree

Charge = 0, Spin = 1

C 3.078772 2.43289 1.965711

C 1.82016 2.98496 1.755977

C 0.825439 2.08218 1.405493

C 1.018608 0.710998 1.281092

C 2.303516 0.182482 1.459363

C 3.32157 1.054398 1.808045

H 1.634582 4.049507 1.845968

H 4.332801 0.690809 1.944071

C -0.26059 0.149075 0.908589

C -1.104104 1.216013 0.78087

O -0.463956 2.400811 1.10191

C -2.482443 1.370387 0.312976

C -3.343012 2.299064 0.910835

C -2.952751 0.614504 -0.767464

C -4.643956 2.458278 0.45579

H -2.992736 2.898359 1.744891

C -4.253939 0.760665 -1.224451

H -2.289408 -0.089289 -1.259986

C -5.103992 1.683507 -0.610337

H -5.312997 3.174135 0.92106

H -4.612721 0.172582 -2.063996

C -0.587227 -1.28551 0.78101

C -1.847497 -1.716236 1.199032

C 0.35614 -2.200896 0.265671

C -2.211724 -3.051228 1.062306

H -2.553761 -1.018721 1.633023

C -0.061694 -3.532818 0.108471

C -1.327053 -3.96267 0.50302

H -1.604534 -5.002912 0.365671

C 2.530415 -1.284908 1.203749

H 2.117461 -1.855787 2.046424

C 1.779981 -1.792437 -0.076176

H 2.34051 -2.703049 -0.314245

C 1.890304 -0.873431 -1.291662

C 0.803029 -0.644967 -2.138996

C 3.100321 -0.246268 -1.607861

C 0.903672 0.188869 -3.247209

H -0.150011 -1.122325 -1.934789

C 3.217556 0.593761 -2.709463

H 3.968053 -0.41001 -0.978686

C 2.11354 0.817132 -3.532161

H 0.047566 0.359971 -3.891687

H 4.164573 1.077305 -2.93321

O 3.925195 -1.51959 1.124083

O 2.164815 1.635686 -4.627931

O 0.731367 -4.512244 -0.428413

O -3.457568 -3.41307 1.486019

O -6.39083 1.870546 -1.018231

H 3.053259 2.002617 -4.708668

H 1.461204 -4.124886 -0.924164

H -3.580261 -4.359206 1.341715

H -6.58487 1.271804 -1.749981

H 4.060455 -2.473537 1.14147

O 4.155987 3.199597 2.314394

H 3.882516 4.122002 2.387155

MalibatolA_Conf51.log

Energy (E) = -1605.16522412 Hartree

Enthalpy (H) = -1604.709164 Hartree

Gibbs free energy (G) = -1604.796996 Hartree

Charge = 0, Spin = 1

C -2.430587 -3.344529 1.407773

C -1.144972 -3.649057 0.975041

C -0.294278 -2.564211 0.829369

C -0.645017 -1.24419 1.100545

C -1.950679 -0.956751 1.509904

C -2.830157 -2.019098 1.663415

H -0.84123 -4.665901 0.75849

H -3.850677 -1.834718 1.983575

C 0.526063 -0.443432 0.822642

C 1.465259 -1.321239 0.357079

O 0.989395 -2.620933 0.37922

C 2.805137 -1.181582 -0.219849

C 3.090515 -0.153775 -1.128613

C 3.8123 -2.098623 0.096163

C 4.351753 -0.030509 -1.688765

H 2.313549 0.553466 -1.400306

C 5.078201 -1.987129 -0.464269

H 3.608148 -2.90522 0.792724

C 5.35158 -0.947919 -1.354675

H 4.572516 0.764161 -2.393227

H 5.856855 -2.699944 -0.209634

C 0.671533 1.014401 1.006173

C 1.934599 1.503015 1.353472

C -0.415009 1.899157 0.826257

C 2.162878 2.869684 1.448746

H 2.753624 0.821845 1.548526

C -0.126738 3.276632 0.871595

C 1.137973 3.767262 1.179859

H 1.304586 4.839169 1.208644

C -2.361863 0.475223 1.756407

H -1.922424 0.808454 2.702718

C -1.871611 1.484421 0.666268

H -2.463118 2.361524 0.944533

C -2.30808 1.110014 -0.742802

C -1.466773 0.475357 -1.663016

C -3.616392 1.394537 -1.148635

C -1.917797 0.113224 -2.925601

H -0.435931 0.261313 -1.402118

C -4.085919 1.034658 -2.408068

H -4.285042 1.920205 -0.471934

C -3.234576 0.384015 -3.298505

H -1.255488 -0.37806 -3.630853

H -5.105542 1.266279 -2.702623

O -3.760852 0.598011 1.93741

O -3.632618 0.003309 -4.549514

O -1.076167 4.238749 0.638802

O 3.41862 3.279116 1.791526

O 6.574857 -0.783022 -1.932213

H -4.558975 0.243102 -4.673546

H -1.793074 3.881819 0.102426

H 3.436093 4.242394 1.842061

H 7.173579 -1.4746 -1.623757

H -4.193478 0.276474 1.135409

O -3.299237 -4.388414 1.565719

H -4.15001 -4.053615 1.873828

MalibatolA_Conf52.log

Energy (E) = -1605.16443422 Hartree

Enthalpy (H) = -1604.708712 Hartree

Gibbs free energy (G) = -1604.796923 Hartree

Charge = 0, Spin = 1

C 3.024164 2.522898 1.897413

C 1.756941 3.052631 1.680611

C 0.771586 2.129235 1.364734

C 0.979716 0.756073 1.283531

C 2.267742 0.247027 1.48112

C 3.280569 1.142191 1.787672

H 1.567536 4.117642 1.735405

H 4.298334 0.788031 1.91892

C -0.2894 0.168959 0.912911

C -1.144206 1.222759 0.752974

O -0.5204 2.423199 1.047767

C -2.521492 1.354276 0.273674

C -2.978463 0.580642 -0.801853

C -3.392772 2.282005 0.853301

C -4.275217 0.712674 -1.271286

H -2.305793 -0.125106 -1.278736

C -4.692455 2.426794 0.385362

H -3.054613 2.894196 1.682968

C -5.137003 1.637744 -0.676161

H -4.628662 0.116474 -2.105656

H -5.364768 3.14626 0.843211

C -0.596968 -1.271383 0.797609

C -1.862516 -1.709594 1.191583

C 0.36397 -2.185271 0.311533

C -2.215369 -3.047597 1.054085

H -2.58295 -1.015302 1.606873

C -0.043309 -3.51953 0.148148

C -1.314373 -3.955855 0.516349

H -1.582918 -4.998076 0.376392

C 2.497701 -1.234982 1.289477

H 2.046368 -1.767269 2.136307

C 1.797587 -1.778437 0.006164

H 2.373654 -2.689785 -0.182239

C 1.944377 -0.892117 -1.229598

C 3.162936 -0.264607 -1.517791

C 0.8909 -0.70107 -2.124482

C 3.319404 0.535678 -2.641256

H 4.005399 -0.397268 -0.847748

C 1.031812 0.094612 -3.258655

H -0.067331 -1.176744 -1.941115

C 2.24882 0.71979 -3.516765

H 4.267354 1.021218 -2.849335

H 0.196501 0.232165 -3.939761

O 3.873366 -1.574377 1.215664

O 2.450036 1.519273 -4.609069

O 0.767813 -4.494756 -0.369076

O -3.467344 -3.415272 1.45387

O -6.400817 1.733732 -1.177059

H 1.6383 1.553019 -5.129199

H 1.503614 -4.102702 -0.852278

H -3.580799 -4.362749 1.310997

H -6.886892 2.411677 -0.691621

H 4.244447 -1.499403 2.102145

O 4.023587 3.405085 2.201366

H 4.855202 2.925374 2.294305

MalibatolA_Conf53.log

Energy (E) = -1605.16553701 Hartree

Enthalpy (H) = -1604.709192 Hartree

Gibbs free energy (G) = -1604.796896 Hartree

Charge = 0, Spin = 1

C -2.436333 -3.33171 1.401405

C -1.151519 -3.639046 0.970027

C -0.29663 -2.554756 0.829549

C -0.644518 -1.236171 1.104319

C -1.950097 -0.947198 1.518513

C -2.831614 -2.006469 1.666782

H -0.843843 -4.654218 0.745553

H -3.851921 -1.832344 1.988187

C 0.526398 -0.436496 0.823469

C 1.463992 -1.315236 0.355897

O 0.986033 -2.614025 0.378063

C 2.803888 -1.177092 -0.221752

C 3.086984 -0.156712 -1.139505

C 3.814067 -2.087011 0.105003

C 4.349086 -0.033242 -1.697774

H 2.307699 0.544707 -1.419555

C 5.080777 -1.975571 -0.453685

H 3.611668 -2.887727 0.80886

C 5.351938 -0.943346 -1.352871

H 4.568129 0.755723 -2.409152

H 5.861815 -2.682747 -0.190748

C 0.672715 1.02203 1.000139

C 1.940566 1.511763 1.334709

C -0.412636 1.906211 0.823152

C 2.171973 2.878415 1.417854

H 2.755729 0.823098 1.526666

C -0.12163 3.28518 0.859065

C 1.144946 3.775302 1.152918

H 1.319982 4.844781 1.176704

C -2.353266 0.485778 1.771287

H -1.900655 0.816888 2.712127

C -1.87078 1.492284 0.674695

H -2.460191 2.370314 0.954591

C -2.317025 1.112783 -0.730078

C -1.481264 0.476394 -1.654122

C -3.629235 1.391959 -1.12688

C -1.941387 0.106863 -2.911306

H -0.447792 0.266397 -1.400408

C -4.107789 1.024805 -2.380741

H -4.294016 1.91863 -0.447157

C -3.26199 0.371777 -3.274736

H -1.28342 -0.386152 -3.619422

H -5.130413 1.252087 -2.66812

O -3.749142 0.614663 1.969464

O -3.669491 -0.017165 -4.520153

O -1.073015 4.245972 0.628835

O 3.393871 3.393974 1.739867

O 6.575959 -0.778469 -1.928741

H -4.598294 0.216925 -4.636537

H -1.797603 3.884489 0.106155

H 4.021746 2.672243 1.86811

H 7.17651 -1.465265 -1.613232

H -4.193912 0.28841 1.17618

O -3.389505 -4.294622 1.580754

H -3.016618 -5.157797 1.364461

MalibatolA_Conf54.log

Energy (E) = -1605.16551108 Hartree

Enthalpy (H) = -1604.709053 Hartree

Gibbs free energy (G) = -1604.796682 Hartree

Charge = 0, Spin = 1

C -2.469455 -3.311318 1.406736

C -1.183614 -3.629508 0.986378

C -0.319727 -2.552022 0.848765

C -0.659769 -1.230273 1.117968

C -1.966816 -0.930047 1.519526

C -2.8573 -1.982288 1.664195

H -0.881961 -4.647534 0.766738

H -3.879199 -1.799523 1.975615

C 0.51858 -0.439942 0.842404

C 1.451986 -1.326223 0.380658

O 0.964881 -2.621621 0.404225

C 2.790911 -1.196975 -0.200901

C 3.794657 -2.122108 0.109828

C 3.077982 -0.172106 -1.110031

C 5.056606 -2.019702 -0.457644

H 3.587616 -2.927073 0.807468

C 4.338242 -0.057 -1.677585

H 2.305098 0.54085 -1.3783

C 5.332298 -0.982346 -1.350046

H 5.836303 -2.733354 -0.214179

H 4.553526 0.739697 -2.383541

C 0.673976 1.017918 1.016278

C 1.941353 1.499862 1.36357

C -0.402898 1.908905 0.823701

C 2.18118 2.865283 1.443877

H 2.749556 0.80634 1.567271

C -0.102897 3.285927 0.856688

C 1.163605 3.768313 1.163276

H 1.346178 4.836597 1.184201

C -2.361888 0.507369 1.760002

H -1.915161 0.841274 2.702647

C -1.861776 1.5033 0.661448

H -2.448074 2.387194 0.928755

C -2.293906 1.118335 -0.746381

C -1.449774 0.476641 -1.659122

C -3.600326 1.401091 -1.159408

C -1.896278 0.106009 -2.920928

H -0.420031 0.263659 -1.393118

C -4.065352 1.032854 -2.418003

H -4.271057 1.932443 -0.489277

C -3.211292 0.375405 -3.300793

H -1.231581 -0.390625 -3.620169

H -5.083576 1.263514 -2.718124

O -3.758418 0.647818 1.945338

O -3.605109 -0.013088 -4.550771

O -1.045259 4.251902 0.610327

O 3.402953 3.373606 1.77758

O 6.587393 -0.921514 -1.877234

H -4.530598 0.22748 -4.679801

H -1.761111 3.89508 0.072423

H 4.024427 2.647899 1.91403

H 6.649832 -0.165862 -2.474517

H -4.199066 0.319467 1.150679

O -3.431141 -4.266673 1.581214

H -3.063365 -5.133089 1.369037

MalibatolA_Conf55.log

Energy (E) = -1605.16524883 Hartree

Enthalpy (H) = -1604.708960 Hartree

Gibbs free energy (G) = -1604.796646 Hartree

Charge = 0, Spin = 1

C -2.469942 -3.318338 1.413236

C -1.184101 -3.636085 0.990635

C -0.322491 -2.559554 0.846854

C -0.66319 -1.235612 1.111366

C -1.969128 -0.934767 1.510085

C -2.859309 -1.988378 1.661234

H -0.888341 -4.656394 0.779384

H -3.880307 -1.793118 1.97328

C 0.516233 -0.446037 0.83744

C 1.450148 -1.333388 0.379482

O 0.963236 -2.628997 0.404016

C 2.791256 -1.204931 -0.196966

C 3.79248 -2.132198 0.115535

C 3.083313 -0.17833 -1.102511

C 5.05687 -2.030049 -0.446485

H 3.581595 -2.938487 0.810472

C 4.345917 -0.063705 -1.664848

H 2.312561 0.536482 -1.371943

C 5.337543 -0.990921 -1.335242

H 5.834729 -2.745108 -0.201282

H 4.565218 0.734566 -2.367784

C 0.672641 1.011402 1.015301

C 1.936505 1.491411 1.371491

C -0.404727 1.903954 0.820395

C 2.174998 2.856743 1.460884

H 2.748133 0.804492 1.577234

C -0.10574 3.279291 0.859774

C 1.160113 3.761159 1.176996

H 1.335688 4.831784 1.200775

C -2.368563 0.502285 1.745893

H -1.930137 0.837566 2.691935

C -1.863006 1.499327 0.651358

H -2.449598 2.383168 0.918227

C -2.290763 1.11839 -0.758734

C -3.595714 1.40882 -1.176379

C -1.447054 0.475541 -1.668694

C -4.057427 1.046769 -2.436164

H -4.265531 1.941631 -0.506452

C -1.890979 0.110659 -2.934852

H -0.419358 0.255955 -1.400011

C -3.202939 0.388257 -3.318555

H -5.070893 1.279143 -2.746094

H -1.22012 -0.387577 -3.629192

O -3.767011 0.639429 1.920436

O -3.695529 0.049192 -4.547861

O -1.044968 4.247582 0.611397

O 3.430843 3.258362 1.812496

O 6.59493 -0.930346 -1.857073

H -3.006243 -0.396927 -5.054664

H -1.758701 3.893756 0.068616

H 3.455399 4.221809 1.856694

H 6.661189 -0.172472 -2.451111

H -4.200256 0.31666 1.119375

O -3.349333 -4.353648 1.568414

H -4.200031 -4.009798 1.866758

MalibatolA_Conf56.log

Energy (E) = -1605.16549393 Hartree

Enthalpy (H) = -1604.709220 Hartree

Gibbs free energy (G) = -1604.796604 Hartree

Charge = 0, Spin = 1

C -2.424788 -3.362007 1.377399

C -1.138417 -3.65927 0.941827

C -0.290958 -2.570834 0.804701

C -0.645475 -1.253988 1.086362

C -1.951479 -0.973796 1.499515

C -2.827947 -2.039875 1.644288

H -0.831662 -4.673498 0.71741

H -3.848718 -1.860936 1.96671

C 0.523319 -0.447753 0.814558

C 1.465255 -1.319453 0.343065

O 0.993168 -2.62051 0.354716

C 2.806803 -1.17149 -0.228211

C 3.089626 -0.142997 -1.136941

C 3.819256 -2.079754 0.096104

C 4.353458 -0.010507 -1.689211

H 2.308765 0.557633 -1.414603

C 5.087797 -1.959037 -0.456365

H 3.617128 -2.88644 0.793175

C 5.35839 -0.919433 -1.347183

H 4.572331 0.784591 -2.393781

H 5.87052 -2.665024 -0.195284

C 0.665389 1.008926 1.008252

C 1.931675 1.497828 1.350268

C -0.422185 1.89237 0.84245

C 2.159294 2.863822 1.451572

H 2.748616 0.808905 1.533538

C -0.135053 3.271627 0.896938

C 1.12989 3.761328 1.198124

H 1.301918 4.830882 1.236161

C -2.366111 0.454332 1.761894

H -1.927868 0.77748 2.712264

C -1.878887 1.477647 0.683955

H -2.470284 2.350641 0.97523

C -2.319858 1.119949 -0.727902

C -3.630574 1.410826 -1.126665

C -1.483539 0.493775 -1.656032

C -4.104681 1.064675 -2.38647

H -4.295251 1.930573 -0.441534

C -1.939992 0.145145 -2.922139

H -0.452153 0.274574 -1.40161

C -3.257378 0.422107 -3.287379

H -5.122553 1.2968 -2.681783

H -1.274808 -0.340402 -3.63073

O -3.765544 0.571356 1.943399

O -3.762239 0.09732 -4.515597

O -1.089138 4.232953 0.679607

O 3.379653 3.378332 1.780914

O 6.583876 -0.746172 -1.917324

H -3.077646 -0.340572 -5.035959

H -1.809835 3.878941 0.146501

H 4.00926 2.656504 1.899783

H 7.184974 -1.434412 -1.606002

H -4.196508 0.262265 1.135518

O -3.290044 -4.409849 1.527214

H -4.141067 -4.080938 1.840919

MalibatolA_Conf57.log

Energy (E) = -1605.16466583 Hartree

Enthalpy (H) = -1604.708898 Hartree

Gibbs free energy (G) = -1604.796604 Hartree

Charge = 0, Spin = 1

C 3.031469 2.521941 1.90061

C 1.765934 3.052628 1.678128

C 0.780895 2.128201 1.356962

C 0.989464 0.756135 1.277217

C 2.279127 0.247005 1.475399

C 3.289022 1.141662 1.785884

H 1.568827 4.117416 1.734252

H 4.308813 0.798992 1.920449

C -0.279742 0.168582 0.908661

C -1.134618 1.222064 0.745775

O -0.510165 2.422671 1.037873

C -2.511594 1.351343 0.265405

C -2.968888 0.569356 -0.804021

C -3.382842 2.283505 0.838045

C -4.265927 0.697246 -1.27378

H -2.296111 -0.139591 -1.276018

C -4.682627 2.424573 0.369251

H -3.04456 2.902168 1.662847

C -5.12756 1.627026 -0.685767

H -4.619451 0.094578 -2.103458

H -5.354834 3.147737 0.821385

C -0.58827 -1.272074 0.803714

C -1.854831 -1.706946 1.203726

C 0.369964 -2.189542 0.324679

C -2.208204 -3.045651 1.077299

H -2.569794 -1.003359 1.616361

C -0.038092 -3.526857 0.174222

C -1.307294 -3.959209 0.547293

H -1.583103 -4.999639 0.418757

C 2.511541 -1.234552 1.281195

H 2.071148 -1.767963 2.133179

C 1.800983 -1.783266 0.006436

H 2.375893 -2.695092 -0.182998

C 1.933426 -0.90325 -1.235734

C 0.874462 -0.740662 -2.132464

C 3.137607 -0.255168 -1.530455

C 0.997645 0.047607 -3.271515

H -0.07341 -1.234441 -1.9433

C 3.276729 0.540082 -2.662092

H 3.984164 -0.365135 -0.861417

C 2.201323 0.696199 -3.536661

H 0.163845 0.167519 -3.955592

H 4.218566 1.040836 -2.86884

O 3.887644 -1.568887 1.19272

O 2.276039 1.467845 -4.66442

O 0.773883 -4.505184 -0.336223

O -3.434832 -3.512747 1.452115

O -6.39152 1.718971 -1.186916

H 3.155529 1.859872 -4.722017

H 1.504736 -4.116237 -0.82933

H -3.972981 -2.775364 1.765103

H -6.876871 2.401984 -0.707884

H 4.269247 -1.488221 2.074239

O 4.101171 3.311483 2.219563

H 3.817977 4.232872 2.262107

MalibatolA_Conf58.log

Energy (E) = -1605.16450568 Hartree

Enthalpy (H) = -1604.708823 Hartree

Gibbs free energy (G) = -1604.796592 Hartree

Charge = 0, Spin = 1

C 3.069427 2.4835 1.903798

C 1.80883 3.029521 1.690355

C 0.812193 2.118165 1.367781

C 1.004947 0.744279 1.278961

C 2.28999 0.219762 1.467032

C 3.311117 1.101301 1.778589

H 1.623677 4.096008 1.754188

H 4.327865 0.746815 1.90548

C -0.272359 0.173166 0.912757

C -1.115728 1.237135 0.759135

O -0.476823 2.429115 1.055692

C -2.492876 1.382627 0.284019

C -3.358584 2.313471 0.870845

C -2.956773 0.615621 -0.791229

C -4.658647 2.464164 0.410247

H -3.013126 2.921084 1.700908

C -4.256933 0.753317 -1.253747

H -2.2893 -0.090414 -1.274895

C -5.112257 1.67843 -0.650547

H -5.331764 3.181573 0.867167

H -4.611055 0.156655 -2.08923

C -0.59819 -1.263609 0.803303

C -1.865195 -1.686087 1.209354

C 0.34867 -2.189847 0.313682

C -2.233394 -3.020885 1.081183

H -2.574907 -0.982093 1.626751

C -0.073084 -3.520941 0.161953

C -1.34572 -3.941837 0.542796

H -1.626323 -4.981957 0.41108

C 2.505996 -1.262822 1.260746

H 2.066097 -1.797779 2.112027

C 1.781101 -1.795571 -0.012744

H 2.345872 -2.711433 -0.21327

C 1.912471 -0.908187 -1.249767

C 0.849467 -0.7336 -2.139399

C 3.118651 -0.264994 -1.546908

C 0.970436 0.062275 -3.273345

H -0.099959 -1.223649 -1.948328

C 3.255699 0.537783 -2.673542

H 3.968463 -0.384664 -0.883696

C 2.176089 0.70633 -3.540624

H 0.133448 0.191704 -3.951764

H 4.199082 1.034849 -2.882182

O 3.878001 -1.610453 1.160403

O 2.248074 1.485947 -4.663088

O 0.723462 -4.507616 -0.356032

O -3.486821 -3.372286 1.491153

O -6.398348 1.856809 -1.064654

H 3.128983 1.874363 -4.723199

H 1.461553 -4.126299 -0.844351

H -3.613631 -4.318498 1.351254

H -6.58699 1.250764 -1.79182

H 4.266974 -1.537683 2.039412

O 4.149415 3.25882 2.223131

H 3.877305 4.183158 2.27352

MalibatolA_Conf59.log

Energy (E) = -1605.16466171 Hartree

Enthalpy (H) = -1604.708926 Hartree

Gibbs free energy (G) = -1604.796541 Hartree

Charge = 0, Spin = 1

C 3.058161 2.502142 1.893729

C 1.797088 3.04409 1.672681

C 0.803184 2.128355 1.354038

C 0.999213 0.754334 1.275607

C 2.284556 0.233672 1.471789

C 3.303001 1.119496 1.779749

H 1.609655 4.110642 1.72819

H 4.319943 0.767838 1.912774

C -0.275704 0.177805 0.909916

C -1.121249 1.238738 0.746926

O -0.485838 2.434133 1.036582

C -2.497953 1.376936 0.268394

C -3.367315 2.309532 0.847022

C -2.958358 0.599642 -0.800999

C -4.667539 2.452056 0.384247

H -3.024595 2.924988 1.67243

C -4.258646 0.729064 -1.265468

H -2.288217 -0.108382 -1.278058

C -5.117569 1.65617 -0.6705

H -5.343424 3.170918 0.834746

H -4.610121 0.124505 -2.096398

C -0.597561 -1.260161 0.807923

C -1.866838 -1.683185 1.211951

C 0.351133 -2.186936 0.328014

C -2.232423 -3.018834 1.088773

H -2.574511 -0.972556 1.625076

C -0.069076 -3.520873 0.180965

C -1.34114 -3.941345 0.557951

H -1.626435 -4.979544 0.432081

C 2.503283 -1.250163 1.27828

H 2.060074 -1.778826 2.131766

C 1.784848 -1.79372 0.005721

H 2.350976 -2.710913 -0.184413

C 1.921985 -0.916345 -1.237815

C 0.863111 -0.748008 -2.133567

C 3.130209 -0.277007 -1.535018

C 0.99032 0.037507 -3.274072

H -0.087832 -1.235111 -1.942491

C 3.273396 0.515479 -2.66811

H 3.976881 -0.391794 -0.866952

C 2.198065 0.677473 -3.541707

H 0.156562 0.161967 -3.957385

H 4.218366 1.009536 -2.876677

O 3.876065 -1.597075 1.186908

O 2.27658 1.446567 -4.670936

O 0.733027 -4.507088 -0.329876

O -3.462382 -3.473659 1.467764

O -6.403856 1.826374 -1.087149

H 3.158347 1.833192 -4.730118

H 1.467089 -4.1252 -0.823768

H -3.991637 -2.730544 1.782453

H -6.588711 1.215029 -1.810874

H 4.260922 -1.517171 2.067091

O 4.135641 3.281969 2.210395

H 3.861673 4.206169 2.252511

MalibatolA_Conf60.log

Energy (E) = -1605.16440596 Hartree

Enthalpy (H) = -1604.708697 Hartree

Gibbs free energy (G) = -1604.796522 Hartree

Charge = 0, Spin = 1

C -3.048215 -2.504825 1.890272

C -1.785327 -3.044984 1.673857

C -0.791831 -2.12953 1.360439

C -0.988308 -0.754506 1.281378

C -2.272273 -0.234886 1.477936

C -3.293021 -1.121898 1.781891

H -1.604979 -4.111616 1.727551

H -4.307933 -0.759422 1.912651

C 0.28617 -0.17738 0.913779

C 1.132298 -1.238077 0.753052

O 0.49818 -2.433801 1.044696

C 2.509484 -1.378249 0.276002

C 3.376935 -2.31 0.858837

C 2.971799 -0.606143 -0.79622

C 4.677163 -2.456667 0.397445

H 3.032718 -2.921504 1.686545

C 4.272006 -0.740027 -1.259709

H 2.303465 0.10169 -1.276059

C 5.129069 -1.666076 -0.660423

H 5.351591 -3.174651 0.851514

H 4.625038 -0.139343 -2.092775

C 0.605902 1.260633 0.801536

C 1.874149 1.687989 1.198654

C -0.346405 2.183081 0.314744

C 2.238542 3.023036 1.062855

H 2.588109 0.987359 1.614411

C 0.07204 3.514243 0.15399

C 1.346058 3.939577 0.524884

H 1.623742 4.97962 0.386535

C -2.489318 1.249316 1.288005

H -2.034634 1.776516 2.136226

C -1.78277 1.788469 0.006405

H -2.350734 2.704912 -0.181881

C -1.934971 0.904773 -1.230579

C -3.15852 0.288448 -1.521691

C -0.881506 0.705115 -2.123599

C -3.319925 -0.509475 -2.646124

H -4.001072 0.428002 -0.853171

C -1.027269 -0.088371 -3.25872

H 0.080538 1.172127 -1.937934

C -2.249293 -0.702518 -3.519635

H -4.271737 -0.98642 -2.856412

H -0.191866 -0.232862 -3.938257

O -3.86192 1.60068 1.212749

O -2.455512 -1.499534 -4.612794

O -0.730141 4.496727 -0.363424

O 3.493334 3.379215 1.464358

O 6.415302 -1.84047 -1.075706

H -1.643019 -1.540471 -5.131191

H -1.468811 4.111347 -0.847645

H 3.616636 4.325213 1.319967

H 6.602445 -1.231069 -1.800454

H -4.235403 1.525496 2.098216

O -4.055195 -3.378958 2.19252

H -4.882459 -2.892188 2.287412

MalibatolA_Conf61.log

Energy (E) = -1605.16454551 Hartree

Enthalpy (H) = -1604.708807 Hartree

Gibbs free energy (G) = -1604.796516 Hartree

Charge = 0, Spin = 1

C 3.028701 2.52824 1.894827

C 1.763682 3.059305 1.670201

C 0.777868 2.134718 1.351721

C 0.985324 0.762301 1.276098

C 2.274215 0.252628 1.47774

C 3.284905 1.147252 1.785506

H 1.567405 4.124419 1.722951

H 4.304103 0.803753 1.922498

C -0.283898 0.174442 0.908132

C -1.138174 1.227983 0.742743

O -0.512937 2.429108 1.031727

C -2.515706 1.357207 0.263839

C -2.974709 0.574742 -0.80445

C -3.386032 2.289592 0.837409

C -4.272474 0.70251 -1.272277

H -2.302899 -0.134934 -1.276713

C -4.686632 2.43034 0.370761

H -3.04648 2.908497 1.661502

C -5.133292 1.632252 -0.683063

H -4.627517 0.099123 -2.100783

H -5.358163 3.153431 0.824014

C -0.59284 -1.26646 0.80414

C -1.857596 -1.701112 1.204565

C 0.366853 -2.184526 0.323918

C -2.21068 -3.040298 1.079546

H -2.576954 -1.003352 1.615898

C -0.040808 -3.520008 0.172397

C -1.310922 -3.953168 0.547478

H -1.579842 -4.996534 0.417091

C 2.503704 -1.230071 1.290343

H 2.05551 -1.759107 2.140992

C 1.799654 -1.779939 0.012203

H 2.374585 -2.692709 -0.172821

C 1.94206 -0.901643 -1.229809

C 3.155846 -0.266793 -1.52184

C 0.888455 -0.727464 -2.128075

C 3.307443 0.524377 -2.652507

H 3.998422 -0.386604 -0.849528

C 1.024649 0.058615 -3.269421

H -0.066096 -1.209366 -1.941731

C 2.236879 0.691439 -3.531413

H 4.251713 1.015761 -2.863576

H 0.189379 0.182806 -3.953181

O 3.879051 -1.569709 1.212465

O 2.433111 1.482206 -4.630976

O 0.769172 -4.499361 -0.338917

O -3.461668 -3.404524 1.485574

O -6.39833 1.723542 -1.181806

H 1.621767 1.504474 -5.152361

H 1.502235 -4.111104 -0.829356

H -3.57536 -4.353264 1.351574

H -6.883446 2.405424 -0.700939

H 4.256051 -1.482247 2.095311

O 4.099044 3.317853 2.21141

H 3.817163 4.23985 2.249092

MalibatolA_Conf62.log

Energy (E) = -1605.16558217 Hartree

Enthalpy (H) = -1604.709072 Hartree

Gibbs free energy (G) = -1604.796437 Hartree

Charge = 0, Spin = 1

C -2.437344 -3.338888 1.393192

C -1.151571 -3.643849 0.962929

C -0.29806 -2.558135 0.824988

C -0.648101 -1.240361 1.101074

C -1.954656 -0.953796 1.513874

C -2.834825 -2.014538 1.659617

H -0.842078 -4.658323 0.737782

H -3.855743 -1.842262 1.98006

C 0.522354 -0.438816 0.823498

C 1.461803 -1.315693 0.356176

O 0.985406 -2.615106 0.375421

C 2.802764 -1.17502 -0.218425

C 3.085932 -0.154354 -1.135818

C 3.814071 -2.082638 0.111198

C 4.349095 -0.028511 -1.691183

H 2.305896 0.545442 -1.417855

C 5.081879 -1.968773 -0.444494

H 3.611666 -2.883413 0.81499

C 5.352978 -0.936454 -1.343588

H 4.568221 0.760656 -2.402313

H 5.863776 -2.67415 -0.179296

C 0.667128 1.019448 1.003515

C 1.934394 1.509654 1.339745

C -0.418928 1.903005 0.828103

C 2.164521 2.876321 1.425898

H 2.750129 0.82134 1.530503

C -0.129226 3.28219 0.867135

C 1.136771 3.772843 1.162539

H 1.310812 4.842431 1.18864

C -2.360389 0.478006 1.769022

H -1.91083 0.807187 2.712011

C -1.876546 1.488374 0.676799

H -2.466748 2.365141 0.959066

C -2.320394 1.11453 -0.730201

C -3.633023 1.397137 -1.128711

C -1.484105 0.482754 -1.654528

C -4.108577 1.038501 -2.384374

H -4.298124 1.920081 -0.446435

C -1.942082 0.121253 -2.916559

H -0.451416 0.269307 -1.400443

C -3.260912 0.391223 -3.28159

H -5.127861 1.264595 -2.679503

H -1.276829 -0.368477 -3.622214

O -3.757026 0.604406 1.963694

O -3.76695 0.054846 -4.506215

O -1.081351 4.242636 0.638553

O 3.38587 3.392293 1.749351

O 6.577954 -0.769508 -1.916796

H -3.080797 -0.382551 -5.024866

H -1.805347 3.881857 0.114561

H 4.014316 2.670825 1.87626

H 7.178967 -1.455186 -1.599737

H -4.199245 0.278683 1.168764

O -3.389357 -4.303289 1.570527

H -3.014859 -5.165886 1.35476

MalibatolA_Conf63.log

Energy (E) = -1605.16533375 Hartree

Enthalpy (H) = -1604.708919 Hartree

Gibbs free energy (G) = -1604.796410 Hartree

Charge = 0, Spin = 1

C -2.452058 -3.305655 1.43463

C -1.167793 -3.62318 1.009206

C -0.307648 -2.54436 0.859176

C -0.650034 -1.221576 1.120062

C -1.955268 -0.922364 1.52831

C -2.841968 -1.975996 1.685701

H -0.864461 -4.641963 0.795421

H -3.86232 -1.794073 2.002666

C 0.524807 -0.430029 0.832646

C 1.45891 -1.317429 0.374797

O 0.975136 -2.61392 0.409708

C 2.798521 -1.190686 -0.205881

C 3.085632 -0.177176 -1.129984

C 3.803533 -2.105765 0.122419

C 4.34675 -0.06524 -1.692819

H 2.310236 0.527889 -1.411622

C 5.069219 -2.005875 -0.440809

H 3.597902 -2.901554 0.830905

C 5.344589 -0.980014 -1.345951

H 4.568949 0.718579 -2.408892

H 5.846379 -2.716878 -0.176681

C 0.677083 1.029654 0.995262

C 1.942621 1.517695 1.33385

C -0.405628 1.916313 0.801512

C 2.177157 2.884708 1.407121

H 2.758668 0.835828 1.538809

C -0.111082 3.292947 0.82482

C 1.156239 3.782522 1.124408

H 1.327881 4.853965 1.136197

C -2.352136 0.515607 1.762521

H -1.897631 0.85728 2.698646

C -1.864174 1.504241 0.651877

H -2.452429 2.387973 0.91559

C -2.305942 1.104535 -0.748801

C -1.466903 0.458013 -1.662771

C -3.617314 1.377132 -1.152853

C -1.922993 0.072756 -2.916823

H -0.433795 0.252574 -1.403855

C -4.091882 0.994163 -2.40338

H -4.284475 1.912154 -0.482041

C -3.242791 0.331774 -3.287266

H -1.262242 -0.427722 -3.617069

H -5.113883 1.216937 -2.696493

O -3.74726 0.653798 1.959702

O -3.646654 -0.071681 -4.529247

O -1.055902 4.255636 0.57694

O 3.435037 3.293656 1.742635

O 6.568021 -0.826031 -1.926248

H -4.574991 0.161707 -4.650905

H -1.777588 3.891983 0.051612

H 3.457307 4.257523 1.777447

H 7.1663 -1.512411 -1.605607

H -4.19432 0.31905 1.171325

O -3.409821 -4.262704 1.621151

H -3.040648 -5.12916 1.41154

MalibatolA_Conf64.log

Energy (E) = -1605.16530081 Hartree

Enthalpy (H) = -1604.708791 Hartree

Gibbs free energy (G) = -1604.796388 Hartree

Charge = 0, Spin = 1

C -2.480562 -3.291119 1.43431

C -1.194922 -3.617171 1.019542

C -0.327212 -2.543838 0.873902

C -0.663413 -1.218673 1.130896

C -1.970373 -0.91066 1.527006

C -2.864665 -1.958626 1.679233

H -0.896324 -4.638089 0.809322

H -3.886719 -1.769737 1.986475

C 0.517878 -0.434624 0.849771

C 1.448851 -1.327814 0.396887

O 0.957619 -2.621513 0.431512

C 2.787667 -1.207783 -0.186761

C 3.788164 -2.134741 0.128917

C 3.077033 -0.191478 -1.10471

C 5.049182 -2.042391 -0.442357

H 3.579335 -2.933292 0.833364

C 4.336321 -0.086614 -1.676365

H 2.306609 0.522552 -1.377143

C 5.327183 -1.013516 -1.343781

H 5.826369 -2.757422 -0.194938

H 4.553255 0.703449 -2.389237

C 0.677917 1.024294 1.011357

C 1.943349 1.505211 1.360337

C -0.397471 1.916891 0.805823

C 2.185277 2.870975 1.432769

H 2.753477 0.81853 1.572883

C -0.095127 3.291827 0.828522

C 1.172285 3.774308 1.13887

H 1.350601 4.844693 1.149579

C -2.361187 0.530455 1.752127

H -1.913999 0.873037 2.691439

C -1.856548 1.512348 0.642917

H -2.442029 2.400473 0.897492

C -2.285473 1.111161 -0.761404

C -1.440108 0.458012 -1.664878

C -3.590366 1.391229 -1.180887

C -1.883989 0.07438 -2.923777

H -0.411444 0.246069 -1.39381

C -4.052795 1.009967 -2.436496

H -4.261789 1.931424 -0.518579

C -3.197553 0.341845 -3.31004

H -1.218258 -0.430901 -3.615812

H -5.069835 1.239045 -2.74181

O -3.757368 0.677625 1.935147

O -3.588939 -0.058962 -4.556898

O -1.032063 4.259369 0.568995

O 3.442545 3.273624 1.778118

O 6.581181 -0.96249 -1.874785

H -4.513521 0.182516 -4.690836

H -1.747395 3.900435 0.031702

H 3.470161 4.23749 1.809121

H 6.645918 -0.210771 -2.476781

H -4.198763 0.343412 1.143372

O -3.445624 -4.242029 1.614808

H -3.080496 -5.111039 1.408639

MalibatolA_Conf65.log

Energy (E) = -1605.16554908 Hartree

Enthalpy (H) = -1604.708965 Hartree

Gibbs free energy (G) = -1604.796306 Hartree

Charge = 0, Spin = 1

C -2.469957 -3.314274 1.402954

C -1.183718 -3.631286 0.982925

C -0.320698 -2.553027 0.845872

C -0.661984 -1.231566 1.114953

C -1.96932 -0.932598 1.516508

C -2.858937 -1.985639 1.660701

H -0.881037 -4.649053 0.763491

H -3.880974 -1.803904 1.972261

C 0.516018 -0.44027 0.840479

C 1.450603 -1.325866 0.379799

O 0.964383 -2.621612 0.402512

C 2.790954 -1.195862 -0.198364

C 3.795338 -2.118334 0.118151

C 3.079318 -0.172536 -1.108781

C 5.059139 -2.014813 -0.444962

H 3.587345 -2.921957 0.817059

C 4.341421 -0.05631 -1.672041

H 2.306094 0.538432 -1.381317

C 5.336086 -0.978963 -1.338726

H 5.839367 -2.726287 -0.196869

H 4.557813 0.739291 -2.378891

C 0.670429 1.017623 1.015126

C 1.937614 1.50025 1.362285

C -0.407076 1.908038 0.823526

C 2.176649 2.865761 1.443152

H 2.746309 0.807184 1.565523

C -0.107875 3.285258 0.857053

C 1.158454 3.7683 1.16329

H 1.340336 4.836694 1.184662

C -2.365506 0.504217 1.758568

H -1.919338 0.837107 2.701846

C -1.865885 1.502105 0.661698

H -2.452149 2.385444 0.931027

C -2.299167 1.120026 -0.746494

C -3.607142 1.40558 -1.158007

C -1.457534 0.479864 -1.660203

C -4.073474 1.041562 -2.415498

H -4.275788 1.935697 -0.484804

C -1.906186 0.112849 -2.924092

H -0.427771 0.264123 -1.396364

C -3.220838 0.385786 -3.301737

H -5.089239 1.270171 -2.720655

H -1.236874 -0.383327 -3.621388

O -3.762227 0.643363 1.943608

O -3.71796 0.044517 -4.528634

O -1.050856 4.250819 0.611659

O 3.398242 3.374657 1.776678

O 6.59304 -0.916958 -1.861328

H -3.028989 -0.398022 -5.039015

H -1.767388 3.89371 0.074866

H 4.020087 2.649237 1.912977

H 6.656496 -0.162569 -2.460106

H -4.202481 0.314607 1.1489

O -3.430869 -4.270439 1.577077

H -3.062209 -5.136599 1.365406

MalibatolA_Conf66.log

Energy (E) = -1605.16452854 Hartree

Enthalpy (H) = -1604.708760 Hartree

Gibbs free energy (G) = -1604.796242 Hartree

Charge = 0, Spin = 1

C 3.054529 2.508659 1.886775

C 1.793963 3.05068 1.663131

C 0.799327 2.134492 1.347859

C 0.994355 0.760126 1.274428

C 2.27896 0.239208 1.474768

C 3.298162 1.125226 1.779333

H 1.607357 4.11759 1.714425

H 4.314522 0.772959 1.915247

C -0.280604 0.18296 0.909638

C -1.125615 1.243867 0.743569

O -0.489432 2.43991 1.029355

C -2.502952 1.382259 0.266686

C -3.369846 2.317748 0.844317

C -2.966689 0.602556 -0.79945

C -4.670878 2.460691 0.383933

H -3.024654 2.934963 1.667371

C -4.267733 0.732536 -1.261677

H -2.298842 -0.108478 -1.27524

C -5.124244 1.66232 -0.667437

H -5.344927 3.181493 0.83409

H -4.621926 0.125718 -2.089797

C -0.602563 -1.255358 0.808235

C -1.870344 -1.678491 1.21133

C 0.347965 -2.182406 0.327142

C -2.235813 -3.014421 1.087412

H -2.582904 -0.974114 1.623154

C -0.071671 -3.514458 0.177734

C -1.345071 -3.935959 0.555007

H -1.623768 -4.976896 0.425742

C 2.49444 -1.245881 1.28921

H 2.042271 -1.769439 2.141168

C 1.783749 -1.790862 0.012663

H 2.350044 -2.709035 -0.172283

C 1.932129 -0.91506 -1.230426

C 3.151166 -0.291579 -1.524973

C 0.878644 -0.73192 -2.127088

C 3.30804 0.497536 -2.656351

H 3.993792 -0.418718 -0.854075

C 1.02004 0.052232 -3.269134

H -0.079887 -1.205111 -1.938845

C 2.237525 0.673961 -3.53345

H 4.256386 0.980175 -2.869299

H 0.1848 0.183627 -3.95157

O 3.866368 -1.598851 1.210173

O 2.439026 1.462557 -4.633601

O 0.728909 -4.501363 -0.33387

O -3.489943 -3.366355 1.49474

O -6.411333 1.832866 -1.08169

H 1.627089 1.491869 -5.15369

H 1.465001 -4.119953 -0.825187

H -3.614433 -4.31341 1.358529

H -6.59924 1.217895 -1.80152

H 4.246121 -1.510275 2.09175

O 4.132607 3.288922 2.200354

H 3.859758 4.213681 2.237239

MalibatolA_Conf67.log

Energy (E) = -1605.16540341 Hartree

Enthalpy (H) = -1604.709050 Hartree

Gibbs free energy (G) = -1604.796241 Hartree

Charge = 0, Spin = 1

C -2.449292 -3.350855 1.374465

C -1.162564 -3.656428 0.945565

C -0.307238 -2.573536 0.813237

C -0.654308 -1.25475 1.09512

C -1.96121 -0.965786 1.499184

C -2.845506 -2.026084 1.638518

H -0.86159 -4.672471 0.721546

H -3.867253 -1.839934 1.953641

C 0.520741 -0.455907 0.829434

C 1.458191 -1.332645 0.358097

O 0.977978 -2.630773 0.367129

C 2.797448 -1.190083 -0.219438

C 3.804318 -2.115505 0.080193

C 3.082172 -0.151091 -1.11318

C 5.067109 -1.999592 -0.482768

H 3.599064 -2.931285 0.765674

C 4.343191 -0.022484 -1.676017

H 2.306879 0.562487 -1.372717

C 5.340435 -0.948259 -1.359414

H 5.849266 -2.713405 -0.247819

H 4.556841 0.785185 -2.369895

C 0.670969 0.999525 1.026157

C 1.937037 1.480012 1.380532

C -0.409132 1.890025 0.850593

C 2.172456 2.844591 1.483586

H 2.747807 0.785878 1.571486

C -0.113711 3.267395 0.906694

C 1.151492 3.748857 1.219749

H 1.330471 4.817247 1.258552

C -2.369306 0.4654 1.755394

H -1.937761 0.787894 2.709037

C -1.866785 1.484033 0.680053

H -2.454895 2.361198 0.965137

C -2.297306 1.127636 -0.735424

C -1.454557 0.496548 -1.656835

C -3.601111 1.425235 -1.146212

C -1.900058 0.150613 -2.925796

H -0.426928 0.272563 -1.391719

C -4.065167 1.081595 -2.412308

H -4.270312 1.948785 -0.468424

C -3.212539 0.434517 -3.304119

H -1.236731 -0.337909 -3.632009

H -5.081318 1.323208 -2.710826

O -3.769647 0.591042 1.923959

O -3.604683 0.069915 -4.561785

O -1.05976 4.234419 0.678844

O 3.393008 3.351136 1.824261

O 6.596503 -0.874286 -1.882486

H -4.528224 0.318144 -4.690153

H -1.774462 3.885694 0.13413

H 4.016841 2.625073 1.947443

H 6.656829 -0.109992 -2.468875

H -4.194721 0.284336 1.112019

O -3.322319 -4.392976 1.519506

H -4.17307 -4.058377 1.828005

MalibatolA_Conf68.log

Energy (E) = -1605.16529347 Hartree

Enthalpy (H) = -1604.708929 Hartree

Gibbs free energy (G) = -1604.796236 Hartree

Charge = 0, Spin = 1

C -2.450943 -3.322407 1.422016

C -1.166567 -3.635698 0.991818

C -0.309774 -2.555986 0.842721

C -0.654034 -1.233209 1.108029

C -1.958278 -0.936994 1.515866

C -2.843461 -1.993789 1.672738

H -0.868026 -4.654975 0.779526

H -3.862923 -1.802386 1.992206

C 0.52044 -0.439183 0.826815

C 1.456001 -1.323372 0.366116

O 0.974033 -2.620729 0.39373

C 2.797421 -1.191019 -0.208749

C 3.087942 -0.167701 -1.120885

C 3.80148 -2.10914 0.113981

C 4.351323 -0.049262 -1.67721

H 2.31354 0.540076 -1.398318

C 5.069501 -2.002572 -0.442653

H 3.593311 -2.912522 0.81304

C 5.348207 -0.967118 -1.335799

H 4.576044 0.742292 -2.383913

H 5.84569 -2.716042 -0.182439

C 0.671423 1.019099 1.001021

C 1.93491 1.505249 1.349928

C -0.41102 1.90667 0.810617

C 2.167762 2.87172 1.436806

H 2.750691 0.822297 1.552432

C -0.117886 3.283278 0.847008

C 1.147288 3.771274 1.157244

H 1.318069 4.842712 1.179004

C -2.360496 0.498929 1.754267

H -1.913052 0.83753 2.694789

C -1.868782 1.495119 0.652099

H -2.457571 2.377142 0.920201

C -2.305245 1.107163 -0.753343

C -3.615357 1.387775 -1.161603

C -1.464197 0.468488 -1.668418

C -4.083871 1.020745 -2.417355

H -4.283803 1.917521 -0.487885

C -1.914988 0.098557 -2.930765

H -0.432975 0.256699 -1.407103

C -3.231338 0.367156 -3.305399

H -5.101135 1.24599 -2.720004

H -1.245663 -0.395893 -3.629248

O -3.757201 0.631823 1.943787

O -3.730247 0.024058 -4.531072

O -1.063571 4.24676 0.603115

O 3.423673 3.278949 1.781557

O 6.574073 -0.806377 -1.909073

H -3.040449 -0.414867 -5.043393

H -1.775528 3.889894 0.060071

H 3.443943 4.242366 1.827815

H 7.170473 -1.497426 -1.595123

H -4.198414 0.306261 1.148341

O -3.325918 -4.360691 1.582035

H -4.17573 -4.019658 1.88591

MalibatolA_Conf69.log

Energy (E) = -1605.16469373 Hartree

Enthalpy (H) = -1604.708831 Hartree

Gibbs free energy (G) = -1604.796181 Hartree

Charge = 0, Spin = 1

C 3.017803 2.550478 1.880027

C 1.752053 3.076311 1.64743

C 0.769357 2.146458 1.334654

C 0.980748 0.774059 1.271128

C 2.270242 0.269416 1.481182

C 3.277861 1.169203 1.783836

H 1.553011 4.141348 1.690424

H 4.297395 0.829281 1.927169

C -0.286011 0.179698 0.90511

C -1.142966 1.229458 0.729536

O -0.521686 2.434606 1.0097

C -2.520488 1.350607 0.248313

C -2.975653 0.560147 -0.815713

C -3.395146 2.282255 0.816548

C -4.273952 0.679343 -1.284407

H -2.300508 -0.149062 -1.283922

C -4.696244 2.414555 0.348855

H -3.058543 2.907188 1.637305

C -5.139011 1.608618 -0.700639

H -4.626057 0.069862 -2.109704

H -5.371151 3.137213 0.797733

C -0.590553 -1.262568 0.810272

C -1.857992 -1.697471 1.207674

C 0.371584 -2.181543 0.34193

C -2.208465 -3.037552 1.088108

H -2.575905 -0.992799 1.613217

C -0.033932 -3.520235 0.196914

C -1.303948 -3.952649 0.566837

H -1.577545 -4.994228 0.442973

C 2.50336 -1.214416 1.308509

H 2.053045 -1.736293 2.16246

C 1.805196 -1.777555 0.032998

H 2.381923 -2.69137 -0.140643

C 1.951391 -0.911206 -1.216978

C 3.165388 -0.277334 -1.510348

C 0.901342 -0.748041 -2.121539

C 3.320691 0.502032 -2.64868

H 4.005294 -0.388702 -0.83326

C 1.041336 0.026068 -3.270555

H -0.053235 -1.229575 -1.93435

C 2.2537 0.658055 -3.533917

H 4.265085 0.992726 -2.860802

H 0.208919 0.141698 -3.95929

O 3.879604 -1.552017 1.238781

O 2.453344 1.437371 -4.640964

O 0.782469 -4.499747 -0.304469

O -3.435687 -3.504603 1.460963

O -6.404246 1.691593 -1.200081

H 1.643841 1.453325 -5.165458

H 1.511736 -4.111826 -0.800792

H -3.977101 -2.766096 1.765563

H -6.891883 2.375075 -0.724035

H 4.253099 -1.455957 2.122205

O 4.085178 3.345766 2.192292

H 3.800617 4.267286 2.22091

MalibatolA_Conf70.log

Energy (E) = -1605.16537707 Hartree

Enthalpy (H) = -1604.708849 Hartree

Gibbs free energy (G) = -1604.796139 Hartree

Charge = 0, Spin = 1

C -2.449663 -3.317666 1.422812

C -1.164204 -3.631023 0.997859

C -0.306549 -2.54971 0.85148

C -0.652336 -1.228327 1.115159

C -1.958809 -0.933312 1.522512

C -2.843037 -1.989537 1.676433

H -0.857985 -4.648567 0.782284

H -3.864076 -1.810914 1.99303

C 0.521331 -0.433447 0.831985

C 1.458087 -1.317588 0.373223

O 0.977089 -2.615223 0.403788

C 2.798743 -1.18667 -0.20417

C 3.085493 -0.171451 -1.126486

C 3.805325 -2.099441 0.125711

C 4.347636 -0.05581 -1.686287

H 2.309014 0.532043 -1.409084

C 5.072093 -1.995763 -0.434374

H 3.600072 -2.896349 0.833046

C 5.346949 -0.968464 -1.338035

H 4.569569 0.729303 -2.401025

H 5.850435 -2.704967 -0.168904

C 0.670692 1.02594 0.999821

C 1.935287 1.515232 1.340328

C -0.41357 1.911294 0.809131

C 2.167369 2.882385 1.418212

H 2.752576 0.834258 1.543231

C -0.1215 3.288412 0.837131

C 1.144859 3.779322 1.138426

H 1.314533 4.851031 1.153699

C -2.359793 0.502767 1.761035

H -1.908945 0.841693 2.699934

C -1.871368 1.497702 0.656458

H -2.460903 2.379272 0.92464

C -2.310944 1.106325 -0.747185

C -3.622562 1.383812 -1.15258

C -1.471548 0.465614 -1.662521

C -4.094146 1.012248 -2.405893

H -4.289886 1.913723 -0.477871

C -1.925513 0.090965 -2.922265

H -0.439399 0.255671 -1.403324

C -3.243277 0.356791 -3.29407

H -5.112599 1.234938 -2.706439

H -1.257844 -0.405369 -3.621009

O -3.755863 0.636728 1.95458

O -3.745189 0.008607 -4.517061

O -1.068015 4.250197 0.592259

O 3.424512 3.292483 1.755109

O 6.571297 -0.811022 -1.915431

H -3.056019 -0.430578 -5.030063

H -1.789162 3.886948 0.065929

H 3.444938 4.256254 1.793486

H 7.170179 -1.496844 -1.594722

H -4.199812 0.302246 1.164341

O -3.405273 -4.277349 1.606548

H -3.033427 -5.142748 1.39738

MalibatolA_Conf71.log

Energy (E) = -1605.16533810 Hartree

Enthalpy (H) = -1604.708732 Hartree

Gibbs free energy (G) = -1604.796033 Hartree

Charge = 0, Spin = 1

C -2.480768 -3.295031 1.429534

C -1.19467 -3.61958 1.014981

C -0.327998 -2.545276 0.870241

C -0.665615 -1.220531 1.127492

C -1.972907 -0.914104 1.523751

C -2.866163 -1.963052 1.675198

H -0.894827 -4.640112 0.804657

H -3.888363 -1.77541 1.98272

C 0.515212 -0.435234 0.847738

C 1.447528 -1.327475 0.395708

O 0.957349 -2.621613 0.429075

C 2.78782 -1.206371 -0.184382

C 3.788966 -2.130744 0.136745

C 3.078509 -0.1912 -1.103123

C 5.051889 -2.03697 -0.430045

H 3.579149 -2.928249 0.842084

C 4.339697 -0.084917 -1.670368

H 2.307731 0.520922 -1.379525

C 5.331183 -1.009211 -1.332341

H 5.829637 -2.749864 -0.178265

H 4.557774 0.704366 -2.383752

C 0.673988 1.023697 1.010553

C 1.939102 1.505475 1.359655

C -0.402181 1.915615 0.806225

C 2.179963 2.871363 1.433053

H 2.749855 0.819432 1.571801

C -0.100907 3.290793 0.82979

C 1.166196 3.774103 1.140041

H 1.343582 4.844634 1.151457

C -2.364981 0.526297 1.750972

H -1.918077 0.867694 2.690853

C -1.861124 1.510506 0.64357

H -2.446772 2.397904 0.900442

C -2.290893 1.112678 -0.761387

C -3.597479 1.395008 -1.179365

C -1.447418 0.462391 -1.666262

C -4.060794 1.018785 -2.434333

H -4.267277 1.933135 -0.513721

C -1.892987 0.083028 -2.927652

H -0.418581 0.24831 -1.397448

C -3.206279 0.353629 -3.311679

H -5.075462 1.245397 -2.744596

H -1.222268 -0.420734 -3.618143

O -3.761287 0.671936 1.934263

O -3.700206 0.001098 -4.536654

O -1.038667 4.257773 0.57128

O 3.436997 3.274747 1.778418

O 6.587096 -0.956752 -1.858648

H -3.009193 -0.444509 -5.04148

H -1.754444 3.898472 0.034838

H 3.463722 4.238601 1.810653

H 6.652965 -0.205878 -2.461575

H -4.202616 0.337392 1.142605

O -3.444931 -4.246928 1.609347

H -3.078686 -5.115626 1.403866

MalibatolA_Conf72.log

Energy (E) = -1605.16546028 Hartree

Enthalpy (H) = -1604.708978 Hartree

Gibbs free energy (G) = -1604.795982 Hartree

Charge = 0, Spin = 1

C -2.456214 -3.341127 1.383114

C -1.169645 -3.649831 0.955988

C -0.312783 -2.568503 0.820629

C -0.65842 -1.248315 1.097506

C -1.964991 -0.956357 1.500529

C -2.8507 -2.01501 1.643037

H -0.869857 -4.667029 0.735639

H -3.872186 -1.826599 1.957659

C 0.517726 -0.451799 0.829678

C 1.454675 -1.331452 0.362816

O 0.972803 -2.628955 0.37598

C 2.795512 -1.192809 -0.21203

C 3.801057 -2.116983 0.095788

C 3.083642 -0.158421 -1.109974

C 5.065853 -2.004251 -0.46329

H 3.593219 -2.929118 0.784813

C 4.346676 -0.033013 -1.669045

H 2.309552 0.554215 -1.37561

C 5.342574 -0.957409 -1.344229

H 5.847022 -2.716972 -0.221831

H 4.56304 0.771241 -2.366043

C 0.66931 1.004252 1.020846

C 1.935552 1.484967 1.374335

C -0.409802 1.895077 0.840993

C 2.172131 2.849729 1.472308

H 2.745509 0.790893 1.568891

C -0.113116 3.272382 0.891759

C 1.152275 3.753906 1.203934

H 1.332198 4.822277 1.238668

C -2.370725 0.47626 1.752384

H -1.93701 0.801462 2.704112

C -1.867877 1.489883 0.672372

H -2.454924 2.368938 0.953848

C -2.299765 1.127842 -0.741218

C -3.605704 1.424393 -1.151334

C -1.459693 0.493585 -1.660466

C -4.071993 1.075709 -2.413314

H -4.272666 1.950913 -0.473627

C -1.908266 0.142321 -2.92873

H -0.431453 0.269936 -1.397273

C -3.221352 0.424828 -3.305049

H -5.086238 1.312264 -2.717461

H -1.240316 -0.34957 -3.630336

O -3.770529 0.604714 1.923112

O -3.718714 0.09777 -4.535702

O -1.058088 4.239292 0.659141

O 3.392824 3.356478 1.812193

O 6.600557 -0.88644 -1.863098

H -3.032699 -0.346395 -5.048738

H -1.772654 3.888901 0.115307

H 4.015898 2.63035 1.938771

H 6.663389 -0.125265 -2.453281

H -4.198018 0.294177 1.113946

O -3.33082 -4.381515 1.530886

H -4.181694 -4.044488 1.836425

MalibatolA_Conf73.log

Energy (E) = -1605.16468542 Hartree

Enthalpy (H) = -1604.708816 Hartree

Gibbs free energy (G) = -1604.795909 Hartree

Charge = 0, Spin = 1

C 3.046042 2.52511 1.876649

C 1.784859 3.063158 1.646862

C 0.792718 2.142971 1.335328

C 0.99099 0.76866 1.270699

C 2.276074 0.251548 1.477439

C 3.292796 1.141502 1.77873

H 1.596072 4.129997 1.691105

H 4.309434 0.79197 1.919505

C -0.281927 0.186497 0.906957

C -1.129179 1.244399 0.733227

O -0.496286 2.443546 1.01287

C -2.50638 1.375975 0.254005

C -3.377561 2.310265 0.827171

C -2.966183 0.589632 -0.808968

C -4.67896 2.445643 0.365535

H -3.035377 2.932515 1.647698

C -4.267607 0.711912 -1.27226

H -2.29502 -0.120564 -1.281357

C -5.128326 1.640693 -0.682572

H -5.356285 3.165648 0.812035

H -4.618811 0.100105 -2.097998

C -0.600231 -1.252841 0.8124

C -1.870086 -1.676479 1.214124

C 0.351782 -2.18028 0.340253

C -2.233119 -3.013196 1.09517

H -2.580231 -0.965482 1.622312

C -0.066344 -3.515188 0.195933

C -1.338991 -3.936259 0.569996

H -1.622448 -4.975264 0.446679

C 2.494655 -1.234314 1.302362

H 2.04134 -1.75294 2.156708

C 1.788147 -1.789118 0.027714

H 2.355799 -2.708046 -0.148926

C 1.939089 -0.922131 -1.221235

C 3.157911 -0.298376 -1.516234

C 0.888445 -0.748118 -2.123068

C 3.317304 0.482045 -2.653268

H 3.998423 -0.418481 -0.841406

C 1.032454 0.027181 -3.270812

H -0.0698 -1.221819 -1.9346

C 2.249614 0.649314 -3.53559

H 4.265416 0.964987 -2.866571

H 0.199471 0.151445 -3.957336

O 3.867411 -1.585136 1.229054

O 2.453436 1.429546 -4.641221

O 0.73945 -4.501684 -0.308821

O -3.463371 -3.468625 1.472465

O -6.415821 1.80364 -1.098307

H 1.643008 1.453609 -5.163947

H 1.471803 -4.119885 -0.805382

H -3.995107 -2.725121 1.78202

H -6.599709 1.186073 -1.816987

H 4.244383 -1.491435 2.111277

O 4.121767 3.309632 2.187558

H 3.84705 4.234114 2.217114

VaticahainolB_Conf1.log

Energy (E) = -1680.35096914 Hartree

Enthalpy (H) = -1679.890252 Hartree

Gibbs free energy (G) = -1679.980610 Hartree

Charge = 0, Spin = 1

C 1.675645 3.541017 1.23562

C 0.655506 3.549696 0.203001

C 0.046692 2.381609 -0.086168

C 0.337947 1.121216 0.576759

C 1.38142 1.098443 1.584659

C 2.000029 2.254528 1.889092

H 0.416975 4.473523 -0.309604

H 2.789184 2.284712 2.630877

C -0.476894 0.169941 0.067684

C -1.36601 0.831609 -0.997928

O -0.905186 2.215707 -1.018681

C -2.849258 0.876663 -0.681431

C -3.805967 0.537939 -1.637398

C -3.269071 1.288837 0.584328

C -5.160116 0.599831 -1.334548

H -3.496747 0.212653 -2.623434

C -4.618944 1.355402 0.897511

H -2.538329 1.558183 1.341481

C -5.569545 1.007349 -0.064901

H -5.906806 0.333195 -2.07469

H -4.939756 1.673079 1.885056

C -0.517848 -1.280115 0.305405

C -1.696886 -1.973102 -0.003701

C 0.622052 -1.977831 0.759222

C -1.758428 -3.352563 0.142354

H -2.577142 -1.448674 -0.351489

C 0.525743 -3.374188 0.87383

C -0.646804 -4.061453 0.579908

H -0.695407 -5.140874 0.682918

C 1.732474 -0.224155 2.197785

H 0.881222 -0.5499 2.813218

C 1.938765 -1.315644 1.105785

H 2.539104 -2.077447 1.60936

C 2.741883 -0.844065 -0.10656

C 3.979393 -0.215356 0.081372

C 2.299253 -1.020431 -1.418338

C 4.736563 0.235221 -0.991578

H 4.352508 -0.066606 1.088724

C 3.046639 -0.576026 -2.506127

H 1.350317 -1.509986 -1.61318

C 4.268275 0.057869 -2.29369

H 5.691795 0.724203 -0.830364

H 2.680109 -0.721613 -3.518429

O 2.87203 -0.057713 3.016418

O 2.284335 4.57054 1.56769

O 5.046635 0.519711 -3.319459

O 1.634346 -4.054183 1.282821

O -2.885384 -4.064394 -0.138277

O -6.908457 1.045219 0.184695

O -1.170899 0.27529 -2.251587

H -0.219566 0.184099 -2.400583

H 4.612047 0.326934 -4.159173

H 1.432659 -4.996452 1.332727

H -3.575672 -3.46462 -0.447105

H -7.058883 1.336338 1.092471

H 3.007982 -0.88561 3.490558

VaticahainolB_Conf2.log

Energy (E) = -1680.35073812 Hartree

Enthalpy (H) = -1679.890053 Hartree

Gibbs free energy (G) = -1679.980390 Hartree

Charge = 0, Spin = 1

C 1.667091 3.533077 1.256311

C 0.648266 3.547867 0.222496

C 0.04079 2.38119 -0.075392

C 0.332124 1.116875 0.579956

C 1.375207 1.087879 1.588125

C 1.992593 2.24239 1.900879

H 0.409611 4.47497 -0.284097

H 2.781229 2.268257 2.643391

C -0.482764 0.168769 0.065457

C -1.370671 0.835782 -0.997523

O -0.909435 2.220315 -1.010296

C -2.854272 0.880462 -0.683421

C -3.809443 0.558359 -1.646538

C -3.276067 1.279461 0.585841

C -5.164259 0.624372 -1.347601

H -3.498707 0.243686 -2.635524

C -4.626635 1.349312 0.895411

H -2.546355 1.535541 1.348556

C -5.57581 1.018589 -0.074512

H -5.909822 0.370966 -2.093512

H -4.949028 1.65641 1.885777

C -0.523576 -1.282673 0.295799

C -1.704273 -1.972613 -0.005308

C 0.62095 -1.982902 0.739167

C -1.767303 -3.352598 0.140743

H -2.590869 -1.454886 -0.343214

C 0.526906 -3.378407 0.843531

C -0.650629 -4.064617 0.558557

H -0.691434 -5.145729 0.660093

C 1.727512 -0.238797 2.191599

H 0.876219 -0.570554 2.803759

C 1.936701 -1.321665 1.091416

H 2.536354 -2.086965 1.590563

C 2.742778 -0.840277 -0.114761

C 2.299024 -0.998976 -1.430018

C 3.982655 -0.222241 0.080612

C 3.049223 -0.548544 -2.511269

H 1.346733 -1.480454 -1.62911

C 4.744605 0.234914 -0.988227

H 4.356817 -0.086164 1.089375

C 4.276208 0.073696 -2.292037

H 2.689516 -0.677682 -3.526863

H 5.703466 0.714887 -0.81322

O 2.866125 -0.076861 3.012488

O 2.274084 4.561006 1.596549

O 4.979221 0.500663 -3.385214

O 1.639378 -4.062132 1.235325

O -2.952117 -3.959605 -0.148164

O -6.915502 1.061455 0.170778

O -1.174065 0.286191 -2.253824

H -0.222602 0.197262 -2.40319

H 5.806225 0.903426 -3.094256

H 1.439492 -5.005008 1.280751

H -2.875144 -4.910137 0.000638

H -7.067454 1.339763 1.082306

H 3.002654 -0.90794 3.480889

VaticahainolB_Conf3.log

Energy (E) = -1680.35075167 Hartree

Enthalpy (H) = -1679.890141 Hartree

Gibbs free energy (G) = -1679.980364 Hartree

Charge = 0, Spin = 1

C 1.687337 3.526832 1.259855

C 0.666591 3.547087 0.227995

C 0.053501 2.383325 -0.069716

C 0.340859 1.117094 0.583561

C 1.385562 1.08267 1.589902

C 2.008321 2.234255 1.902775

H 0.430752 4.475717 -0.277106

H 2.798449 2.255996 2.643834

C -0.478682 0.172834 0.069444

C -1.366104 0.84497 -0.991187

O -0.899999 2.227639 -1.002276

C -2.8489 0.893995 -0.673988

C -3.266958 1.306853 0.593966

C -3.805985 0.558902 -1.628347

C -4.614755 1.374799 0.908304

H -2.533956 1.573963 1.349772

C -5.161441 0.623346 -1.324011

H -3.499355 0.233919 -2.615272

C -5.567657 1.028261 -0.053013

H -4.941939 1.689615 1.893276

H -5.904309 0.357064 -2.070065

C -0.524517 -1.278702 0.298668

C -1.709074 -1.963549 0.001381

C 0.61839 -1.983982 0.73831

C -1.777569 -3.343296 0.147018

H -2.594791 -1.441811 -0.332434

C 0.518732 -3.379117 0.842609

C -0.662551 -4.060245 0.560772

H -0.70779 -5.141203 0.662118

C 1.733513 -0.246224 2.191095

H 0.881483 -0.575707 2.803441

C 1.937261 -1.328026 1.088633

H 2.535703 -2.096056 1.585079

C 2.741824 -0.846709 -0.118389

C 3.982892 -0.227021 0.075982

C 2.296995 -1.005167 -1.431542

C 4.741693 0.231907 -0.992235

H 4.357417 -0.092121 1.08477

C 3.045947 -0.552215 -2.514742

H 1.344954 -1.487173 -1.630611

C 4.271362 0.072216 -2.295927

H 5.699744 0.713708 -0.826184

H 2.677896 -0.683887 -3.528386

O 2.873439 -0.090014 3.011246

O 2.299489 4.551836 1.599757

O 5.051622 0.541431 -3.316923

O 1.629399 -4.067569 1.231276

O -2.966211 -3.944739 -0.13808

O -6.879255 1.102815 0.308177

O -1.173592 0.296573 -2.248692

H -0.222805 0.201708 -2.398702

H 4.615297 0.361102 -4.1585

H 1.42578 -5.009628 1.277471

H -2.893881 -4.895504 0.011504

H -7.43354 0.827735 -0.432443

H 3.006244 -0.921778 3.479515

VaticahainolB_Conf4.log

Energy (E) = -1680.35099185 Hartree

Enthalpy (H) = -1679.890185 Hartree

Gibbs free energy (G) = -1679.980204 Hartree

Charge = 0, Spin = 1

C 1.676298 3.541157 1.226878

C 0.653977 3.550334 0.19647

C 0.043126 2.382765 -0.090579

C 0.334215 1.122485 0.572599

C 1.378378 1.099616 1.579729

C 1.999423 2.255054 1.881675

H 0.41568 4.473995 -0.31654

H 2.789071 2.285054 2.622938

C -0.481561 0.171412 0.064563

C -1.371587 0.833499 -1.0002

O -0.910592 2.217378 -1.021233

C -2.854517 0.878301 -0.682125

C -3.812197 0.541916 -1.637969

C -3.27303 1.287464 0.585029

C -5.166028 0.603002 -1.333578

H -3.503973 0.219036 -2.625105

C -4.6226 1.353153 0.899773

H -2.541478 1.554986 1.342038

C -5.574154 1.007318 -0.062482

H -5.913477 0.338132 -2.073592

H -4.942434 1.668331 1.888436

C -0.522718 -1.278711 0.301943

C -1.701359 -1.971607 -0.009043

C 0.616774 -1.976801 0.756294

C -1.762886 -3.351233 0.135282

H -2.581309 -1.447001 -0.357287

C 0.520614 -3.37341 0.868633

C -0.651497 -4.060518 0.572792

H -0.699892 -5.1401 0.674172

C 1.726757 -0.222377 2.195428

H 0.874254 -0.545909 2.810322

C 1.933359 -1.315827 1.105606

H 2.5308 -2.077973 1.612023

C 2.741393 -0.847324 -0.104683

C 2.296119 -1.011427 -1.418848

C 3.984669 -0.23473 0.086264

C 3.048011 -0.571147 -2.503082

H 1.341281 -1.488893 -1.615061

C 4.748306 0.212317 -0.985646

H 4.360141 -0.094663 1.093997

C 4.2782 0.046247 -2.288238

H 2.686936 -0.704277 -3.517678

H 5.709729 0.68833 -0.813987

O 2.865633 -0.056251 3.015044

O 2.287675 4.570012 1.556237

O 4.982354 0.463803 -3.38429

O 1.629079 -4.05378 1.27738

O -2.889454 -4.062942 -0.14717

O -6.912798 1.044336 0.188671

O -1.177796 0.27746 -2.254185

H -0.226562 0.188245 -2.405016

H 5.810561 0.866247 -3.096272

H 1.427674 -4.996212 1.325388

H -3.579543 -3.46307 -0.456259

H -7.062217 1.333202 1.097331

H 2.999057 -0.883166 3.491612

VaticahainolB_Conf5.log

Energy (E) = -1680.35077428 Hartree

Enthalpy (H) = -1679.890050 Hartree

Gibbs free energy (G) = -1679.979816 Hartree

Charge = 0, Spin = 1

C 1.688688 3.527508 1.249868

C 0.665471 3.547955 0.220464

C 0.049865 2.384757 -0.074271

C 0.33695 1.118862 0.579776

C 1.382667 1.084581 1.585015

C 2.008218 2.235521 1.894675

H 0.430085 4.476204 -0.285554

H 2.799087 2.257231 2.634939

C -0.483686 0.174655 0.067205

C -1.371909 0.846653 -0.99294

O -0.905775 2.22931 -1.004707

C -2.854455 0.895371 -0.674652

C -3.271761 1.307069 0.593938

C -3.812116 0.56064 -1.628566

C -4.619357 1.374095 0.90936

H -2.538238 1.573913 1.349331

C -5.16736 0.624133 -1.323132

H -3.506075 0.236537 -2.615963

C -5.572812 1.027776 -0.051496

H -4.945954 1.687931 1.894839

H -5.910634 0.358027 -2.068843

C -0.52955 -1.276893 0.2965

C -1.71375 -1.961851 -0.002171

C 0.61321 -1.982437 0.736161

C -1.781921 -3.341792 0.141537

H -2.599416 -1.440092 -0.336035

C 0.514064 -3.37784 0.837833

C -0.666788 -4.059013 0.554491

H -0.711574 -5.140169 0.653897

C 1.728036 -0.243573 2.189025

H 0.874968 -0.570807 2.801107

C 1.931979 -1.327568 1.088889

H 2.527497 -2.095887 1.588342

C 2.741314 -0.84975 -0.116333

C 2.293638 -0.996461 -1.431588

C 3.988138 -0.24628 0.080382

C 3.046889 -0.548258 -2.511665

H 1.335782 -1.466519 -1.631416

C 4.753217 0.208476 -0.987218

H 4.365251 -0.119682 1.089291

C 4.28084 0.059562 -2.29108

H 2.684139 -0.667862 -3.527341

H 5.717548 0.677024 -0.811294

O 2.86748 -0.08749 3.009902

O 2.303835 4.551835 1.586362

O 4.98661 0.485143 -3.383011

O 1.625007 -4.066428 1.225455

O -2.970253 -3.943216 -0.144832

O -6.884195 1.101214 0.310733

O -1.180254 0.298181 -2.250552

H -0.229488 0.205775 -2.402226

H 5.817479 0.879094 -3.090994

H 1.42211 -5.008758 1.269279

H -2.897473 -4.894238 0.002909

H -7.438835 0.826705 -0.429838

H 2.997779 -0.918204 3.480739

VaticahainolB_Conf6.log

Energy (E) = -1680.35063015 Hartree

Enthalpy (H) = -1679.889860 Hartree

Gibbs free energy (G) = -1679.979807 Hartree

Charge = 0, Spin = 1

C 1.709257 3.518617 1.247667

C 0.679015 3.541632 0.224723

C 0.057431 2.380541 -0.066453

C 0.340201 1.116028 0.591697

C 1.391939 1.078525 1.590125

C 2.03042 2.224929 1.888064

H 0.447196 4.469887 -0.282959

H 2.838185 2.241713 2.611658

C -0.477677 0.170245 0.07721

C -1.362184 0.840767 -0.986783

O -0.89744 2.224734 -0.997461

C -2.846434 0.889845 -0.677273

C -3.799489 0.562937 -1.640848

C -3.2709 1.297971 0.588178

C -5.154994 0.633421 -1.346131

H -3.486484 0.240994 -2.626779

C -4.622177 1.372327 0.893535

H -2.542507 1.558136 1.350848

C -5.569304 1.03692 -0.076855

H -5.898953 0.376388 -2.092401

H -4.946844 1.686822 1.880832

C -0.521655 -1.281827 0.304798

C -1.704392 -1.967654 0.002448

C 0.619954 -1.9874 0.748254

C -1.772036 -3.347726 0.146116

H -2.589415 -1.446468 -0.33426

C 0.522034 -3.382542 0.84958

C -0.657875 -4.064205 0.562841

H -0.702416 -5.145349 0.662466

C 1.73084 -0.256559 2.197238

H 0.871185 -0.579454 2.800688

C 1.939058 -1.330507 1.098248

H 2.54224 -2.092359 1.59622

C 2.734583 -0.844972 -0.114637

C 3.953825 -0.181161 0.070835

C 2.30163 -1.047361 -1.425683

C 4.703426 0.276677 -1.004191

H 4.320021 -0.009308 1.077221

C 3.041265 -0.595424 -2.515787

H 1.365907 -1.562783 -1.617623

C 4.245131 0.072353 -2.305951

H 5.6443 0.793271 -0.844808

H 2.682268 -0.761079 -3.527693

O 2.886726 -0.214886 3.011097

O 2.330106 4.541155 1.578749

O 5.014903 0.543719 -3.333761

O 1.632202 -4.070246 1.240644

O -2.959141 -3.949952 -0.143709

O -6.909622 1.084023 0.164192

O -1.163124 0.29127 -2.242783

H -0.211849 0.190341 -2.385923

H 4.588462 0.331427 -4.172952

H 1.429515 -5.012619 1.28428

H -2.886225 -4.900856 0.004735

H -7.063936 1.368019 1.073564

H 2.677125 0.296357 3.801336

VaticahainolB_Conf7.log

Energy (E) = -1680.35063928 Hartree

Enthalpy (H) = -1679.889810 Hartree

Gibbs free energy (G) = -1679.979515 Hartree

Charge = 0, Spin = 1

C 1.707145 3.520574 1.238219

C 0.675576 3.542945 0.21664

C 0.052871 2.381863 -0.072274

C 0.335779 1.117974 0.586968

C 1.387699 1.081438 1.585194

C 2.027518 2.227737 1.880678

H 0.44376 4.470639 -0.292065

H 2.835276 2.245148 2.604272

C -0.482477 0.171747 0.073806

C -1.367766 0.841617 -0.990064

O -0.903188 2.225525 -1.001936

C -2.851769 0.890319 -0.679252

C -3.805602 0.56424 -1.642362

C -3.27522 1.296818 0.587056

C -5.160847 0.633793 -1.346283

H -3.493379 0.243598 -2.628961

C -4.626253 1.370214 0.893782

H -2.54621 1.556355 1.349338

C -5.574128 1.035501 -0.076096

H -5.905412 0.377334 -2.092147

H -4.950137 1.683317 1.88178

C -0.526236 -1.280211 0.302125

C -1.70829 -1.966639 -0.001689

C 0.615208 -1.985353 0.74674

C -1.775325 -3.346795 0.141255

H -2.593194 -1.445896 -0.339363

C 0.518009 -3.380655 0.846955

C -0.661162 -4.06286 0.558662

H -0.705113 -5.144098 0.657527

C 1.72463 -0.252595 2.195435

H 0.863873 -0.5736 2.798352

C 1.933828 -1.32873 1.098952

H 2.534645 -2.090418 1.599958

C 2.734101 -0.846647 -0.112259

C 2.299281 -1.03997 -1.425743

C 3.958741 -0.197101 0.075899

C 3.044311 -0.594319 -2.512667

H 1.357953 -1.544623 -1.618956

C 4.715596 0.255136 -0.998464

H 4.326603 -0.031118 1.082658

C 4.256817 0.058006 -2.300807

H 2.691692 -0.750313 -3.526962

H 5.662382 0.760225 -0.828742

O 2.879533 -0.210202 3.01064

O 2.329658 4.543008 1.56664

O 4.9551 0.479065 -3.399177

O 1.628228 -4.067959 1.238575

O -2.96172 -3.949644 -0.15011

O -6.914229 1.081557 0.166352

O -1.169586 0.291522 -2.245922

H -0.218336 0.192033 -2.390303

H 5.773154 0.90318 -3.113194

H 1.4261 -5.010506 1.281057

H -2.888129 -4.900627 -0.002477

H -7.067728 1.364489 1.076191

H 2.669644 0.303625 3.799107

VaticahainolB_Conf8.log

Energy (E) = -1680.35066400 Hartree

Enthalpy (H) = -1679.889874 Hartree

Gibbs free energy (G) = -1679.979403 Hartree

Charge = 0, Spin = 1

C 1.725108 3.513959 1.244764

C 0.692077 3.54155 0.224875

C 0.065267 2.382952 -0.065169

C 0.345209 1.116969 0.59123

C 1.398553 1.075219 1.587813

C 2.042263 2.219036 1.884548

H 0.462201 4.470948 -0.281578

H 2.851333 2.232811 2.606751

C -0.477166 0.174392 0.077936

C -1.362278 0.849435 -0.983139

O -0.89301 2.231459 -0.993268

C -2.845428 0.902493 -0.668636

C -3.264661 1.316785 0.59842

C -3.801625 0.570847 -1.625146

C -4.612904 1.389371 0.909936

H -2.532082 1.581409 1.355518

C -5.157464 0.640027 -1.323677

H -3.493971 0.244922 -2.611449

C -5.564928 1.046276 -0.053475

H -4.941122 1.705254 1.894223

H -5.899783 0.376531 -2.071279

C -0.525591 -1.277755 0.304255

C -1.711005 -1.959444 0.002979

C 0.614169 -1.987554 0.745876

C -1.783173 -3.339345 0.145655

H -2.594815 -1.435035 -0.331705

C 0.511534 -3.382433 0.846569

C -0.670954 -4.059877 0.560527

H -0.719153 -5.140917 0.659649

C 1.732196 -0.261216 2.194767

H 0.871181 -0.580553 2.798252

C 1.935784 -1.335621 1.095425

H 2.536448 -2.099937 1.592662

C 2.732646 -0.852117 -0.117388

C 3.9569 -0.197658 0.068595

C 2.296179 -1.046666 -1.428438

C 4.708344 0.258299 -1.00589

H 4.325521 -0.031746 1.075098

C 3.037701 -0.596595 -2.518041

H 1.356315 -1.554344 -1.62083

C 4.246812 0.061468 -2.307689

H 5.653171 0.767524 -0.846158

H 2.676186 -0.756123 -3.530034

O 2.888244 -0.224737 3.008446

O 2.351168 4.533907 1.574436

O 5.018833 0.530224 -3.334999

O 1.619613 -4.074254 1.236344

O -2.972744 -3.937237 -0.143262

O -6.877031 1.125448 0.304827

O -1.168282 0.300825 -2.240335

H -0.21773 0.196281 -2.385688

H 4.589829 0.323627 -4.174304

H 1.41281 -5.015635 1.282222

H -2.903494 -4.888351 0.005574

H -7.430562 0.851871 -0.436901

H 2.681992 0.289601 3.797582

VaticahainolB_Conf9.log

Energy (E) = -1680.35067892 Hartree

Enthalpy (H) = -1679.889841 Hartree

Gibbs free energy (G) = -1679.979130 Hartree

Charge = 0, Spin = 1

C 1.724112 3.515678 1.234392

C 0.689898 3.542749 0.215576

C 0.061119 2.384505 -0.071525

C 0.340401 1.119243 0.586576

C 1.394039 1.078182 1.582796

C 2.039743 2.22159 1.87665

H 0.460778 4.471483 -0.29245

H 2.848834 2.235646 2.59883

C -0.482608 0.176387 0.074809

C -1.368202 0.850491 -0.986493

O -0.898819 2.232659 -0.998041

C -2.851083 0.903494 -0.671254

C -3.269612 1.320767 0.595097

C -3.807795 0.567676 -1.625746

C -4.617601 1.392265 0.90781

H -2.536542 1.588704 1.350584

C -5.163433 0.63574 -1.323072

H -3.500704 0.239275 -2.611401

C -5.570144 1.044959 -0.053615

H -4.945289 1.710509 1.891508

H -5.905997 0.368889 -2.069227

C -0.530833 -1.275605 0.302453

C -1.716028 -1.957611 0.00105

C 0.609005 -1.985315 0.744102

C -1.787777 -3.337632 0.142707

H -2.600013 -1.433361 -0.333387

C 0.507069 -3.380401 0.842932

C -0.675126 -4.058152 0.556401

H -0.722769 -5.139348 0.65408

C 1.725478 -0.257199 2.192956

H 0.863444 -0.574711 2.795888

C 1.93027 -1.333935 1.096195

H 2.527993 -2.098177 1.597028

C 2.732749 -0.854437 -0.11449

C 2.295038 -1.03997 -1.428094

C 3.962382 -0.214606 0.074566

C 3.042239 -0.5962 -2.514317

H 1.349813 -1.537024 -1.62199

C 4.721446 0.235633 -0.999054

H 4.332466 -0.05477 1.081502

C 4.259784 0.046358 -2.30156

H 2.687351 -0.74604 -3.528747

H 5.672158 0.733067 -0.828643

O 2.880645 -0.219791 3.008016

O 2.352399 4.535193 1.560859

O 4.960052 0.465931 -3.39922

O 1.615745 -4.071979 1.23142

O -2.977327 -3.935529 -0.146244

O -6.881994 1.123119 0.305898

O -1.175089 0.300869 -2.24337

H -0.224587 0.197275 -2.389796

H 5.780789 0.88437 -3.112572

H 1.410211 -5.013766 1.274751

H -2.907691 -4.886833 0.00117

H -7.435909 0.84694 -0.434584

H 2.673582 0.296179 3.795846

VaticahainolB_Conf10.log

Energy (E) = -1680.35132885 Hartree

Enthalpy (H) = -1679.889440 Hartree

Gibbs free energy (G) = -1679.978020 Hartree

Charge = 0, Spin = 1

C 1.327505 3.76692 0.83337

C 0.268826 3.639478 -0.150075

C -0.271584 2.417292 -0.334246

C 0.139108 1.223512 0.38632

C 1.191245 1.343516 1.374956

C 1.73774 2.557387 1.578526

H -0.050296 4.507874 -0.71323

H 2.514139 2.703161 2.321139

C -0.598907 0.179282 -0.053643

C -1.597277 0.717763 -1.094127

O -1.244165 2.128177 -1.212805

C -3.05482 0.668199 -0.672505

C -4.054303 0.312463 -1.577877

C -3.412473 1.026127 0.628252

C -5.388217 0.30752 -1.191849

H -3.792989 0.030002 -2.590705

C -4.742352 1.025632 1.024639

H -2.648876 1.306689 1.347571

C -5.735289 0.665535 0.111066

H -6.167838 0.029222 -1.892724

H -5.014002 1.300634 2.039357

C -0.489696 -1.260382 0.218311

C -1.575084 -2.074015 -0.144714

C 0.687916 -1.839228 0.741512

C -1.506673 -3.451561 0.000673

H -2.484515 -1.64264 -0.54141

C 0.727991 -3.242449 0.841016

C -0.349154 -4.04643 0.486875

H -0.289066 -5.125688 0.586224

C 1.600211 0.111099 2.13481

H 0.757921 -0.192623 2.767013

C 1.922774 -1.090688 1.204271

H 2.440652 -1.773279 1.880254

C 2.914702 -0.745925 0.099891

C 4.275694 -0.645341 0.413742

C 2.534778 -0.498129 -1.221369

C 5.219144 -0.278781 -0.538103

H 4.612814 -0.87698 1.420619

C 3.466408 -0.136387 -2.188315

H 1.497318 -0.598615 -1.521632

C 4.812471 -0.016138 -1.845429

H 6.270723 -0.207184 -0.280875

H 3.149812 0.049214 -3.210803

O 2.664897 0.365467 3.027887

O 1.889169 4.850265 1.060863

O 5.772028 0.338693 -2.751601

O 1.881215 -3.803999 1.301692

O -2.543741 -4.269854 -0.33064

O -7.057153 0.643775 0.440245

O -1.45072 0.12192 -2.33525

H -0.512023 0.118822 -2.565883

H 5.35991 0.478655 -3.612808

H 1.775286 -4.761156 1.358863

H -3.278648 -3.737744 -0.660331

H -7.164927 0.921909 1.358024

H 3.42825 0.656546 2.512336

VaticahainolB_Conf11.log

Energy (E) = -1680.35134884 Hartree

Enthalpy (H) = -1679.889375 Hartree

Gibbs free energy (G) = -1679.977853 Hartree

Charge = 0, Spin = 1

C 1.330573 3.765639 0.826117

C 0.268583 3.639702 -0.153926

C -0.274401 2.418368 -0.33603

C 0.136492 1.224066 0.383486

C 1.191447 1.342744 1.369279

C 1.740829 2.555697 1.570565

H -0.050606 4.50834 -0.716662

H 2.519706 2.700206 2.310815

C -0.603294 0.180601 -0.055335

C -1.603002 0.720327 -1.094004

O -1.249966 2.130659 -1.21182

C -3.060077 0.67029 -0.670814

C -4.060303 0.31258 -1.574574

C -3.416479 1.029325 0.629985

C -5.393745 0.306748 -1.186895

H -3.799878 0.029105 -2.587357

C -4.74587 1.02801 1.027999

H -2.652219 1.311428 1.348007

C -5.739579 0.665945 0.116015

H -6.173954 0.026833 -1.886476

H -5.016563 1.303909 2.042728

C -0.494071 -1.25943 0.214934

C -1.580281 -2.072304 -0.147407

C 0.683911 -1.839447 0.736009

C -1.512374 -3.450036 -0.00356

H -2.490077 -1.640166 -0.542373

C 0.723476 -3.24285 0.833796

C -0.354451 -4.04601 0.480293

H -0.294698 -5.125393 0.578432

C 1.600114 0.110214 2.12917

H 0.758784 -0.191643 2.763542

C 1.919651 -1.0928 1.19933

H 2.435555 -1.776205 1.875966

C 2.913614 -0.749706 0.096451

C 2.534649 -0.488188 -1.224239

C 4.273747 -0.661838 0.410429

C 3.467507 -0.124231 -2.186795

H 1.496179 -0.579803 -1.524004

C 5.220809 -0.293669 -0.539618

H 4.609991 -0.903367 1.415296

C 4.815029 -0.015682 -1.843496

H 3.160877 0.074388 -3.208481

H 6.27227 -0.233863 -0.273543

O 2.667515 0.363659 3.019258

O 1.895078 4.848033 1.051254

O 5.693904 0.350449 -2.824004

O 1.877087 -3.805328 1.292413

O -2.550354 -4.267468 -0.334114

O -7.06105 0.643325 0.446736

O -1.457737 0.125522 -2.335813

H -0.519273 0.123081 -2.567512

H 6.584609 0.384622 -2.454417

H 1.770667 -4.762441 1.349351

H -3.285445 -3.734692 -0.662318

H -7.168128 0.922562 1.36426

H 3.431365 0.64789 2.500469

VaticahainolB_Conf12.log

Energy (E) = -1680.34932703 Hartree

Enthalpy (H) = -1679.887862 Hartree

Gibbs free energy (G) = -1679.977537 Hartree

Charge = 0, Spin = 1

C 1.349695 3.766318 0.780181

C 0.276928 3.639094 -0.188648

C -0.280324 2.421418 -0.350692

C 0.127411 1.233529 0.379393

C 1.190929 1.353173 1.353815

C 1.755122 2.561892 1.536944

H -0.038477 4.50449 -0.758465

H 2.540679 2.707604 2.269943

C -0.623467 0.189579 -0.036627

C -1.623293 0.72148 -1.081018

O -1.268097 2.13023 -1.211588

C -3.08187 0.676557 -0.663806

C -3.447275 1.082174 0.622622

C -4.073071 0.26902 -1.553694

C -4.775811 1.075016 1.017517

H -2.688311 1.404479 1.329409

C -5.408633 0.256193 -1.167633

H -3.806918 -0.050794 -2.554029

C -5.762007 0.658618 0.119896

H -5.061338 1.385399 2.016734

H -6.177694 -0.066378 -1.863236

C -0.512757 -1.249251 0.242194

C -1.615214 -2.056587 -0.073357

C 0.686032 -1.8359 0.713637

C -1.546735 -3.436703 0.058755

H -2.541136 -1.618145 -0.42119

C 0.738987 -3.241906 0.751088

C -0.359106 -4.038342 0.449932

H -0.276631 -5.117429 0.512329

C 1.584592 0.12272 2.122165

H 0.734924 -0.175441 2.746891

C 1.912859 -1.085425 1.198913

H 2.410286 -1.751379 1.910837

C 2.93152 -0.752917 0.116199

C 2.569073 -0.468852 -1.204826

C 4.289065 -0.700031 0.446863

C 3.519424 -0.113995 -2.152521

H 1.530838 -0.534921 -1.513663

C 5.25344 -0.342737 -0.489862

H 4.606684 -0.956686 1.454161

C 4.866071 -0.040148 -1.79384

H 3.228705 0.104112 -3.174788

H 6.303172 -0.308963 -0.213062

O 2.644302 0.370439 3.021672

O 1.926633 4.845791 0.986224

O 5.761436 0.318171 -2.760904

O 1.866454 -3.926474 1.114659

O -2.606678 -4.246815 -0.214207

O -7.052643 0.662795 0.556066

O -1.47247 0.113138 -2.315769

H -0.53271 0.108778 -2.542413

H 6.64886 0.333121 -2.382137

H 2.65315 -3.385512 0.981393

H -3.360076 -3.708696 -0.487418

H -7.632349 0.358783 -0.153365

H 3.417337 0.645442 2.511314

VaticahainolB_Conf13.log

Energy (E) = -1680.34925490 Hartree

Enthalpy (H) = -1679.887749 Hartree

Gibbs free energy (G) = -1679.977190 Hartree

Charge = 0, Spin = 1

C 1.32779 3.778653 0.774229

C 0.258232 3.642347 -0.196794

C -0.292024 2.421084 -0.356263

C 0.120413 1.237462 0.378222

C 1.18027 1.366663 1.355519

C 1.737149 2.579088 1.536426

H -0.060538 4.504131 -0.770205

H 2.519201 2.731701 2.271769

C -0.623633 0.188075 -0.036823

C -1.625116 0.712034 -1.083409

O -1.275824 2.121998 -1.218851

C -3.083243 0.663668 -0.665246

C -3.448425 1.067521 0.621758

C -4.074669 0.25942 -1.556243

C -4.777142 1.062195 1.016098

H -2.68919 1.387392 1.329347

C -5.410457 0.248726 -1.170853

H -3.808658 -0.058837 -2.557125

C -5.763696 0.649637 0.117147

H -5.062505 1.371243 2.015784

H -6.179828 -0.071179 -1.867338

C -0.505822 -1.249751 0.245336

C -1.599005 -2.064263 -0.076863

C 0.695941 -1.827499 0.72574

C -1.521164 -3.444622 0.056259

H -2.527866 -1.642042 -0.433331

C 0.758384 -3.230994 0.766933

C -0.333266 -4.036517 0.459107

H -0.236413 -5.115364 0.526254

C 1.578093 0.141322 2.129452

H 0.727373 -0.160217 2.75112

C 1.916781 -1.067274 1.210795

H 2.418055 -1.726875 1.926083

C 2.93388 -0.734344 0.126391

C 4.292421 -0.667619 0.457456

C 2.570906 -0.470176 -1.196925

C 5.25404 -0.31853 -0.482716

H 4.610099 -0.908723 1.468543

C 3.520837 -0.12356 -2.150509

H 1.533609 -0.546043 -1.506805

C 4.866412 -0.039001 -1.792293

H 6.304292 -0.27276 -0.214783

H 3.220766 0.076514 -3.17517

O 2.631734 0.39807 3.033584

O 1.89874 4.861815 0.977878

O 5.842727 0.29691 -2.686305

O 1.886801 -3.909626 1.138148

O -2.631912 -4.169024 -0.253727

O -7.054706 0.656159 0.552527

O -1.472131 0.100995 -2.31645

H -0.532169 0.098324 -2.542278

H 5.443168 0.451296 -3.550974

H 2.671403 -3.36413 1.011158

H -2.447743 -5.109529 -0.138083

H -7.634476 0.353588 -0.157456

H 3.404671 0.680652 2.527475

VaticahainolB_Conf14.log

Energy (E) = -1680.34828178 Hartree

Enthalpy (H) = -1679.886969 Hartree

Gibbs free energy (G) = -1679.977137 Hartree

Charge = 0, Spin = 1

C 1.646739 3.571696 1.016426

C 0.588634 3.552672 0.021107

C -0.045616 2.382932 -0.199896

C 0.256482 1.149512 0.503978

C 1.308955 1.160602 1.497915

C 1.96556 2.312762 1.724691

H 0.349675 4.457522 -0.524132

H 2.772708 2.363537 2.447377

C -0.547319 0.170673 0.032826

C -1.468968 0.795491 -1.02991

O -1.023333 2.184309 -1.098677

C -2.943868 0.830166 -0.675506

C -3.920564 0.474527 -1.604882

C -3.336819 1.253732 0.595233

C -5.26756 0.532453 -1.271425

H -3.632352 0.140273 -2.594267

C -4.679498 1.315964 0.939036

H -2.590598 1.536864 1.331809

C -5.649821 0.952124 0.002715

H -6.02973 0.252755 -1.990665

H -4.979341 1.641983 1.930464

C -0.54122 -1.279768 0.27948

C -1.700666 -2.001219 -0.02465

C 0.626476 -1.954481 0.717024

C -1.723315 -3.386071 0.092409

H -2.603903 -1.503646 -0.348984

C 0.586636 -3.35718 0.740213

C -0.573639 -4.072016 0.454289

H -0.558384 -5.155898 0.506547

C 1.608015 -0.139012 2.188315

H 0.710991 -0.440735 2.746853

C 1.901314 -1.260454 1.156513

H 2.461832 -1.976364 1.765347

C 2.811174 -0.820348 0.011332

C 4.106901 -0.369277 0.292899

C 2.402426 -0.834194 -1.322603

C 4.95668 0.067348 -0.713363

H 4.455191 -0.352459 1.319974

C 3.243244 -0.40145 -2.344719

H 1.412082 -1.192031 -1.58573

C 4.523309 0.055429 -2.039961

H 5.958405 0.415202 -0.483351

H 2.905836 -0.419515 -3.377093

O 2.709787 -0.054154 3.068977

O 2.286198 4.60347 1.275016

O 5.397155 0.494852 -2.995024

O 1.668818 -4.126879 1.068992

O -2.895574 -4.018762 -0.190913

O -6.982725 0.985596 0.282928

O -1.299309 0.213662 -2.275339

H -0.351252 0.143893 -2.45316

H 4.980686 0.430993 -3.862921

H 2.490764 -3.644509 0.924199

H -2.787049 -4.970597 -0.073381

H -7.112924 1.285775 1.190805

H 2.457191 0.51452 3.805718

VaticahainolB_Conf15.log

Energy (E) = -1680.34836194 Hartree

Enthalpy (H) = -1679.887130 Hartree

Gibbs free energy (G) = -1679.977002 Hartree

Charge = 0, Spin = 1

C 1.633388 3.588488 0.968746

C 0.568361 3.558474 -0.018828

C -0.064857 2.3855 -0.22501

C 0.245482 1.158774 0.486881

C 1.301248 1.182073 1.476862

C 1.95763 2.337336 1.688187

H 0.323837 4.457857 -0.570593

H 2.766845 2.397262 2.407778

C -0.555433 0.172645 0.025814

C -1.486711 0.786999 -1.035147

O -1.048146 2.177001 -1.115459

C -2.959483 0.815423 -0.671039

C -3.940322 0.461317 -1.596844

C -3.346658 1.230022 0.60443

C -5.285498 0.511648 -1.255169

H -3.656516 0.134207 -2.589897

C -4.687609 1.284862 0.956352

H -2.597225 1.511253 1.338434

C -5.66195 0.922601 0.023626

H -6.050788 0.233278 -1.971616

H -4.98291 1.603575 1.951504

C -0.539492 -1.276392 0.278516

C -1.692336 -2.007505 -0.036721

C 0.627375 -1.942342 0.726294

C -1.705438 -3.392063 0.076814

H -2.592234 -1.507497 -0.370727

C 0.59578 -3.347263 0.75126

C -0.554771 -4.069309 0.453645

H -0.539313 -5.15213 0.502462

C 1.600861 -0.107972 2.184648

H 0.703402 -0.402942 2.746205

C 1.898127 -1.243252 1.170316

H 2.451684 -1.952479 1.793109

C 2.819425 -0.81941 0.028172

C 2.408718 -0.810601 -1.306955

C 4.125154 -0.405337 0.312059

C 3.258355 -0.390963 -2.324488

H 1.409532 -1.14146 -1.571443

C 4.98649 0.018018 -0.692666

H 4.475632 -0.407075 1.338625

C 4.551001 0.028874 -2.018133

H 2.927091 -0.389078 -3.357664

H 5.997788 0.334808 -0.453424

O 2.702042 -0.009531 3.064545

O 2.273277 4.623532 1.212794

O 5.351835 0.432163 -3.049955

O 1.680928 -4.107494 1.092923

O -2.812969 -4.134224 -0.201121

O -6.993207 0.948926 0.311749

O -1.323229 0.198966 -2.278539

H -0.37634 0.135965 -2.464484

H 6.215659 0.68321 -2.701223

H 2.500611 -3.621215 0.948949

H -3.530897 -3.550514 -0.475903

H -7.119458 1.245468 1.221385

H 2.448333 0.568932 3.793287

VaticahainolB_Conf16.log

Energy (E) = -1680.34839310 Hartree

Enthalpy (H) = -1679.887153 Hartree

Gibbs free energy (G) = -1679.976986 Hartree

Charge = 0, Spin = 1

C 1.648759 3.58238 0.971436

C 0.581407 3.557105 -0.013869

C -0.056677 2.386686 -0.21941

C 0.250966 1.158229 0.490538

C 1.308543 1.176961 1.478632

C 1.969555 2.32963 1.689647

H 0.338944 4.457833 -0.564349

H 2.780379 2.386064 2.407711

C -0.553666 0.175046 0.029705

C -1.485488 0.793685 -1.028432

O -1.043225 2.182494 -1.107397

C -2.957283 0.82547 -0.660817

C -3.341119 1.251571 0.61371

C -3.939612 0.461844 -1.578796

C -4.679293 1.306666 0.969679

H -2.588786 1.541351 1.341417

C -5.285228 0.512359 -1.232172

H -3.659783 0.12611 -2.570098

C -5.657028 0.932893 0.044336

H -4.979357 1.632729 1.959613

H -6.047723 0.224044 -1.949699

C -0.540691 -1.274484 0.280015

C -1.69638 -2.002268 -0.032422

C 0.625849 -1.944088 0.723147

C -1.712425 -3.386996 0.078771

H -2.596336 -1.49937 -0.361794

C 0.591003 -3.348974 0.745801

C -0.562146 -4.067751 0.450389

H -0.54925 -5.150684 0.497464

C 1.60464 -0.115016 2.184278

H 0.706363 -0.408596 2.74524

C 1.898618 -1.249022 1.167348

H 2.450903 -1.960968 1.788236

C 2.820565 -0.824475 0.026248

C 4.127379 -0.410662 0.313374

C 2.412065 -0.812744 -1.307838

C 4.988749 0.015395 -0.687624

H 4.475317 -0.415452 1.340761

C 3.264296 -0.390168 -2.324584

H 1.412982 -1.141987 -1.575106

C 4.555642 0.030062 -2.014266

H 5.99922 0.334009 -0.453605

H 2.927183 -0.387916 -3.357206

O 2.705973 -0.021359 3.064455

O 2.293172 4.61481 1.214573

O 5.440824 0.457661 -2.964172

O 1.675304 -4.11229 1.083121

O -2.822739 -4.126003 -0.196586

O -6.957901 0.99616 0.443685

O -1.326198 0.206921 -2.273018

H -0.379817 0.138403 -2.459667

H 5.022292 0.417341 -3.832513

H 2.495727 -3.627027 0.939773

H -3.540198 -3.539998 -0.467603

H -7.530441 0.712449 -0.27976

H 2.454703 0.558492 3.792934

VaticahainolB_Conf17.log

Energy (E) = -1680.34831073 Hartree

Enthalpy (H) = -1679.887103 Hartree

Gibbs free energy (G) = -1679.976976 Hartree

Charge = 0, Spin = 1

C 1.661637 3.569627 1.025466

C 0.600173 3.555474 0.033724

C -0.037537 2.387731 -0.187829

C 0.263209 1.151922 0.512515

C 1.320637 1.157783 1.501449

C 1.980719 2.307914 1.728501

H 0.361382 4.462119 -0.508591

H 2.791617 2.354591 2.447262

C -0.546198 0.176832 0.043061

C -1.466495 0.805859 -1.01888

O -1.018972 2.193909 -1.08363

C -2.941872 0.841959 -0.667484

C -3.337568 1.276743 0.600402

C -3.915408 0.47536 -1.593396

C -4.67915 1.33702 0.942229

H -2.591738 1.569536 1.333673

C -5.264477 0.5316 -1.261208

H -3.626263 0.132886 -2.579672

C -5.648236 0.960173 0.008992

H -4.988765 1.669742 1.92699

H -6.020097 0.240853 -1.984999

C -0.545147 -1.27378 0.289509

C -1.709235 -1.990572 -0.007619

C 0.622219 -1.953362 0.720247

C -1.736813 -3.375353 0.109657

H -2.612315 -1.489366 -0.326585

C 0.576886 -3.355827 0.743916

C -0.587913 -4.065994 0.465027

H -0.576766 -5.149955 0.516869

C 1.622041 -0.144259 2.186344

H 0.729277 -0.444719 2.752299

C 1.903098 -1.264719 1.150315

H 2.46532 -1.98397 1.753527

C 2.805087 -0.827528 -0.002471

C 2.38568 -0.847217 -1.334663

C 4.102127 -0.377245 0.266251

C 3.217641 -0.420633 -2.364062

H 1.393525 -1.206892 -1.587854

C 4.945767 0.053282 -0.75028

H 4.459694 -0.355438 1.290041

C 4.501405 0.035173 -2.072714

H 2.879246 -0.4413 -3.394729

H 5.950328 0.398681 -0.522384

O 2.732021 -0.064346 3.057225

O 2.304095 4.599366 1.284869

O 5.285314 0.443905 -3.11543

O 1.658391 -4.129538 1.065883

O -2.913222 -4.00328 -0.167091

O -6.953009 1.029184 0.394837

O -1.295471 0.226001 -2.265204

H -0.347247 0.156632 -2.44222

H 6.144099 0.723111 -2.775773

H 2.481238 -3.651499 0.911744

H -2.80782 -4.955568 -0.050378

H -7.518746 0.739756 -0.33164

H 2.487178 0.501411 3.79876

VaticahainolB_Conf18.log

Energy (E) = -1680.34833339 Hartree

Enthalpy (H) = -1679.887236 Hartree

Gibbs free energy (G) = -1679.976909 Hartree

Charge = 0, Spin = 1

C 1.695238 3.558924 1.100875

C 0.646947 3.551517 0.094979

C 0.011497 2.385662 -0.14154

C 0.299532 1.146155 0.557932

C 1.357191 1.140754 1.54736

C 2.012789 2.289894 1.791556

H 0.415094 4.461829 -0.444203

H 2.824791 2.328689 2.509579

C -0.518239 0.179696 0.085522

C -1.412604 0.81219 -0.995954

O -0.960016 2.198366 -1.049684

C -2.896583 0.853933 -0.686145

C -3.845001 0.434338 -1.617895

C -3.32547 1.340758 0.549712

C -5.200519 0.492357 -1.320984

H -3.526961 0.048806 -2.579089

C -4.676902 1.40437 0.856584

H -2.599843 1.673173 1.28676

C -5.619336 0.977211 -0.081917

H -5.941024 0.163401 -2.042028

H -5.005976 1.781682 1.820142

C -0.54031 -1.268674 0.34259

C -1.722136 -1.969909 0.07292

C 0.621924 -1.96133 0.757043

C -1.766127 -3.353041 0.199548

H -2.619988 -1.447908 -0.231368

C 0.557012 -3.364049 0.795843

C -0.621585 -4.057845 0.543695

H -0.633176 -5.140323 0.600433

C 1.672188 -0.172878 2.204852

H 0.793878 -0.478974 2.789859

C 1.919049 -1.2801 1.145216

H 2.503763 -2.00572 1.71859

C 2.772657 -0.835082 -0.042131

C 4.033128 -0.266625 0.181016

C 2.34749 -0.975 -1.363796

C 4.833624 0.157152 -0.870336

H 4.391364 -0.145267 1.197622

C 3.139078 -0.556461 -2.430334

H 1.38055 -1.417531 -1.582061

C 4.385636 0.013396 -2.184235

H 5.807823 0.597391 -0.684609

H 2.78889 -0.673715 -3.45191

O 2.804651 -0.110664 3.048223

O 2.331456 4.587691 1.378376

O 5.209676 0.442943 -3.186902

O 1.635961 -4.147674 1.103763

O -2.901814 -4.067339 -0.034623

O -6.959337 1.009943 0.162095

O -1.208703 0.227185 -2.235375

H -0.255881 0.143076 -2.379116

H 4.788458 0.273509 -4.038321

H 2.460439 -3.686019 0.911936

H -3.610219 -3.465899 -0.295271

H -7.118016 1.364122 1.045726

H 2.580065 0.438215 3.80847

VaticahainolB_Conf19.log

Energy (E) = -1680.34925454 Hartree

Enthalpy (H) = -1679.887681 Hartree

Gibbs free energy (G) = -1679.976855 Hartree

Charge = 0, Spin = 1

C 1.31717 3.772412 0.774365

C 0.249557 3.635544 -0.198694

C -0.298484 2.413569 -0.36061

C 0.114477 1.229977 0.373614

C 1.172374 1.359606 1.352883

C 1.727095 2.572666 1.536063

H -0.069151 4.497371 -0.77208

H 2.507817 2.725598 2.272752

C -0.626752 0.179744 -0.044103

C -1.628468 0.703166 -1.090251

O -1.279495 2.113543 -1.22591

C -3.086323 0.654892 -0.671119

C -4.081402 0.268118 -1.568413

C -3.448661 1.044558 0.619218

C -5.415992 0.264483 -1.184685

H -3.816143 -0.039861 -2.572718

C -4.779194 1.045372 1.013229

H -2.688005 1.349365 1.331863

C -5.767924 0.654536 0.107628

H -6.192281 -0.038308 -1.879077

H -5.054667 1.345123 2.019883

C -0.506527 -1.258239 0.235779

C -1.59816 -2.074086 -0.088262

C 0.696095 -1.83436 0.71599

C -1.517961 -3.454474 0.043406

H -2.527496 -1.652858 -0.444898

C 0.76101 -3.237789 0.755428

C -0.329157 -4.044749 0.446102

H -0.230568 -5.12351 0.512137

C 1.571018 0.133594 2.125427

H 0.7198 -0.170941 2.744933

C 1.91434 -1.0723 1.204722

H 2.415939 -1.732414 1.919277

C 2.932908 -0.732366 0.123985

C 2.571617 -0.457949 -1.199457

C 4.288989 -0.664976 0.45809

C 3.521294 -0.098675 -2.146206

H 1.535017 -0.53553 -1.511319

C 5.252627 -0.303255 -0.477688

H 4.606193 -0.913766 1.467469

C 4.866194 -0.010816 -1.784267

H 3.231389 0.111557 -3.170359

H 6.301235 -0.258434 -0.198186

O 2.622588 0.390835 3.031802

O 1.886158 4.856215 0.980188

O 5.761022 0.350484 -2.750766

O 1.890226 -3.914986 1.127013

O -2.627346 -4.180481 -0.267507

O -7.090309 0.632322 0.434873

O -1.47633 0.092756 -2.323556

H -0.536769 0.092749 -2.551158

H 6.647531 0.373113 -2.370232

H 2.674272 -3.36866 1.000161

H -2.441539 -5.120787 -0.152898

H -7.20171 0.932173 1.345319

H 3.396469 0.673307 2.526992

VaticahainolB_Conf20.log

Energy (E) = -1680.34831811 Hartree

Enthalpy (H) = -1679.887001 Hartree

Gibbs free energy (G) = -1679.976750 Hartree

Charge = 0, Spin = 1

C 1.666064 3.564884 1.031221

C 0.606609 3.552355 0.037224

C -0.031955 2.38545 -0.186174

C 0.266357 1.148956 0.513993

C 1.321777 1.153129 1.50504

C 1.982366 2.302483 1.734391

H 0.369859 4.459613 -0.504971

H 2.79195 2.347922 2.454723

C -0.543179 0.174935 0.042621

C -1.462013 0.805599 -1.019329

O -1.012094 2.193027 -1.083754

C -2.93727 0.844153 -0.667467

C -3.331682 1.281272 0.600041

C -3.911849 0.477185 -1.592116

C -4.672927 1.343643 0.942717

H -2.585156 1.574343 1.332482

C -5.260645 0.535454 -1.259044

H -3.623774 0.132759 -2.578034

C -5.643071 0.966453 0.010736

H -4.981434 1.678246 1.92719

H -6.017115 0.244377 -1.981812

C -0.543976 -1.275825 0.288045

C -1.708934 -1.991198 -0.009001

C 0.622678 -1.956658 0.718465

C -1.737966 -3.375959 0.108365

H -2.611606 -1.488883 -0.327392

C 0.576008 -3.359055 0.741942

C -0.589678 -4.067883 0.46326

H -0.579819 -5.151853 0.515239

C 1.620644 -0.150304 2.188375

H 0.726214 -0.45127 2.751404

C 1.903183 -1.2685 1.150001

H 2.465992 -1.988552 1.751761

C 2.805134 -0.827486 -0.001221

C 4.09857 -0.364936 0.272298

C 2.391577 -0.853515 -1.333469

C 4.941465 0.070513 -0.740212

H 4.450144 -0.337582 1.298012

C 3.225561 -0.422155 -2.361816

H 1.402561 -1.21963 -1.590352

C 4.503443 0.045802 -2.065101

H 5.941356 0.42754 -0.516349

H 2.88446 -0.4498 -3.392766

O 2.728314 -0.073181 3.062398

O 2.309102 4.593783 1.29231

O 5.370511 0.484529 -3.026646

O 1.656661 -4.134003 1.063589

O -2.915239 -4.002607 -0.167679

O -6.947494 1.037539 0.397356

O -1.291808 0.225708 -2.265704

H -0.343589 0.151926 -2.441131

H 4.951213 0.410527 -3.892351

H 2.480019 -3.656217 0.911411

H -2.811096 -4.954914 -0.04999

H -7.51402 0.747766 -0.328362

H 2.482474 0.492562 3.803621

VaticahainolB_Conf21.log

Energy (E) = -1680.34831807 Hartree

Enthalpy (H) = -1679.887002 Hartree

Gibbs free energy (G) = -1679.976744 Hartree

Charge = 0, Spin = 1

C 1.666228 3.564876 1.031115

C 0.606776 3.552343 0.037105

C -0.031845 2.385455 -0.18621

C 0.2664 1.148989 0.514004

C 1.321819 1.153175 1.505044

C 1.982449 2.302517 1.734364

H 0.370057 4.459573 -0.505148

H 2.792023 2.347915 2.454709

C -0.543196 0.174985 0.042695

C -1.461968 0.805691 -1.019328

O -1.012043 2.193045 -1.08374

C -2.937254 0.844239 -0.667554

C -3.331718 1.281945 0.599736

C -3.911788 0.476648 -1.592003

C -4.672966 1.344279 0.942401

H -2.585224 1.575548 1.332001

C -5.260591 0.534887 -1.258944

H -3.623665 0.131749 -2.577743

C -5.643062 0.966468 0.010622

H -4.981514 1.679358 1.9267

H -6.017031 0.243331 -1.981551

C -0.54404 -1.275761 0.288187

C -1.709019 -1.991118 -0.008792

C 0.622622 -1.956615 0.718574

C -1.738064 -3.375884 0.10857

H -2.611706 -1.488794 -0.327124

C 0.575949 -3.35901 0.742026

C -0.589768 -4.067823 0.463401

H -0.579916 -5.151793 0.515366

C 1.620643 -0.150242 2.188428

H 0.726198 -0.451176 2.75145

C 1.903143 -1.26847 1.150063

H 2.465963 -1.988497 1.751849

C 2.805079 -0.827532 -0.0012

C 4.098509 -0.364926 0.272274

C 2.391534 -0.853704 -1.333446

C 4.941403 0.070446 -0.740273

H 4.450101 -0.337472 1.29798

C 3.225526 -0.422436 -2.361833

H 1.402551 -1.219899 -1.590354

C 4.50339 0.045589 -2.065161

H 5.941282 0.427522 -0.516433

H 2.884442 -0.450235 -3.392785

O 2.728295 -0.073117 3.062476

O 2.309309 4.593778 1.292141

O 5.370453 0.484207 -3.026758

O 1.656617 -4.13398 1.063505

O -2.915357 -4.002514 -0.167415

O -6.947491 1.037564 0.397211

O -1.291698 0.22574 -2.265698

H -0.343462 0.151923 -2.44104

H 4.951067 0.410293 -3.892429

H 2.479955 -3.656042 0.911682

H -2.811252 -4.954814 -0.049629

H -7.513978 0.747366 -0.32837

H 2.48229 0.492283 3.80391

VaticahainolB_Conf22.log

Energy (E) = -1680.34834453 Hartree

Enthalpy (H) = -1679.887140 Hartree

Gibbs free energy (G) = -1679.976685 Hartree

Charge = 0, Spin = 1

C 1.699726 3.554176 1.101963

C 0.649653 3.550041 0.097988

C 0.012126 2.385475 -0.139439

C 0.299469 1.144322 0.557432

C 1.358376 1.135897 1.545537

C 2.01626 2.283627 1.790246

H 0.418232 4.461543 -0.439364

H 2.829175 2.320142 2.507355

C -0.520064 0.179726 0.084185

C -1.41499 0.815457 -0.995177

O -0.961038 2.201254 -1.046376

C -2.898686 0.857916 -0.68411

C -3.848061 0.442786 -1.616913

C -3.326364 1.340967 0.553618

C -5.203347 0.5013 -1.319152

H -3.530949 0.060334 -2.579632

C -4.677597 1.40504 0.861361

H -2.599902 1.670012 1.291356

C -5.620978 0.982195 -0.078148

H -5.944562 0.175759 -2.041015

H -5.005784 1.779362 1.826383

C -0.543558 -1.269095 0.33881

C -1.725992 -1.968506 0.067074

C 0.617706 -1.964001 0.752556

C -1.771823 -3.351808 0.191029

H -2.622956 -1.444891 -0.237003

C 0.550802 -3.366714 0.789132

C -0.628491 -4.058608 0.534965

H -0.641416 -5.14117 0.589783

C 1.671967 -0.178698 2.201765

H 0.793865 -0.483798 2.787619

C 1.916248 -1.285722 1.141471

H 2.499131 -2.013025 1.714459

C 2.770908 -0.841252 -0.045408

C 2.339093 -0.967466 -1.367959

C 4.035819 -0.286503 0.176724

C 3.128403 -0.547026 -2.433055

H 1.367782 -1.401034 -1.584647

C 4.836581 0.139338 -0.875907

H 4.400412 -0.175763 1.192288

C 4.381254 0.010795 -2.188506

H 2.779733 -0.65003 -3.455357

H 5.815618 0.56897 -0.682978

O 2.805614 -0.118566 3.043713

O 2.338242 4.581432 1.379929

O 5.122515 0.409383 -3.265928

O 1.628348 -4.152446 1.096719

O -2.908183 -4.064202 -0.045642

O -6.960821 1.015427 0.16668

O -1.213191 0.233131 -2.236174

H -0.260642 0.151877 -2.38309

H 5.961487 0.773401 -2.958555

H 2.45377 -3.69196 0.90618

H -3.615478 -3.461348 -0.306063

H -7.118667 1.366444 1.051712

H 2.582625 0.429909 3.804719

VaticahainolB_Conf23.log

Energy (E) = -1680.34838663 Hartree

Enthalpy (H) = -1679.887152 Hartree

Gibbs free energy (G) = -1679.976623 Hartree

Charge = 0, Spin = 1

C 1.649905 3.583844 0.976064

C 0.582037 3.559087 -0.008601

C -0.055662 2.388608 -0.21497

C 0.252229 1.159553 0.494004

C 1.311467 1.177465 1.48051

C 1.972252 2.330189 1.691967

H 0.3389 4.460236 -0.558098

H 2.784304 2.385861 2.408705

C -0.553599 0.1771 0.033572

C -1.484025 0.796113 -1.025744

O -1.042451 2.185131 -1.102807

C -2.956693 0.826997 -0.661926

C -3.344032 1.253576 0.611349

C -3.936351 0.462005 -1.582202

C -4.683162 1.307547 0.963908

H -2.593631 1.544553 1.340598

C -5.282876 0.511428 -1.239028

H -3.65375 0.12598 -2.572605

C -5.658233 0.932235 0.036345

H -4.986078 1.633932 1.952864

H -6.043234 0.22201 -1.958378

C -0.54215 -1.272349 0.284878

C -1.699034 -1.99917 -0.025313

C 0.624299 -1.942937 0.726701

C -1.716334 -3.383813 0.086954

H -2.598885 -1.495708 -0.354069

C 0.588007 -3.347717 0.750722

C -0.566342 -4.065523 0.457755

H -0.554492 -5.14844 0.505405

C 1.610496 -0.115075 2.183998

H 0.714911 -0.408698 2.749192

C 1.899443 -1.248871 1.165644

H 2.453621 -1.961462 1.783998

C 2.81629 -0.825749 0.019625

C 2.400057 -0.817764 -1.313766

C 4.123509 -0.412376 0.297641

C 3.24561 -0.399572 -2.335309

H 1.399519 -1.148186 -1.573556

C 4.980825 0.009518 -0.711105

H 4.478391 -0.41345 1.322668

C 4.53981 0.019605 -2.03475

H 2.90993 -0.398334 -3.367062

H 5.993349 0.325836 -0.476434

O 2.716205 -0.022121 3.058865

O 2.293851 4.616307 1.220247

O 5.336722 0.421617 -3.070102

O 1.672357 -4.111713 1.086742

O -2.827688 -4.121907 -0.186686

O -6.96015 0.994365 0.432521

O -1.321594 0.210046 -2.270329

H -0.374838 0.1455 -2.456454

H 6.201902 0.672788 -2.724824

H 2.493028 -3.628762 0.936993

H -3.54447 -3.535487 -0.458572

H -7.530666 0.709456 -0.292048

H 2.467715 0.554858 3.790546

VaticahainolB_Conf24.log

Energy (E) = -1680.34846496 Hartree

Enthalpy (H) = -1679.886929 Hartree

Gibbs free energy (G) = -1679.976408 Hartree

Charge = 0, Spin = 1

C 1.610465 3.594422 1.030679

C 0.566297 3.561126 0.021956

C -0.050389 2.383226 -0.20389

C 0.260499 1.152283 0.501467

C 1.306311 1.176193 1.503055

C 1.93671 2.340488 1.744343

H 0.31905 4.462798 -0.52482

H 2.725956 2.404212 2.483941

C -0.54264 0.170554 0.035302

C -1.463246 0.78722 -1.033119

O -1.021928 2.175754 -1.107524

C -2.939535 0.818583 -0.683159

C -3.911677 0.438821 -1.607817

C -3.338188 1.259995 0.579708

C -5.259653 0.490168 -1.277268

H -3.618942 0.090268 -2.590901

C -4.681921 1.316418 0.920352

H -2.595715 1.562223 1.312544

C -5.647612 0.928277 -0.011047

H -6.018147 0.191735 -1.992857

H -4.986307 1.656966 1.905498

C -0.538023 -1.277077 0.294142

C -1.699758 -2.002342 -0.000893

C 0.630031 -1.946296 0.730637

C -1.719593 -3.385923 0.123734

H -2.601043 -1.499065 -0.3261

C 0.590385 -3.350754 0.766903

C -0.56816 -4.067909 0.490644

H -0.558794 -5.15047 0.546271

C 1.630229 -0.114624 2.188028

H 0.750941 -0.423101 2.771801

C 1.907407 -1.248851 1.154346

H 2.468899 -1.968772 1.75863

C 2.810644 -0.819568 0.001336

C 2.392314 -0.842722 -1.331373

C 4.108299 -0.370787 0.269748

C 3.225688 -0.420734 -2.361354

H 1.40021 -1.202675 -1.584178

C 4.953159 0.055852 -0.747659

H 4.462859 -0.345728 1.294365

C 4.509916 0.034268 -2.070308

H 2.888333 -0.44474 -3.392308

H 5.958243 0.400068 -0.520187

O 2.729301 0.091791 3.049257

O 2.232396 4.634695 1.298199

O 5.295456 0.438003 -3.113935

O 1.677936 -4.113101 1.098727

O -2.835021 -4.123412 -0.134321

O -6.981146 0.954766 0.26647

O -1.288957 0.198404 -2.274793

H -0.340414 0.137527 -2.453097

H 6.15434 0.717017 -2.774404

H 2.496976 -3.639893 0.912345

H -3.551421 -3.53757 -0.408388

H -7.116081 1.273881 1.167184

H 2.829 -0.701722 3.587052

VaticahainolB_Conf25.log

Energy (E) = -1680.34847200 Hartree

Enthalpy (H) = -1679.886806 Hartree

Gibbs free energy (G) = -1679.976029 Hartree

Charge = 0, Spin = 1

C 1.612033 3.591517 1.029673

C 0.568533 3.558813 0.020151

C -0.049074 2.381427 -0.205766

C 0.260702 1.150426 0.499922

C 1.30488 1.173948 1.503116

C 1.936124 2.337721 1.744646

H 0.322577 4.460589 -0.527044

H 2.724384 2.401148 2.485325

C -0.541744 0.168742 0.032556

C -1.462815 0.78604 -1.034839

O -1.020173 2.174305 -1.109998

C -2.938468 0.81875 -0.68207

C -3.912882 0.441473 -1.605351

C -3.334208 1.258752 0.582235

C -5.260157 0.493953 -1.27205

H -3.622509 0.093929 -2.589507

C -4.677181 1.316327 0.925611

H -2.590115 1.558909 1.314246

C -5.645143 0.93068 -0.004439

H -6.020436 0.197385 -1.986519

H -4.979153 1.655684 1.911912

C -0.536786 -1.279102 0.289998

C -1.697979 -2.004476 -0.006983

C 0.630969 -1.948238 0.727355

C -1.717569 -3.388127 0.116726

H -2.599068 -1.501176 -0.332721

C 0.591734 -3.352766 0.762442

C -0.566288 -4.070039 0.484266

H -0.556756 -5.152625 0.539395

C 1.625803 -0.11707 2.189048

H 0.744049 -0.425693 2.769008

C 1.906772 -1.250573 1.155268

H 2.467092 -1.970637 1.760507

C 2.813459 -0.818947 0.006086

C 4.110428 -0.367807 0.282014

C 2.401437 -0.839421 -1.326984

C 4.957639 0.063717 -0.728835

H 4.459246 -0.345482 1.308638

C 3.239846 -0.412511 -2.353377

H 1.410709 -1.199233 -1.585796

C 4.520887 0.045478 -2.054133

H 5.960335 0.411758 -0.503284

H 2.900087 -0.436312 -3.384885

O 2.72122 0.088924 3.054926

O 2.234913 4.631253 1.296924

O 5.392424 0.479232 -3.014101

O 1.67889 -4.115146 1.09527

O -2.832574 -4.125773 -0.142755

O -6.978082 0.958305 0.275767

O -1.291009 0.19721 -2.276786

H -0.342721 0.133117 -2.455372

H 4.972893 0.411357 -3.880193

H 2.4982 -3.641105 0.912286

H -3.549203 -3.539859 -0.41607

H -7.110764 1.275509 1.177489

H 2.820591 -0.705866 3.590901

VaticahainolB_Conf26.log

Energy (E) = -1680.34836727 Hartree

Enthalpy (H) = -1679.886747 Hartree

Gibbs free energy (G) = -1679.976014 Hartree

Charge = 0, Spin = 1

C 1.611226 3.584914 1.061592

C 0.570326 3.559025 0.049301

C -0.045291 2.382678 -0.18759

C 0.263305 1.146675 0.509942

C 1.307829 1.1626 1.513185

C 1.936917 2.325264 1.765389

H 0.324521 4.464853 -0.491222

H 2.725148 2.383095 2.506566

C -0.540529 0.16918 0.036455

C -1.457579 0.792904 -1.030514

O -1.013601 2.181575 -1.095992

C -2.934691 0.826229 -0.684714

C -3.905189 0.458011 -1.615615

C -3.335772 1.260843 0.57971

C -5.254152 0.514388 -1.289875

H -3.610623 0.114957 -2.600071

C -4.68047 1.321767 0.915738

H -2.594505 1.554046 1.317421

C -5.64462 0.94537 -0.022059

H -6.011434 0.224901 -2.010404

H -4.986802 1.656887 1.902138

C -0.539477 -1.279802 0.288969

C -1.70381 -1.999553 5.2e-05

C 0.630471 -1.953847 0.718644

C -1.728972 -3.383477 0.127056

H -2.608564 -1.501589 -0.319353

C 0.58704 -3.356363 0.751898

C -0.577524 -4.070243 0.482862

H -0.564454 -5.15385 0.541116

C 1.633163 -0.13409 2.186444

H 0.754697 -0.448344 2.768298

C 1.909492 -1.259055 1.141919

H 2.474548 -1.982872 1.738181

C 2.807694 -0.818472 -0.01068

C 2.387969 -0.841788 -1.342884

C 4.101885 -0.359493 0.257242

C 3.216325 -0.409883 -2.372836

H 1.398541 -1.209566 -1.595123

C 4.941813 0.076946 -0.760083

H 4.45757 -0.334222 1.281441

C 4.496938 0.055424 -2.082181

H 2.877792 -0.433826 -3.403406

H 5.944193 0.429182 -0.532941

O 2.732694 0.065286 3.048843

O 2.231411 4.623479 1.339734

O 5.277484 0.468936 -3.125801

O 1.67274 -4.12496 1.074273

O -2.905396 -4.014895 -0.141427

O -6.979169 0.977216 0.250602

O -1.281004 0.210612 -2.27483

H -0.332112 0.148206 -2.450652

H 6.133901 0.755921 -2.786684

H 2.492932 -3.652179 0.891718

H -2.797977 -4.966381 -0.020329

H -7.115555 1.287563 1.154147

H 2.830997 -0.731691 3.581751

VaticahainolB_Conf27.log

Energy (E) = -1680.34840467 Hartree

Enthalpy (H) = -1679.886813 Hartree

Gibbs free energy (G) = -1679.975969 Hartree

Charge = 0, Spin = 1

C 1.625423 3.581694 1.061994

C 0.58102 3.560033 0.053202

C -0.039191 2.385887 -0.182608

C 0.267918 1.148218 0.51261

C 1.315374 1.160108 1.512841

C 1.949075 2.320499 1.763942

H 0.336259 4.467139 -0.485649

H 2.739639 2.375173 2.502874

C -0.539975 0.17345 0.040377

C -1.458074 0.800934 -1.023679

O -1.011484 2.188765 -1.087638

C -2.934306 0.83647 -0.674917

C -3.332585 1.273193 0.591517

C -3.905938 0.467202 -1.601721

C -4.674777 1.33271 0.931004

H -2.588525 1.567936 1.325813

C -5.255651 0.522615 -1.271852

H -3.614864 0.123169 -2.586879

C -5.641956 0.95309 -0.003084

H -4.986306 1.666934 1.914653

H -6.009759 0.229699 -1.996349

C -0.542023 -1.275889 0.291304

C -1.709007 -1.992272 0.004711

C 0.62717 -1.953602 0.717346

C -1.737565 -3.37627 0.130211

H -2.613274 -1.491478 -0.311547

C 0.580074 -3.35604 0.749139

C -0.587058 -4.066518 0.482208

H -0.576779 -5.150229 0.539195

C 1.638196 -0.137975 2.184515

H 0.759958 -0.450032 2.767867

C 1.909063 -1.262973 1.138628

H 2.473003 -1.988896 1.733394

C 2.806451 -0.824353 -0.015305

C 2.384292 -0.845526 -1.346774

C 4.102506 -0.369477 0.250636

C 3.212153 -0.415427 -2.377897

H 1.393335 -1.210212 -1.597553

C 4.941938 0.065125 -0.767862

H 4.460028 -0.345923 1.274238

C 4.494671 0.045827 -2.089194

H 2.871759 -0.43768 -3.407892

H 5.945779 0.414197 -0.542285

O 2.74001 0.05748 3.044868

O 2.250102 4.617945 1.338716

O 5.274639 0.457763 -3.133867

O 1.664451 -4.128007 1.067757

O -2.916432 -4.004202 -0.135884

O -6.947408 1.021374 0.380668

O -1.284905 0.220243 -2.269278

H -0.336443 0.155314 -2.446485

H 6.132545 0.741759 -2.795991

H 2.485589 -3.656625 0.885788

H -2.811491 -4.956043 -0.015377

H -7.511782 0.730193 -0.34616

H 2.83621 -0.739694 3.577854

VaticahainolB_Conf28.log

Energy (E) = -1680.34786393 Hartree

Enthalpy (H) = -1679.886386 Hartree

Gibbs free energy (G) = -1679.975645 Hartree

Charge = 0, Spin = 1

C 1.324491 3.786222 0.723852

C 0.245708 3.641957 -0.235614

C -0.313051 2.421877 -0.369816

C 0.0973 1.245269 0.374901

C 1.167495 1.383013 1.340283

C 1.733481 2.594671 1.49769

H -0.074469 4.496517 -0.818928

H 2.526363 2.750133 2.220633

C -0.642634 0.189276 -0.033971

C -1.643879 0.709492 -1.080104

O -1.315773 2.118478 -1.210468

C -3.111122 0.655167 -0.674058

C -4.063969 0.014527 -1.465276

C -3.519696 1.270297 0.511359

C -5.400942 -0.008317 -1.088905

H -3.769205 -0.491429 -2.378356

C -4.85111 1.251805 0.899589

H -2.794085 1.770543 1.14611

C -5.797436 0.611959 0.095056

H -6.141972 -0.507266 -1.703881

H -5.162755 1.731452 1.822642

C -0.508431 -1.246572 0.252034

C -1.595464 -2.073483 -0.057631

C 0.702582 -1.812433 0.724132

C -1.502782 -3.452981 0.072489

H -2.533635 -1.661638 -0.399161

C 0.779555 -3.215633 0.762732

C -0.304913 -4.033178 0.461774

H -0.195566 -5.110981 0.526316

C 1.576653 0.167355 2.123521

H 0.733475 -0.132591 2.75604

C 1.917696 -1.046528 1.215108

H 2.410416 -1.703676 1.938599

C 2.948375 -0.721232 0.142065

C 4.30766 -0.728691 0.475305

C 2.598272 -0.389543 -1.169991

C 5.2864 -0.394497 -0.452965

H 4.609926 -1.01473 1.479595

C 3.565073 -0.055667 -2.11061

H 1.558525 -0.394882 -1.47972

C 4.913551 -0.052561 -1.751621

H 6.337331 -0.40753 -0.184171

H 3.276426 0.198479 -3.126498

O 2.63933 0.43773 3.013115

O 1.902729 4.869149 0.907671

O 5.905564 0.264572 -2.635076

O 1.917172 -3.882542 1.12749

O -2.609866 -4.187346 -0.226908

O -7.118302 0.561097 0.423684

O -1.354864 0.073497 -2.280306

H -1.911578 0.452203 -2.974466

H 5.514721 0.464766 -3.494268

H 2.69609 -3.330535 0.993688

H -2.41523 -5.126218 -0.115671

H -7.259866 1.024274 1.258425

H 3.413987 0.686612 2.491487

VaticahainolB_Conf29.log

Energy (E) = -1680.34840755 Hartree

Enthalpy (H) = -1679.886700 Hartree

Gibbs free energy (G) = -1679.975632 Hartree

Charge = 0, Spin = 1

C 1.626162 3.579679 1.060675

C 0.582519 3.558206 0.051007

C -0.038272 2.384367 -0.184719

C 0.267943 1.146818 0.51104

C 1.313754 1.158729 1.512881

C 1.947968 2.318795 1.764094

H 0.338787 4.465245 -0.488429

H 2.73751 2.373464 2.504128

C -0.539117 0.171894 0.037787

C -1.457647 0.79968 -1.02551

O -1.010107 2.18727 -1.090322

C -2.93332 0.836146 -0.674221

C -3.32904 1.272657 0.593118

C -3.906941 0.467751 -1.599284

C -4.670535 1.332982 0.935162

H -2.583576 1.566615 1.326273

C -5.255998 0.523902 -1.26681

H -3.617952 0.123789 -2.585088

C -5.639691 0.954268 0.002793

H -4.979939 1.667067 1.919532

H -6.011688 0.231637 -1.989916

C -0.54067 -1.277581 0.287692

C -1.707024 -1.994366 -0.000538

C 0.628355 -1.954909 0.71471

C -1.735082 -3.378403 0.124404

H -2.611264 -1.493796 -0.317255

C 0.58191 -3.357404 0.745791

C -0.58458 -4.068291 0.47719

H -0.573925 -5.152012 0.533938

C 1.633994 -0.139368 2.185669

H 0.753502 -0.451608 2.765514

C 1.908621 -1.263771 1.139852

H 2.471617 -1.989654 1.735606

C 2.809225 -0.823049 -0.010529

C 4.104726 -0.365985 0.262571

C 2.393158 -0.841927 -1.342287

C 4.946564 0.072615 -0.749688

H 4.456623 -0.344744 1.288142

C 3.226143 -0.407853 -2.370165

H 1.403415 -1.206244 -1.598767

C 4.505774 0.055785 -2.073667

H 5.948125 0.425265 -0.526248

H 2.883275 -0.430425 -3.400671

O 2.732395 0.055975 3.050327

O 2.251462 4.615614 1.337045

O 5.372191 0.496514 -3.035146

O 1.666143 -4.128999 1.065711

O -2.913433 -4.006784 -0.142925

O -6.944378 1.023217 0.388978

O -1.286467 0.21881 -2.271286

H -0.338205 0.151367 -2.448769

H 4.950775 0.427836 -3.900254

H 2.487372 -3.65691 0.886035

H -2.80842 -4.958514 -0.021626

H -7.510265 0.732797 -0.336978

H 2.828415 -0.742324 3.581664

epi-vaticahainolB_Conf1.log

Energy (E) = -1680.35529575 Hartree

Enthalpy (H) = -1679.894054 Hartree

Gibbs free energy (G) = -1679.983750 Hartree

Charge = 0, Spin = 1

C 3.922848 1.037247 1.88998

C 2.709407 1.474296 2.554145

C 1.561282 0.844964 2.232934

C 1.456584 -0.235549 1.264931

C 2.655304 -0.63232 0.549008

C 3.81593 -0.029719 0.873006

H 2.752002 2.270772 3.286916

H 4.734356 -0.284224 0.35784

C 0.176564 -0.672078 1.251584

C -0.631194 0.208591 2.222138

O 0.358616 1.121534 2.7639

C -1.6914 1.017975 1.501384

C -3.049965 0.760494 1.681715

C -1.301483 2.016536 0.608115

C -4.005568 1.496282 0.99309

H -3.368832 -0.017815 2.364998

C -2.247425 2.761154 -0.08191

H -0.24806 2.21673 0.432922

C -3.605294 2.499896 0.110382

H -5.063323 1.300413 1.132257

H -1.935546 3.530818 -0.78163

C -0.448955 -1.745864 0.468625

C -1.601887 -2.354682 0.984373

C 0.080396 -2.148971 -0.773739

C -2.230807 -3.369774 0.275167

H -2.000525 -2.053756 1.944519

C -0.585677 -3.176198 -1.461647

C -1.726872 -3.786389 -0.95079

H -2.224918 -4.576787 -1.503527

C 2.546867 -1.663604 -0.541619

H 2.452459 -2.647118 -0.057839

C 1.283541 -1.493116 -1.423804

H 1.510582 -2.0904 -2.310913

C 0.976286 -0.075182 -1.910067

C -0.324389 0.259121 -2.302985

C 1.960544 0.909033 -2.033179

C -0.644728 1.527891 -2.766622

H -1.115598 -0.480705 -2.233718

C 1.651246 2.193337 -2.472834

H 2.990139 0.683832 -1.782542

C 0.341731 2.509387 -2.828869

H -1.662053 1.773425 -3.052366

H 2.428227 2.949698 -2.540008

O 3.735621 -1.625863 -1.308236

O 5.028902 1.542899 2.13889

O -0.028455 3.760545 -3.242903

O -0.083181 -3.554587 -2.671509

O -3.349973 -3.995534 0.736417

O -4.582318 3.191868 -0.541918

O -1.206232 -0.467768 3.28766

H -0.551921 -1.076475 3.656071

H 0.741881 4.3413 -3.221687

H -0.62103 -4.266168 -3.039229

H -3.60699 -3.611572 1.583896

H -4.179513 3.851318 -1.119882

H 3.727495 -2.39458 -1.889567

epi-vaticahainolB_Conf2.log

Energy (E) = -1680.35501855 Hartree

Enthalpy (H) = -1679.893876 Hartree

Gibbs free energy (G) = -1679.982858 Hartree

Charge = 0, Spin = 1

C 3.610131 0.951981 2.308646

C 2.361517 1.540657 2.759601

C 1.214336 0.952173 2.365796

C 1.148182 -0.222228 1.512751

C 2.391112 -0.807613 1.046921

C 3.548364 -0.240692 1.436854

H 2.378839 2.418837 3.393325

H 4.501042 -0.638621 1.108305

C -0.151064 -0.546454 1.328675

C -1.0046 0.471332 2.110333

O -0.02126 1.369065 2.691452

C -1.920675 1.280711 1.214307

C -1.365002 2.172648 0.293836

C -3.304244 1.126502 1.256936

C -2.172609 2.89983 -0.566813

H -0.288308 2.300634 0.240209

C -4.124541 1.85039 0.398633

H -3.75355 0.43843 1.963123

C -3.558838 2.737126 -0.516786

H -1.741067 3.592025 -1.281762

H -5.202732 1.727118 0.437117

C -0.763149 -1.602065 0.510281

C -2.056084 -2.023841 0.843594

C -0.099557 -2.139871 -0.614701

C -2.707348 -2.968615 0.061433

H -2.559023 -1.634819 1.717865

C -0.802472 -3.069677 -1.395637

C -2.089601 -3.48965 -1.068124

H -2.601318 -4.217789 -1.691706

C 2.307385 -1.999872 0.140347

H 1.909181 -2.839099 0.729652

C 1.309575 -1.767933 -1.030685

H 1.607967 -2.512972 -1.773072

C 1.420739 -0.401666 -1.706749

C 0.293178 0.333213 -2.078226

C 2.676128 0.136238 -2.014496

C 0.403758 1.561748 -2.722166

H -0.69899 -0.047784 -1.858739

C 2.803948 1.366168 -2.648058

H 3.572837 -0.41291 -1.750307

C 1.662557 2.085022 -3.003405

H -0.483117 2.122372 -2.998709

H 3.787996 1.769483 -2.870756

O 3.608162 -2.317892 -0.312087

O 4.712325 1.423543 2.630089

O 1.727182 3.300565 -3.628209

O -0.182664 -3.557035 -2.508092

O -3.95962 -3.345139 0.443344

O -4.310889 3.469572 -1.38607

O -1.739879 -0.084699 3.147969

H -1.179053 -0.713817 3.621479

H 2.653248 3.539274 -3.755205

H -0.760331 -4.190137 -2.951177

H -4.301306 -3.999826 -0.17771

H -5.244328 3.262013 -1.255509

H 3.548386 -3.13963 -0.812192

epi-vaticahainolB_Conf3.log

Energy (E) = -1680.35524109 Hartree

Enthalpy (H) = -1679.893935 Hartree

Gibbs free energy (G) = -1679.982601 Hartree

Charge = 0, Spin = 1

C 3.591504 0.960756 2.330219

C 2.337137 1.541129 2.776177

C 1.195472 0.947908 2.373718

C 1.140557 -0.223683 1.516173

C 2.389146 -0.801113 1.055894

C 3.541143 -0.229489 1.454383

H 2.346232 2.41714 3.413059

H 4.497816 -0.62161 1.130495

C -0.155909 -0.553985 1.323133

C -1.01916 0.455837 2.104426

O -0.044345 1.356903 2.693924

C -1.936152 1.2632 1.207606

C -1.381176 2.158986 0.290446

C -3.319295 1.104472 1.246594

C -2.189018 2.885263 -0.57068

H -0.304792 2.290729 0.239868

C -4.139902 1.827511 0.387852

H -3.76812 0.413604 1.950407

C -3.574869 2.718034 -0.524304

H -1.758002 3.580419 -1.283074

H -5.21778 1.700773 0.423555

C -0.757763 -1.608682 0.496412

C -2.053246 -2.037363 0.819516

C -0.085564 -2.138083 -0.625371

C -2.69558 -2.979847 0.02769

H -2.556238 -1.650695 1.696558

C -0.780855 -3.067305 -1.416873

C -2.067008 -3.491988 -1.100793

H -2.578537 -4.216796 -1.726314

C 2.316301 -1.991174 0.145583

H 1.917916 -2.8337 0.730023

C 1.324919 -1.759853 -1.031005

H 1.630666 -2.501799 -1.773505

C 1.435324 -0.391349 -1.702666

C 0.307715 0.342708 -2.075598

C 2.690806 0.149804 -2.004243

C 0.418325 1.573692 -2.714887

H -0.684555 -0.040781 -1.860991

C 2.818599 1.382031 -2.633283

H 3.587541 -0.398619 -1.738642

C 1.677166 2.100126 -2.990019

H -0.468607 2.133636 -2.992653

H 3.802684 1.787816 -2.851257

O 3.621203 -2.302447 -0.299524

O 4.689276 1.437228 2.65935

O 1.741942 3.317915 -3.61037

O -0.150302 -3.545726 -2.52725

O -3.949352 -3.435276 0.305243

O -4.327095 3.450035 -1.393691

O -1.755926 -0.109628 3.136223

H -1.191649 -0.733763 3.612265

H 2.668075 3.558654 -3.733091

H -0.722142 -4.179185 -2.977461

H -4.284206 -2.99713 1.097312

H -5.260217 3.239368 -1.265867

H 3.569022 -3.125361 -0.798533

epi-vaticahainolB_Conf4.log

Energy (E) = -1680.35498447 Hartree

Enthalpy (H) = -1679.893382 Hartree

Gibbs free energy (G) = -1679.982236 Hartree

Charge = 0, Spin = 1

C 3.232288 1.550216 2.45851

C 1.926726 2.13831 2.695935

C 0.843974 1.426542 2.323435

C 0.899984 0.120948 1.689087

C 2.199251 -0.477284 1.453945

C 3.293026 0.21309 1.829776

H 1.851742 3.115243 3.157437

H 4.288159 -0.193897 1.688941

C -0.361687 -0.289255 1.433015

C -1.321169 0.818507 1.92298

O -0.430306 1.827365 2.466448

C -2.117197 1.451286 0.795977

C -3.479654 1.200171 0.63553

C -1.46942 2.275888 -0.125793

C -4.182299 1.753594 -0.427074

H -4.004456 0.570804 1.344343

C -2.160227 2.832513 -1.193146

H -0.41001 2.488328 -0.021371

C -3.522686 2.567467 -1.347944

H -5.242204 1.559491 -0.551289

H -1.648244 3.47396 -1.904506

C -0.852026 -1.487065 0.734576

C -2.174268 -1.880765 0.978939

C -0.058445 -2.18646 -0.204413

C -2.733415 -2.943487 0.282871

H -2.773403 -1.376255 1.723349

C -0.674642 -3.230779 -0.914319

C -1.992526 -3.614326 -0.681137

H -2.430617 -4.434249 -1.243962

C 2.256131 -1.836109 0.808969

H 1.829113 -2.560743 1.511672

C 1.407792 -1.925528 -0.493234

H 1.784947 -2.840815 -0.953384

C 1.682141 -0.796817 -1.479195

C 2.893801 -0.768812 -2.181286

C 0.777237 0.239056 -1.713369

C 3.211167 0.271658 -3.045521

H 3.599862 -1.587693 -2.07337

C 1.074555 1.286511 -2.578298

H -0.191845 0.235527 -1.227925

C 2.301719 1.311261 -3.23851

H 4.15328 0.279849 -3.583544

H 0.351534 2.08136 -2.742265

O 3.58382 -2.269176 0.594539

O 4.283117 2.133885 2.767178

O 2.655019 2.31597 -4.095146

O 0.068685 -3.872639 -1.859799

O -4.020067 -3.280025 0.577236

O -4.256189 3.079495 -2.376046

O -2.184874 0.42196 2.933187

H -1.690223 -0.107596 3.572924

H 1.935964 2.957524 -4.144222

H -0.443997 -4.595027 -2.242318

H -4.29059 -4.027545 0.030281

H -3.689216 3.61517 -2.944472

H 4.025785 -1.630102 0.021097

epi-vaticahainolB_Conf5.log

Energy (E) = -1680.35537654 Hartree

Enthalpy (H) = -1679.893866 Hartree

Gibbs free energy (G) = -1679.982160 Hartree

Charge = 0, Spin = 1

C 3.606372 0.966401 2.311796

C 2.351577 1.534689 2.771934

C 1.210892 0.936949 2.373497

C 1.157157 -0.228809 1.507929

C 2.406072 -0.793667 1.033008

C 3.557213 -0.216999 1.426671

H 2.359728 2.40604 3.415185

H 4.513962 -0.599654 1.091896

C -0.138496 -0.566521 1.322498

C -1.002565 0.433924 2.115116

O -0.029152 1.335647 2.705644

C -1.927501 1.242793 1.227614

C -1.381583 2.15141 0.317699

C -3.309377 1.073556 1.268563

C -2.197161 2.88076 -0.53351

H -0.306267 2.290943 0.264934

C -4.137586 1.799209 0.419404

H -3.751398 0.37241 1.966478

C -3.581618 2.703341 -0.484856

H -1.77317 3.586064 -1.240131

H -5.214373 1.664034 0.456661

C -0.739786 -1.619821 0.493398

C -2.029687 -2.059737 0.823775

C -0.072949 -2.136875 -0.637294

C -2.671799 -3.001077 0.030386

H -2.528514 -1.682413 1.707244

C -0.767842 -3.065756 -1.42954

C -2.048511 -3.501405 -1.106286

H -2.560046 -4.225291 -1.732874

C 2.334209 -1.976992 0.113926

H 1.947223 -2.826975 0.695213

C 1.330877 -1.744356 -1.052109

H 1.636302 -2.477786 -1.803174

C 1.423226 -0.369884 -1.714066

C 2.672066 0.186087 -2.023501

C 0.286796 0.355125 -2.071624

C 2.782702 1.422324 -2.644813

H 3.575786 -0.356296 -1.769295

C 0.380412 1.59236 -2.704563

H -0.700518 -0.037879 -1.851678

C 1.631315 2.13283 -2.987044

H 3.756586 1.843977 -2.871468

H -0.519834 2.14013 -2.968644

O 3.637367 -2.275037 -0.34517

O 4.703381 1.447575 2.636484

O 1.789399 3.346758 -3.598362

O -0.142691 -3.532237 -2.547999

O -3.920477 -3.466646 0.314168

O -4.341668 3.439026 -1.344348

O -1.732209 -0.140604 3.146856

H -1.162557 -0.76366 3.617748

H 0.920875 3.728693 -3.774581

H -0.713416 -4.166859 -2.997994

H -4.252765 -3.035208 1.110979

H -5.272942 3.220897 -1.21538

H 3.587196 -3.09616 -0.847365

epi-vaticahainolB_Conf6.log

Energy (E) = -1680.35509535 Hartree

Enthalpy (H) = -1679.893650 Hartree

Gibbs free energy (G) = -1679.982024 Hartree

Charge = 0, Spin = 1

C 3.636003 0.905595 2.291184

C 2.390869 1.473397 2.77685

C 1.241464 0.889377 2.382902

C 1.169084 -0.261757 1.499073

C 2.408517 -0.825575 0.998414

C 3.568038 -0.262121 1.386771

H 2.412333 2.334057 3.434049

H 4.518051 -0.644098 1.032654

C -0.131053 -0.588088 1.324632

C -0.979035 0.410078 2.137539

O 0.008616 1.290801 2.736801

C -1.896016 1.243817 1.264426

C -3.280715 1.081691 1.295802

C -1.341546 2.16799 0.378373

C -4.100141 1.830845 0.461049

H -3.728378 0.36701 1.976226

C -2.150249 2.923611 -0.458672

H -0.265252 2.301955 0.331193

C -3.53544 2.753506 -0.419769

H -5.177427 1.706865 0.484586

H -1.71031 3.641247 -1.144712

C -0.749206 -1.626488 0.488619

C -2.034796 -2.064275 0.829756

C -0.098892 -2.132862 -0.658451

C -2.691612 -2.994005 0.034251

H -2.527777 -1.70012 1.720205

C -0.806553 -3.049375 -1.450778

C -2.086248 -3.48527 -1.11513

H -2.602038 -4.201998 -1.748469

C 2.318888 -1.992698 0.06018

H 1.937152 -2.850979 0.632642

C 1.299943 -1.738024 -1.087358

H 1.592412 -2.460071 -1.854385

C 1.385904 -0.353693 -1.729497

C 2.631423 0.210596 -2.037229

C 0.245343 0.371366 -2.073942

C 2.735012 1.454533 -2.644304

H 3.53821 -0.331737 -1.79419

C 0.331936 1.615831 -2.693601

H -0.739646 -0.02784 -1.85459

C 1.579708 2.164297 -2.974469

H 3.706403 1.882475 -2.869875

H -0.571122 2.162779 -2.949355

O 3.614232 -2.287759 -0.422609

O 4.740105 1.37484 2.609465

O 1.73111 3.385518 -3.573008

O -0.199289 -3.506773 -2.582695

O -3.936603 -3.386186 0.423975

O -4.380234 3.462931 -1.220666

O -1.712764 -0.167298 3.164136

H -1.150546 -0.804158 3.62538

H 0.860634 3.76516 -3.744305

H -0.777932 -4.134911 -3.031542

H -4.286493 -4.022525 -0.211427

H -3.867569 4.056873 -1.782773

H 3.554135 -3.102372 -0.934227

epi-vaticahainolB_Conf7.log

Energy (E) = -1680.35515315 Hartree

Enthalpy (H) = -1679.893713 Hartree

Gibbs free energy (G) = -1679.982021 Hartree

Charge = 0, Spin = 1

C 3.622536 0.965719 2.288873

C 2.372828 1.541956 2.752705

C 1.227336 0.9468 2.364135

C 1.16366 -0.223656 1.505493

C 2.407585 -0.796565 1.026904

C 3.563317 -0.222442 1.410776

H 2.388174 2.416922 3.390907

H 4.516487 -0.610952 1.072548

C -0.13447 -0.556468 1.328896

C -0.989938 0.452867 2.119607

O -0.008935 1.352892 2.700777

C -1.913794 1.261926 1.231049

C -1.366686 2.164673 0.316006

C -3.296167 1.097773 1.275844

C -2.181637 2.893482 -0.536307

H -0.290996 2.300076 0.260143

C -4.123698 1.822834 0.425512

H -3.73903 0.401076 1.977617

C -3.566579 2.72115 -0.483808

H -1.7568 3.594379 -1.246784

H -5.200881 1.691667 0.465744

C -0.744769 -1.612676 0.509804

C -2.032681 -2.044526 0.849675

C -0.085192 -2.141028 -0.622006

C -2.682892 -2.990029 0.067523

H -2.532627 -1.662532 1.728746

C -0.786661 -3.072774 -1.401933

C -2.06874 -3.502778 -1.067762

H -2.579581 -4.231774 -1.691057

C 2.326398 -1.984676 0.1147

H 1.9397 -2.830101 0.70278

C 1.317415 -1.75523 -1.047108

H 1.616571 -2.493566 -1.795857

C 1.410314 -0.384532 -1.716828

C 2.659092 0.167473 -2.033572

C 0.273999 0.340664 -2.074377

C 2.769863 1.400278 -2.661668

H 3.562688 -0.375244 -1.779656

C 0.367694 1.574315 -2.71422

H -0.713241 -0.049435 -1.848973

C 1.618611 2.111084 -3.003665

H 3.743741 1.818946 -2.893833

H -0.532446 2.122326 -2.978096

O 3.625996 -2.289768 -0.349868

O 4.723409 1.444142 2.604531

O 1.776729 3.321601 -3.621778

O -0.170884 -3.551499 -2.520345

O -3.930541 -3.375718 0.455423

O -4.326001 3.455843 -1.344815

O -1.718434 -0.111005 3.157689

H -1.152199 -0.738193 3.627202

H 0.908284 3.704395 -3.796501

H -0.747225 -4.186618 -2.962287

H -4.273423 -4.027424 -0.168102

H -5.257755 3.241887 -1.212431

H 3.569403 -3.111408 -0.850525

epi-vaticahainolB_Conf8.log

Energy (E) = -1680.35498379 Hartree

Enthalpy (H) = -1679.893234 Hartree

Gibbs free energy (G) = -1679.981973 Hartree

Charge = 0, Spin = 1

C 3.214422 1.610807 2.44767

C 1.906422 2.20123 2.664899

C 0.827191 1.479868 2.30076

C 0.889315 0.161606 1.693655

C 2.191112 -0.438971 1.478973

C 3.281325 0.260875 1.847553

H 1.826923 3.187497 3.105325

H 4.27797 -0.147248 1.721351

C -0.370118 -0.255747 1.438013

C -1.334424 0.858658 1.901723

O -0.448643 1.881134 2.427965

C -2.128134 1.465823 0.759419

C -3.490218 1.211193 0.601892

C -1.477846 2.26828 -0.180059

C -4.190036 1.739203 -0.475364

H -4.017168 0.599347 1.324318

C -2.165796 2.79913 -1.262314

H -0.418632 2.482905 -0.078275

C -3.527929 2.530316 -1.414115

H -5.249683 1.542389 -0.597528

H -1.651642 3.422665 -1.987859

C -0.854606 -1.467864 0.760482

C -2.179026 -1.856176 1.001681

C -0.053451 -2.186838 -0.156974

C -2.732537 -2.93348 0.323804

H -2.784871 -1.334215 1.728488

C -0.663624 -3.246575 -0.849045

C -1.983396 -3.625351 -0.618834

H -2.416623 -4.457474 -1.167337

C 2.254356 -1.810959 0.863079

H 1.824458 -2.521336 1.57848

C 1.414364 -1.930337 -0.441729

H 1.795558 -2.855278 -0.878629

C 1.692701 -0.824243 -1.452239

C 0.786335 0.205752 -1.714474

C 2.904867 -0.811946 -2.150831

C 1.083119 1.229907 -2.604492

H -0.183635 0.211537 -1.231034

C 3.221982 0.20726 -3.042666

H 3.613067 -1.625761 -2.021352

C 2.310853 1.238967 -3.264895

H 0.364954 2.021521 -2.794444

H 4.167917 0.196322 -3.576654

O 3.584034 -2.246055 0.665443

O 4.262346 2.20281 2.750295

O 2.56254 2.267682 -4.129042

O 0.087856 -3.908695 -1.773756

O -4.021976 -3.262827 0.613994

O -4.259318 3.016671 -2.456161

O -2.199841 0.478639 2.916911

H -1.706319 -0.0417 3.565053

H 3.434198 2.144182 -4.523984

H -0.422641 -4.637724 -2.146321

H -4.288432 -4.021876 0.081125

H -3.691334 3.537564 -3.037152

H 4.026627 -1.620505 0.07782

epi-vaticahainolB_Conf9.log

Energy (E) = -1680.35521879 Hartree

Enthalpy (H) = -1679.893499 Hartree

Gibbs free energy (G) = -1679.981755 Hartree

Charge = 0, Spin = 1

C 3.21469 1.545384 2.481578

C 1.90539 2.125824 2.717732

C 0.827534 1.411838 2.33555

C 0.89221 0.11101 1.692436

C 2.195071 -0.479579 1.458574

C 3.284193 0.213119 1.843525

H 1.823993 3.099021 3.185984

H 4.281717 -0.188311 1.70362

C -0.366475 -0.303353 1.428222

C -1.332923 0.797103 1.921236

O -0.449334 1.80579 2.475924

C -2.126543 1.434159 0.794885

C -3.487293 1.17901 0.626438

C -1.477957 2.26812 -0.117851

C -4.187434 1.737729 -0.435052

H -4.012723 0.542299 1.328256

C -2.166161 2.830068 -1.184041

H -0.419889 2.483961 -0.007031

C -3.526932 2.561177 -1.346752

H -5.246056 1.540681 -0.565387

H -1.65351 3.478871 -1.888192

C -0.848185 -1.498189 0.719125

C -2.171396 -1.900472 0.955705

C -0.048889 -2.18583 -0.221088

C -2.722874 -2.959739 0.248547

H -2.768073 -1.401436 1.707772

C -0.658697 -3.227829 -0.943273

C -1.974115 -3.617858 -0.719171

H -2.412612 -4.43259 -1.28688

C 2.260396 -1.833576 0.804543

H 1.833902 -2.564925 1.500601

C 1.417651 -1.917763 -0.501556

H 1.799958 -2.828842 -0.965745

C 1.692172 -0.782108 -1.479377

C 2.905846 -0.746855 -2.177589

C 0.785718 0.253387 -1.709192

C 3.223528 0.300065 -3.033892

H 3.613347 -1.564905 -2.072759

C 1.083404 1.307282 -2.566093

H -0.184789 0.244623 -1.226663

C 2.312387 1.338953 -3.22268

H 4.167185 0.313817 -3.569086

H 0.359203 2.101719 -2.72685

O 3.590785 -2.259176 0.592114

O 4.26146 2.131605 2.798952

O 2.665937 2.349958 -4.071766

O 0.092726 -3.857475 -1.890714

O -3.998708 -3.391509 0.453209

O -4.257772 3.078646 -2.373914

O -2.199822 0.389721 2.924666

H -1.703971 -0.135866 3.566822

H 1.945265 2.989806 -4.119607

H -0.413937 -4.580803 -2.279507

H -4.418599 -2.851522 1.134235

H -3.690393 3.62152 -2.935079

H 4.03192 -1.614059 0.024805

epi-vaticahainolB_Conf10.log

Energy (E) = -1680.35496103 Hartree

Enthalpy (H) = -1679.893212 Hartree

Gibbs free energy (G) = -1679.981596 Hartree

Charge = 0, Spin = 1

C 3.194506 1.655417 2.442623

C 1.879934 2.232841 2.654433

C 0.80918 1.498215 2.291776

C 0.886364 0.17787 1.690654

C 2.195012 -0.410106 1.482271

C 3.276878 0.3031 1.849831

H 1.788936 3.220267 3.090021

H 4.278121 -0.095015 1.728091

C -0.36815 -0.253543 1.433927

C -1.344842 0.852967 1.889904

O -0.470933 1.886234 2.415464

C -2.140283 1.45046 0.74374

C -1.492918 2.257422 -0.196476

C -3.499492 1.189665 0.58647

C -2.183966 2.788873 -1.274297

H -0.434263 2.474844 -0.094655

C -4.204117 1.719468 -0.489066

H -4.023679 0.573029 1.306863

C -3.546449 2.519115 -1.423008

H -1.681107 3.417143 -2.001754

H -5.264321 1.51429 -0.6032

C -0.838739 -1.474746 0.762928

C -2.159461 -1.875688 1.004045

C -0.027478 -2.192363 -0.146569

C -2.698708 -2.965788 0.335143

H -2.772853 -1.355065 1.725461

C -0.623721 -3.265386 -0.830302

C -1.939677 -3.657218 -0.599864

H -2.361696 -4.499539 -1.141506

C 2.274623 -1.784648 0.874002

H 1.852161 -2.495995 1.592868

C 1.437716 -1.921185 -0.430972

H 1.829749 -2.84442 -0.861846

C 1.704897 -0.818357 -1.448017

C 0.786995 0.199143 -1.718798

C 2.918012 -0.796707 -2.144718

C 1.073012 1.220145 -2.615912

H -0.183678 0.197066 -1.236712

C 3.224468 0.21959 -3.043592

H 3.635447 -1.601283 -2.008678

C 2.301435 1.238685 -3.274872

H 0.345921 2.001929 -2.81249

H 4.171295 0.215681 -3.576063

O 3.609484 -2.205403 0.679991

O 4.235537 2.260082 2.744022

O 2.541815 2.263677 -4.146598

O 0.137977 -3.927357 -1.746664

O -3.984448 -3.308177 0.626792

O -4.182367 3.066312 -2.496846

O -2.209771 0.468632 2.903854

H -1.712798 -0.042898 3.55638

H 3.415286 2.147705 -4.539766

H -0.362568 -4.666107 -2.113454

H -4.238195 -4.080645 0.107171

H -5.115735 2.82122 -2.47831

H 4.045449 -1.577635 0.089751

epi-vaticahainolB_Conf11.log

Energy (E) = -1680.35519008 Hartree

Enthalpy (H) = -1679.893380 Hartree

Gibbs free energy (G) = -1679.981485 Hartree

Charge = 0, Spin = 1

C 3.177762 1.674269 2.450252

C 1.85905 2.244142 2.657455

C 0.793784 1.502852 2.292363

C 0.880644 0.182316 1.69306

C 2.193245 -0.398203 1.489599

C 3.269877 0.321699 1.859493

H 1.760932 3.231547 3.091547

H 4.273741 -0.070819 1.741172

C -0.370491 -0.256573 1.43225

C -1.354911 0.844715 1.884156

O -0.489053 1.883532 2.411465

C -2.150779 1.436302 0.735276

C -1.504658 2.244106 -0.205088

C -3.508672 1.170051 0.575681

C -2.195528 2.770919 -1.285251

H -0.447063 2.465823 -0.101486

C -4.213176 1.695145 -0.502241

H -4.032178 0.552927 1.296207

C -3.556684 2.495781 -1.436203

H -1.693605 3.399813 -2.012806

H -5.272352 1.485732 -0.618152

C -0.83187 -1.480226 0.759829

C -2.154254 -1.886696 0.994243

C -0.014447 -2.193545 -0.144806

C -2.686165 -2.977618 0.32111

H -2.766152 -1.363864 1.717419

C -0.603979 -3.269746 -0.832906

C -1.917995 -3.665344 -0.610191

H -2.340288 -4.506566 -1.15096

C 2.282416 -1.77308 0.883618

H 1.861714 -2.485779 1.602195

C 1.450618 -1.916285 -0.423748

H 1.848333 -2.838672 -0.851235

C 1.715891 -0.814517 -1.442501

C 0.793945 0.198016 -1.718167

C 2.930939 -0.788709 -2.135695

C 1.077591 1.21827 -2.616828

H -0.178083 0.192217 -1.238857

C 3.235007 0.226976 -3.036103

H 3.651753 -1.589612 -1.996028

C 2.307751 1.241075 -3.272429

H 0.347249 1.996021 -2.817319

H 4.183255 0.226219 -3.566049

O 3.62022 -2.186495 0.694182

O 4.214178 2.285539 2.75399

O 2.545437 2.265024 -4.146109

O 0.166382 -3.927333 -1.745284

O -3.960654 -3.413566 0.525568

O -4.192331 3.038922 -2.51218

O -2.220446 0.455413 2.896027

H -1.720778 -0.048472 3.552527

H 3.420571 2.152553 -4.53657

H -0.329765 -4.66711 -2.116059

H -4.395589 -2.850077 1.177543

H -5.124913 2.790744 -2.495324

H 4.053901 -1.557461 0.103584

epi-vaticahainolB_Conf12.log

Energy (E) = -1680.35521718 Hartree

Enthalpy (H) = -1679.893338 Hartree

Gibbs free energy (G) = -1679.981457 Hartree

Charge = 0, Spin = 1

C 3.197871 1.593401 2.475923

C 1.887201 2.178082 2.693122

C 0.811656 1.457244 2.317339

C 0.880316 0.144984 1.698297

C 2.184585 -0.449783 1.483395

C 3.271318 0.249602 1.862963

H 1.803037 3.159834 3.142662

H 4.269671 -0.154325 1.736725

C -0.376683 -0.27377 1.433122

C -1.346201 0.834088 1.901971

O -0.466047 1.853616 2.442521

C -2.135458 1.449811 0.761104

C -3.495905 1.192858 0.593381

C -1.482448 2.263764 -0.166525

C -4.191402 1.729856 -0.482229

H -4.024817 0.571876 1.306588

C -2.165976 2.803622 -1.247072

H -0.424499 2.480588 -0.056629

C -3.52648 2.532564 -1.409112

H -5.24978 1.531313 -0.612314

H -1.649659 3.436078 -1.963289

C -0.854234 -1.480393 0.74145

C -2.179647 -1.877111 0.97499

C -0.048788 -2.185035 -0.180689

C -2.726968 -2.948273 0.28267

H -2.781605 -1.363185 1.712727

C -0.654406 -3.238885 -0.88909

C -1.971715 -3.624086 -0.667717

H -2.406542 -4.448536 -1.224111

C 2.253717 -1.815322 0.854182

H 1.822236 -2.533858 1.560432

C 1.419954 -1.923238 -0.455659

H 1.804584 -2.843453 -0.899451

C 1.701914 -0.807297 -1.454135

C 0.797197 0.226076 -1.708631

C 2.916602 -0.788605 -2.148151

C 1.097998 1.259425 -2.586577

H -0.174496 0.227604 -1.228594

C 3.237645 0.239766 -3.027961

H 3.623825 -1.604158 -2.024275

C 2.328115 1.274516 -3.24248

H 0.381052 2.053524 -2.770721

H 4.185466 0.233844 -3.55868

O 3.585216 -2.245292 0.657963

O 4.24271 2.185441 2.788954

O 2.583602 2.312119 -4.094779

O 0.103524 -3.8854 -1.819735

O -4.004654 -3.37531 0.485719

O -4.253434 3.027599 -2.450074

O -2.216309 0.442347 2.908882

H -1.722847 -0.076015 3.558722

H 3.455822 2.19135 -4.489354

H -0.402277 -4.612569 -2.202323

H -4.429621 -2.821814 1.152624

H -3.68389 3.556294 -3.022469

H 4.028792 -1.612516 0.078834

epi-vaticahainolB_Conf13.log

Energy (E) = -1680.35523229 Hartree

Enthalpy (H) = -1679.893385 Hartree

Gibbs free energy (G) = -1679.981309 Hartree

Charge = 0, Spin = 1

C 3.129454 1.878374 2.373576

C 1.805262 2.447464 2.540838

C 0.748282 1.674019 2.2211

C 0.848183 0.317375 1.709615

C 2.166955 -0.262002 1.546579

C 3.235296 0.492116 1.870245

H 1.696159 3.459947 2.909651

H 4.242913 0.102389 1.77856

C -0.398798 -0.146568 1.471734

C -1.391451 0.981788 1.828895

O -0.537725 2.051335 2.310603

C -2.149137 1.4958 0.617832

C -1.471627 2.246258 -0.347829

C -3.497507 1.205842 0.422981

C -2.121983 2.692586 -1.487555

H -0.42063 2.484926 -0.217193

C -4.161679 1.650381 -0.715334

H -4.045141 0.633192 1.162032

C -3.473164 2.392083 -1.674682

H -1.595846 3.276236 -2.235386

H -5.213818 1.423393 -0.859395

C -0.850259 -1.414157 0.877904

C -2.177216 -1.80279 1.119172

C -0.021697 -2.189419 0.036797

C -2.70259 -2.935632 0.514276

H -2.799715 -1.229969 1.793326

C -0.606016 -3.307071 -0.587732

C -1.92356 -3.685508 -0.358056

H -2.340257 -4.559984 -0.847882

C 2.277856 -1.675842 1.040416

H 1.877436 -2.341388 1.813904

C 1.447432 -1.940362 -0.247401

H 1.84506 -2.900411 -0.581874

C 1.726596 -0.94587 -1.365962

C 2.91543 -1.046481 -2.099032

C 0.849712 0.088848 -1.694163

C 3.238403 -0.12746 -3.090154

H 3.596571 -1.872146 -1.911185

C 1.15353 1.015578 -2.684846

H -0.10301 0.176963 -1.185386

C 2.35843 0.915217 -3.379242

H 4.162144 -0.217047 -3.652125

H 0.453035 1.812518 -2.920829

O 3.623476 -2.07225 0.869359

O 4.159538 2.517085 2.640423

O 2.718059 1.796734 -4.360014

O 0.173763 -4.023442 -1.445944

O -3.981235 -3.353684 0.730015

O -4.067055 2.851972 -2.811589

O -2.286932 0.662827 2.838353

H -1.809798 0.195679 3.537303

H 2.021477 2.455761 -4.467843

H -0.325232 -4.777041 -1.783469

H -4.420356 -2.750699 1.342777

H -4.993677 2.582585 -2.823201

H 4.032775 -1.486914 0.218735

epi-vaticahainolB_Conf14.log

Energy (E) = -1680.35503250 Hartree

Enthalpy (H) = -1679.893260 Hartree

Gibbs free energy (G) = -1679.981299 Hartree

Charge = 0, Spin = 1

C 3.22228 1.587784 2.446784

C 1.912475 2.168846 2.677995

C 0.835083 1.447206 2.309034

C 0.900782 0.13764 1.683762

C 2.204393 -0.453768 1.455247

C 3.292912 0.246389 1.82815

H 1.830248 3.148503 3.132449

H 4.291013 -0.154949 1.692106

C -0.357715 -0.282649 1.428485

C -1.325338 0.821011 1.91087

O -0.442086 1.840143 2.447899

C -2.126524 1.442259 0.781262

C -1.484174 2.266184 -0.147491

C -3.487121 1.186267 0.62747

C -2.181096 2.818135 -1.211221

H -0.424774 2.480942 -0.047693

C -4.197562 1.73647 -0.433808

H -4.007574 0.556847 1.339431

C -3.544492 2.551839 -1.357319

H -1.681999 3.459588 -1.929711

H -5.258614 1.534338 -0.545426

C -0.838915 -1.489087 0.738611

C -2.158174 -1.891197 0.985517

C -0.039124 -2.190139 -0.193766

C -2.707777 -2.96529 0.299327

H -2.76177 -1.385159 1.725308

C -0.646249 -3.245738 -0.894742

C -1.961008 -3.638256 -0.658665

H -2.391696 -4.467113 -1.214071

C 2.271011 -1.816618 0.819846

H 1.847032 -2.538996 1.526702

C 1.42533 -1.920056 -0.48313

H 1.809853 -2.835217 -0.937395

C 1.692263 -0.795017 -1.475342

C 2.906077 -0.759685 -2.173372

C 0.778294 0.230614 -1.7192

C 3.216497 0.278408 -3.042923

H 3.619511 -1.571149 -2.058296

C 1.068588 1.275594 -2.589512

H -0.192471 0.220564 -1.237247

C 2.297924 1.30815 -3.245339

H 4.160425 0.292342 -3.577638

H 0.338654 2.062545 -2.760704

O 3.6017 -2.24315 0.610941

O 4.268716 2.180691 2.75265

O 2.644854 2.311189 -4.106487

O 0.103269 -3.889714 -1.833814

O -3.991201 -3.310841 0.597451

O -4.186108 3.11802 -2.417798

O -2.185567 0.42407 2.923992

H -1.686557 -0.097836 3.566632

H 1.920522 2.946356 -4.16046

H -0.402679 -4.619968 -2.210242

H -4.252471 -4.071138 0.063762

H -5.118527 2.86922 -2.400421

H 4.041411 -1.604749 0.035064

epi-vaticahainolB_Conf15.log

Energy (E) = -1680.35274041 Hartree

Enthalpy (H) = -1679.891931 Hartree

Gibbs free energy (G) = -1679.981223 Hartree

Charge = 0, Spin = 1

C 3.51443 1.002736 2.378648

C 2.258866 1.615738 2.777825

C 1.116922 1.017786 2.383089

C 1.066347 -0.187403 1.574864

C 2.313638 -0.804426 1.172198

C 3.46658 -0.22937 1.561465

H 2.268698 2.519447 3.374723

H 4.423485 -0.652991 1.280624

C -0.227339 -0.508506 1.357225

C -1.09708 0.541005 2.07737

O -0.12508 1.450924 2.660259

C -1.976407 1.327811 1.126562

C -1.383441 2.191872 0.202528

C -3.360756 1.176486 1.120253

C -2.155223 2.892943 -0.711017

H -0.305413 2.318228 0.18715

C -4.14557 1.875861 0.209888

H -3.838271 0.509339 1.828124

C -3.543043 2.734036 -0.709193

H -1.695238 3.564721 -1.427809

H -5.224808 1.755605 0.210623

C -0.823432 -1.572899 0.536947

C -2.127514 -1.976322 0.841691

C -0.140405 -2.122258 -0.57526

C -2.781674 -2.904999 0.0406

H -2.640814 -1.580595 1.707123

C -0.864273 -2.996868 -1.400288

C -2.161892 -3.401556 -1.096556

H -2.67262 -4.092192 -1.759858

C 2.230146 -2.04027 0.329787

H 1.764577 -2.830954 0.935587

C 1.309266 -1.827514 -0.911503

H 1.644545 -2.629384 -1.5779

C 1.533463 -0.505928 -1.643179

C 2.828301 -0.120288 -2.015774

C 0.480775 0.344258 -1.979187

C 3.064793 1.073028 -2.683071

H 3.666214 -0.763297 -1.770341

C 0.700775 1.543835 -2.651856

H -0.537706 0.082536 -1.711559

C 1.996223 1.912206 -3.00371

H 4.072458 1.362477 -2.962901

H -0.134534 2.193908 -2.897829

O 3.53729 -2.430022 -0.035901

O 4.611308 1.484288 2.702612

O 2.277927 3.076859 -3.662831

O -0.348133 -3.534301 -2.547601

O -4.043062 -3.269081 0.402966

O -4.25934 3.442717 -1.626811

O -1.869011 0.022205 3.107467

H -1.330097 -0.600094 3.614616

H 1.45604 3.554014 -3.83024

H 0.378281 -2.994816 -2.879694

H -4.387785 -3.906863 -0.23395

H -5.198291 3.24521 -1.522457

H 3.481226 -3.308963 -0.427382

epi-vaticahainolB_Conf16.log

Energy (E) = -1680.35266147 Hartree

Enthalpy (H) = -1679.891827 Hartree

Gibbs free energy (G) = -1679.980923 Hartree

Charge = 0, Spin = 1

C 3.518346 0.976956 2.377066

C 2.267427 1.602298 2.771722

C 1.121092 1.013456 2.376053

C 1.061672 -0.193359 1.570872

C 2.304286 -0.821989 1.171621

C 3.461389 -0.25661 1.562691

H 2.283983 2.507449 3.366285

H 4.415136 -0.689049 1.284544

C -0.234302 -0.504322 1.352357

C -1.096432 0.55315 2.070038

O -0.117738 1.458027 2.6496

C -1.972063 1.342857 1.118391

C -1.375329 2.202018 0.192272

C -3.357165 1.19813 1.112861

C -2.144034 2.904438 -0.722855

H -0.29675 2.323249 0.176385

C -4.138909 1.899001 0.201056

H -3.837686 0.53508 1.82254

C -3.532553 2.751945 -0.720407

H -1.681014 3.572101 -1.441543

H -5.21871 1.783843 0.202358

C -0.83744 -1.566336 0.534208

C -2.143529 -1.961708 0.840601

C -0.158063 -2.121816 -0.577189

C -2.803194 -2.888597 0.041924

H -2.653922 -1.561521 1.705697

C -0.887227 -2.993988 -1.400032

C -2.186923 -3.390815 -1.09464

H -2.701894 -4.080126 -1.756047

C 2.211472 -2.057641 0.32974

H 1.738357 -2.844024 0.935226

C 1.293799 -1.83721 -0.912942

H 1.624102 -2.641016 -1.579429

C 1.52877 -0.515923 -1.64202

C 0.480942 0.340465 -1.983774

C 2.826496 -0.134125 -2.00233

C 0.710726 1.542423 -2.645738

H -0.540347 0.080083 -1.725559

C 3.073396 1.064218 -2.65998

H 3.660779 -0.780919 -1.754534

C 2.010909 1.910418 -2.981092

H -0.114477 2.201417 -2.895655

H 4.088648 1.345527 -2.925471

O 3.515613 -2.459005 -0.034215

O 4.618764 1.449553 2.702294

O 2.19131 3.104681 -3.622631

O -0.374675 -3.536379 -2.546563

O -4.066185 -3.245308 0.406021

O -4.2458 3.461567 -1.639681

O -1.870549 0.04177 3.102378

H -1.334777 -0.582234 3.610751

H 3.133197 3.242875 -3.779439

H 0.355753 -3.002395 -2.878784

H -4.414506 -3.883056 -0.228971

H -5.185567 3.267946 -1.535341

H 3.452414 -3.338474 -0.423506

epi-vaticahainolB_Conf17.log

Energy (E) = -1680.35312546 Hartree

Enthalpy (H) = -1679.891319 Hartree

Gibbs free energy (G) = -1679.980721 Hartree

Charge = 0, Spin = 1

C 3.17136 1.878384 2.312295

C 1.866229 2.499424 2.444893

C 0.784775 1.747196 2.157838

C 0.84312 0.365393 1.713995

C 2.140772 -0.263911 1.580968

C 3.233388 0.467013 1.874383

H 1.7909 3.53191 2.76317

H 4.227839 0.040423 1.805768

C -0.415926 -0.073193 1.497554

C -1.375054 1.101584 1.793521

O -0.489263 2.168914 2.219673

C -2.115675 1.567149 0.552649

C -3.467103 1.280802 0.360985

C -1.422519 2.255674 -0.445002

C -4.115226 1.66942 -0.804333

H -4.025606 0.752675 1.124497

C -2.058471 2.645556 -1.615512

H -0.370203 2.490805 -0.318547

C -3.41076 2.348776 -1.798194

H -5.166869 1.449355 -0.953

H -1.511505 3.181598 -2.3856

C -0.904048 -1.348249 0.951346

C -2.23069 -1.700088 1.226088

C -0.109003 -2.1582 0.103499

C -2.799603 -2.826517 0.64507

H -2.830467 -1.104346 1.899611

C -0.749445 -3.237099 -0.528719

C -2.069066 -3.585355 -0.257872

H -2.51022 -4.439356 -0.761672

C 2.196668 -1.700686 1.137573

H 1.753097 -2.316208 1.928502

C 1.376463 -1.980561 -0.156552

H 1.756402 -2.971706 -0.423962

C 1.718763 -1.045136 -1.30787

C 2.909393 -1.224161 -2.022398

C 0.889428 0.013413 -1.681565

C 3.282009 -0.354157 -3.040111

H 3.552094 -2.070517 -1.795447

C 1.244016 0.891343 -2.698362

H -0.064133 0.159101 -1.187601

C 2.451615 0.715683 -3.37355

H 4.206833 -0.50261 -3.587562

H 0.581164 1.708785 -2.969945

O 3.523969 -2.163867 1.003873

O 4.221119 2.494328 2.554489

O 2.858815 1.54848 -4.376784

O -0.112394 -4.04024 -1.435637

O -4.088966 -3.118175 0.972618

O -4.090909 2.701293 -2.925167

O -2.282269 0.866338 2.815239

H -1.819403 0.440236 3.549279

H 2.193635 2.234838 -4.509812

H 0.620454 -3.568833 -1.848568

H -4.371269 -3.90984 0.498263

H -3.496601 3.161307 -3.530646

H 3.970554 -1.621155 0.341122

epi-vaticahainolB_Conf18.log

Energy (E) = -1680.35285012 Hartree

Enthalpy (H) = -1679.891765 Hartree

Gibbs free energy (G) = -1679.980427 Hartree

Charge = 0, Spin = 1

C 3.560205 0.947874 2.338862

C 2.311829 1.545569 2.782359

C 1.162358 0.956553 2.395658

C 1.097128 -0.229657 1.561121

C 2.336586 -0.83114 1.113991

C 3.496187 -0.258595 1.485628

H 2.332992 2.43476 3.400402

H 4.448583 -0.662405 1.159903

C -0.200288 -0.543738 1.353569

C -1.056956 0.491411 2.109931

O -0.074474 1.383725 2.703621

C -1.941781 1.302912 1.185196

C -1.352254 2.168041 0.260035

C -3.328151 1.172838 1.202891

C -2.129551 2.891282 -0.63119

H -0.272668 2.277628 0.226154

C -4.11845 1.894366 0.314805

H -3.802491 0.504786 1.912018

C -3.5193 2.7531 -0.606076

H -1.672014 3.562738 -1.349818

H -5.199199 1.790297 0.333821

C -0.811449 -1.585985 0.515578

C -2.114093 -1.988112 0.827597

C -0.144899 -2.118438 -0.615303

C -2.782509 -2.898749 0.017409

H -2.615802 -1.605868 1.70577

C -0.880807 -2.977531 -1.445603

C -2.177252 -3.380825 -1.133298

H -2.698253 -4.058916 -1.801531

C 2.231982 -2.052882 0.243887

H 1.771572 -2.850256 0.843707

C 1.297708 -1.808851 -0.970263

H 1.633787 -2.588753 -1.660913

C 1.493809 -0.463655 -1.669332

C 2.782028 -0.012989 -1.987836

C 0.415536 0.339094 -2.039374

C 2.986614 1.196487 -2.636255

H 3.641791 -0.615874 -1.716745

C 0.603593 1.553268 -2.695474

H -0.598691 0.028682 -1.810296

C 1.892323 1.98649 -2.993024

H 3.989509 1.536677 -2.872698

H -0.251692 2.164983 -2.96928

O 3.483801 -2.494518 -0.242995

O 4.662544 1.423714 2.652184

O 2.142761 3.169438 -3.631485

O -0.3831 -3.499085 -2.607396

O -4.04235 -3.260905 0.387012

O -4.240827 3.481252 -1.504225

O -1.82003 -0.046151 3.136559

H -1.276784 -0.677838 3.627141

H 1.305854 3.610955 -3.82094

H 0.362139 -2.977315 -2.925525

H -4.398062 -3.885644 -0.256754

H -5.180491 3.294493 -1.387407

H 3.992457 -2.831719 0.503861

epi-vaticahainolB_Conf19.log

Energy (E) = -1680.35274247 Hartree

Enthalpy (H) = -1679.891656 Hartree

Gibbs free energy (G) = -1679.980415 Hartree

Charge = 0, Spin = 1

C 3.502479 0.970604 2.401611

C 2.246728 1.587754 2.793995

C 1.1052 0.998047 2.386026

C 1.05556 -0.201795 1.570017

C 2.302906 -0.822106 1.172774

C 3.455403 -0.255867 1.575996

H 2.256229 2.487526 3.396814

H 4.412532 -0.682213 1.30006

C -0.237888 -0.516587 1.341873

C -1.108394 0.529887 2.065469

O -0.137172 1.435062 2.656271

C -1.98589 1.322454 1.118061

C -1.390832 2.189376 0.198088

C -3.370523 1.173647 1.110276

C -2.160668 2.895157 -0.713431

H -0.312606 2.314034 0.184294

C -4.153459 1.877865 0.202071

H -3.849745 0.504855 1.815486

C -3.548739 2.73835 -0.713443

H -1.698927 3.568716 -1.427416

H -5.232918 1.759589 0.201574

C -0.8318 -1.574265 0.5119

C -2.139155 -1.979072 0.809403

C -0.145053 -2.117356 -0.598889

C -2.789095 -2.904482 0.001539

H -2.648742 -1.584126 1.679153

C -0.866246 -2.98897 -1.43233

C -2.163125 -3.39468 -1.135914

H -2.677185 -4.081478 -1.798676

C 2.219678 -2.050812 0.319812

H 1.747003 -2.844437 0.91612

C 1.307193 -1.823577 -0.925331

H 1.643719 -2.620365 -1.597011

C 1.540216 -0.495278 -1.64218

C 0.490749 0.359351 -1.983231

C 2.838414 -0.105162 -1.991766

C 0.719303 1.567534 -2.634308

H -0.530928 0.092843 -1.732858

C 3.084032 1.09942 -2.638298

H 3.673937 -0.750428 -1.744212

C 2.019861 1.943672 -2.959016

H -0.107203 2.224985 -2.883981

H 4.099606 1.387126 -2.895557

O 3.527329 -2.443382 -0.041007

O 4.599023 1.444321 2.738077

O 2.199141 3.143664 -3.590063

O -0.3429 -3.520584 -2.579353

O -4.051763 -3.343125 0.263832

O -4.263196 3.451283 -1.629085

O -1.882734 0.004091 3.090749

H -1.343295 -0.618119 3.597542

H 3.141416 3.286917 -3.73992

H 0.381209 -2.976059 -2.908113

H -4.384766 -2.912555 1.060823

H -5.202441 3.254037 -1.526895

H 3.47015 -3.320145 -0.437251

epi-vaticahainolB_Conf20.log

Energy (E) = -1680.35267647 Hartree

Enthalpy (H) = -1679.891656 Hartree

Gibbs free energy (G) = -1679.980347 Hartree

Charge = 0, Spin = 1

C 3.51124 0.968228 2.393932

C 2.254776 1.571488 2.804479

C 1.113852 0.977513 2.400815

C 1.064765 -0.214126 1.572666

C 2.313191 -0.821479 1.158764

C 3.46517 -0.250114 1.556365

H 2.26292 2.465204 3.416273

H 4.422753 -0.666621 1.267345

C -0.228688 -0.53395 1.351009

C -1.099574 0.504118 2.08615

O -0.128534 1.40346 2.686683

C -1.97444 1.308003 1.145118

C -3.360484 1.157619 1.128145

C -1.376838 2.190165 0.244038

C -4.139445 1.878844 0.232158

H -3.841052 0.474781 1.818811

C -2.144324 2.914695 -0.656951

H -0.29857 2.31535 0.234779

C -3.532199 2.759846 -0.662664

H -5.218155 1.766238 0.219556

H -1.67171 3.601171 -1.353132

C -0.823681 -1.5872 0.515698

C -2.126791 -1.997024 0.815927

C -0.140165 -2.120533 -0.604063

C -2.77921 -2.917568 0.004048

H -2.64065 -1.613061 1.686304

C -0.86191 -2.988116 -1.438378

C -2.15833 -3.399787 -1.138588

H -2.66725 -4.084473 -1.809381

C 2.232293 -2.043634 0.296323

H 1.771278 -2.84586 0.890272

C 1.307472 -1.814499 -0.938984

H 1.645266 -2.603888 -1.618729

C 1.524157 -0.480835 -1.650817

C 2.816904 -0.08233 -2.017143

C 0.466433 0.367008 -1.976882

C 3.046452 1.120469 -2.669668

H 3.658591 -0.723144 -1.779028

C 0.679531 1.575857 -2.635074

H -0.550587 0.095819 -1.713111

C 1.972845 1.956445 -2.981666

H 4.052517 1.419568 -2.94508

H -0.159535 2.223467 -2.874598

O 3.540032 -2.422591 -0.078691

O 4.607448 1.446789 2.724629

O 2.247666 3.13031 -3.627263

O -0.345825 -3.511768 -2.591912

O -4.039653 -3.288432 0.362643

O -4.33939 3.445833 -1.520535

O -1.875751 -0.029056 3.10549

H -1.338665 -0.657892 3.606522

H 1.422807 3.603499 -3.791214

H 0.38325 -2.97103 -2.915868

H -4.383936 -3.918832 -0.281812

H -3.80237 4.02582 -2.074344

H 3.487144 -3.297097 -0.480455

epi-vaticahainolB_Conf21.log

Energy (E) = -1680.35281633 Hartree

Enthalpy (H) = -1679.891690 Hartree

Gibbs free energy (G) = -1679.980255 Hartree

Charge = 0, Spin = 1

C 3.504944 0.97966 2.404218

C 2.245735 1.583312 2.806214

C 1.107517 0.988088 2.397026

C 1.06421 -0.205306 1.571225

C 2.315134 -0.812239 1.164602

C 3.464538 -0.239799 1.567963

H 2.250145 2.477927 3.416715

H 4.424023 -0.656129 1.28504

C -0.227629 -0.528146 1.344854

C -1.103464 0.507446 2.077667

O -0.137132 1.413427 2.67504

C -1.985157 1.302604 1.136163

C -1.39447 2.179462 0.222791

C -3.369026 1.147092 1.127418

C -2.167934 2.888636 -0.682999

H -0.316799 2.30926 0.209611

C -4.155567 1.854553 0.22483

H -3.844796 0.470497 1.827497

C -3.555223 2.725089 -0.684028

H -1.709599 3.569938 -1.391812

H -5.234423 1.731011 0.223644

C -0.816815 -1.583594 0.508634

C -2.121396 -1.997814 0.80562

C -0.129174 -2.114936 -0.607285

C -2.767774 -2.920965 -0.00766

H -2.631721 -1.611563 1.678847

C -0.846921 -2.985179 -1.445245

C -2.141005 -3.400053 -1.149359

H -2.652412 -4.085082 -1.815992

C 2.2391 -2.035422 0.30316

H 1.775921 -2.837348 0.895858

C 1.319703 -1.808109 -0.936484

H 1.660079 -2.598312 -1.61399

C 1.5388 -0.474932 -1.64844

C 2.833082 -0.075179 -2.007893

C 0.481996 0.371744 -1.980339

C 3.064883 1.128088 -2.658744

H 3.674154 -0.715148 -1.765391

C 0.697356 1.581193 -2.636673

H -0.536173 0.099543 -1.72215

C 1.992184 1.963332 -2.975875

H 4.072132 1.428276 -2.928589

H -0.141172 2.228156 -2.879891

O 3.548732 -2.413843 -0.065547

O 4.598883 1.458853 2.741303

O 2.269465 3.137981 -3.618915

O -0.323216 -3.506348 -2.596827

O -4.02778 -3.368025 0.253312

O -4.273241 3.441589 -1.594082

O -1.874724 -0.029847 3.099142

H -1.331522 -0.652087 3.60186

H 1.445136 3.61083 -3.786561

H 0.398967 -2.95744 -2.922425

H -4.362292 -2.944249 1.053298

H -5.211401 3.238308 -1.493816

H 3.498085 -3.288473 -0.46731

epi-vaticahainolB_Conf22.log

Energy (E) = -1680.35301001 Hartree

Enthalpy (H) = -1679.891493 Hartree

Gibbs free energy (G) = -1679.980221 Hartree

Charge = 0, Spin = 1

C -3.164094 -1.898715 2.312248

C -1.858138 -2.518864 2.439905

C -0.77802 -1.764365 2.153751

C -0.838114 -0.380666 1.715843

C -2.136845 0.247784 1.588134

C -3.228149 -0.485704 1.880076

H -1.781027 -3.552563 2.753776

H -4.223032 -0.059656 1.814586

C 0.420373 0.059646 1.498995

C 1.380336 -1.117915 1.782625

O 0.49643 -2.185385 2.21169

C 2.108126 -1.579058 0.532294

C 1.405558 -2.272867 -0.457395

C 3.453487 -1.283829 0.323064

C 2.028107 -2.65863 -1.634404

H 0.35631 -2.513436 -0.316996

C 4.089737 -1.668403 -0.8522

H 4.019215 -0.751953 1.078689

C 3.375913 -2.352811 -1.835542

H 1.482095 -3.197471 -2.401338

H 5.139351 -1.437414 -1.008155

C 0.906487 1.33997 0.962839

C 2.232724 1.690996 1.240427

C 0.109053 2.158433 0.125252

C 2.798858 2.82587 0.673265

H 2.834438 1.088338 1.905901

C 0.745749 3.248866 -0.491043

C 2.065116 3.596038 -0.217162

H 2.503336 4.458368 -0.709174

C -2.196426 1.686693 1.152248

H -1.755993 2.299061 1.947383

C -1.375396 1.977573 -0.138723

H -1.758667 2.969247 -0.398698

C -1.712633 1.050902 -1.298907

C -0.87998 -0.004919 -1.67894

C -2.898043 1.236393 -2.017414

C -1.227422 -0.870375 -2.706464

H 0.070556 -0.155088 -1.180675

C -3.264113 0.377366 -3.04916

H -3.543639 2.079107 -1.785257

C -2.429851 -0.686831 -3.389541

H -0.568714 -1.685786 -2.988741

H -4.187827 0.539099 -3.597145

O -3.524927 2.146704 1.019228

O -4.21287 -2.516508 2.553976

O -2.729196 -1.565734 -4.391441

O 0.106633 4.065285 -1.384363

O 4.088494 3.114873 1.002229

O 3.942062 -2.749497 -3.009777

O 2.297876 -0.890504 2.796746

H 1.843491 -0.467984 3.538139

H -3.578281 -1.328073 -4.783044

H -0.630784 3.602729 -1.798829

H 4.369248 3.9127 0.537349

H 4.867566 -2.476654 -3.029347

H -3.968055 1.608059 0.350851

epi-vaticahainolB_Conf23.log

Energy (E) = -1680.35290775 Hartree

Enthalpy (H) = -1679.891723 Hartree

Gibbs free energy (G) = -1679.980146 Hartree

Charge = 0, Spin = 1

C 3.538991 0.943361 2.36565

C 2.28585 1.536821 2.801681

C 1.140723 0.947092 2.403483

C 1.084931 -0.235473 1.563425

C 2.328914 -0.832737 1.123642

C 3.484407 -0.25951 1.506655

H 2.300454 2.423365 3.423698

H 4.440187 -0.660161 1.186984

C -0.210071 -0.551243 1.343787

C -1.074954 0.476614 2.100638

O -0.099649 1.370038 2.703437

C -1.959919 1.288227 1.176333

C -1.370513 2.160033 0.257298

C -3.345673 1.151494 1.187401

C -2.147289 2.882734 -0.634788

H -0.291375 2.27516 0.228813

C -4.135524 1.872745 0.298685

H -3.820009 0.478508 1.891919

C -3.536512 2.737725 -0.616356

H -1.689947 3.55946 -1.348583

H -5.215864 1.763805 0.312627

C -0.81192 -1.589757 0.495054

C -2.117324 -1.998347 0.795758

C -0.136455 -2.112434 -0.632867

C -2.776116 -2.906691 -0.024332

H -2.618985 -1.619525 1.677202

C -0.864987 -2.968856 -1.475357

C -2.160264 -3.377802 -1.174923

H -2.680677 -4.052312 -1.845306

C 2.232792 -2.050607 0.24736

H 1.767275 -2.850929 0.839279

C 1.309173 -1.800426 -0.973893

H 1.650611 -2.576625 -1.666055

C 1.512742 -0.45138 -1.663163

C 2.804216 -0.006907 -1.977174

C 0.440199 0.362068 -2.026128

C 3.017512 1.206725 -2.614815

H 3.65944 -0.618231 -1.710594

C 0.637025 1.581041 -2.670709

H -0.57634 0.05692 -1.800337

C 1.928844 2.007661 -2.964531

H 4.022827 1.541852 -2.848194

H -0.213908 2.20153 -2.938374

O 3.488994 -2.489504 -0.230368

O 4.6378 1.420009 2.689984

O 2.187975 3.194465 -3.592251

O -0.355138 -3.480067 -2.636672

O -4.037717 -3.347155 0.240197

O -4.257773 3.46587 -1.514561

O -1.840202 -0.071211 3.12063

H -1.294673 -0.700268 3.612154

H 1.35424 3.641735 -3.782226

H 0.388105 -2.950635 -2.946521

H -4.363238 -2.929972 1.047322

H -5.196813 3.272479 -1.403533

H 3.991588 -2.828728 0.519688

epi-vaticahainolB_Conf24.log

Energy (E) = -1680.35199057 Hartree

Enthalpy (H) = -1679.890959 Hartree

Gibbs free energy (G) = -1679.979895 Hartree

Charge = 0, Spin = 1

C 3.608765 0.78555 2.343868

C 2.37401 1.389786 2.8149

C 1.212372 0.841252 2.405872

C 1.120212 -0.309908 1.526362

C 2.346392 -0.908573 1.039545

C 3.517946 -0.376616 1.433744

H 2.414006 2.252161 3.468952

H 4.461471 -0.779403 1.082

C -0.183655 -0.603085 1.327126

C -1.011782 0.419239 2.124842

O -0.015229 1.285932 2.726262

C -1.899034 1.27443 1.236787

C -1.318016 2.212628 0.37869

C -3.281808 1.105298 1.214655

C -2.099719 2.971543 -0.477837

H -0.241303 2.350194 0.370374

C -4.077005 1.862919 0.36178

H -3.756358 0.375817 1.862005

C -3.485815 2.796441 -0.488278

H -1.648594 3.699255 -1.143776

H -5.154156 1.72666 0.352253

C -0.815781 -1.609743 0.461761

C -2.112036 -2.026257 0.787964

C -0.175321 -2.095662 -0.702546

C -2.796366 -2.907409 -0.040906

H -2.583668 -1.678498 1.697878

C -0.928208 -2.927404 -1.54834

C -2.215259 -3.343851 -1.222499

H -2.755865 -3.998079 -1.897011

C 2.217482 -2.080811 0.106063

H 1.765711 -2.908013 0.670857

C 1.259231 -1.768076 -1.073283

H 1.580156 -2.508931 -1.812257

C 1.443374 -0.387082 -1.702361

C 0.356036 0.418477 -2.045061

C 2.726513 0.095983 -1.984269

C 0.532618 1.664947 -2.637871

H -0.655345 0.081355 -1.842044

C 2.919869 1.340974 -2.568781

H 3.593271 -0.50678 -1.736119

C 1.818212 2.13222 -2.896552

H -0.322948 2.281993 -2.892

H 3.924361 1.701552 -2.772043

O 3.456862 -2.500395 -0.429821

O 4.721481 1.222509 2.67696

O 1.948187 3.366497 -3.470767

O -0.45517 -3.40569 -2.738539

O -4.050572 -3.354294 0.2477

O -4.21067 3.562582 -1.350527

O -1.685855 -0.234628 3.149059

H -2.269987 0.39958 3.587097

H 2.88568 3.566856 -3.580327

H 0.294937 -2.884282 -3.045183

H -4.349335 -2.962625 1.077628

H -5.146486 3.341709 -1.266799

H 3.972814 -2.893874 0.283645

epi-vaticahainolB_Conf25.log

Energy (E) = -1680.35281219 Hartree

Enthalpy (H) = -1679.891401 Hartree

Gibbs free energy (G) = -1679.979762 Hartree

Charge = 0, Spin = 1

C 3.604251 0.760535 2.373485

C 2.362249 1.335336 2.860531

C 1.208269 0.78407 2.434224

C 1.130321 -0.337086 1.51438

C 2.36475 -0.907896 1.014359

C 3.529421 -0.378759 1.43335

H 2.390572 2.175086 3.543991

H 4.477236 -0.768759 1.082055

C -0.17059 -0.638735 1.310033

C -1.015324 0.348278 2.140913

O -0.023599 1.193227 2.783064

C -1.903296 1.22225 1.277699

C -3.289359 1.069605 1.267649

C -1.319416 2.17243 0.439175

C -4.08159 1.853979 0.438997

H -3.759043 0.334711 1.910815

C -2.100806 2.962724 -0.391708

H -0.241407 2.299791 0.42446

C -3.48771 2.802124 -0.394347

H -5.159977 1.738019 0.430521

H -1.638611 3.70118 -1.039986

C -0.793166 -1.633851 0.425213

C -2.081232 -2.077484 0.74915

C -0.149566 -2.085719 -0.750397

C -2.753518 -2.95599 -0.092368

H -2.557841 -1.751736 1.664925

C -0.891308 -2.917171 -1.606901

C -2.168804 -3.36283 -1.282852

H -2.700702 -4.014636 -1.966564

C 2.258005 -2.044953 0.043924

H 1.833483 -2.905874 0.580239

C 1.275263 -1.720686 -1.122288

H 1.59772 -2.437047 -1.885274

C 1.427745 -0.32483 -1.724408

C 2.699087 0.186648 -2.018291

C 0.322598 0.46677 -2.035128

C 2.861434 1.444433 -2.581635

H 3.57833 -0.408143 -1.797501

C 0.468509 1.727327 -2.609117

H -0.679884 0.108467 -1.824728

C 1.740985 2.222178 -2.878606

H 3.851735 1.83162 -2.79813

H -0.407442 2.327454 -2.83898

O 3.551598 -2.368196 -0.421921

O 4.711526 1.200594 2.720438

O 1.950055 3.4547 -3.433176

O -0.415316 -3.366308 -2.807675

O -3.99908 -3.42885 0.191778

O -4.306223 3.545173 -1.191842

O -1.775714 -0.247733 3.13724

H -1.230733 -0.905037 3.590681

H 1.098572 3.883535 -3.582482

H 0.312519 -2.81556 -3.116601

H -4.305376 -3.051836 1.025779

H -3.774439 4.153958 -1.719266

H 3.486099 -3.192232 -0.917416

epi-vaticahainolB_Conf26.log

Energy (E) = -1680.35323843 Hartree

Enthalpy (H) = -1679.891160 Hartree

Gibbs free energy (G) = -1679.979630 Hartree

Charge = 0, Spin = 1

C -3.149177 -1.902413 2.321387

C -1.839181 -2.513827 2.447828

C -0.764555 -1.753251 2.157743

C -0.834985 -0.371396 1.71588

C -2.137816 0.248624 1.589129

C -3.223813 -0.490637 1.886343

H -1.755186 -3.546279 2.764109

H -4.221659 -0.071269 1.823078

C 0.4198 0.077024 1.494874

C 1.389067 -1.091047 1.785775

O 0.513384 -2.165395 2.214183

C 2.129233 -1.550816 0.542163

C 3.47848 -1.257534 0.346174

C 1.43651 -2.242622 -0.453647

C 4.124674 -1.642767 -0.821361

H 4.036731 -0.726174 1.107591

C 2.070374 -2.628947 -1.626405

H 0.385618 -2.482437 -0.324435

C 3.420583 -2.325687 -1.813127

H 5.174606 -1.417154 -0.973808

H 1.523601 -3.166733 -2.395381

C 0.897158 1.355817 0.948293

C 2.224133 1.717365 1.218533

C 0.094231 2.159299 0.104707

C 2.782081 2.847129 0.634129

H 2.82352 1.121429 1.894327

C 0.725601 3.243131 -0.532613

C 2.042318 3.599612 -0.267764

H 2.481699 4.454084 -0.769657

C -2.205022 1.685557 1.147996

H -1.762291 2.302697 1.93811

C -1.39124 1.973216 -0.148446

H -1.778164 2.962837 -0.411366

C -1.731476 1.038881 -1.301499

C -2.92463 1.213426 -2.01261

C -0.897062 -0.014008 -1.680538

C -3.294913 0.344613 -3.032153

H -3.571423 2.055253 -1.780731

C -1.249174 -0.89073 -2.699012

H 0.058135 -0.15589 -1.188732

C -2.459494 -0.719626 -3.37071

H -4.221943 0.489374 -3.576832

H -0.582786 -1.703787 -2.974841

O -3.535364 2.1412 1.019973

O -4.193446 -2.526685 2.566178

O -2.864735 -1.551578 -4.37539

O 0.077712 4.03913 -1.437912

O 4.060973 3.242956 0.884974

O 4.099025 -2.674066 -2.942238

O 2.297728 -0.848822 2.804878

H 1.832834 -0.435102 3.544689

H -2.196762 -2.234637 -4.510989

H -0.659012 3.564577 -1.840089

H 4.476254 2.625018 1.499204

H 3.505305 -3.137961 -3.545266

H -3.981513 1.59792 0.357458

epi-vaticahainolB_Conf27.log

Energy (E) = -1680.35312254 Hartree

Enthalpy (H) = -1679.891329 Hartree

Gibbs free energy (G) = -1679.979501 Hartree

Charge = 0, Spin = 1

C -3.147447 -1.911856 2.322403

C -1.83796 -2.525597 2.445435

C -0.762629 -1.765816 2.155474

C -0.831248 -0.382517 1.717904

C -2.13342 0.239528 1.594774

C -3.220173 -0.499136 1.890598

H -1.754726 -3.558941 2.758915

H -4.217359 -0.077978 1.828582

C 0.424199 0.064144 1.496309

C 1.391068 -1.108577 1.776975

O 0.514305 -2.180466 2.208776

C 2.118139 -1.565727 0.524737

C 1.416117 -2.261354 -0.464116

C 3.461916 -1.265336 0.312823

C 2.037444 -2.643336 -1.642966

H 0.368138 -2.506173 -0.321663

C 4.097025 -1.646203 -0.864249

H 4.027427 -0.732394 1.067894

C 3.383598 -2.332027 -1.846889

H 1.491725 -3.183284 -2.409328

H 5.145405 -1.411105 -1.022318

C 0.901976 1.34641 0.957818

C 2.228982 1.705364 1.231579

C 0.099114 2.15862 0.122389

C 2.786675 2.84181 0.66003

H 2.829045 1.102271 1.900384

C 0.72936 3.251436 -0.500359

C 2.046104 3.605041 -0.231909

H 2.484806 4.465909 -0.723359

C -2.200425 1.678014 1.158902

H -1.759123 2.292164 1.952194

C -1.385573 1.971696 -0.135274

H -1.773576 2.961844 -0.394066

C -1.723089 1.043813 -1.294407

C -0.886826 -0.007663 -1.678475

C -2.912141 1.224142 -2.008183

C -1.23393 -0.873794 -2.705552

H 0.066334 -0.153581 -1.183991

C -3.277971 0.364327 -3.039301

H -3.560935 2.063482 -1.772808

C -2.439727 -0.69533 -3.384037

H -0.572267 -1.68565 -2.991144

H -4.204578 0.522065 -3.583544

O -3.53106 2.13318 1.031079

O -4.192202 -2.534941 2.567887

O -2.738457 -1.574416 -4.385907

O 0.08101 4.060241 -1.394086

O 4.066036 3.233853 0.914199

O 3.948443 -2.725001 -3.022935

O 2.310307 -0.875624 2.788591

H 1.854417 -0.460873 3.53349

H -3.589682 -1.339776 -4.774677

H -0.654616 3.590983 -1.804254

H 4.479661 2.611111 1.52471

H 4.872782 -2.448358 -3.044706

H -3.974886 1.593211 0.36426

epi-vaticahainolB_Conf28.log

Energy (E) = -1680.35250527 Hartree

Enthalpy (H) = -1679.891569 Hartree

Gibbs free energy (G) = -1679.979426 Hartree

Charge = 0, Spin = 1

C 4.077281 1.666461 0.934005

C 3.06219 1.787169 1.964583

C 1.996327 0.964086 1.890159

C 1.804425 -0.034915 0.853694

C 2.759374 -0.089663 -0.235166

C 3.839768 0.712811 -0.1704

H 3.188014 2.508525 2.762691

H 4.568344 0.724852 -0.973351

C 0.69173 -0.744295 1.145455

C 0.003802 -0.046245 2.328851

O 0.976256 0.9337 2.765367

C -1.237337 0.681962 1.8297

C -2.438661 -0.015492 1.676212

C -1.177744 2.021228 1.442115

C -3.55553 0.606816 1.135465

H -2.503874 -1.057207 1.972276

C -2.293979 2.656869 0.918286

H -0.255515 2.581435 1.543819

C -3.478233 1.942493 0.740292

H -4.484282 0.062899 1.001821

H -2.237768 3.694717 0.605224

C 0.124963 -1.914212 0.456667

C -0.52182 -2.880713 1.231337

C 0.24739 -2.0681 -0.94269

C -1.061922 -4.012066 0.628249

H -0.599985 -2.766531 2.30293

C -0.35143 -3.199268 -1.518359

C -0.98872 -4.169975 -0.746847

H -1.432136 -5.030382 -1.237522

C 2.470631 -0.974211 -1.425981

H 2.754357 -1.99741 -1.155379

C 0.966291 -1.047497 -1.808992

H 1.037091 -1.452443 -2.823119

C 0.172589 0.257553 -1.920434

C 0.745593 1.530463 -1.965608

C -1.220143 0.175085 -2.036929

C -0.03594 2.676019 -2.093267

H 1.817613 1.665888 -1.878449

C -2.012916 1.304998 -2.177562

H -1.705587 -0.795605 -2.010421

C -1.419463 2.566292 -2.189453

H 0.424659 3.65827 -2.10159

H -3.092841 1.213036 -2.247702

O 3.286359 -0.660427 -2.537436

O 5.110341 2.354835 0.942861

O -2.152702 3.717507 -2.272659

O -0.364719 -3.446324 -2.861915

O -1.666686 -4.924055 1.440026

O -4.58178 2.501262 0.167577

O -0.240958 -0.907661 3.384342

H -0.904055 -0.506585 3.961514

H -3.091043 3.496928 -2.225009

H -0.124488 -2.657371 -3.359987

H -2.003091 -5.656756 0.909609

H -4.377594 3.406545 -0.098477

H 3.082175 0.234566 -2.834484

epi-vaticahainolB_Conf29.log

Energy (E) = -1680.35316509 Hartree

Enthalpy (H) = -1679.891241 Hartree

Gibbs free energy (G) = -1679.979401 Hartree

Charge = 0, Spin = 1

C -3.151736 -1.904928 2.321167

C -1.845367 -2.526711 2.437875

C -0.766514 -1.771439 2.149251

C -0.828747 -0.385551 1.719265

C -2.127522 0.244271 1.601255

C -3.217681 -0.489795 1.896156

H -1.767263 -3.56212 2.745811

H -4.212739 -0.063053 1.838288

C 0.42856 0.055877 1.498384

C 1.390126 -1.122442 1.773033

O 0.508323 -2.193329 2.196906

C 2.117713 -1.573633 0.51902

C 3.464865 -1.277485 0.312088

C 1.415248 -2.255649 -0.476579

C 4.09886 -1.647922 -0.866866

H 4.030927 -0.755151 1.074105

C 2.037109 -2.627812 -1.66028

H 0.366161 -2.498517 -0.338785

C 3.384229 -2.318741 -1.859348

H 5.146838 -1.41919 -1.027788

H 1.482611 -3.158221 -2.428868

C 0.911683 1.337262 0.963185

C 2.240491 1.689842 1.236625

C 0.112454 2.153296 0.128139

C 2.80381 2.822896 0.663925

H 2.837459 1.084133 1.905942

C 0.749212 3.239985 -0.498443

C 2.067562 3.58744 -0.230717

H 2.51119 4.444173 -0.724964

C -2.187243 1.68481 1.170721

H -1.740229 2.293762 1.964815

C -1.374579 1.977878 -0.124992

H -1.755178 2.971952 -0.380142

C -1.722448 1.055535 -1.285342

C -0.893192 0.000697 -1.675395

C -2.913318 1.243909 -1.993977

C -1.248868 -0.861261 -2.70301

H 0.061186 -0.150977 -1.184966

C -3.287753 0.388297 -3.025522

H -3.556632 2.086275 -1.754393

C -2.456535 -0.674991 -3.376074

H -0.592465 -1.67579 -2.99315

H -4.215628 0.552233 -3.565749

O -3.515683 2.147725 1.048023

O -4.199319 -2.52344 2.566157

O -2.763997 -1.55021 -4.378722

O 0.103655 4.048999 -1.394733

O 4.084769 3.209482 0.918165

O 4.049222 -2.650965 -3.001372

O 2.308627 -0.898765 2.787554

H 1.852607 -0.488437 3.534839

H -3.615457 -1.310109 -4.763654

H -0.618264 3.572551 -1.820891

H 4.495474 2.585346 1.529218

H 3.446189 -3.098472 -3.607741

H -3.964075 1.611808 0.380991

epi-vaticahainolB_Conf30.log

Energy (E) = -1680.35312939 Hartree
[truncated: 2,563,724 more chars]
